# Supplementary material for: A Fragment-Based Electrophile-First Approach to Target Histidine with Aryl-Fluorosulfates: Application to hMcl‑1
Source: J Med Chem. 2025 Nov 12;68(22):24305–15. doi: 10.1021/acs.jmedchem.5c02199 (PMC12670387; doi:10.1021/acs.jmedchem.5c02199)
Supplement: Supplementary file 2 [file jm5c02199_si_002.pdf]

SUPPLEMENTARY INFORMATION FOR:

A fragment-based electrophile-first approach to target histidine with aryl-fluorosulfates: application to hMcl-1

Giulia Alboreggia[1], Kendall Muzzarelli[2], Zahra Assar[2], and Maurizio Pellecchia[1*]

[1] Division of Biomedical Sciences, School of Medicine, University of California Riverside, 900 University Avenue, Riverside, CA 92521, USA.

[2] Cayman Chemical Co., 1180 E. Ellsworth Road, Ann Arbor, MI 48108.

* Correspondence: Maurizio.Pellecchia@ucr.edu; Tel.: 951- 827 7829

Supplementary information contains:

Figure S1 page S3 **General properties of the fragment library**

Figure S2 page S4 **Stability of fluorosulfates**

Figure S3 page S5 **Summary of primary screening results**

Figure S4 page S6 **Thermal denaturation curves**

Figure S5 page S7 **Mass spectrometry data**

Figure S6 page S8 **Long-range His HSQC NMR data**

Figure S7 page S9 **Backbone NMR data**

Figure S8 page S10 **Displacement measurement by TSA**

Figure S9 page S11 **Synthetic scheme of the hit 2 analogs and purity of 165D9**

Figure S10 page S12 **Dose-response DELFIA inhibition curves**

Figure S11 page S13 **Thermal denaturation data with 165D9 and various mutants**

Figure S12 page S14 **Long-range HSQC NMR spectra with hit compound 2 at various pH values**

Figure S13 page S15-S32 **1D ^{1}H, ^{13}C, ^{19}F NMR spectra of the synthesized compounds.**

Table S1 page S33-S87 **Chemical structures and properties of the 320 fragments in the library**

Table S2 page S88-S89 **Structures and characterization of the positive and negative hits**

Table S3 page S90 **Mass spectrometry data of hMcl-1(172-323) and its mutants**

Table S4 page S91 **Mass spectrometry analyses of the synthesized compounds**

Table S5 page S92-S93 **Data collection and refinement statistics for the complex between hMcl-1(172-323) and fragment hit 2**

PDB coordinates for a model of 165D9 in complex with hMcl-1 computed based on PDB ID 9EFJ pages S94-S142

Supplementary Figure S1: General properties of the fragment library. LogP, number of H-bond acceptors, number of H-bond donors, and number of rotatable bonds distributions of the 320 fragments that compose the library.

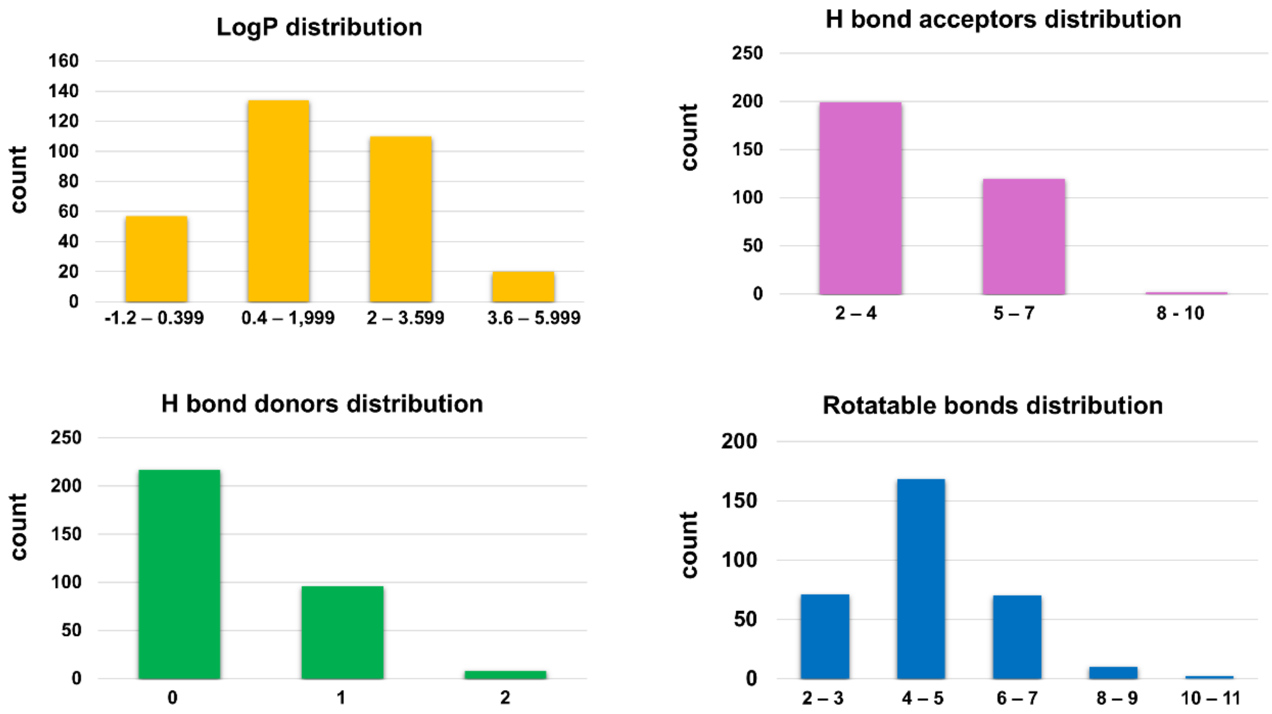

Supplementary Figure S2: Stability of fluorosulfates. Stability was assessed via 1D ^{1}H NMR spectra collected at different time points (0 min, 1 h, 24 h, 48 h).

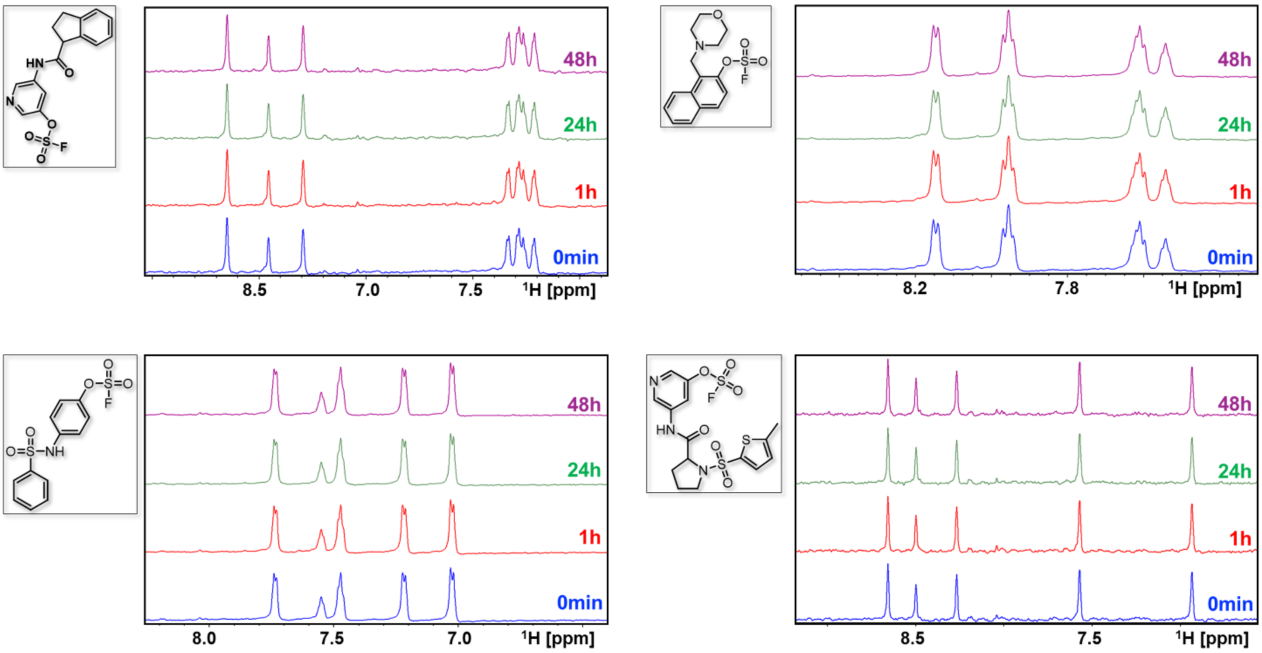

Supplementary Figure S3: Summary of primary screening results. Data based on denaturation thermal shift measurements for the 4 plates of 80 compounds each (320 compounds listed in supplementary **Table S1**). In this screening we considered compounds that induced a sizable positive (> 1.2 ºC, 3 fragments, green) and negative (> -1.2 ºC, 8 fragments, red) for further characterizations.

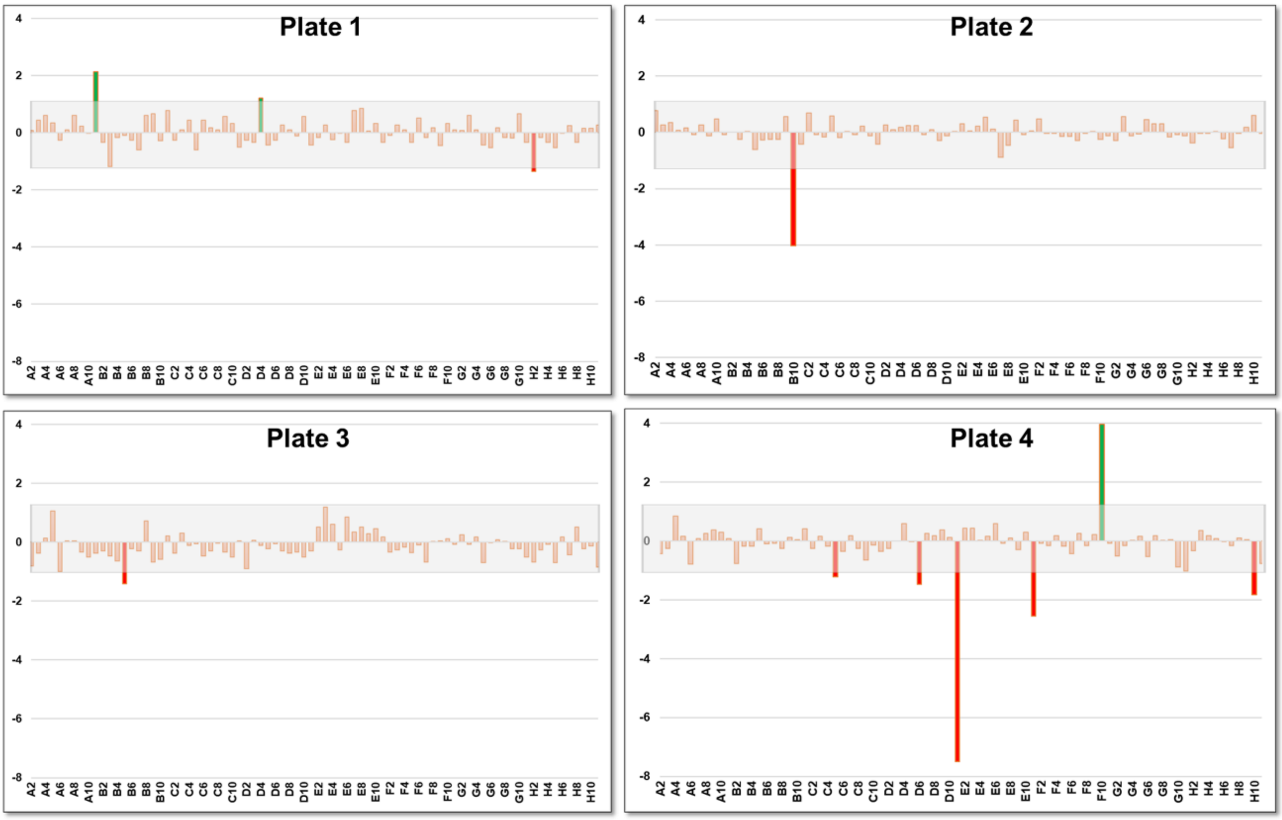

Supplementary Figure S4: Thermal denaturation curves. Denaturation curves for hMcl-1(172-323) or mutant proteins, as indicated, collected in absence and presence of fragment hit **1** (A) or fragment hit **2** (B). **A)** ΔTm (*wt*-hMcl-1) = 2.11 ± 0.01; ΔTm (hMcl-1 H224A) = -0.34 ± 0.09; ΔTm (hMcl-1 H252A) = 2.90 ± 0.17; ΔTm (hMcl-1 K234A) = 1.96 ± 0.17. **B)** ΔTm (*wt*-hMcl-1) = 3.97 ± 0.17; ΔTm (hMcl-1 H224A) = -1.28 ± 0.17; ΔTm (hMcl-1 H252A) = 6.57 ± 0.26; ΔTm (hMcl-1 K234A) = 4.77 ± 0.26.

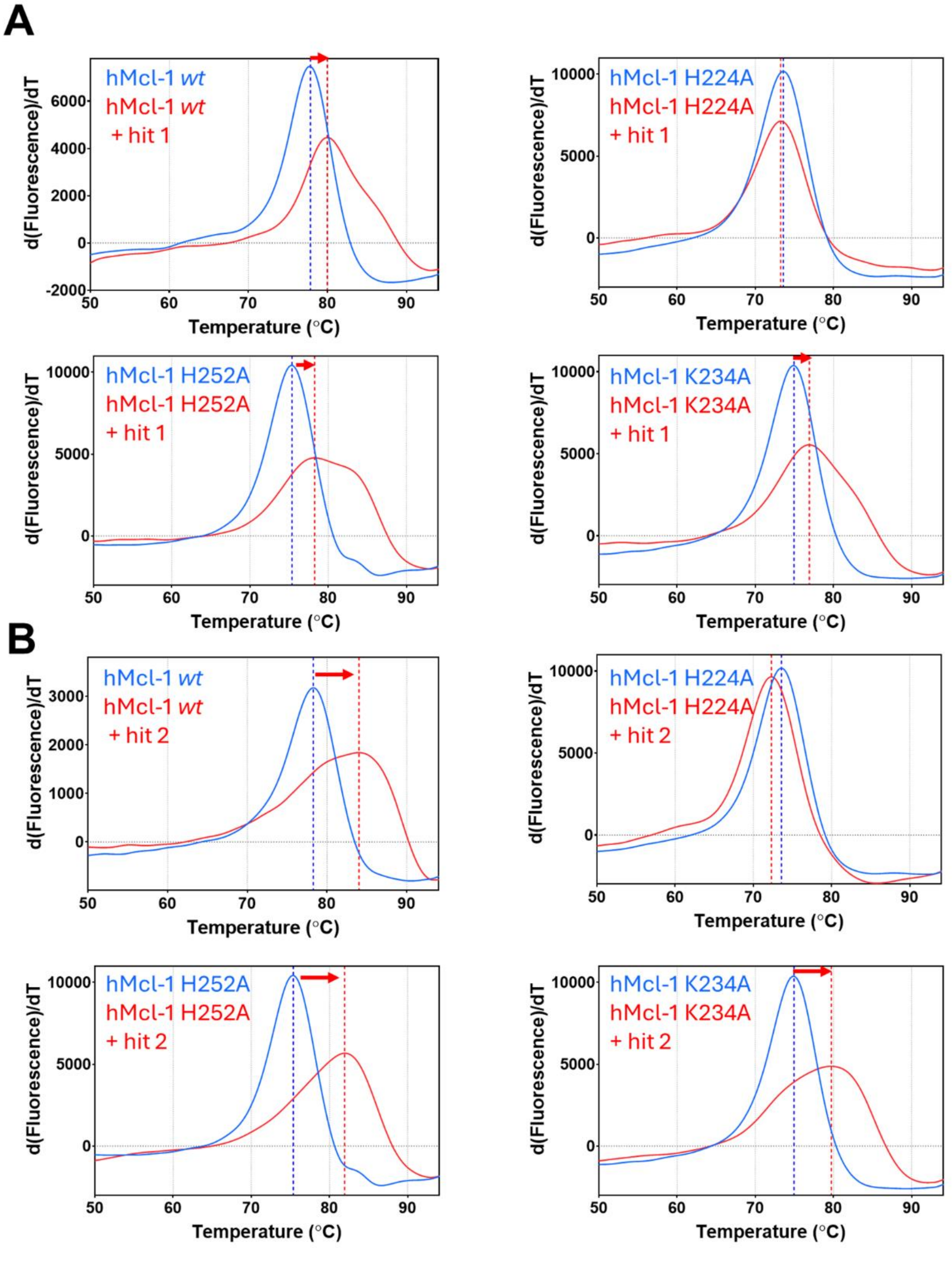

Supplementary Figure S5: Mass spectrometry data. MS analyses of hMcl-1(171-323) and mutants, as indicated, collected in presence of fragment hit **1** (A) or fragment hit **2** (B).

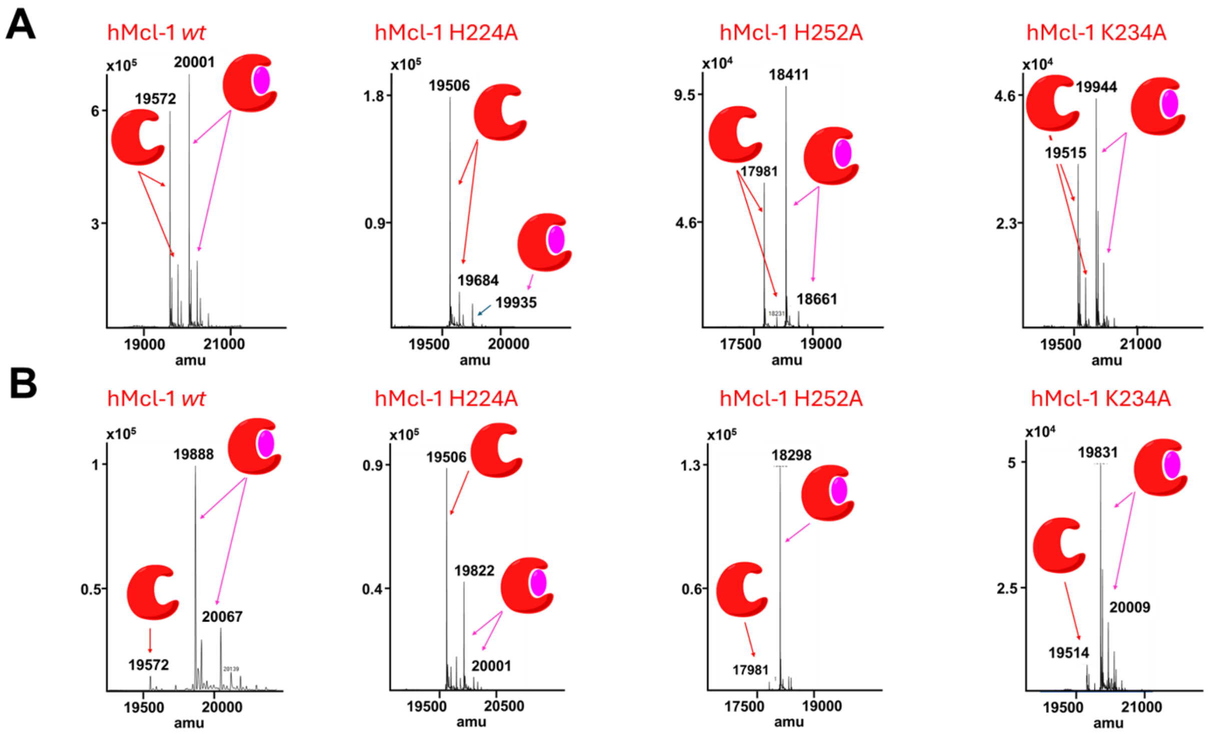

Supplementary Figure S6: Long range His HSQC NMR data. Heteronuclear long range [^{15}N,^{1}H] NMR correlation spectra for His side chains detection. The spectra for hMcl-1(172-323) His252Ala, collected in absence (blue) or presence of fragment hit **2** after various incubation times (red, 1 h; yellow, 25 h) are superimposed. On the right, a zoomed region highlighting the chemical shift perturbations at His 224 is displayed.

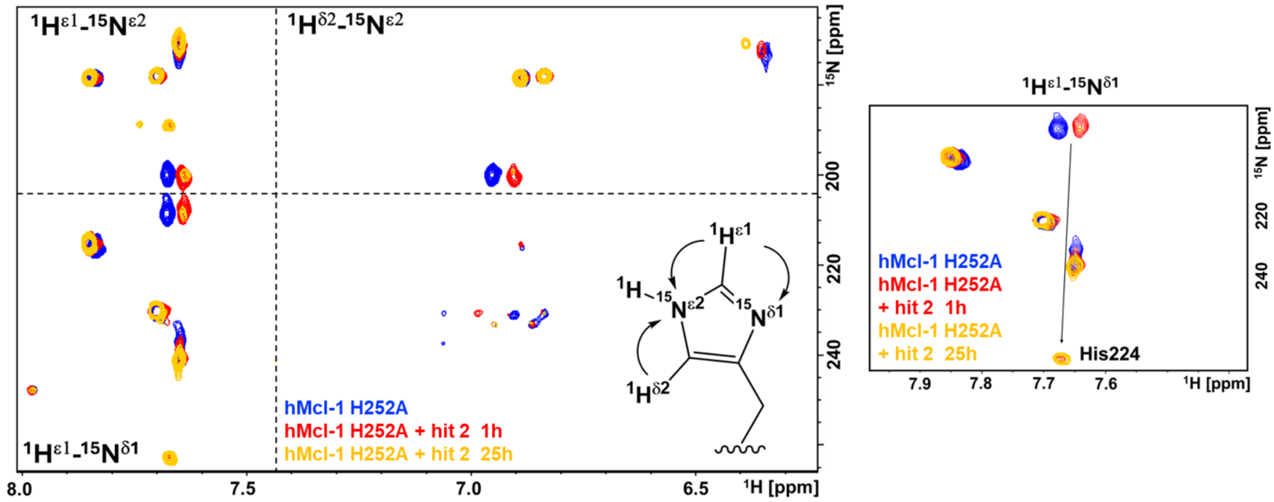

Supplementary Figure S7: Backbone NMR data. Backbone 2D [^{15}N,^{1}H] correlation spectra for ^{15}N-hMcl-1(172-323) (50 μM) collected in absence (blue) or presence of fragment hit **2** (1 mM) after various incubation times (red, 1 h; green, 25 h). Resonance assignments are also reported.

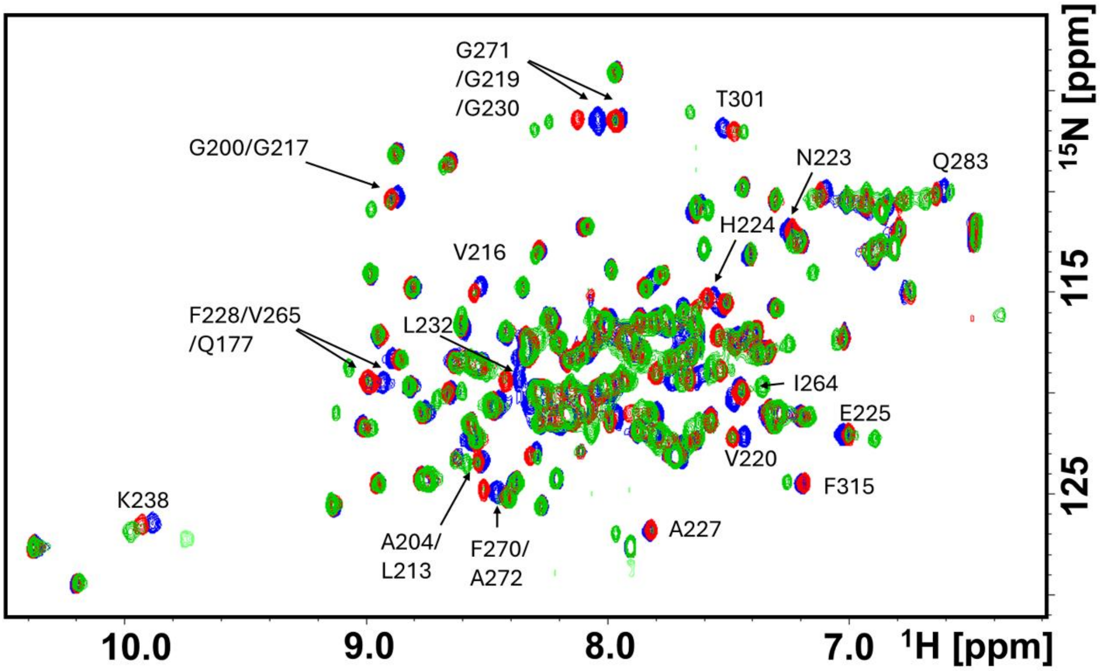

Supplementary Figure S8: Displacement measurement by TSA. Thermal denaturation curves for hMcl-1(172-323) collected in absence (blue) or presence of 200 µM of hit **2**, after 48 h incubation (red), 200 µM of hit **2**, (48 h incubation) plus an additional 2 h with 20 µM of compound **6** (added later) (magenta), or 20 µM of compound **6** (incubation 2 h) (orange). Tm (*wt*-hMcl-1) = 77.62 ± 0.05; ΔTm (hMcl-1+ hit **2** 2d) = 5.81 ± 0.05; ΔTm (hMcl-1+hit **2** 2d+comp. **6** 2h) = 7.08 ± 0.05; ΔTm (hMcl-1+comp. **6** 2h) = 22.09 ± 0.01.

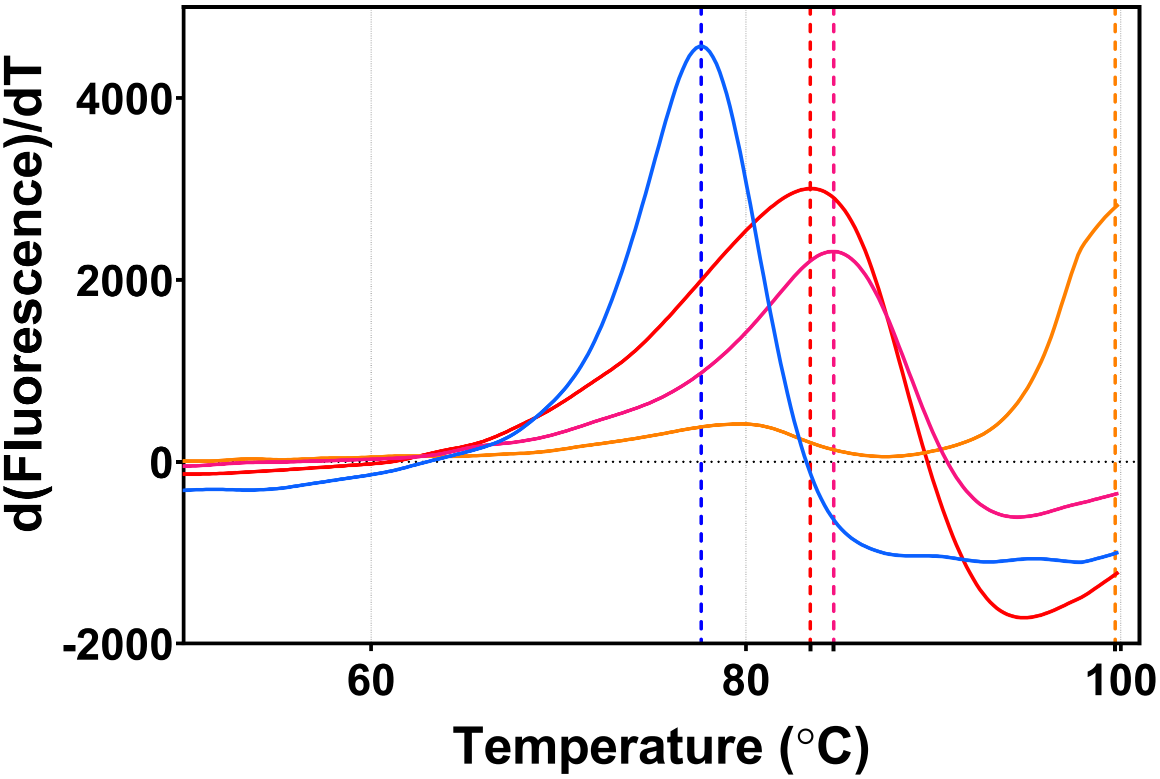

Supplementary Figure S9: Synthetic scheme and purity. Synthetic route used to obtain hit **2** analogs reported in **Figure 6**. The purity of **165D9** by HPLC is also reported in panel F (purity > 98%).

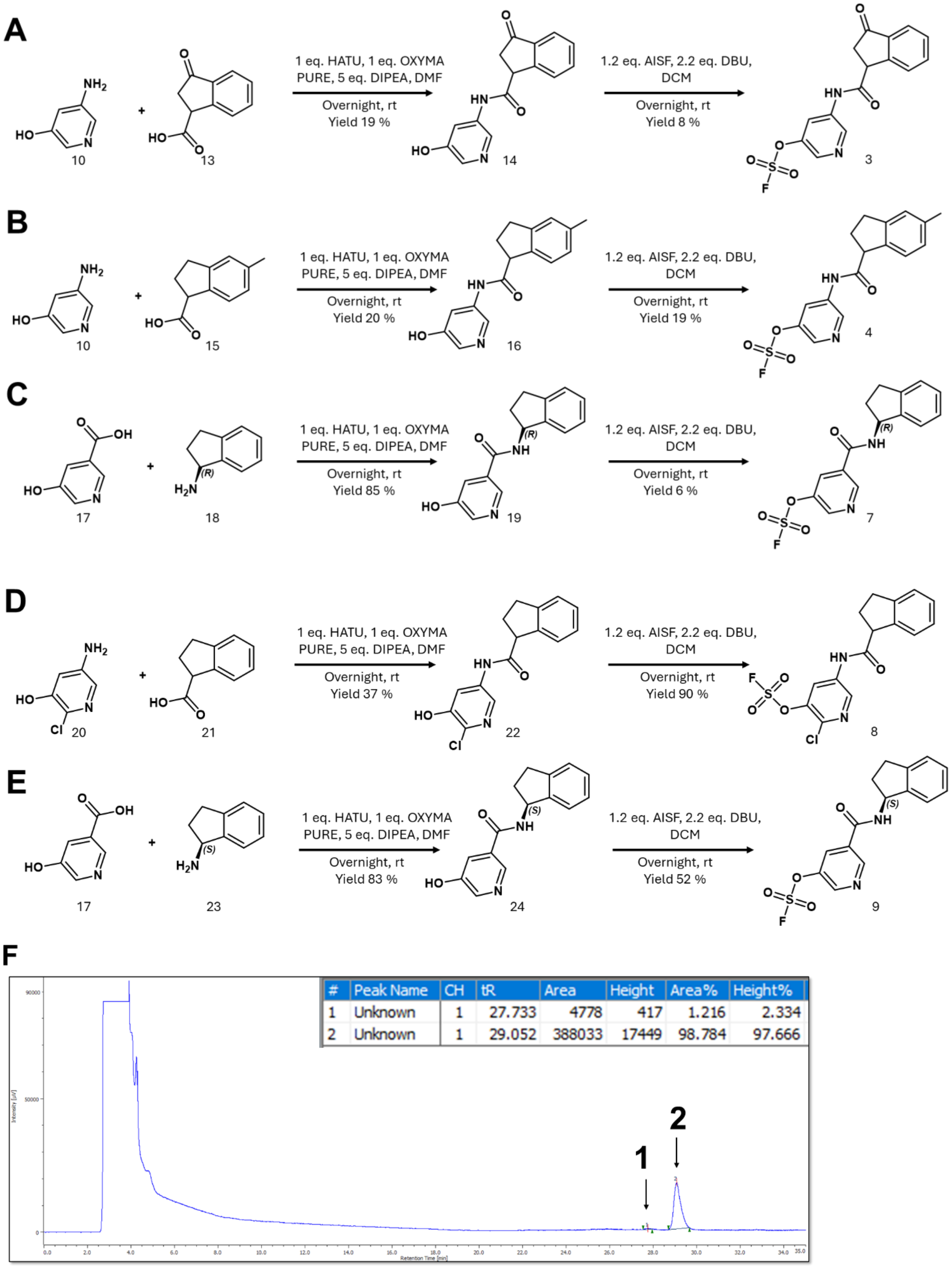

#	Peak Name	CH	tR	Area	Height	Area%	Height%
1	Unknown	1	27.733	4778	417	1.216	2.334
2	Unknown	1	29.052	388033	17449	98.784	97.666

Supplementary Figure S10: Dose-response DELFIA inhibition curves. DELFIA data with compound **165D9** after 24 h incubation (IC_{50} ~ 3.0 μM; this experiment is an independent replicate of the data reported in **Figure 6D** that resulted in an IC_{50} value of ~ 2.5 μM) or 4 h incubation (IC_{50} ~ 12 μM).

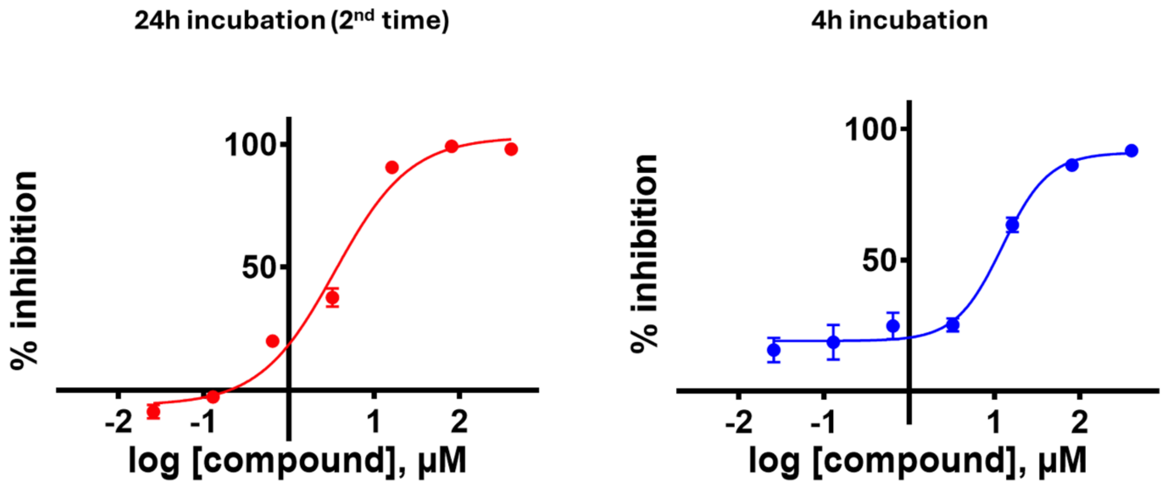

Supplementary Figure S11: Thermal denaturation data with 165D9 and various mutants. A) Thermal denaturation curves for hMcl-1(172-323) or its mutants as indicated, collected in absence (blue) or presence of (250 µM) **165D9**, after 8 h incubation. ΔTm (*wt*-hMcl-1) = 10.23 ± 0.05; ΔTm (hMcl-1 H224A) = -1.15 ± 0.04; ΔTm (hMcl-1 H252A) = 10.44 ± 0.06; ΔTm (hMcl-1 K234A) = 9.96 ± 0.20.

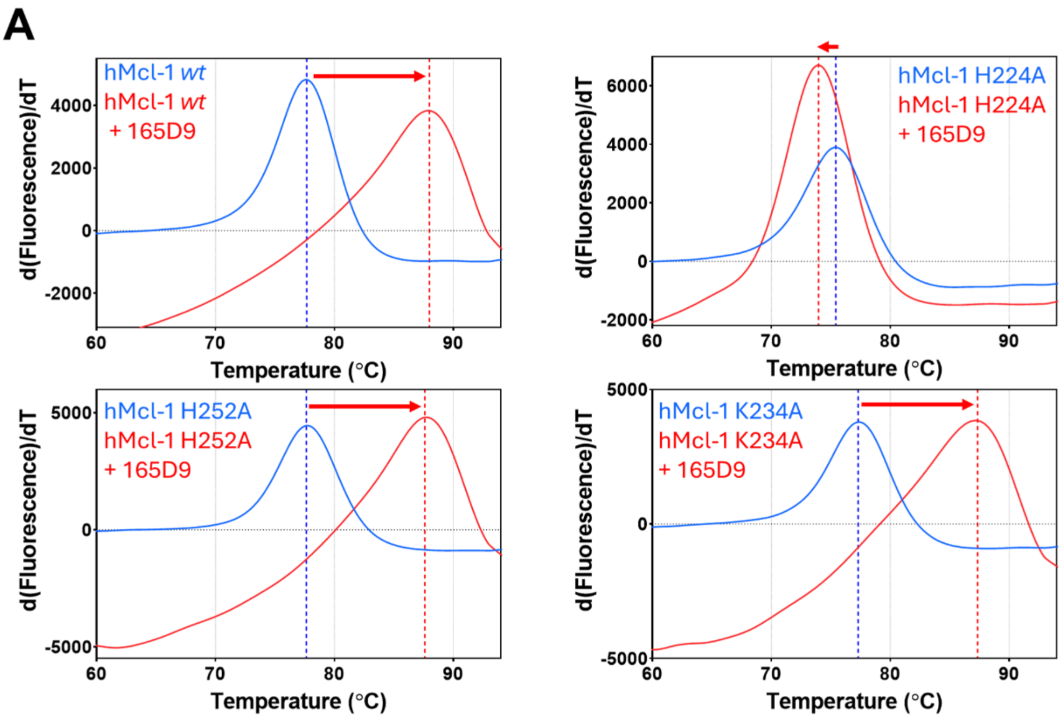

Supplementary Figure S12: Long range HSQC NMR spectra with hit compound 2 at various pH values. A) 2D long-range $[^{15}N, ^{1}H]$ correlation spectra for His side chains of ^{15}N-hMcl-1(172-323) (50 μM) collected at various pH values, as indicated. **B**) 2D long-range $[^{15}N,^{1}H]$ correlation spectra for His side chains of hMcl-1(172-323) (50 μM) collected at pH= 6.5 in absence (blue) or presence of fragment hit **2** (1 mM) after various incubation times (red, 1 h; green, 42 h).

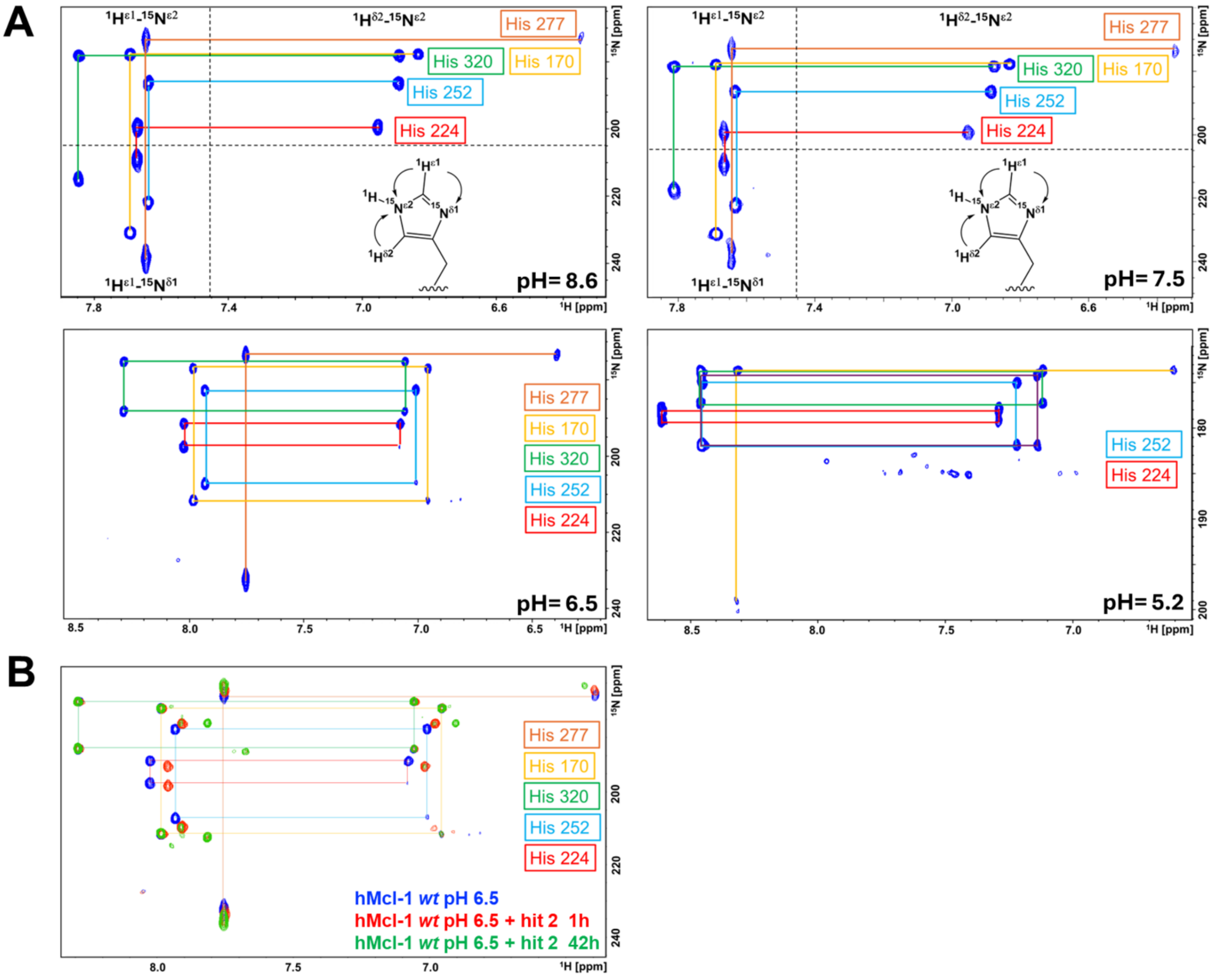

Supplementary Figure S13. **1D ^{1}H, ^{13}C, ^{19}F NMR data of synthesized compounds**. ^{1}H and ^{13}C NMR spectra were acquired on a Bruker Avance III 700 MHz spectrometer equipped with a TCI cryoprobe. ^{19}F NMR spectra were recorded on a Bruker Avance 600 MHz spectrometer. All NMR data were processed and analyzed using TopSpin 4.1.0 (Bruker, Billerica, MA).

Compound 3 (165D6): ^{1}H NMR (700 MHz, in deuterated methanol, MeOD D4): δ 2.64 (s), 2.68 (s), 2.97 (d, J = 7.62 Hz), 3.00 (d, J = 7.85 Hz), 3.05 (s), 3.07 (s), 7.55 (t, J = 7.12 Hz), 7.73 (s), 7.79 (d, J = 7.57 Hz), 8.49 (s), 8.83 (s).

^{13}C NMR (700 MHz, in deuterated DMSO, DMSO D6): δ 30.30, δ 45.72, δ 119.20, δ 123.66, δ 126.74, δ 129.23, δ 135.60, δ 137.19, δ 137.32, δ 141.71, δ 146.88, δ 152.98, δ 172.05, δ 204.85.

^{19}F NMR (600 MHz, in deuterated DMSO, DMSO D6): δ 39.66.

^{1}H NMR:

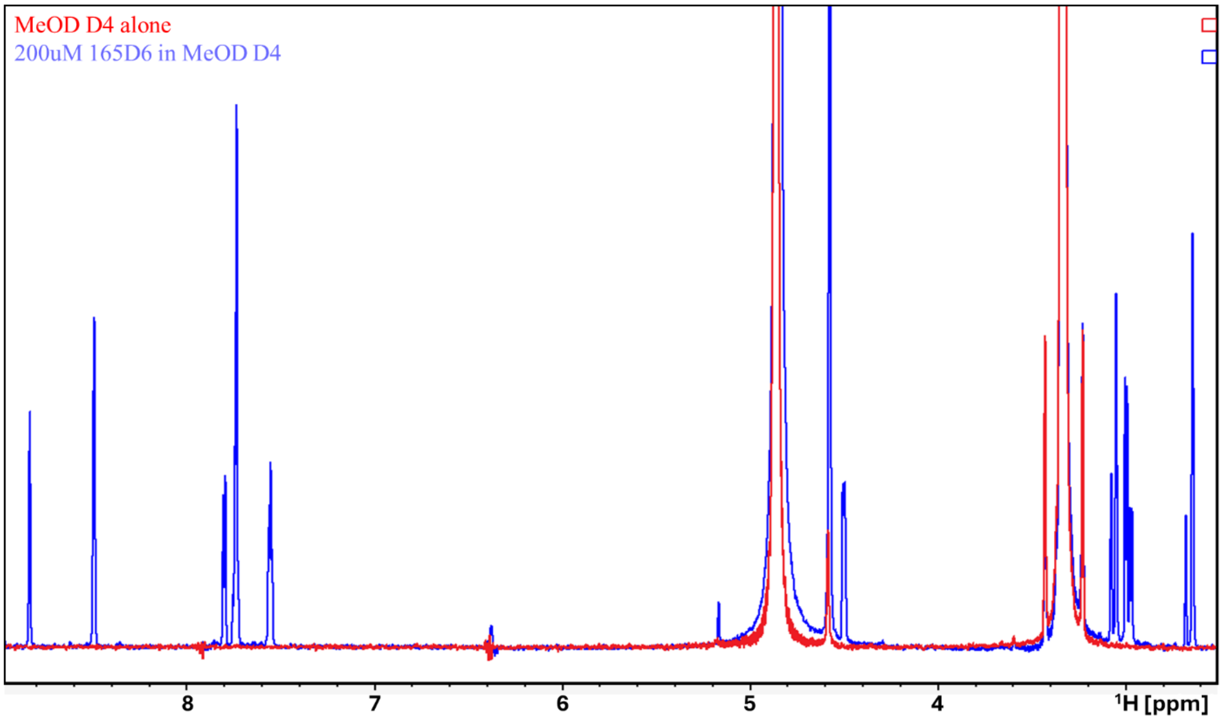

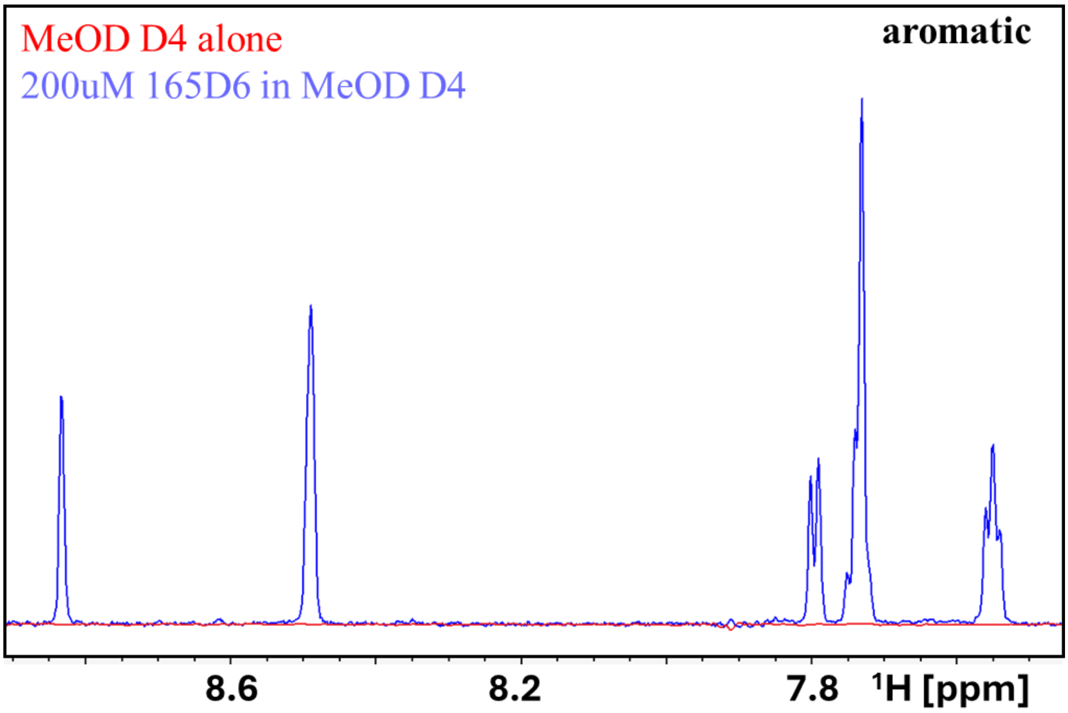
MeOD D4 alone
200uM 165D6 in MeOD D4
aromatic
8.6
8.2
7.8
¹H [ppm]

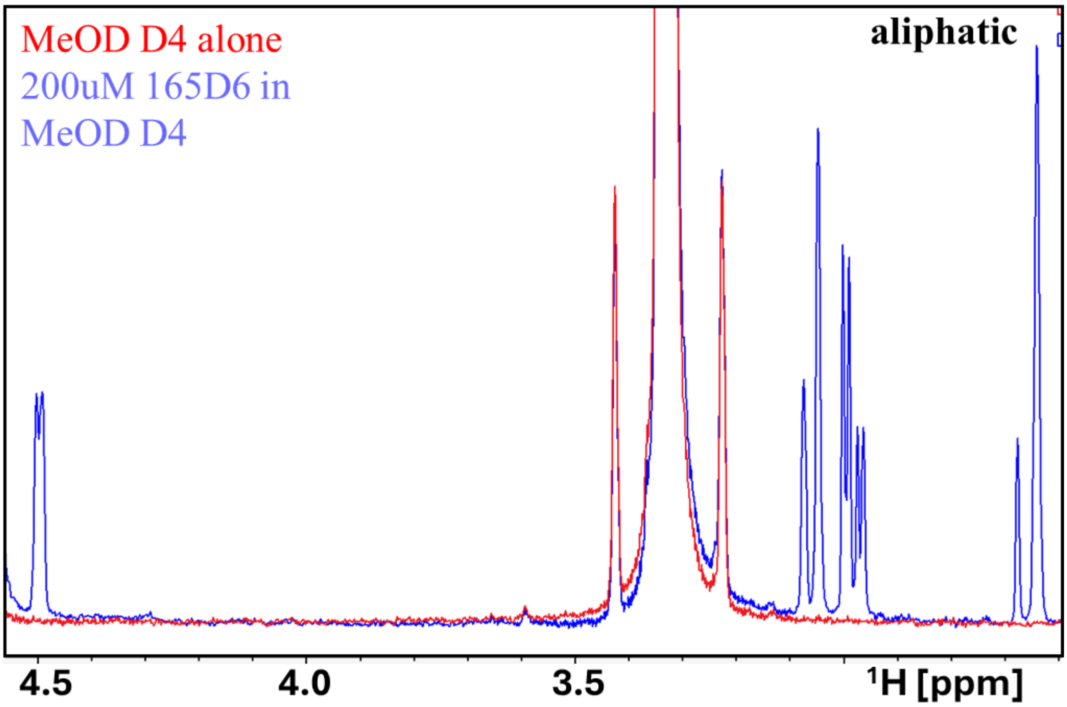
MeOD D4 alone
200uM 165D6 in
MeOD D4
aliphatic
4.5
4.0
3.5
¹H [ppm]

^{13}C NMR:

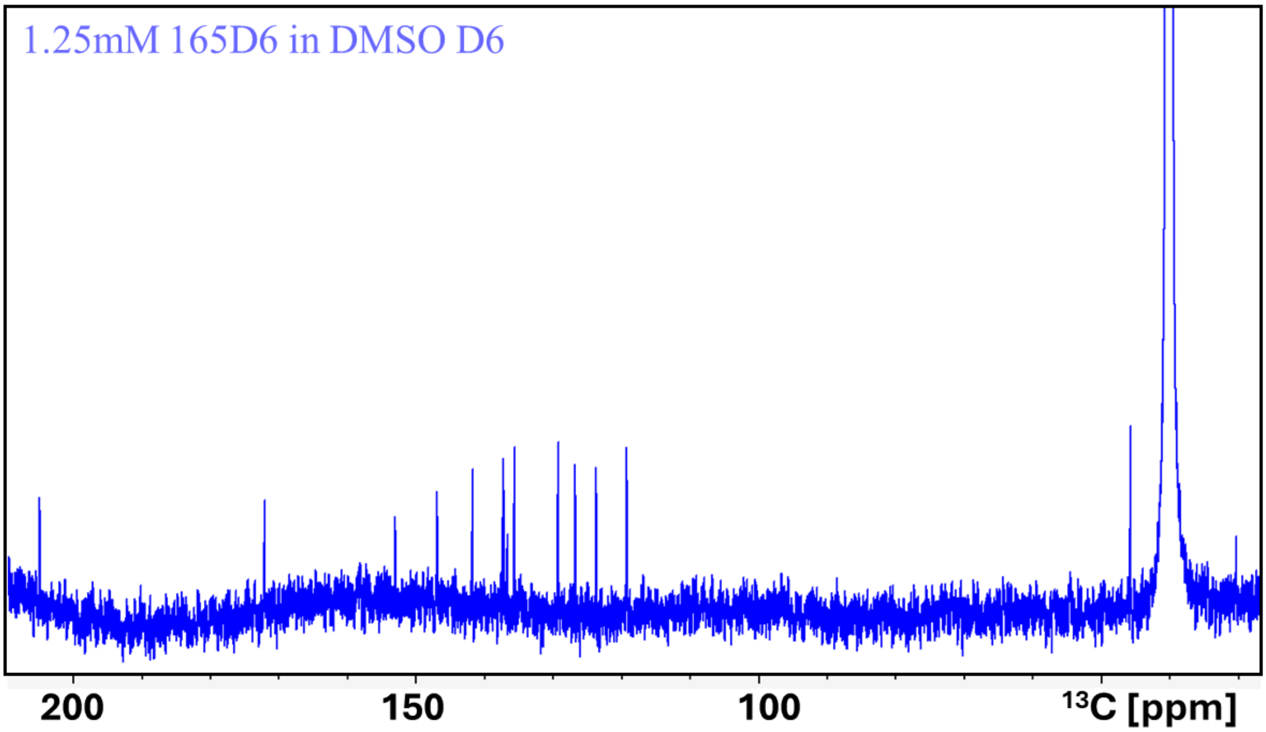

^{19}F NMR:

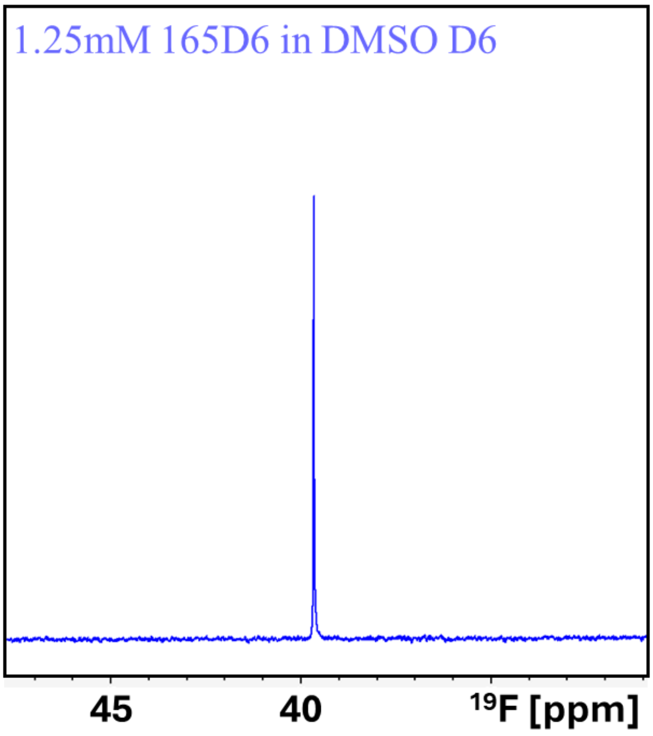

Compound 4 (165D7): H NMR (MeOD D4, 700 MHz): δ 2.33 (s), 2.43 (q, J = 7.1 Hz), 2.64 (s), 2.95 (m), 3.13 (m), 4.14 (t, J = 7.35 Hz), 7.01 (d, J = 7.83 Hz), 7.12 (s), 7.18 (d, J = 7.50 Hz), 8.46 (s), 8.51 (s), 8.81 (s).

^{13}C NMR (700 MHz, in deuterated DMSO, DMSO D6): δ 21.34, δ 29.26, δ 31.95, δ 51.65, δ 118.82, δ 124.39, δ 125.61, δ 127.51, δ 136.68, δ 136.98, δ 137.68, δ 139.33, δ 141.52, δ 144.71, δ 146.92, δ 173.98.

^{19}F NMR (600 MHz, in deuterated DMSO, DMSO D6): δ 39.59.

^{1}H NMR:

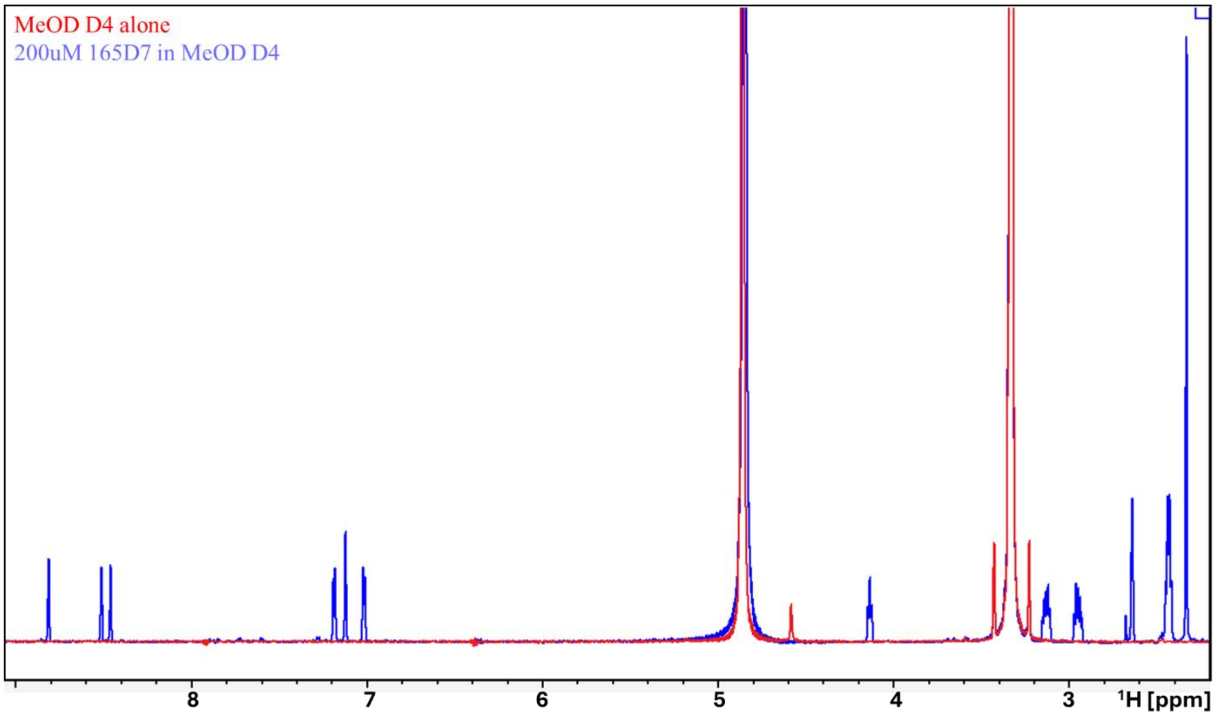

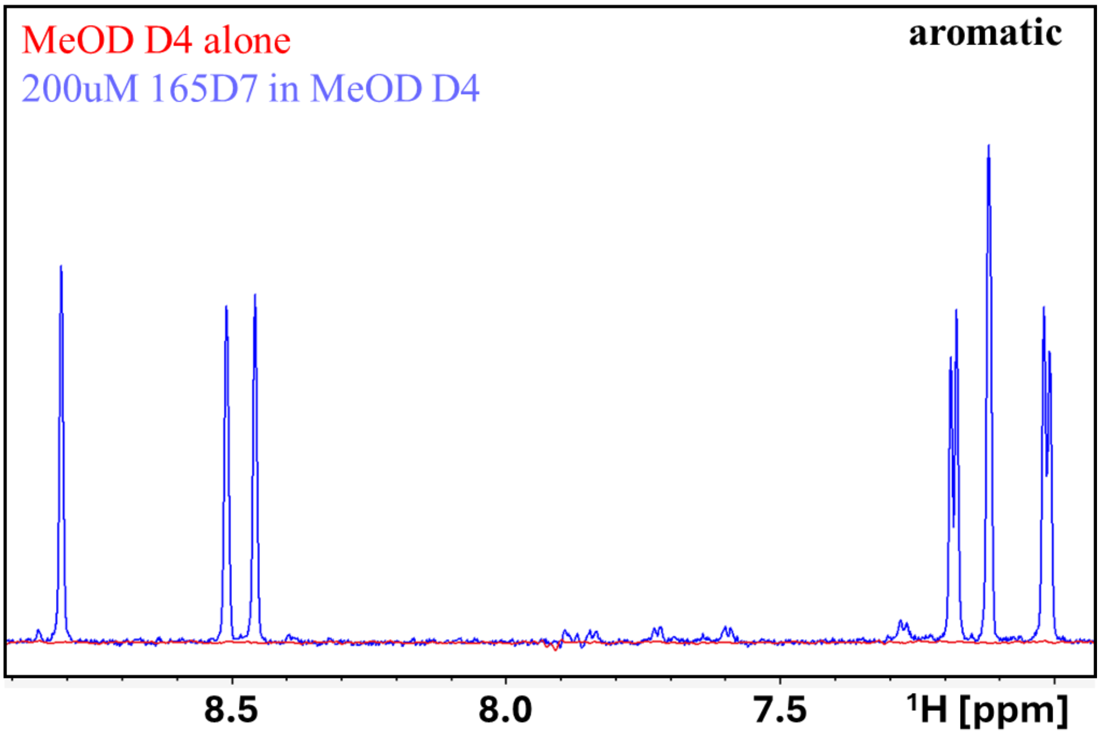
MeOD D4 alone
200uM 165D7 in MeOD D4
aromatic
8.5
8.0
7.5
¹H [ppm]

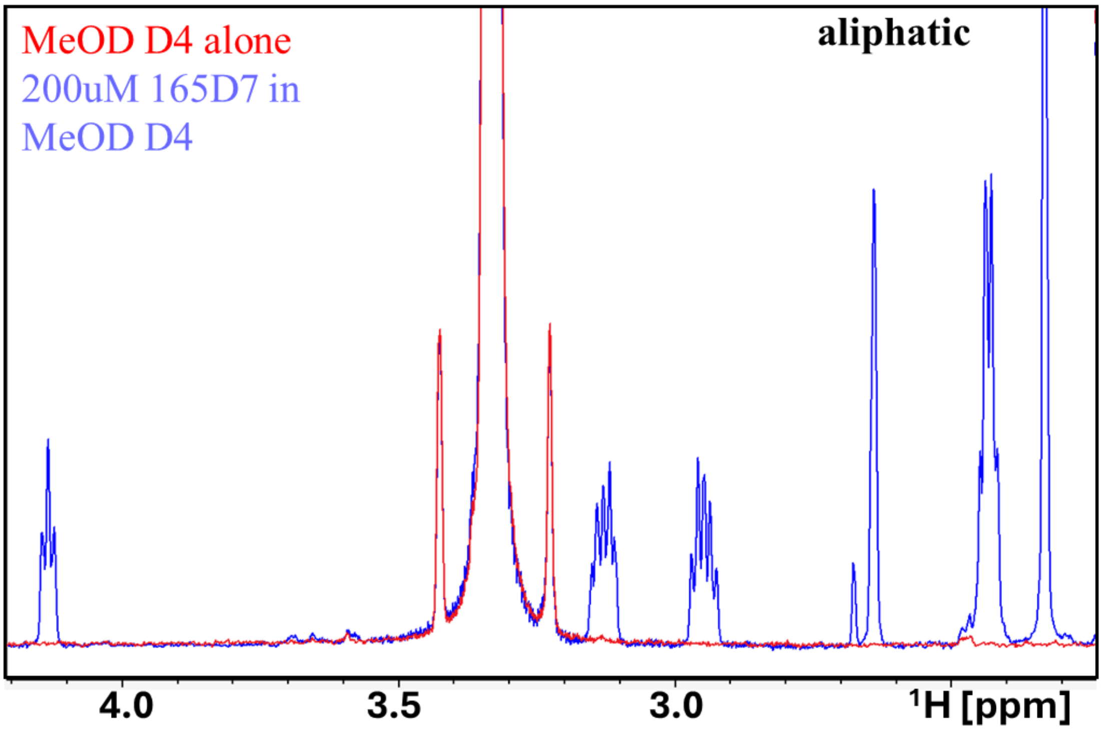
MeOD D4 alone
200uM 165D7 in
MeOD D4
aliphatic
4.0
3.5
3.0
¹H [ppm]

^{13}C NMR:

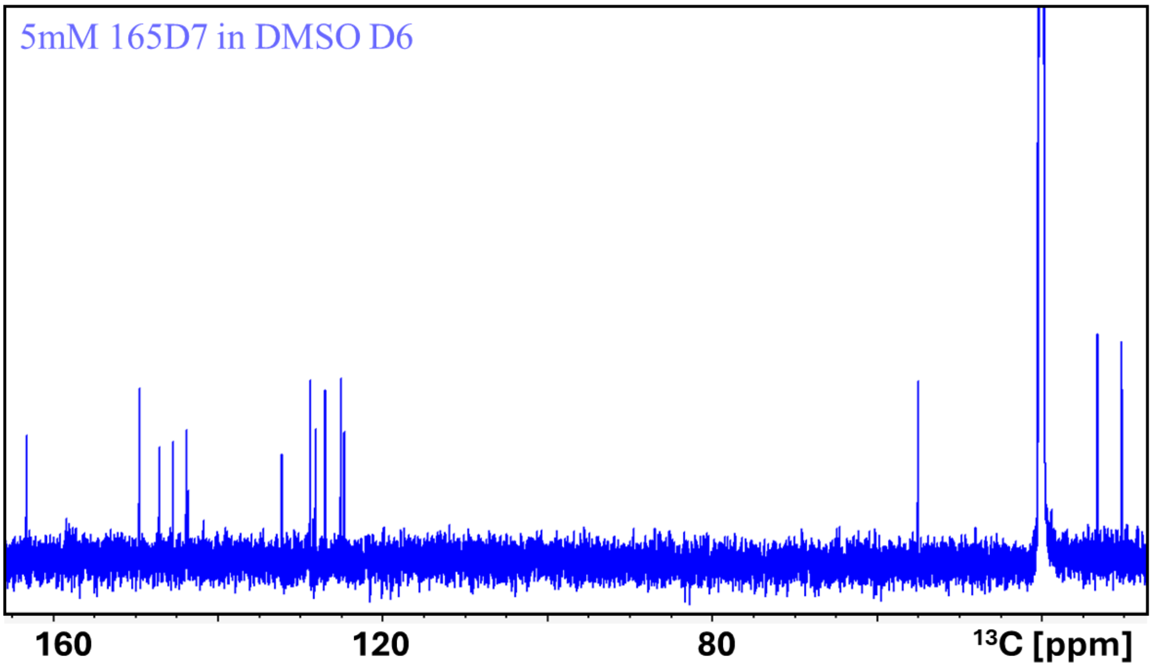

^{19}F NMR:

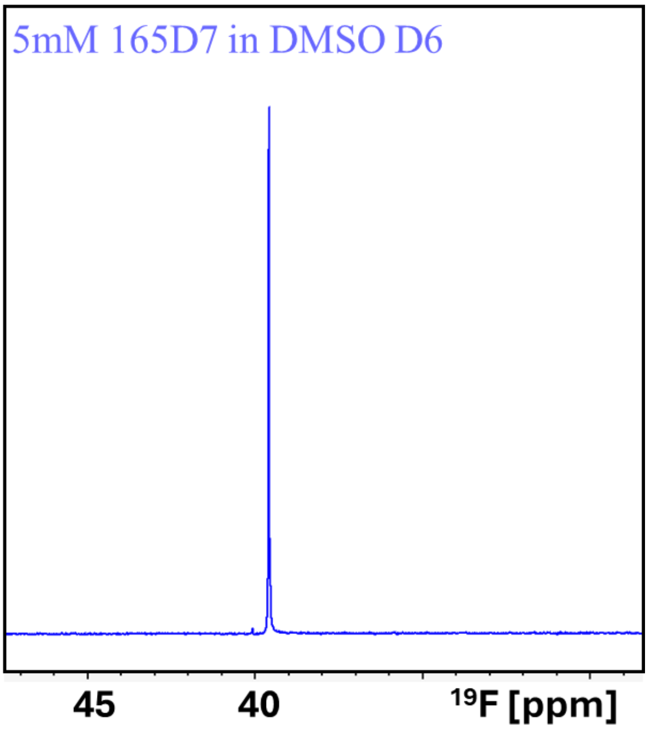

Compound 5 (165D9): H NMR (MeOD D4, 700 MHz): δ 2.48 (m), 2.64 (s), 2.97 (m), 3.14 (m), 4.14 (s), 4.19 (t, J = 6.91 Hz), 7.24 (d, J = 8.08 Hz), 7.28 (d, J = 7.54 Hz), 7.32 (s), 8.47 (s), 8.52 (s), 8.82 (s).

^{13}C NMR (700 MHz, in deuterated DMSO, DMSO D6): δ 29.78, δ 31.54, δ 51.91, δ 119.02, δ 124.92, δ 126.61, δ 127.76, δ 131.25, δ 136.84, δ 147.54, δ 141.63, δ 143.81, δ 144.59, δ 146.91, δ 173.22.

^{19}F NMR (600 MHz, in deuterated DMSO, DMSO D6): δ 39.61.

^{1}H NMR:

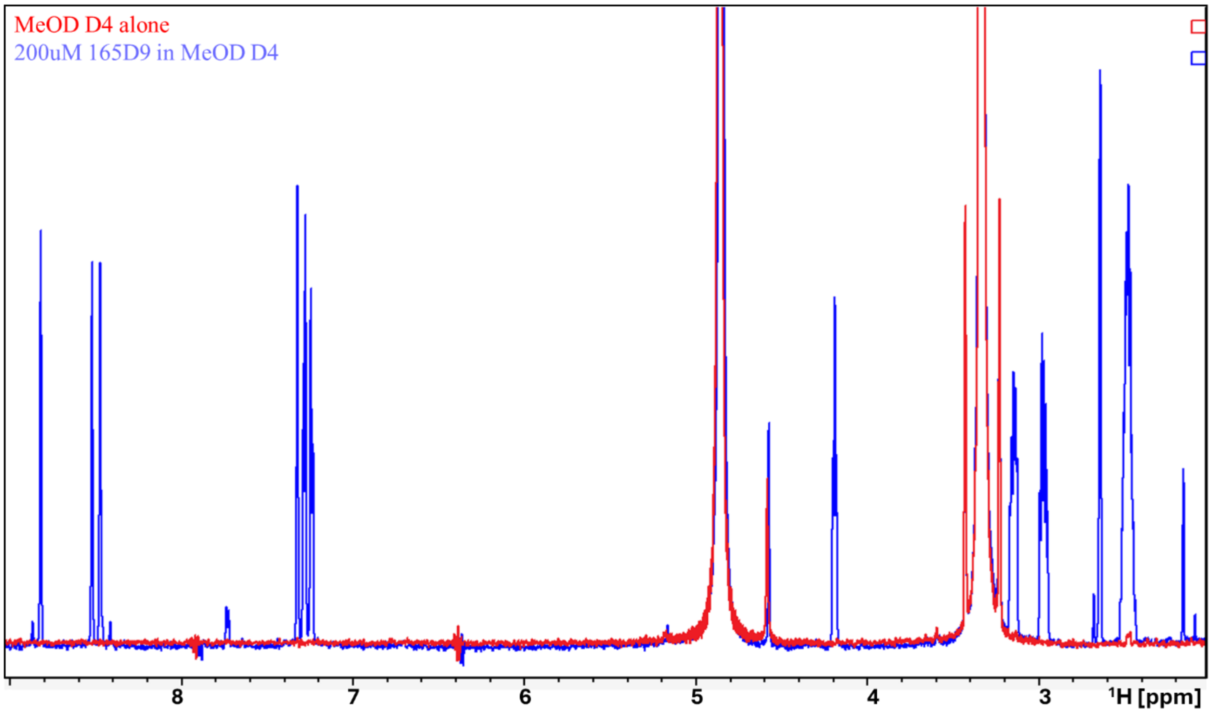

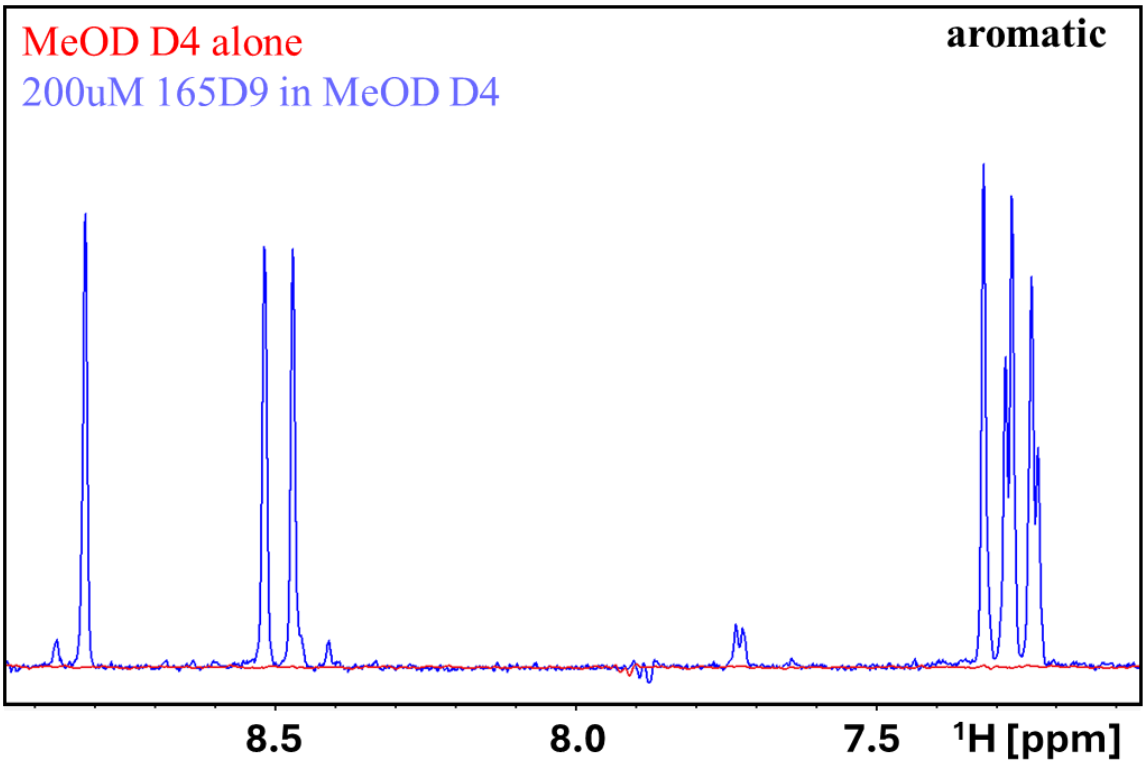

MeOD D4 alone
200uM 165D9 in MeOD D4
aromatic
8.5
8.0
7.5
^{1}H [ppm]

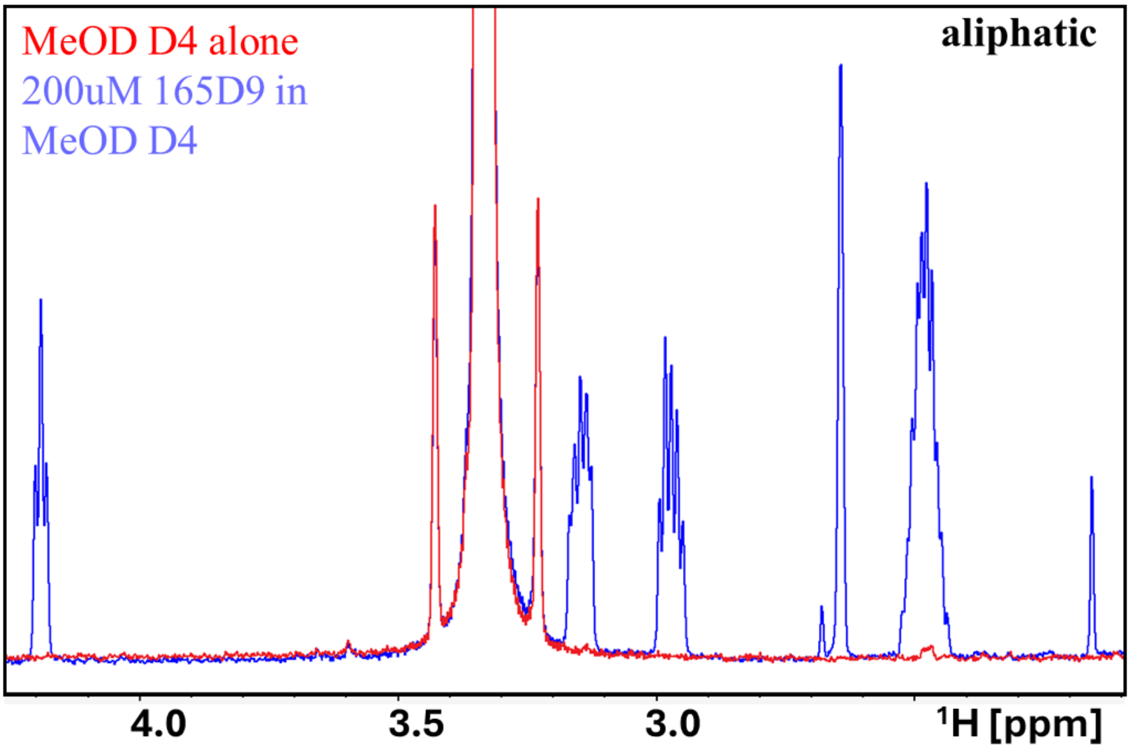

MeOD D4 alone
200uM 165D9 in MeOD D4
aliphatic
4.0
3.5
3.0
^{1}H [ppm]

^{13}C NMR:

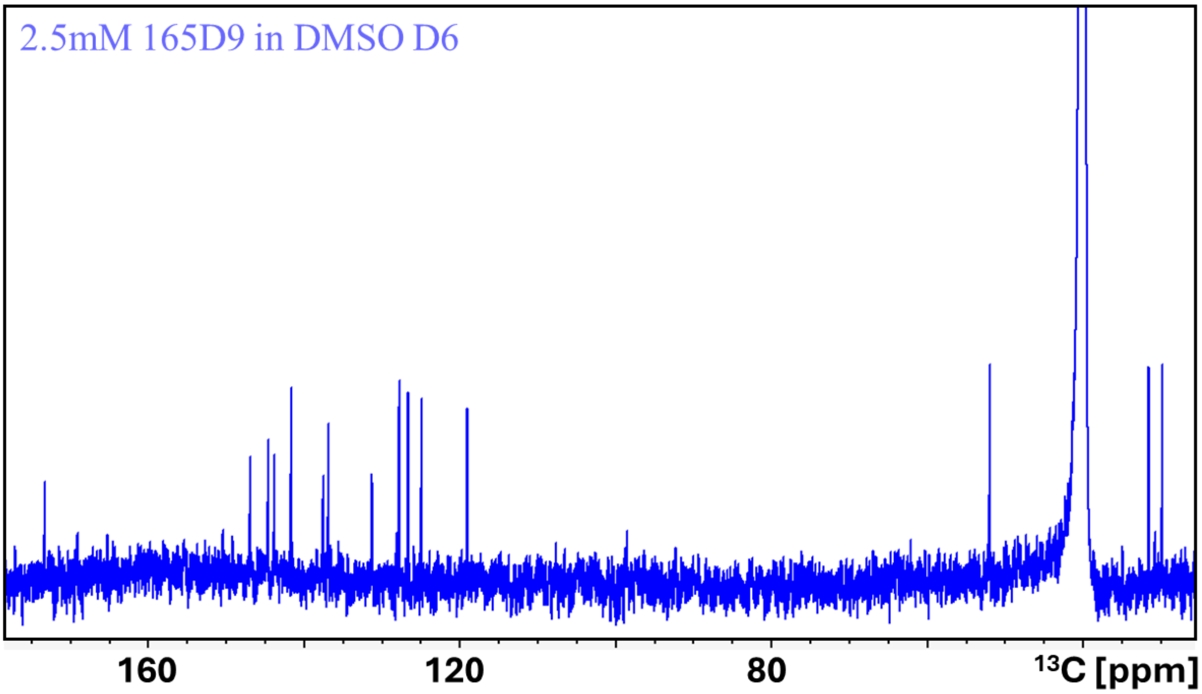

^{19}F NMR:

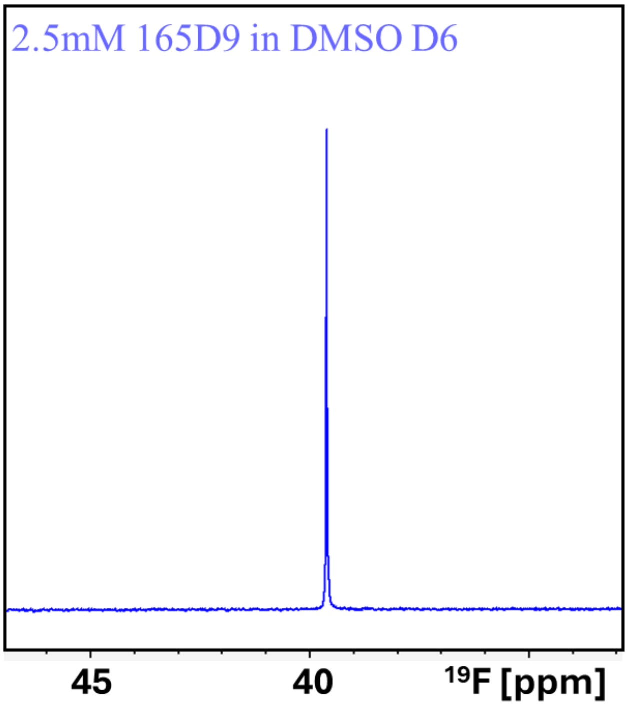

Compound 7 (165D12): H NMR (MeOD D4, 700 MHz): δ 2.07 (m), 1.18 (s), 2.64 (s), 2.96 (m), 3.11 (m), 5.68 (t, J = 7.77 Hz), 7.24 (m), 7.30 (d, J = 6.82 Hz), 7.36 (d, J = 7.60 Hz), 8.43 (s), 8.91 (s), 9.16 (s).

^{13}C NMR (700 MHz, in deuterated DMSO, DMSO D6): δ 30.38, δ 33.26, δ 55.02, δ 124.64, δ 125.05, δ 126.95, δ 128.11, δ 128.73, δ 132.21, δ 143.59, δ 143.78, δ 145.39, δ 147.08, δ 149.51, δ 163.19.

^{19}F NMR (600 MHz, in deuterated DMSO, DMSO D6): δ 40.28.

^{1}H NMR:

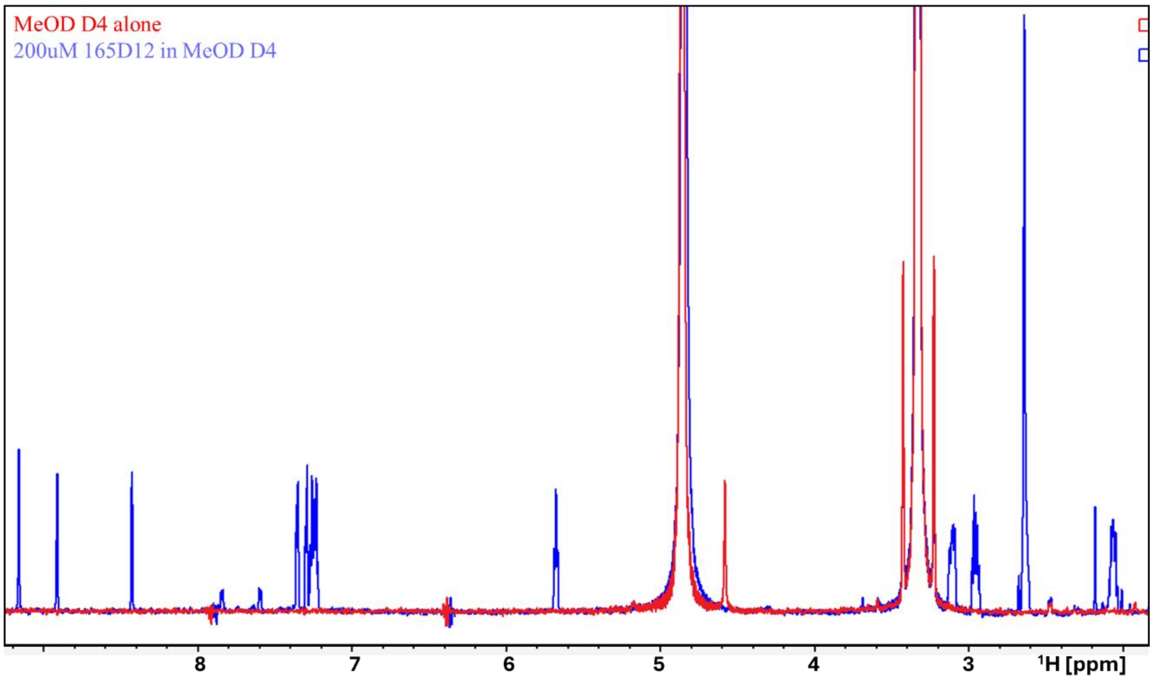

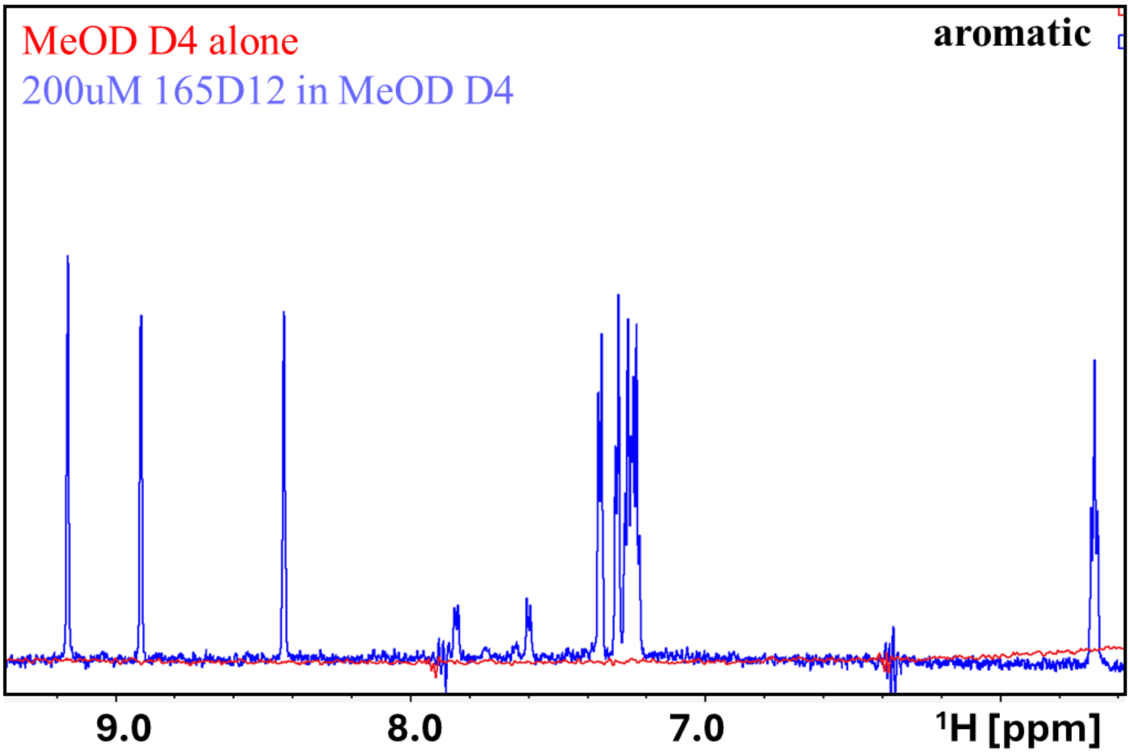

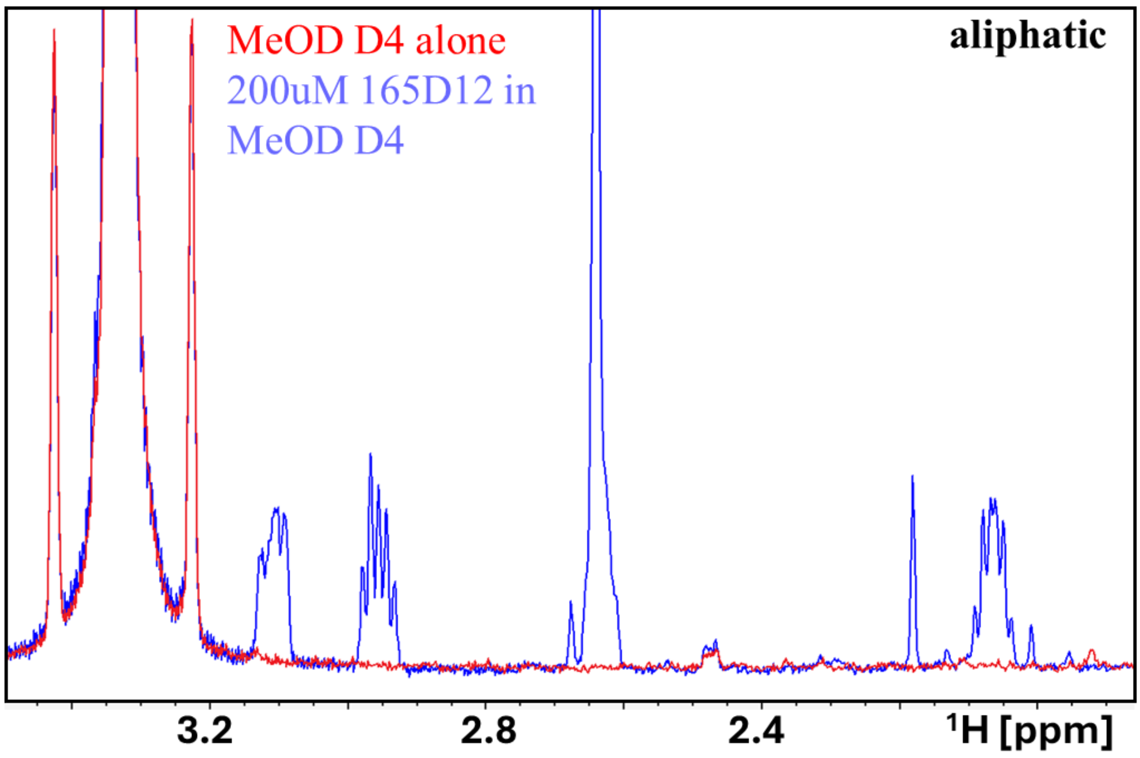
MeOD D4 alone
200uM 165D12 in
MeOD D4
aliphatic
3.2
2.8
2.4
^{1}H [ppm]

^{13}C NMR:

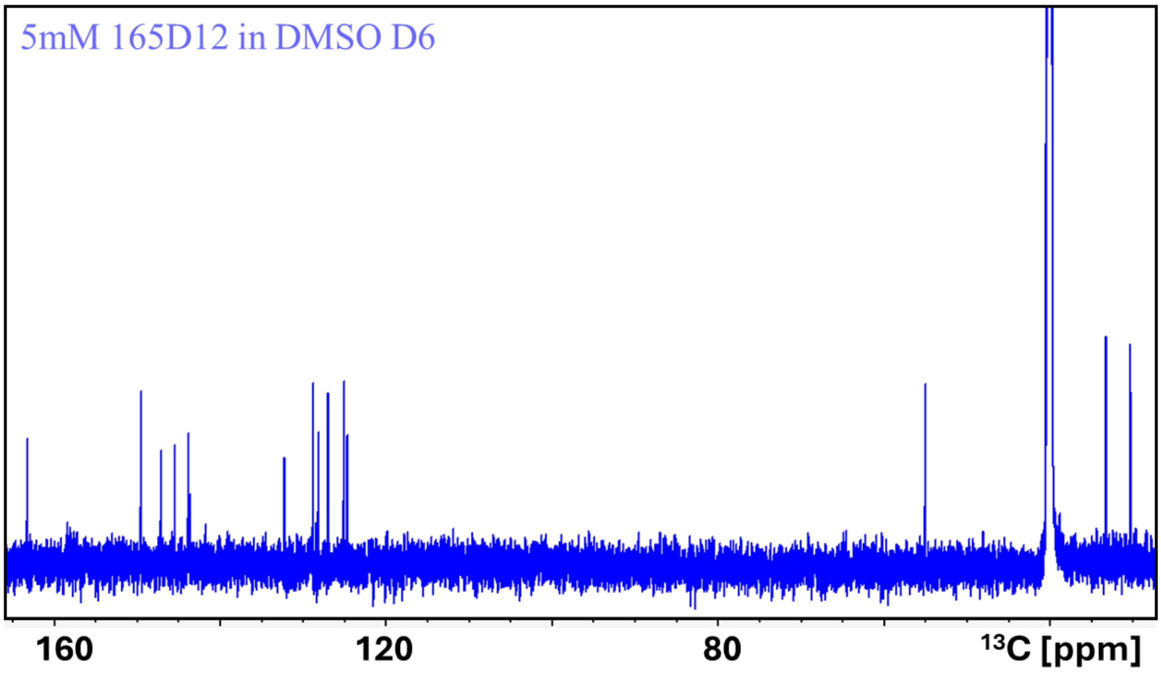
5mM 165D12 in DMSO D6
160
120
80
^{13}C [ppm]

^{19}F NMR:

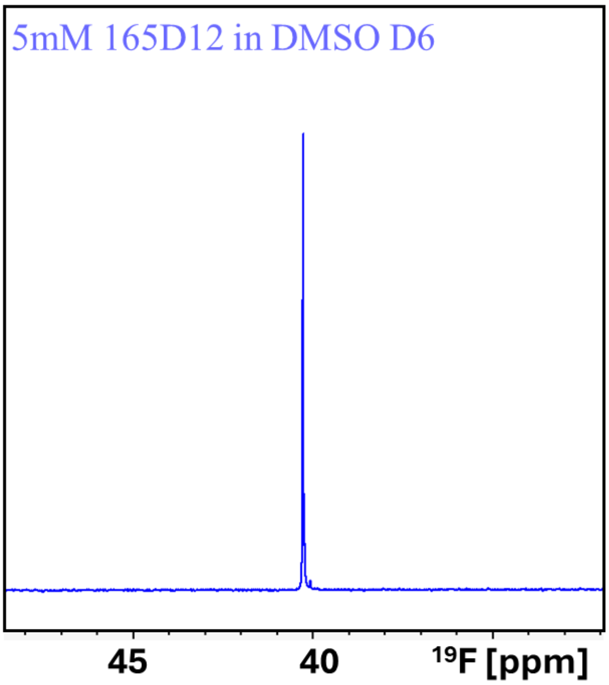

Compound 8 (165E12): H NMR (MeOD D4, 700 MHz): δ 2.45 (q, J = 7.1 Hz), 2.64 (s), 2.99 (m), 3.17 (m), 4.18 (t, J = 7.55 Hz), 7.19 (t, J = 7.09 Hz), 7.23 (t, J = 7.18 Hz), 7.31 (t, J = 7.6 Hz), 8.63 (s), 8.66 (s).

^{13}C NMR (700 MHz, in deuterated DMSO, DMSO D6): δ 29.24, δ 32.06, δ 52.06, δ 122.20, δ 124.78, δ 125.09, δ 126.82, δ 127.86, δ 135.28, δ 137.57, δ 140.82, δ 142.04, δ 142.12, δ 144.62, δ 173.84.

^{19}F NMR (600 MHz, in deuterated DMSO, DMSO D6): δ 42.65.

^{1}H NMR:

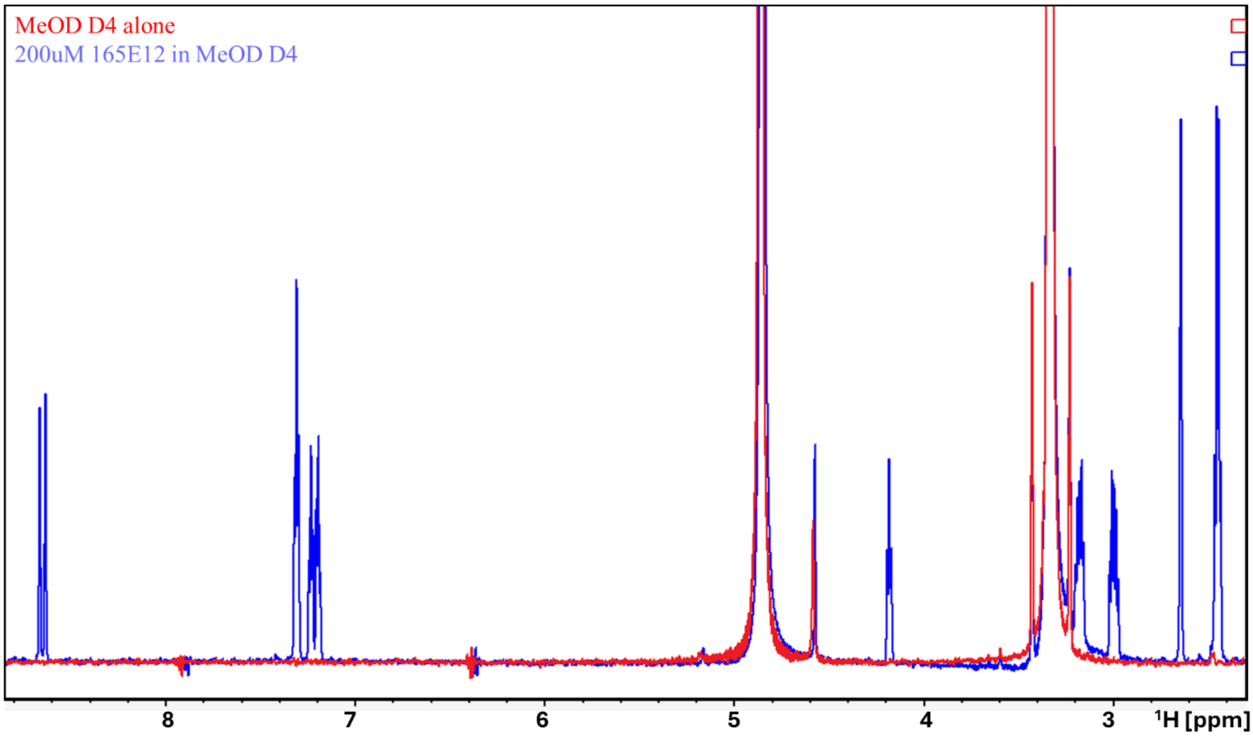

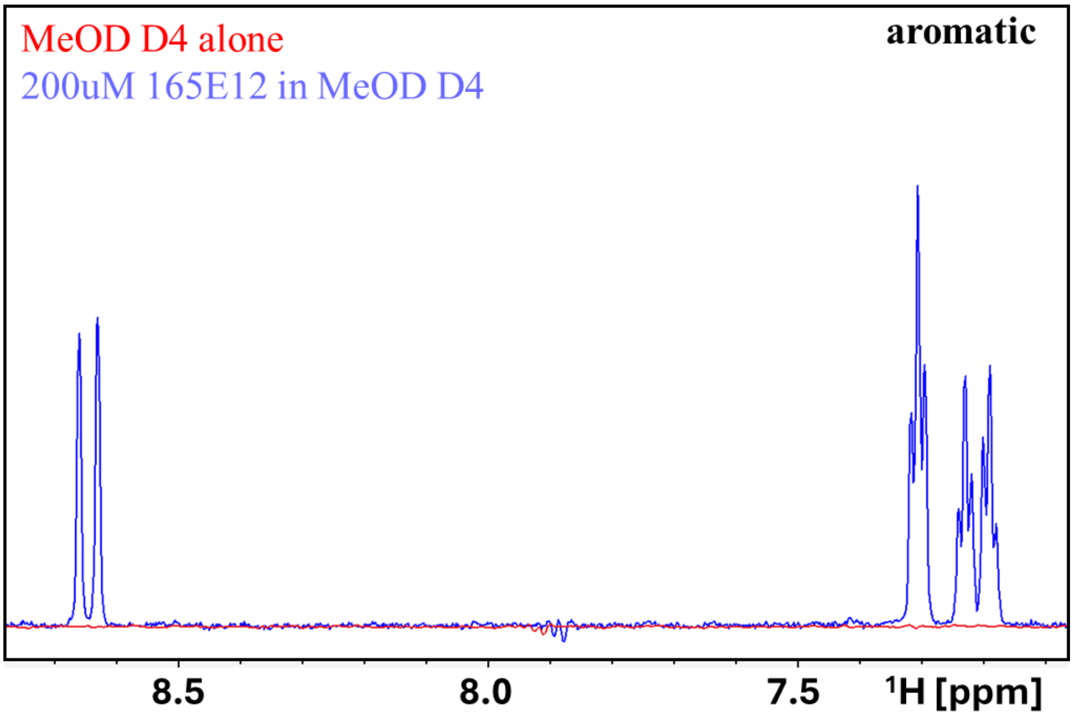

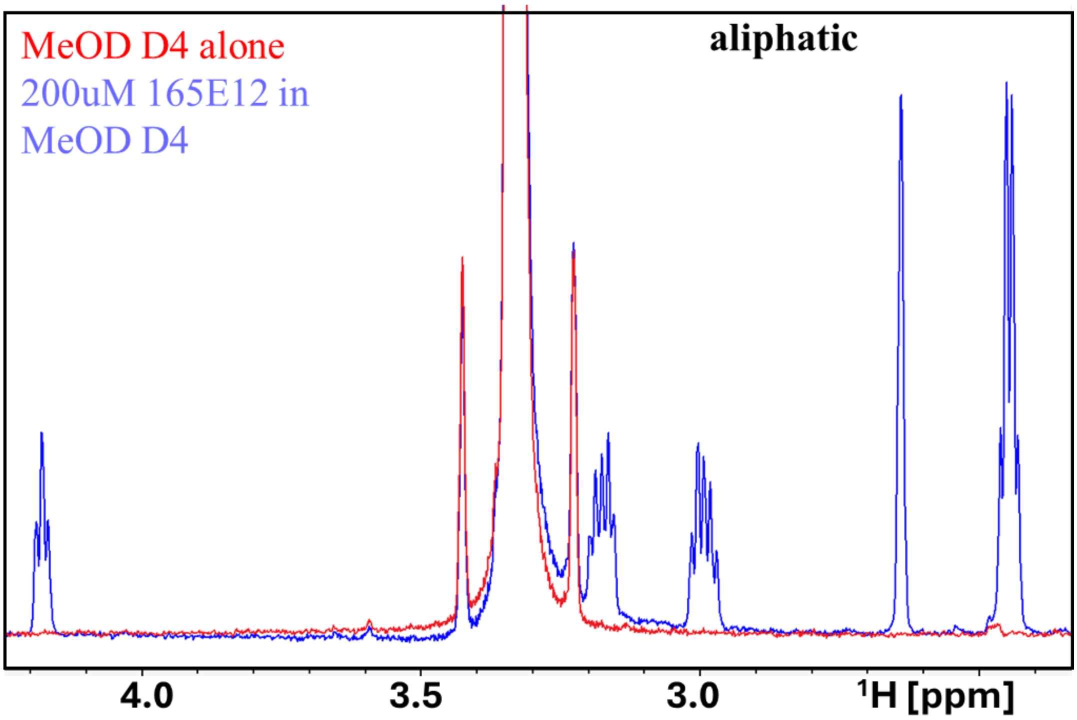
MeOD D4 alone
200uM 165E12 in
MeOD D4
aliphatic
4.0
3.5
3.0
1H [ppm]

^{13}C NMR:

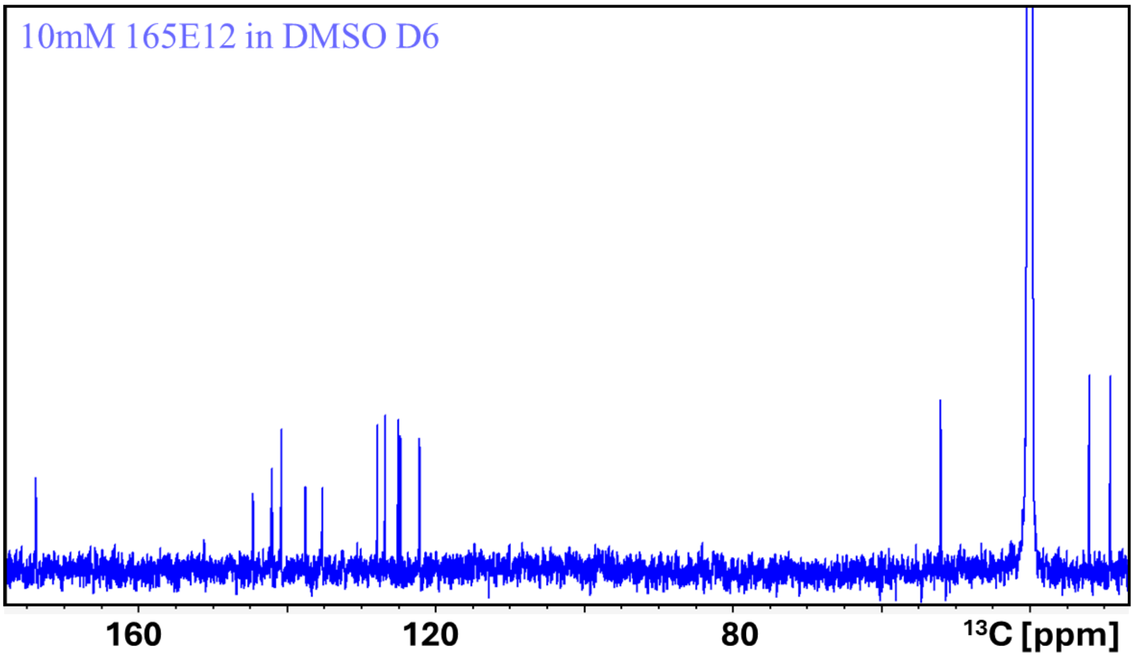
10mM 165E12 in DMSO D6
160
120
80
13C [ppm]

^{19}F NMR:

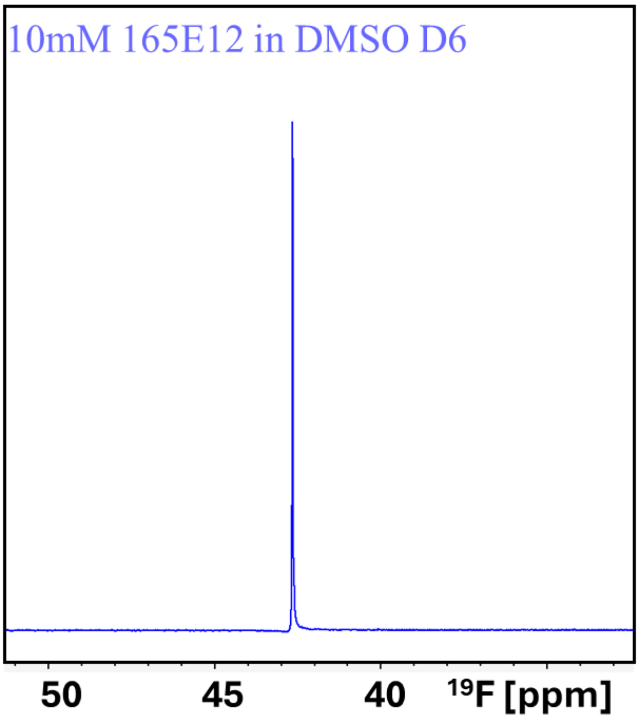

Compound 9 (165F6): H NMR (MeOD D4, 700 MHz): δ 2.07 (m), 2.64 (s), 2.96 (m), 3.11 (m), 5.68 (t, J = 8.00 Hz), 7.25 (m), 7.30 (d, J = 6.83 Hz), 7.35 (d, J = 7.29 Hz), 8.43 (s), 8.91 (s), 9.16 (s).

^{13}C NMR (700 MHz, in deuterated DMSO, DMSO D6): δ 30.36, δ 33.25, δ 55.03, δ 124.68, δ 125.08, δ 126.98, δ 128.13, δ 128.77, δ 132.24, δ 143.63, δ 143.81, δ 145.44, δ 147.10, δ 149.53, δ 163.22.

^{19}F NMR (600 MHz, in deuterated DMSO, DMSO D6): δ 40.28

^{1}H NMR:

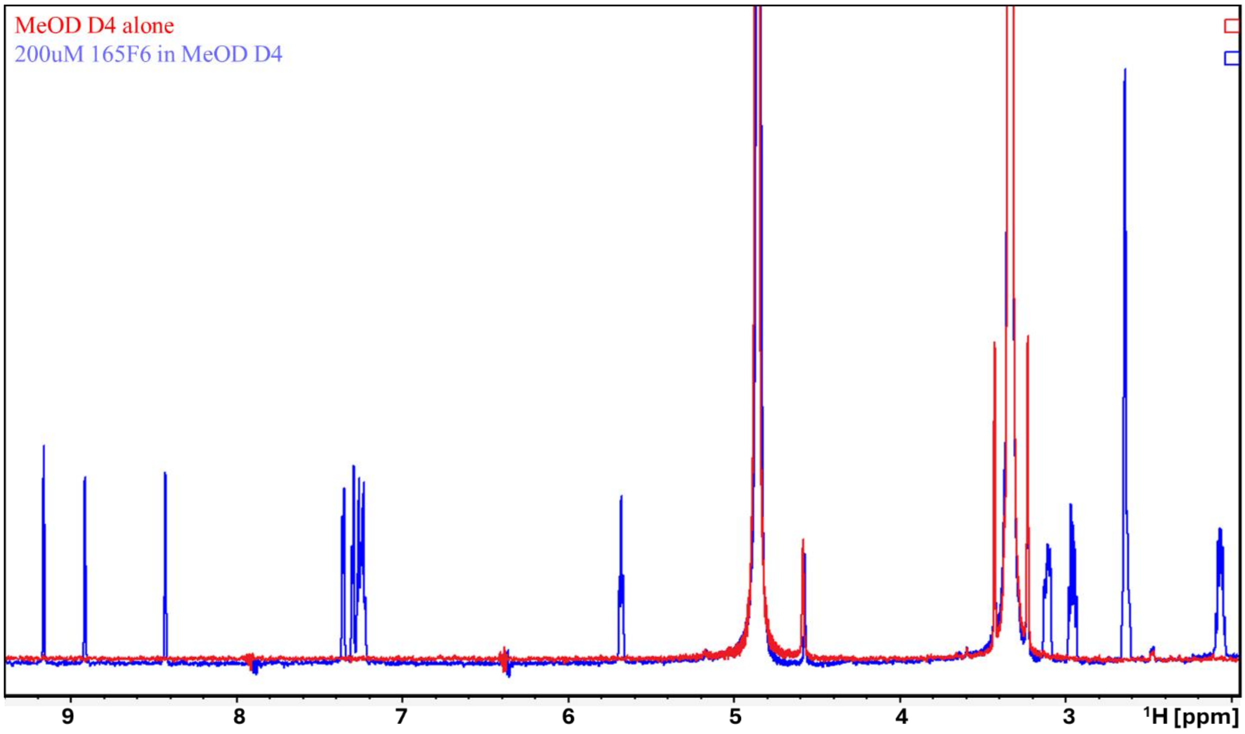

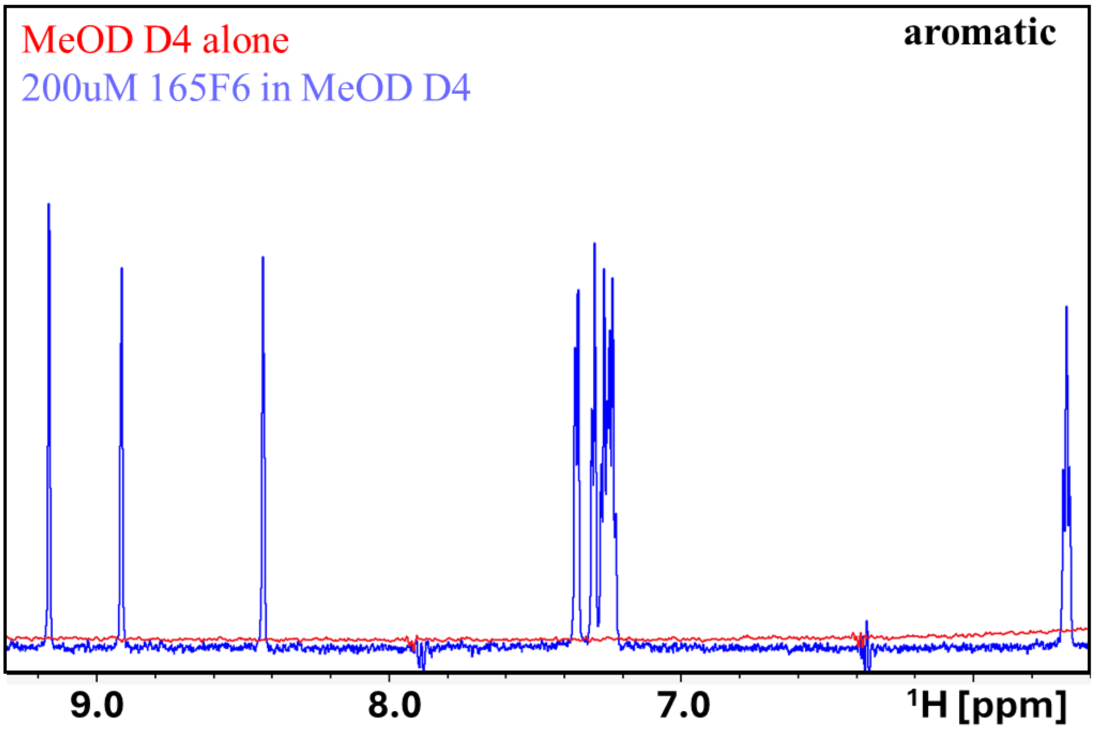
MeOD D4 alone
200uM 165F6 in MeOD D4
aromatic
9.0
8.0
7.0
1H [ppm]

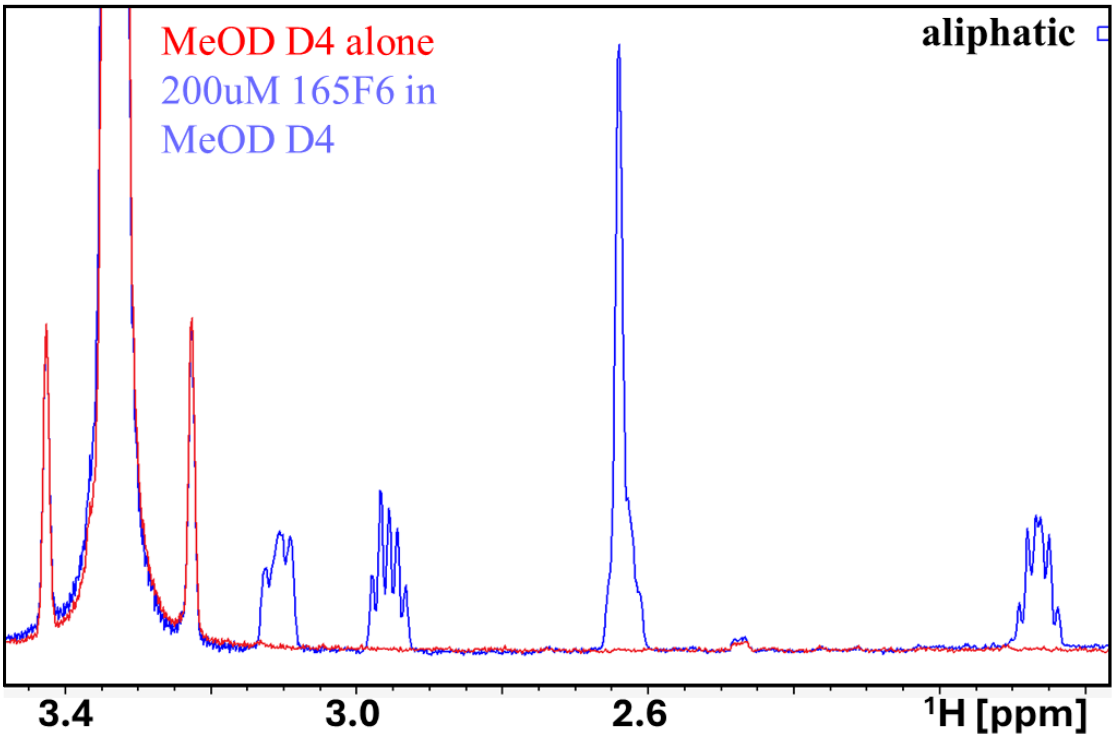
MeOD D4 alone
200uM 165F6 in MeOD D4
aliphatic
3.4
3.0
2.6
1H [ppm]

^{13}C NMR:

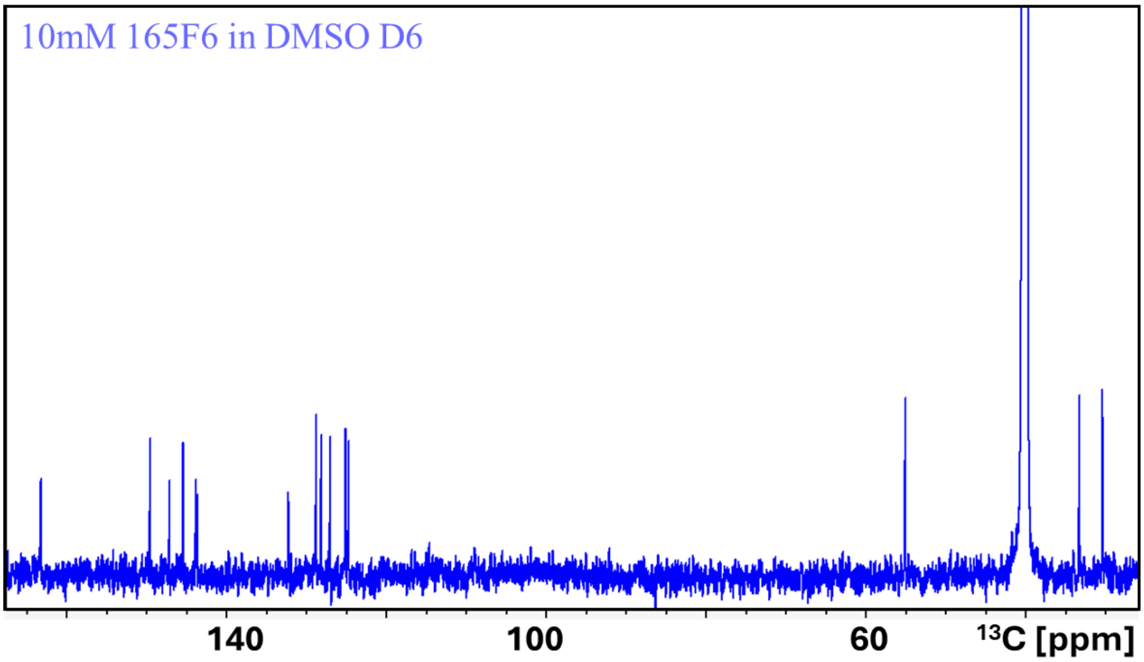

^{19}F NMR:

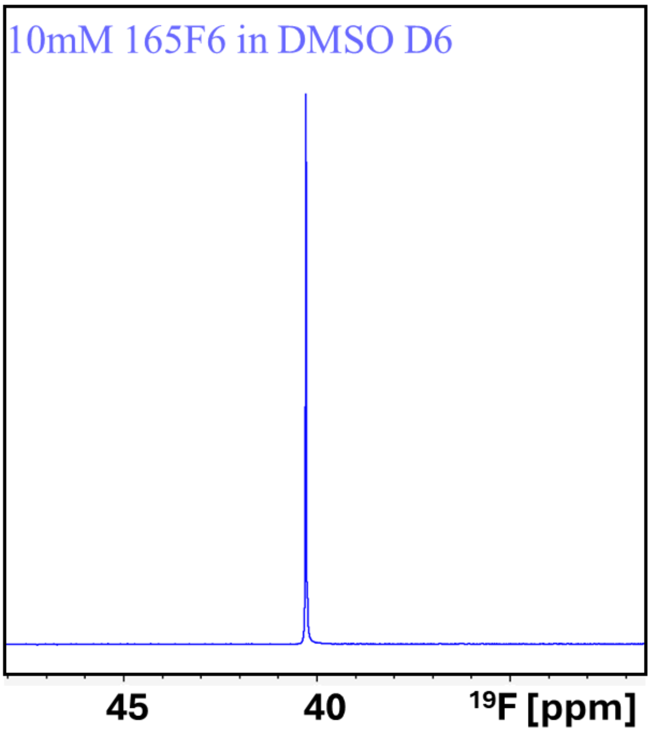

SUPPLEMENTARY TABLES

Supplementary Table S1: Chemical structures and properties of the 320 fragments in the library

Structure	MW	LogP	H bond acceptors	H bond donors	Rotatable bonds
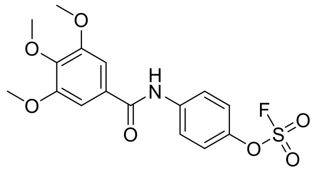 $C_{16}H_{16}FNO_7S$	385.362	2.112	6	1	8
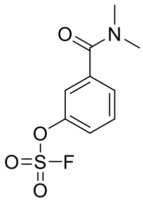 $C_9H_{10}FNO_4S$	247.24	1.063	3	0	4
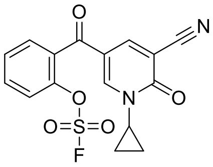 $C_{16}H_{11}FN_2O_5S$	362.331	0.926	5	0	6
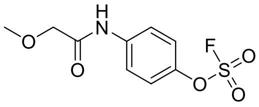 $C_9H_{10}FNO_5S$	263.239	0.254	4	1	6
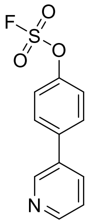 $C_{11}H_8FNO_3S$	253.247	2.023	3	0	3

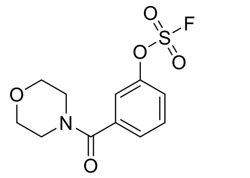 $C_{11}H_{12}FNO_5S$	289.277	0.664	4	0	4
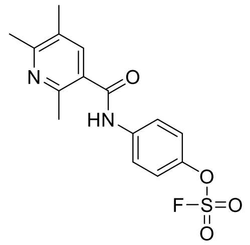 $C_{15}H_{15}FN_2O_4S$	338.353	3.049	4	1	5
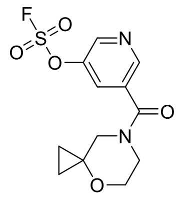 $C_{12}H_{13}FN_2O_5S$	316.303	-0.498	5	0	4
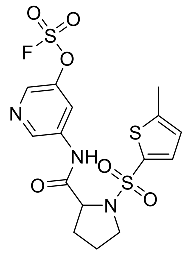 $C_{15}H_{16}FN_3O_6S_3$	449.486	1.063	6	1	7
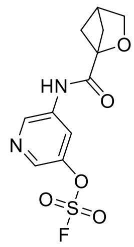 $C_{11}H_{11}FN_2O_5S$	302.276	-0.669	5	1	5

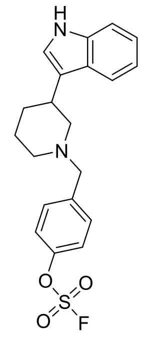 $C_{20}H_{21}FN_2O_3S$	388.457	3.488	4	1	5
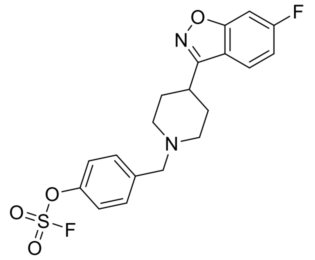 $C_{19}H_{18}F_2N_2O_4S$	408.42	3.617	6	0	5
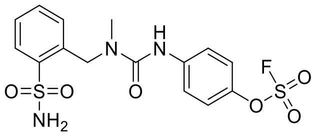 $C_{15}H_{16}FN_3O_6S_2$	417.426	1.214	5	2	8
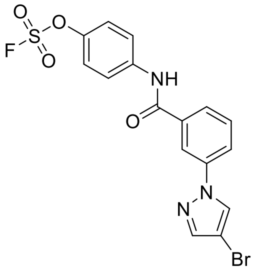 $C_{16}H_{11}BrFN_3O_4S$	440.243	3.319	5	1	6
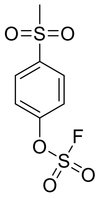 $C_7H_7FO_5S_2$	254.246	0.391	4	0	3
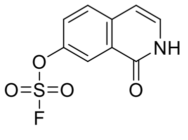 $C_9H_6FNO_4S$	243.208	0.934	3	1	2

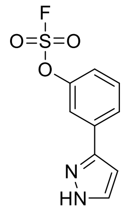 $C_9H_7FN_2O_3S$	242.224	1.885	4	1	3
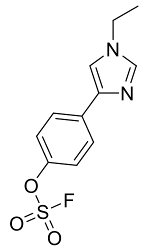 $C_{11}H_{11}FN_2O_3S$	270.278	1.658	4	0	4
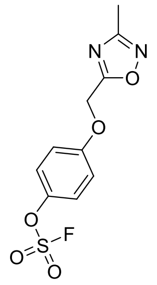 $C_{10}H_9FN_2O_5S$	288.249	2.578	6	0	5
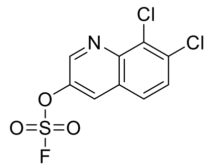 $C_9H_4Cl_2FNO_3S$	296.093	2.885	3	0	2
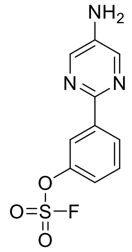 $C_{10}H_8FN_3O_3S$	269.25	1.515	5	1	3

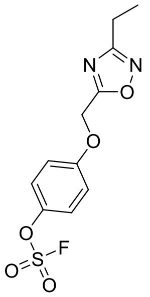 $C_{11}H_{11}FN_2O_5S$	302.276	3.151	6	0	6
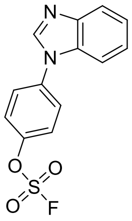 $C_{13}H_9FN_2O_3S$	292.284	2.361	4	0	3
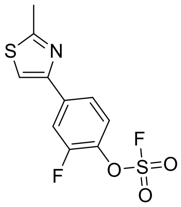 $C_{10}H_7F_2NO_3S_2$	291.287	3.351	4	0	3
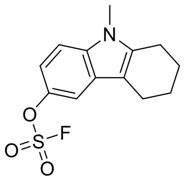 $C_{13}H_{14}FNO_3S$	283.317	2.691	3	0	2
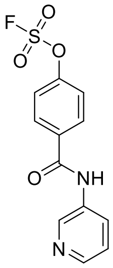 $C_{12}H_9FN_2O_4S$	296.272	1.154	4	1	5

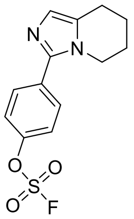 $C_{13}H_{13}FN_2O_3S$	296.316	2.006	4	0	3
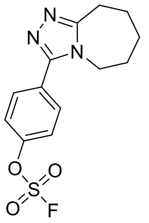 $C_{13}H_{14}FN_3O_3S$	311.331	2.6	5	0	3
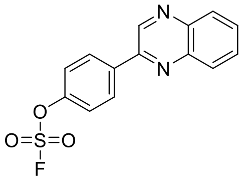 $C_{14}H_9FN_2O_3S$	304.295	2.954	4	0	3
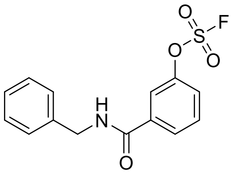 $C_{14}H_{12}FNO_4S$	309.311	2.56	3	1	6
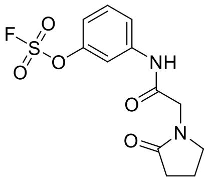 $C_{12}H_{13}FN_2O_5S$	316.303	-0.175	4	1	6
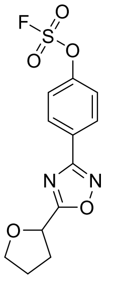 $C_{12}H_{11}FN_2O_5S$	314.287	2.71	6	0	4

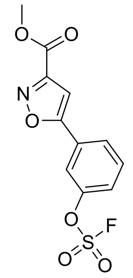 $C_{11}H_8FNO_6S$	301.244	1.833	5	0	5
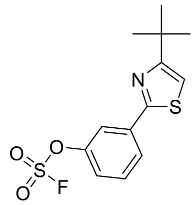 $C_{13}H_{14}FNO_3S_2$	315.377	4.605	3	0	4
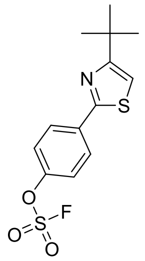 $C_{13}H_{14}FNO_3S_2$	315.377	4.605	3	0	4
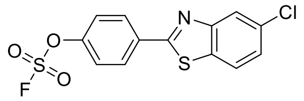 $C_{13}H_7ClFNO_3S_2$	343.771	4.512	3	0	3
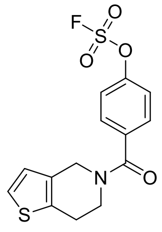 $C_{14}H_{12}FNO_4S_2$	341.371	2.582	3	0	4
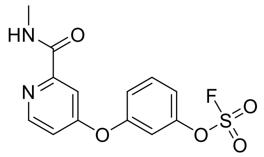 $C_{13}H_{11}FN_2O_5S$	326.298	1.451	5	1	6

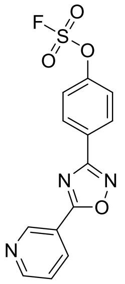 $C_{13}H_8FN_3O_4S$	321.282	2.693	6	0	4
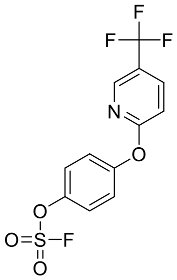 $C_{12}H_7F_4NO_4S$	337.245	3.522	7	0	5
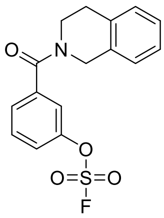 $C_{16}H_{14}FNO_4S$	335.349	2.774	3	0	4
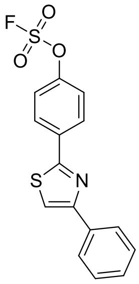 $C_{15}H_{10}FNO_3S_2$	335.367	4.576	3	0	4
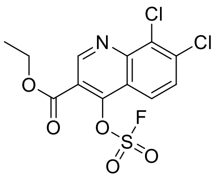 $C_{12}H_8Cl_2FNO_5S$	368.156	3.044	4	0	5

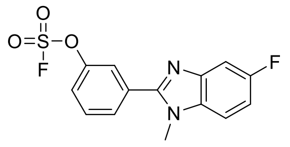<br>**$C_{14}H_{10}F_2N_2O_3S$**	**324.302**	**2.909**	**5**	**0**	**3**
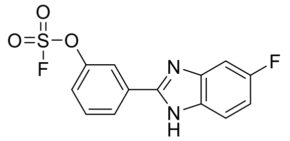<br>**$C_{13}H_8F_2N_2O_3S$**	**310.275**	**2.674**	**5**	**1**	**3**
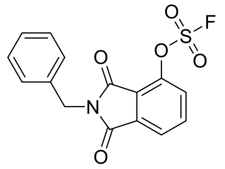<br>**$C_{15}H_{10}FNO_5S$**	**335.305**	**2.372**	**4**	**0**	**4**
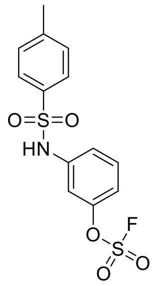<br>**$C_{13}H_{12}FNO_5S_2$**	**345.359**	**2.579**	**4**	**1**	**5**
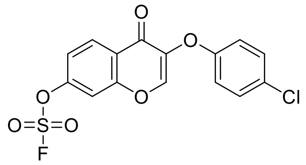<br>**$C_{15}H_8ClFO_6S$**	**370.731**	**2.601**	**5**	**0**	**4**
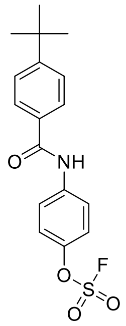<br>**$C_{17}H_{18}FNO_4S$**	**351.392**	**4.195**	**3**	**1**	**6**

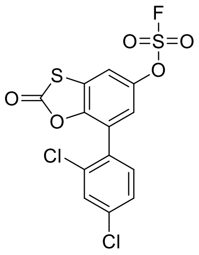 $C_{13}H_5Cl_2FO_5S_2$	395.196	4.844	3	0	3
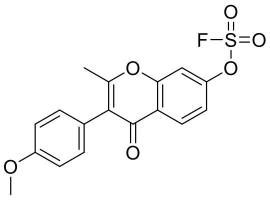 $C_{17}H_{13}FO_6S$	364.343	2.063	5	0	4
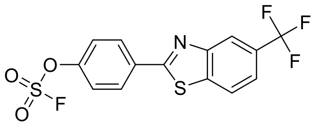 $C_{14}H_7F_4NO_3S_2$	377.328	4.875	6	0	4
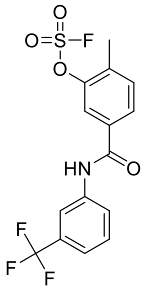 $C_{15}H_{11}F_4NO_4S$	377.31	3.899	6	1	6
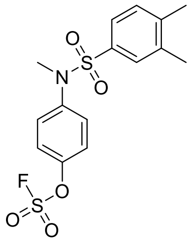 $C_{15}H_{16}FNO_5S_2$	373.413	4.332	4	0	5
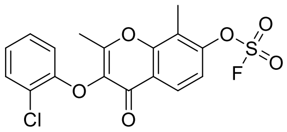 $C_{17}H_{12}ClFO_6S$	398.785	2.714	5	0	4

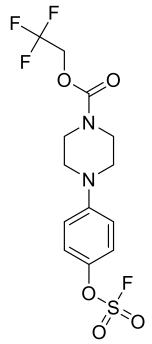 $C_{13}H_{14}F_4N_2O_5S$	386.318	2.561	7	0	7
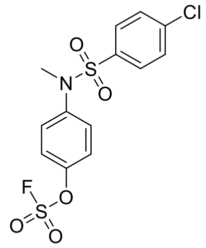 $C_{13}H_{11}ClFNO_5S_2$	379.801	4.121	4	0	5
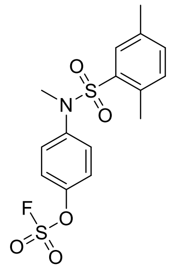 $C_{15}H_{16}FNO_5S_2$	373.413	4.332	4	0	5
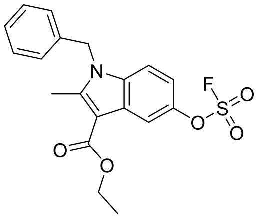 $C_{19}H_{18}FNO_5S$	391.413	3.689	4	0	7
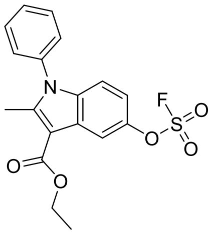 $C_{18}H_{16}FNO_5S$	377.386	3.62	4	0	6

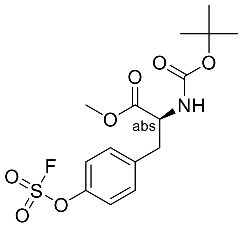 $C_{15}H_{20}FNO_7S$	377.383	2.086	4	1	10
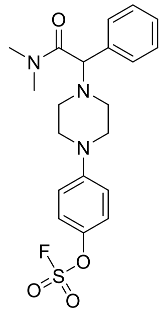 $C_{20}H_{24}FN_3O_4S$	421.487	2.7	5	0	7
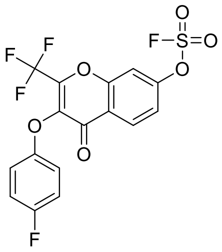 $C_{16}H_7F_5O_6S$	422.278	2.467	9	0	5
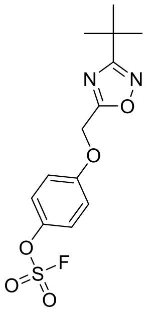 $C_{13}H_{15}FN_2O_5S$	330.33	4.263	6	0	6
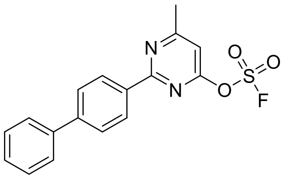 $C_{17}H_{13}FN_2O_3S$	344.36	5.413	4	0	4

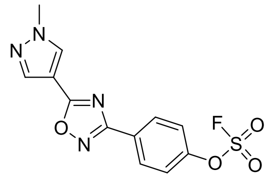 $C_{12}H_9FN_4O_4S$	324.286	2.366	7	0	4
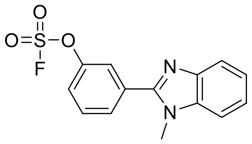 $C_{14}H_{11}FN_2O_3S$	306.311	2.751	4	0	3
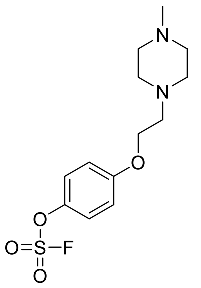 $C_{13}H_{19}FN_2O_4S$	318.363	1.314	5	0	6
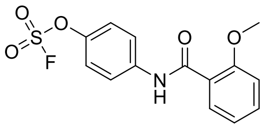 $C_{14}H_{12}FNO_5S$	325.31	2.364	4	1	6
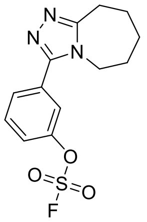 $C_{13}H_{14}FN_3O_3S$	311.331	2.6	5	0	3
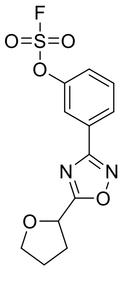 $C_{12}H_{11}FN_2O_5S$	314.287	2.71	6	0	4

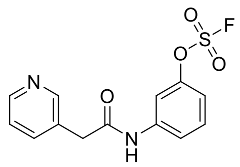<br>$C_{13}H_{11}FN_2O_4S$	310.299	1.098	4	1	6
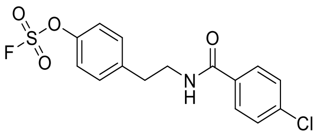<br>$C_{15}H_{13}ClFNO_4S$	357.78	3.398	3	1	7
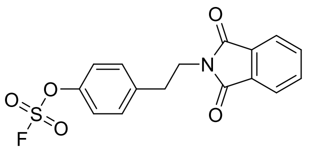<br>$C_{16}H_{12}FNO_5S$	349.332	2.651	4	0	5
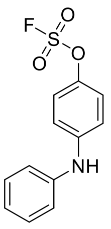<br>$C_{12}H_{10}FNO_3S$	267.274	3.15	3	1	4
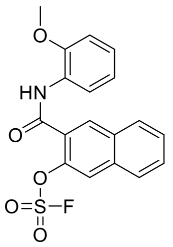<br>$C_{18}H_{14}FNO_5S$	375.37	3.362	4	1	6
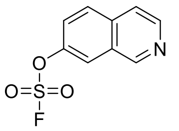<br>$C_9H_6FNO_3S$	227.209	1.345	3	0	2
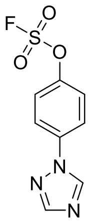	243.212	1.237	5	0	3

**$C_8H_6FN_3O_3S$**					
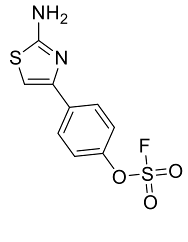 **$C_9H_7FN_2O_3S_2$**	**274.284**	**2.701**	**4**	**1**	**3**
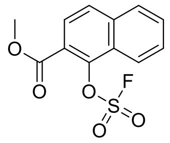 **$C_{12}H_9FO_5S$**	**284.257**	**2.503**	**3**	**0**	**4**
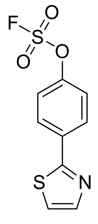 **$C_9H_6FNO_3S_2$**	**259.269**	**2.477**	**3**	**0**	**3**
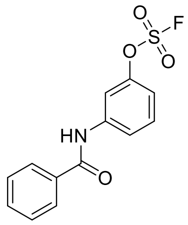 **$C_{13}H_{10}FNO_4S$**	**295.284**	**2.491**	**3**	**1**	**5**
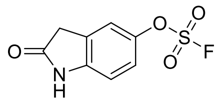 **$C_8H_6FNO_4S$**	**231.197**	**0.401**	**3**	**1**	**2**

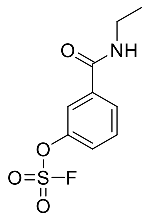<br>**$C_9H_{10}FNO_4S$**	**247.24**	**1.165**	**3**	**1**	**5**
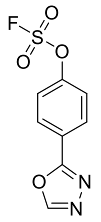<br>**$C_8H_5FN_2O_4S$**	**244.196**	**0.867**	**5**	**0**	**3**
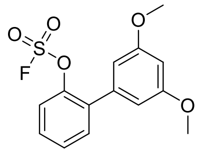<br>**$C_{14}H_{13}FO_5S$**	**312.311**	**3.108**	**4**	**0**	**5**
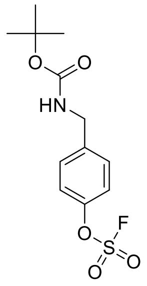<br>**$C_{12}H_{16}FNO_5S$**	**305.32**	**2.122**	**3**	**1**	**7**
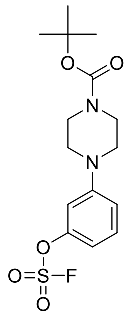<br>**$C_{15}H_{21}FN_2O_5S$**	**360.4**	**2.457**	**4**	**0**	**6**
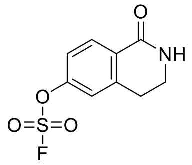	**245.224**	**0.806**	**3**	**1**	**2**

**$C_9H_8FNO_4S$**					
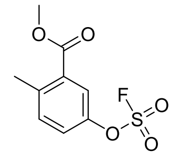<br>**$C_9H_9FO_5S$**	**248.224**	**1.992**	**3**	**0**	**4**
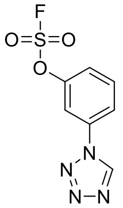<br>**$C_7H_5FN_4O_3S$**	**244.2**	**1.047**	**6**	**0**	**3**
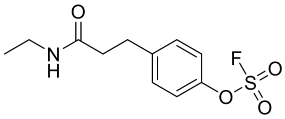<br>**$C_{11}H_{14}FNO_4S$**	**275.294**	**1.527**	**3**	**1**	**7**
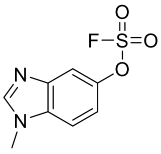<br>**$C_8H_7FN_2O_3S$**	**230.213**	**0.698**	**4**	**0**	**2**
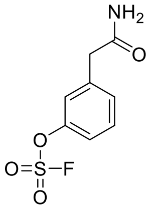<br>**$C_8H_8FNO_4S$**	**233.213**	**0.536**	**3**	**1**	**4**
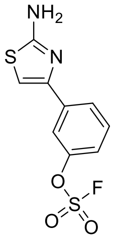<br>**$C_9H_7FN_2O_3S_2$**	**274.284**	**2.701**	**4**	**1**	**3**

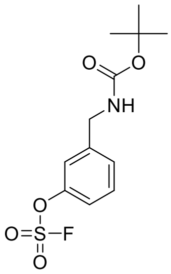 $C_{12}H_{16}FNO_5S$	305.32	2.122	3	1	7
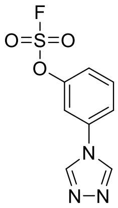 $C_8H_6FN_3O_3S$	243.212	0.641	5	0	3
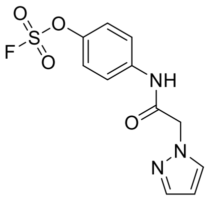 $C_{11}H_{10}FN_3O_4S$	299.276	0.536	5	1	6
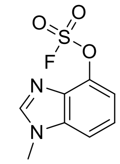 $C_8H_7FN_2O_3S$	230.213	0.698	4	0	2
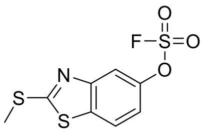 $C_8H_6FNO_3S_3$	279.318	3.321	3	0	3
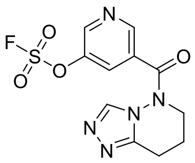 $C_{11}H_{10}FN_5O_4S$	327.29	-0.263	7	0	4

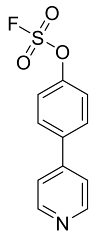 $C_{11}H_8FNO_3S$	253.247	2.023	3	0	3
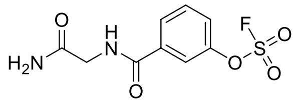 $C_9H_9FN_2O_5S$	276.238	-0.557	4	2	6
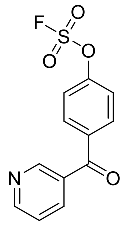 $C_{12}H_8FNO_4S$	281.257	1.558	4	0	4
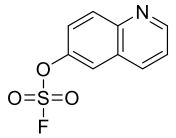 $C_9H_6FNO_3S$	227.209	1.769	3	0	2
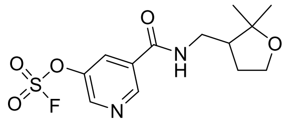 $C_{13}H_{17}FN_2O_5S$	332.346	-0.046	5	1	6
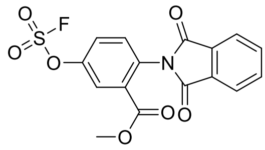 $C_{16}H_{10}FNO_7S$	379.314	2.123	5	0	5
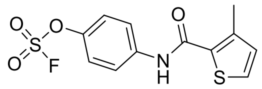 $C_{12}H_{10}FNO_4S_2$	315.333	2.96	3	1	5

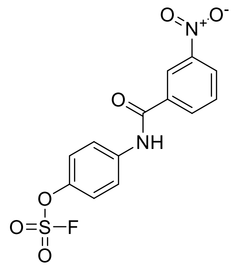 $C_{13}H_9FN_2O_6S$	340.281	3.149	4	1	6
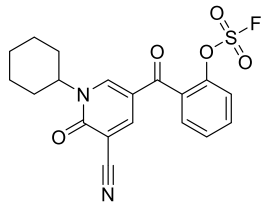 $C_{19}H_{17}FN_2O_5S$	404.412	2.178	5	0	6
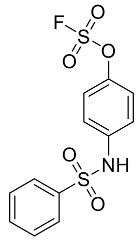 $C_{12}H_{10}FNO_5S_2$	331.332	2.092	4	1	5
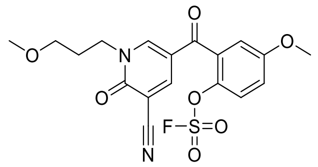 $C_{18}H_{17}FN_2O_7S$	424.399	0.454	7	0	10
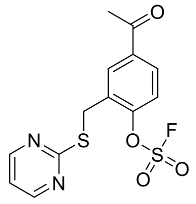 $C_{13}H_{11}FN_2O_4S_2$	342.359	1.746	5	0	6
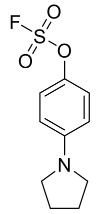 $C_{10}H_{12}FNO_3S$	245.268	2.286	3	0	3

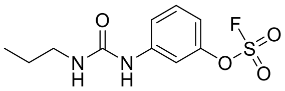 **$C_{10}H_{13}FN_2O_4S$**	**276.282**	**1.325**	**3**	**2**	**7**
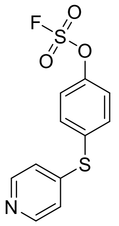 **$C_{11}H_8FNO_3S_2$**	**285.307**	**2.451**	**3**	**0**	**4**
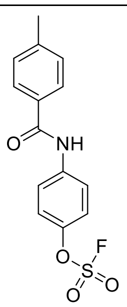 **$C_{14}H_{12}FNO_4S$**	**309.311**	**2.978**	**3**	**1**	**5**
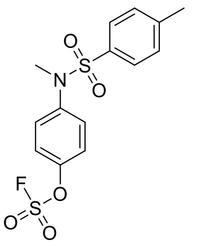 **$C_{14}H_{14}FNO_5S_2$**	**359.386**	**3.917**	**4**	**0**	**5**
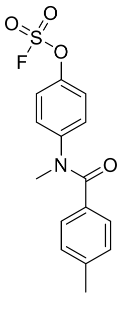 **$C_{15}H_{14}FNO_4S$**	**323.338**	**3.214**	**3**	**0**	**5**
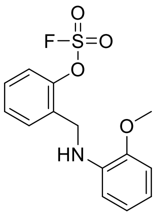	**311.327**	**2.788**	**4**	**1**	**6**

$C_{14}H_{14}FNO_4S$					
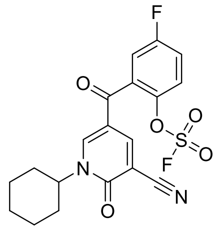 $C_{19}H_{16}F_2N_2O_5S$	422.403	2.336	6	0	6
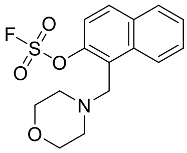 $C_{15}H_{16}FNO_4S$	325.354	2.225	4	0	4
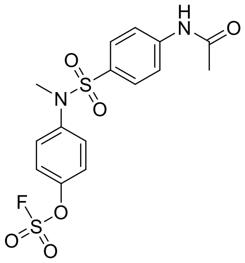 $C_{15}H_{15}FN_2O_6S_2$	402.411	2.918	5	1	7
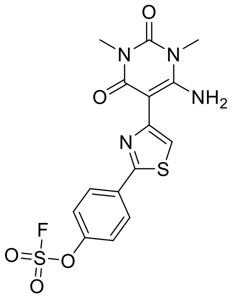 $C_{15}H_{13}FN_4O_5S_2$	412.41	1.37	6	1	4
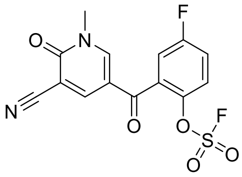 $C_{14}H_8F_2N_2O_5S$	354.284	0.788	6	0	5

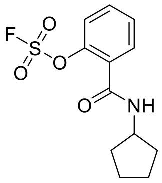 **$C_{12}H_{14}FNO_4S$**	**287.305**	**1.958**	**3**	**1**	**5**
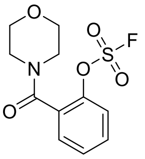 **$C_{11}H_{12}FNO_5S$**	**289.277**	**0.664**	**4**	**0**	**4**
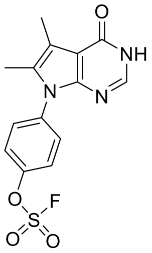 **$C_{14}H_{12}FN_3O_4S$**	**337.325**	**1.911**	**5**	**1**	**3**
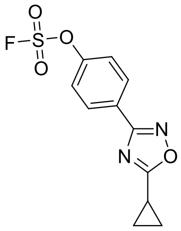 **$C_{11}H_9FN_2O_4S$**	**284.261**	**3.208**	**5**	**0**	**4**
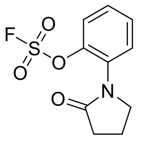 **$C_{10}H_{10}FNO_4S$**	**259.251**	**0.974**	**3**	**0**	**3**
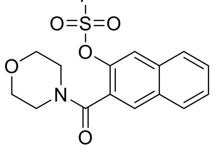 **$C_{15}H_{14}FNO_5S$**	**339.337**	**1.661**	**4**	**0**	**4**

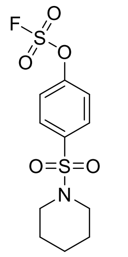<br>$C_{11}H_{14}FNO_5S_2$	323.353	1.697	4	0	4
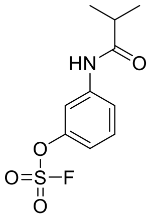<br>$C_{10}H_{12}FNO_4S$	261.267	1.814	3	1	5
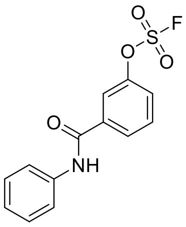<br>$C_{13}H_{10}FNO_4S$	295.284	2.491	3	1	5
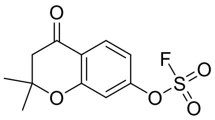<br>$C_{11}H_{11}FO_5S$	274.262	1.273	4	0	2
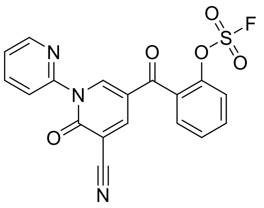<br>$C_{18}H_{10}FN_3O_5S$	399.352	1.673	6	0	6
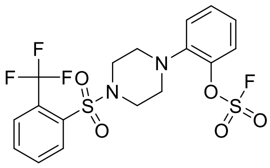<br>$C_{17}H_{16}F_4N_2O_5S_2$	468.438	3.717	8	0	6

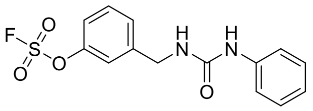 **$C_{14}H_{13}FN_2O_4S$**	**324.326**	**2.233**	**3**	**2**	**7**
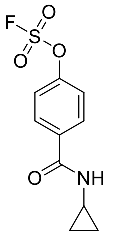 **$C_{10}H_{10}FNO_4S$**	**259.251**	**1.123**	**3**	**1**	**5**
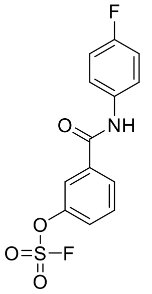 **$C_{13}H_9F_2NO_4S$**	**313.275**	**2.649**	**4**	**1**	**5**
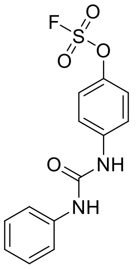 **$C_{13}H_{11}FN_2O_4S$**	**310.299**	**2.164**	**3**	**2**	**6**
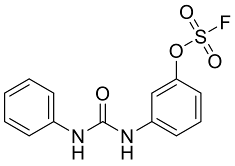 **$C_{13}H_{11}FN_2O_4S$**	**310.299**	**2.164**	**3**	**2**	**6**
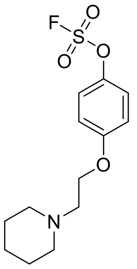	**303.348**	**2.291**	**4**	**0**	**6**

$C_{13}H_{18}FNO_4S$					
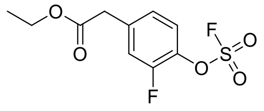 $C_{10}H_{10}F_2O_5S$	280.242	1.946	4	0	6
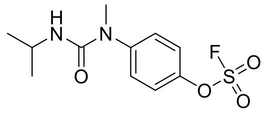 $C_{11}H_{15}FN_2O_4S$	290.309	1.393	3	1	6
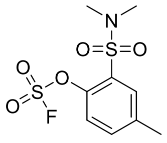 $C_9H_{12}FNO_5S_2$	297.315	1.452	4	0	4
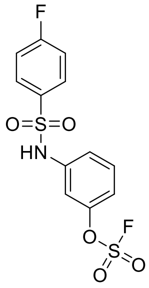 $C_{12}H_9F_2NO_5S_2$	349.323	2.25	5	1	5
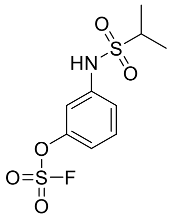 $C_9H_{12}FNO_5S_2$	297.315	0.911	4	1	5

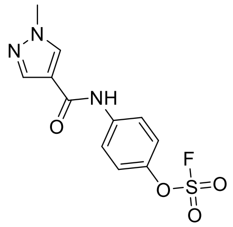 $C_{11}H_{10}FN_3O_4S$	299.276	0.827	5	1	5
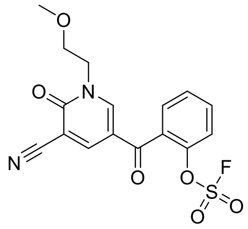 $C_{16}H_{13}FN_2O_6S$	380.346	0.475	6	0	8
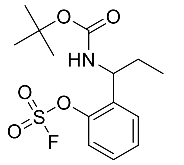 $C_{14}H_{20}FNO_5S$	333.374	2.927	3	1	8
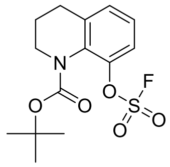 $C_{14}H_{18}FNO_5S$	331.358	2.685	3	0	5
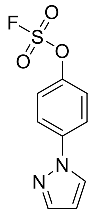 $C_9H_7FN_2O_3S$	242.224	1.685	4	0	3
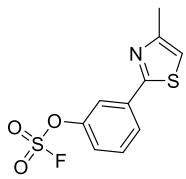 $C_{10}H_8FNO_3S_2$	273.296	3.181	3	0	3

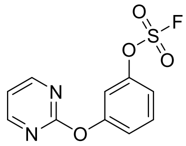 $C_{10}H_7FN_2O_4S$	270.234	1.798	5	0	4
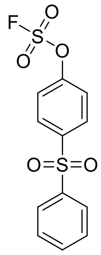 $C_{12}H_9FO_5S_2$	316.317	2.576	4	0	4
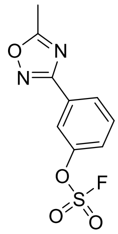 $C_9H_7FN_2O_4S$	258.223	2.647	5	0	3
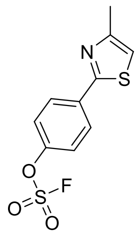 $C_{10}H_8FNO_3S_2$	273.296	3.181	3	0	3
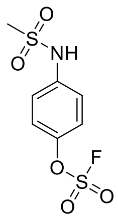 $C_7H_8FNO_5S_2$	269.261	-0.094	4	1	4
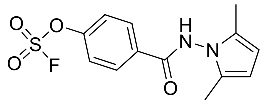 $C_{13}H_{13}FN_2O_4S$	312.315	2.208	4	1	5

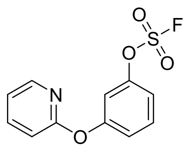 $C_{11}H_8FNO_4S$	269.246	2.601	4	0	4
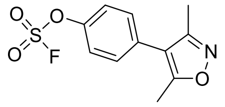 $C_{11}H_{10}FNO_4S$	271.262	2.574	4	0	3
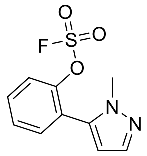 $C_{10}H_9FN_2O_3S$	256.251	1.753	4	0	3
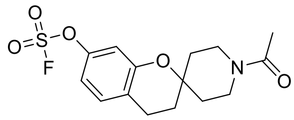 $C_{15}H_{18}FNO_5S$	343.369	1.052	4	0	3
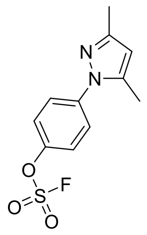 $C_{11}H_{11}FN_2O_3S$	270.278	2.726	4	0	3
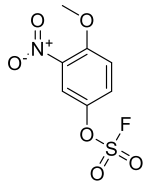 $C_7H_6FNO_6S$	251.184	2.575	4	0	4
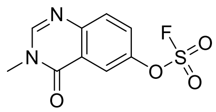 $C_9H_7FN_2O_4S$	258.223	0.748	4	0	2

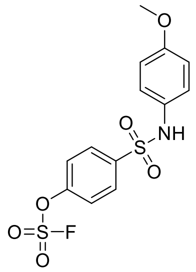 $C_{13}H_{12}FNO_6S_2$	361.358	1.966	5	1	6
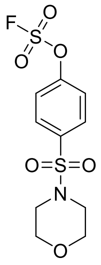 $C_{10}H_{12}FNO_6S_2$	325.325	0.565	5	0	4
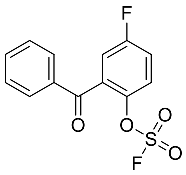 $C_{13}H_8F_2O_4S$	298.26	3.053	4	0	4
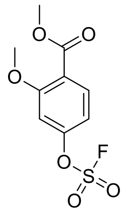 $C_9H_9FO_6S$	264.223	1.379	4	0	5
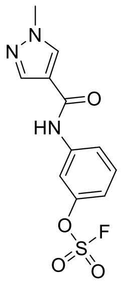 $C_{11}H_{10}FN_3O_4S$	299.276	0.827	5	1	5
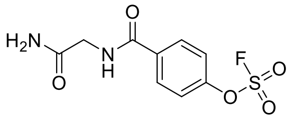 $C_9H_9FN_2O_5S$	276.238	-0.557	4	2	6

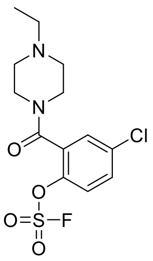 $C_{13}H_{16}ClFN_2O_4S$	350.789	1.715	4	0	5
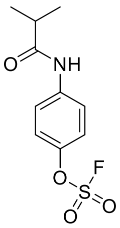 $C_{10}H_{12}FNO_4S$	261.267	1.814	3	1	5
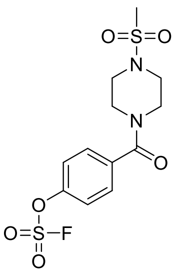 $C_{12}H_{15}FN_2O_6S_2$	366.378	-0.297	5	0	5
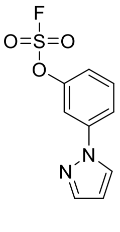 $C_9H_7FN_2O_3S$	242.224	1.685	4	0	3
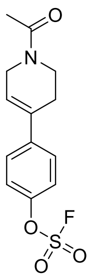 $C_{13}H_{14}FNO_4S$	299.316	1.081	3	0	4

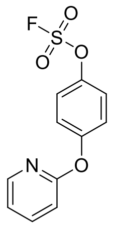 $C_{11}H_8FNO_4S$	269.246	2.601	4	0	4
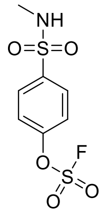 $C_7H_8FNO_5S_2$	269.261	2.154	4	1	4
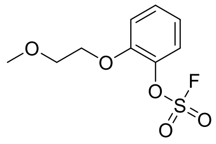 $C_9H_{11}FO_5S$	250.24	1.403	4	0	6
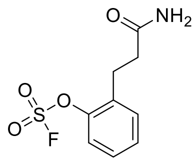 $C_9H_{10}FNO_4S$	247.24	0.953	3	1	5
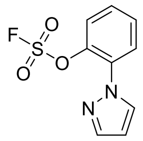 $C_9H_7FN_2O_3S$	242.224	1.685	4	0	3
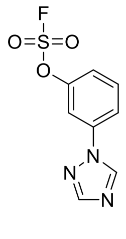 $C_8H_6FN_3O_3S$	243.212	1.237	5	0	3

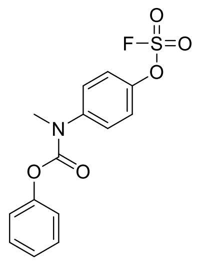 $C_{14}H_{12}FNO_5S$	325.31	3.078	3	0	6
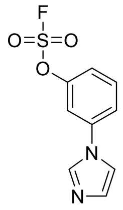 $C_9H_7FN_2O_3S$	242.224	0.885	4	0	3
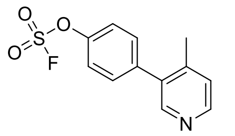 $C_{12}H_{10}FNO_3S$	267.274	2.511	3	0	3
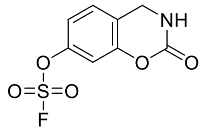 $C_8H_6FNO_5S$	247.196	0.877	3	1	2
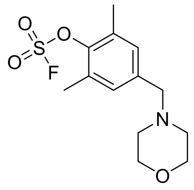 $C_{13}H_{18}FNO_4S$	303.348	2.202	4	0	4
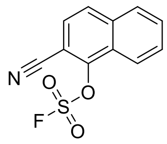 $C_{11}H_6FNO_3S$	251.231	2.716	3	0	3
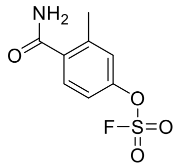 $C_8H_8FNO_4S$	233.213	1.079	3	1	3

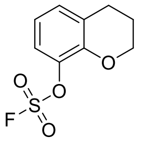 $C_9H_9FO_4S$	232.225	1.954	3	0	2
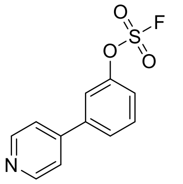 $C_{11}H_8FNO_3S$	253.247	2.023	3	0	3
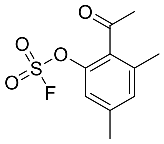 $C_{10}H_{11}FO_4S$	246.252	1.972	3	0	3
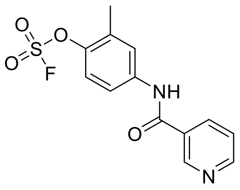 $C_{13}H_{11}FN_2O_4S$	310.299	1.641	4	1	5
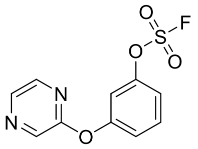 $C_{10}H_7FN_2O_4S$	270.234	1.264	5	0	4
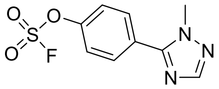 $C_9H_8FN_3O_3S$	257.239	1.627	5	0	3
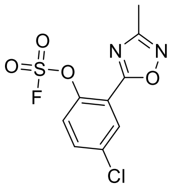 $C_9H_6ClFN_2O_4S$	292.665	2.933	5	0	3

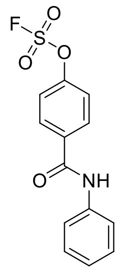 $C_{13}H_{10}FNO_4S$	295.284	2.491	3	1	5
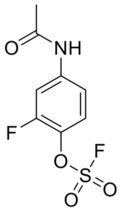 $C_8H_7F_2NO_4S$	251.204	0.751	4	1	4
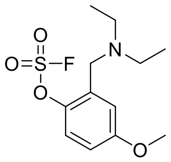 $C_{12}H_{18}FNO_4S$	291.337	2.177	4	0	7
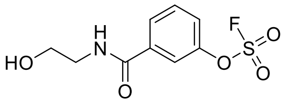 $C_9H_{10}FNO_5S$	263.239	0.31	4	2	6
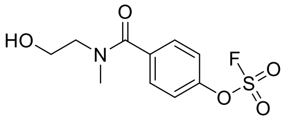 $C_{10}H_{12}FNO_5S$	277.266	0.546	4	1	6
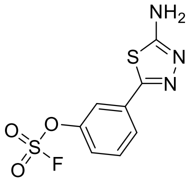 $C_8H_6FN_3O_3S_2$	275.272	2.411	5	1	3
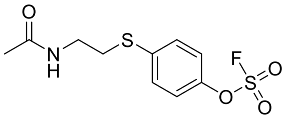 $C_{10}H_{12}FNO_4S_2$	293.327	1.159	3	1	7

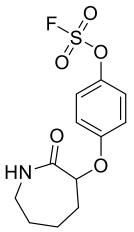 $C_{12}H_{14}FNO_5S$	303.304	1.297	4	1	4
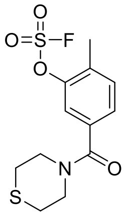 $C_{12}H_{14}FNO_4S_2$	319.365	1.872	3	0	4
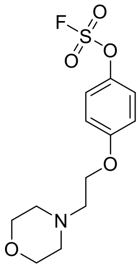 $C_{12}H_{16}FNO_5S$	305.32	1.159	5	0	6
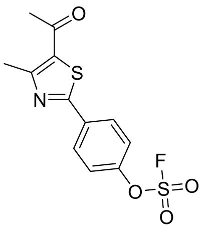 $C_{12}H_{10}FNO_4S_2$	315.333	2.549	4	0	4
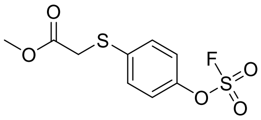 $C_9H_9FO_5S_2$	280.284	1.654	3	0	6
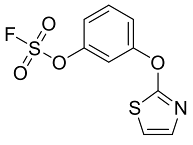 $C_9H_6FNO_4S_2$	275.268	2.942	4	0	4

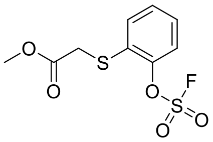 $C_9H_9FO_5S_2$	280.284	1.654	3	0	6
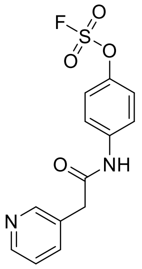 $C_{13}H_{11}FN_2O_4S$	310.299	1.098	4	1	6
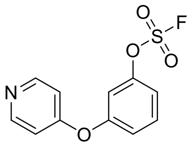 $C_{11}H_8FNO_4S$	269.246	1.885	4	0	4
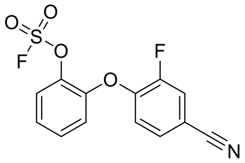 $C_{13}H_7F_2NO_4S$	311.259	3.414	5	0	5
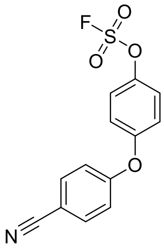 $C_{13}H_8FNO_4S$	293.268	3.256	4	0	5
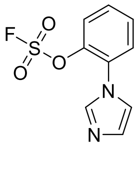 $C_9H_7FN_2O_3S$	242.224	0.885	4	0	3

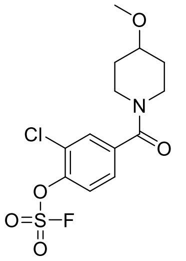 $C_{13}H_{15}ClFNO_5S$	351.773	1.413	4	0	5
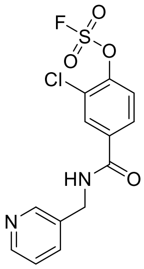 $C_{13}H_{10}ClFN_2O_4S$	344.741	1.781	4	1	6
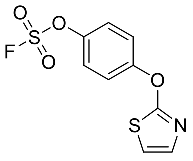 $C_9H_6FNO_4S_2$	275.268	2.942	4	0	4
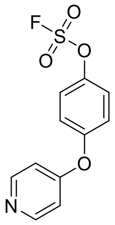 $C_{11}H_8FNO_4S$	269.246	1.885	4	0	4
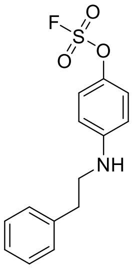 $C_{14}H_{14}FNO_3S$	295.328	3.194	3	1	6

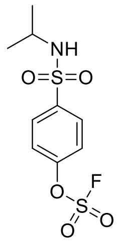 $C_9H_{12}FNO_5S_2$	297.315	2.921	4	1	5
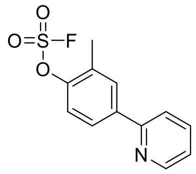 $C_{12}H_{10}FNO_3S$	267.274	2.934	3	0	3
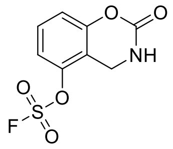 $C_8H_6FNO_5S$	247.196	0.877	3	1	2
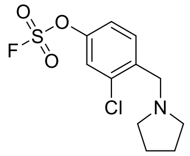 $C_{11}H_{13}ClFNO_3S$	293.737	2.501	3	0	4
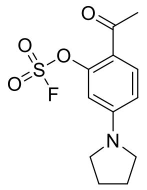 $C_{12}H_{14}FNO_4S$	287.305	1.598	4	0	4
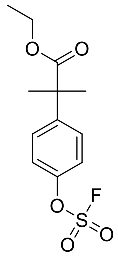 $C_{12}H_{15}FO_5S$	290.305	3.061	3	0	6

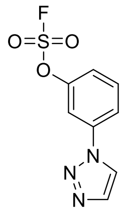 $C_8H_6FN_3O_3S$	243.212	1.291	5	0	3
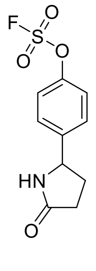 $C_{10}H_{10}FNO_4S$	259.251	0.788	3	1	3
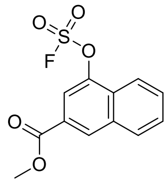 $C_{12}H_9FO_5S$	284.257	2.503	3	0	4
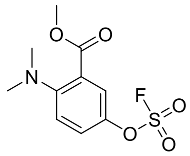 $C_{10}H_{12}FNO_5S$	277.266	1.79	4	0	5
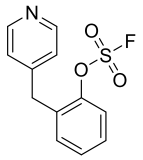 $C_{12}H_{10}FNO_3S$	267.274	2.441	3	0	4
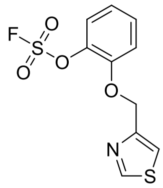 $C_{10}H_8FNO_4S_2$	289.295	2.453	4	0	5

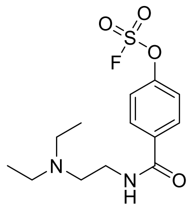 $C_{13}H_{19}FN_2O_4S$	**318.363**	**1.503**	**4**	**1**	**9**
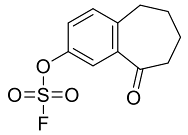 $C_{11}H_{11}FO_4S$	**258.263**	**2.057**	**3**	**0**	**2**
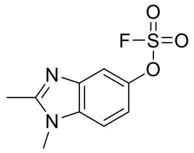 $C_9H_9FN_2O_3S$	**244.24**	**1.368**	**4**	**0**	**2**
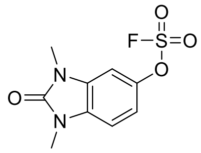 $C_9H_9FN_2O_4S$	**260.239**	**0.602**	**3**	**0**	**2**
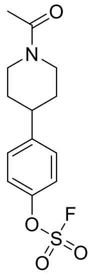 $C_{13}H_{16}FNO_4S$	**301.332**	**1.416**	**3**	**0**	**4**
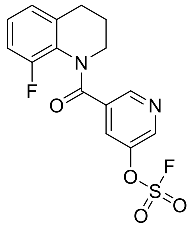 $C_{15}H_{12}F_2N_2O_4S$	**354.328**	**1.943**	**5**	**0**	**4**

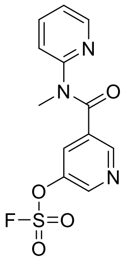 $C_{12}H_{10}FN_3O_4S$	311.287	0.768	5	0	5
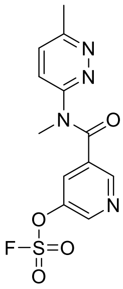 $C_{12}H_{11}FN_4O_4S$	326.302	1.079	6	0	5
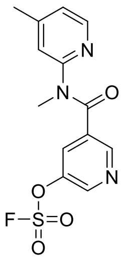 $C_{13}H_{12}FN_3O_4S$	325.314	1.255	5	0	5
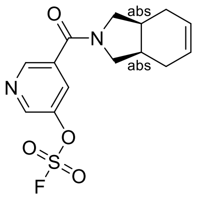 $C_{14}H_{15}FN_2O_4S$	326.342	0.856	4	0	4
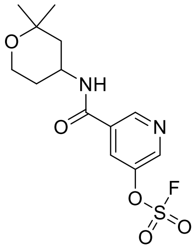 $C_{13}H_{17}FN_2O_5S$	332.346	-0.181	5	1	5

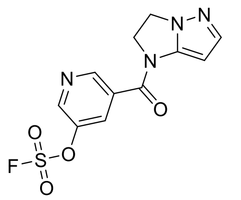 $C_{11}H_9FN_4O_4S$	312.275	-0.33	6	0	4
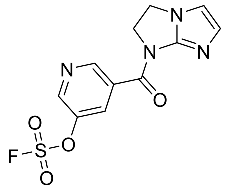 $C_{11}H_9FN_4O_4S$	312.275	-0.337	6	0	4
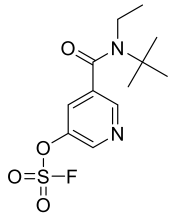 $C_{12}H_{17}FN_2O_4S$	304.336	0.939	4	0	6
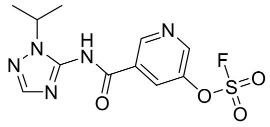 $C_{11}H_{12}FN_5O_4S$	329.306	0.679	7	1	6
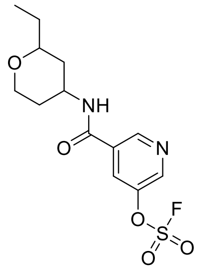 $C_{13}H_{17}FN_2O_5S$	332.346	0.087	5	1	6
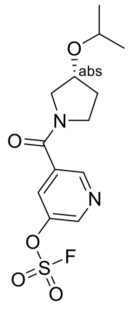 $C_{13}H_{17}FN_2O_5S$	332.346	0.069	5	0	6

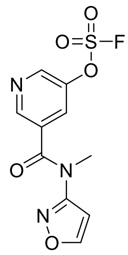 $C_{10}H_8FN_3O_5S$	301.248	0.278	6	0	5
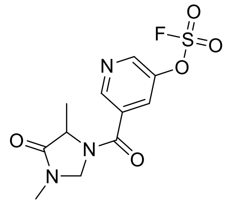 $C_{11}H_{12}FN_3O_5S$	317.291	-0.662	5	0	4
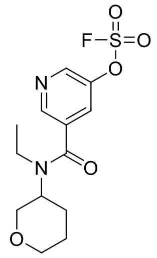 $C_{13}H_{17}FN_2O_5S$	332.346	0.204	5	0	6
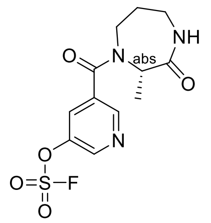 $C_{12}H_{14}FN_3O_5S$	331.318	-1.068	5	1	4
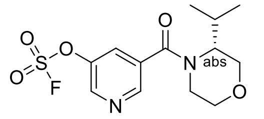 $C_{13}H_{17}FN_2O_5S$	332.346	0.53	5	0	5
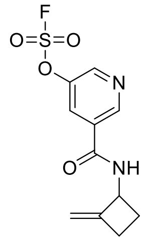 $C_{11}H_{11}FN_2O_4S$	286.277	0.247	4	1	5

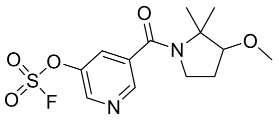 $C_{13}H_{17}FN_2O_5S$	332.346	-0.051	5	0	5
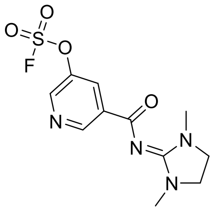 $C_{11}H_{13}FN_4O_4S$	316.307	0.678	6	0	4
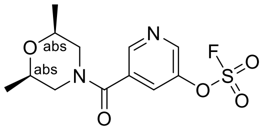 $C_{12}H_{15}FN_2O_5S$	318.319	-0.037	5	0	4
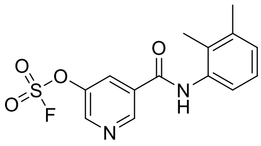 $C_{14}H_{13}FN_2O_4S$	324.326	2.128	4	1	5
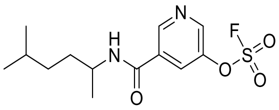 $C_{13}H_{19}FN_2O_4S$	318.363	1.797	4	1	8
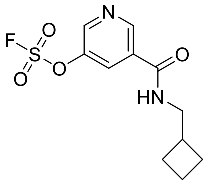 $C_{11}H_{13}FN_2O_4S$	288.293	0.632	4	1	6
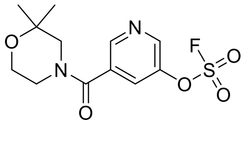 $C_{12}H_{15}FN_2O_5S$	318.319	-0.137	5	0	4

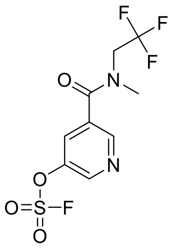 $C_9H_8F_4N_2O_4S$	316.227	0.705	7	0	6
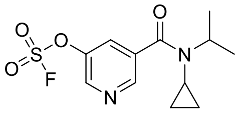 $C_{12}H_{15}FN_2O_4S$	302.32	0.678	4	0	6
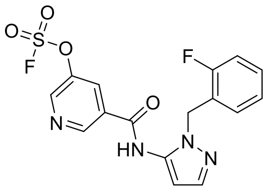 $C_{16}H_{12}F_2N_4O_4S$	394.353	1.569	7	1	7
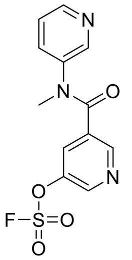 $C_{12}H_{10}FN_3O_4S$	311.287	0.052	5	0	5
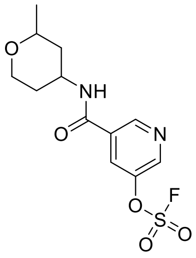 $C_{12}H_{15}FN_2O_5S$	318.319	-0.399	5	1	5
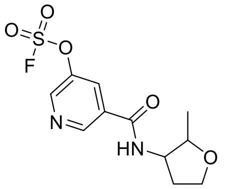 $C_{11}H_{13}FN_2O_5S$	304.292	-0.505	5	1	5

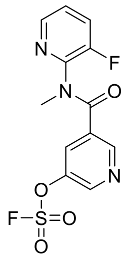 $C_{12}H_9F_2N_3O_4S$	329.278	0.926	6	0	5
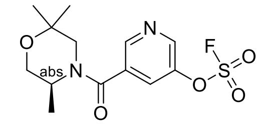 $C_{13}H_{17}FN_2O_5S$	332.346	0.181	5	0	4
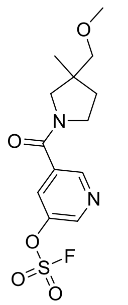 $C_{13}H_{17}FN_2O_5S$	332.346	0.279	5	0	6
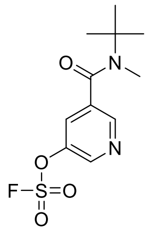 $C_{11}H_{15}FN_2O_4S$	290.309	0.6	4	0	5
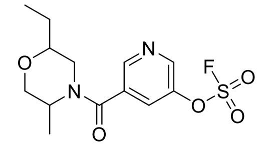 $C_{13}H_{17}FN_2O_5S$	332.346	0.449	5	0	5
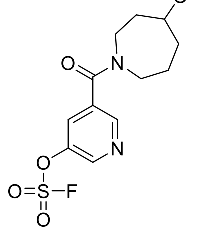 $C_{13}H_{17}FN_2O_5S$	332.346	-0.028	5	0	5

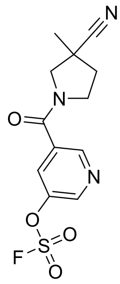<br>$C_{12}H_{12}FN_3O_4S$	313.303	0.526	5	0	5
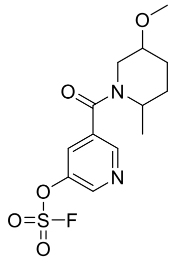<br>$C_{13}H_{17}FN_2O_5S$	332.346	0.185	5	0	5
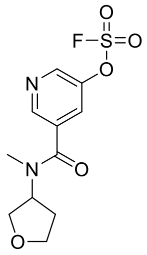<br>$C_{11}H_{13}FN_2O_5S$	304.292	-0.588	5	0	5
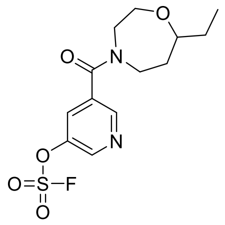<br>$C_{13}H_{17}FN_2O_5S$	332.346	0.237	5	0	5
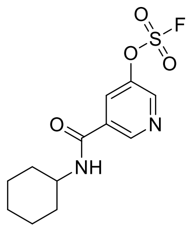<br>$C_{12}H_{15}FN_2O_4S$	302.32	1.038	4	1	5
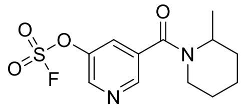	302.32	0.777	4	0	4

$C_{12}H_{15}FN_2O_4S$					
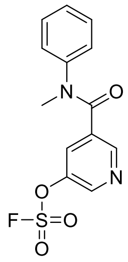 $C_{13}H_{11}FN_2O_4S$	310.299	1.389	4	0	5
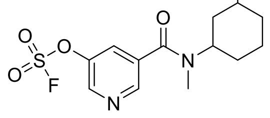 $C_{14}H_{19}FN_2O_4S$	330.374	1.604	4	0	5
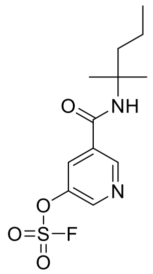 $C_{12}H_{17}FN_2O_4S$	304.336	1.268	4	1	7
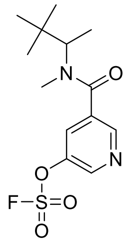 $C_{13}H_{19}FN_2O_4S$	318.363	1.807	4	0	6
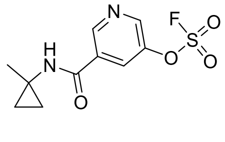 $C_{10}H_{11}FN_2O_4S$	274.266	0.004	4	1	5
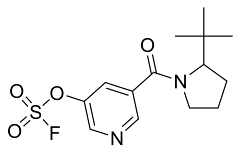 $C_{14}H_{19}FN_2O_4S$	330.374	1.784	4	0	5

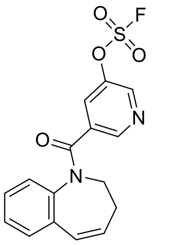 $C_{16}H_{13}FN_2O_4S$	348.348	1.883	4	0	4
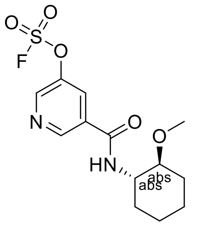 $C_{13}H_{17}FN_2O_5S$	332.346	0.445	5	1	6
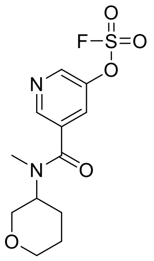 $C_{12}H_{15}FN_2O_5S$	318.319	-0.134	5	0	5
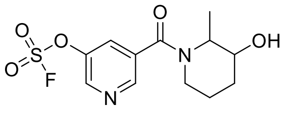 $C_{12}H_{15}FN_2O_5S$	318.319	-0.177	5	1	4
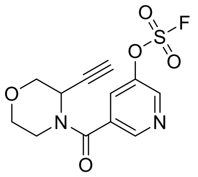 $C_{12}H_{11}FN_2O_5S$	314.287	-0.478	5	0	5
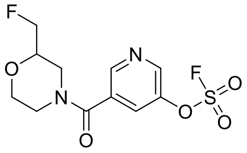 $C_{11}H_{12}F_2N_2O_5S$	322.283	-0.503	6	0	5

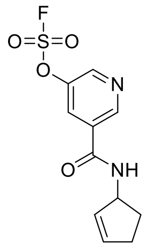 $C_{11}H_{11}FN_2O_4S$	286.277	0.439	4	1	5
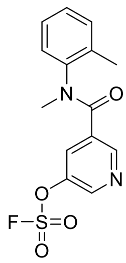 $C_{14}H_{13}FN_2O_4S$	324.326	1.877	4	0	5
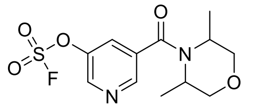 $C_{12}H_{15}FN_2O_5S$	318.319	-0.037	5	0	4
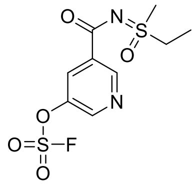 $C_9H_{11}FN_2O_5S_2$	310.314	0	5	0	5
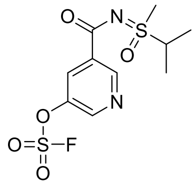 $C_{10}H_{13}FN_2O_5S_2$	324.341	0	5	0	5
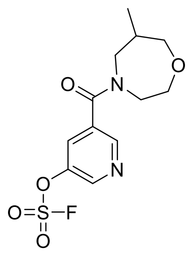 $C_{12}H_{15}FN_2O_5S$	318.319	-0.082	5	0	4

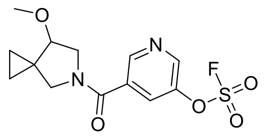 **$C_{13}H_{15}FN_2O_5S$**	**330.33**	**0.026**	**5**	**0**	**5**
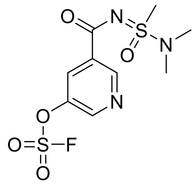 **$C_9H_{12}FN_3O_5S_2$**	**325.329**	**0**	**5**	**0**	**5**
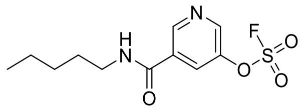 **$C_{11}H_{15}FN_2O_4S$**	**290.309**	**1.149**	**4**	**1**	**8**
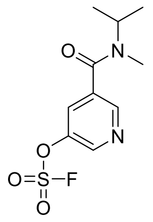 **$C_{10}H_{13}FN_2O_4S$**	**276.282**	**0.383**	**4**	**0**	**5**
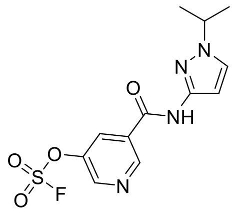 **$C_{12}H_{13}FN_4O_4S$**	**328.318**	**0.862**	**6**	**1**	**6**
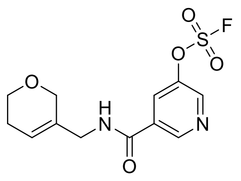 **$C_{12}H_{13}FN_2O_5S$**	**316.303**	**-0.483**	**5**	**1**	**6**

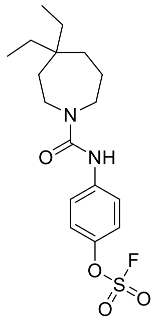 $C_{17}H_{25}FN_2O_4S$	372.455	3.521	3	1	7
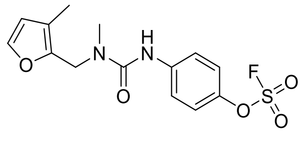 $C_{14}H_{15}FN_2O_5S$	342.341	1.572	4	1	7
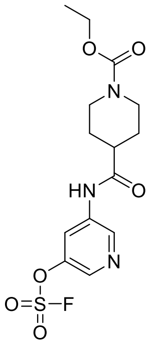 $C_{14}H_{18}FN_3O_6S$	375.371	-0.103	5	1	8
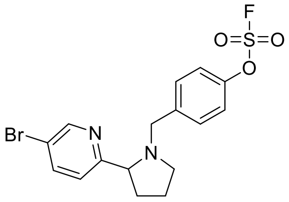 $C_{16}H_{16}BrFN_2O_3S$	415.277	3.571	4	0	5
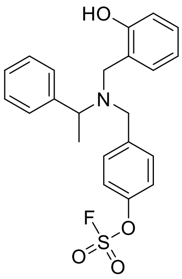 $C_{22}H_{22}FNO_4S$	415.479	5.022	4	1	8

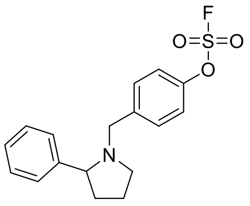<br>$C_{17}H_{18}FNO_3S$	335.393	3.656	3	0	5
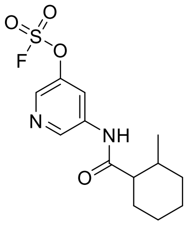<br>$C_{13}H_{17}FN_2O_4S$	316.347	1.56	4	1	5
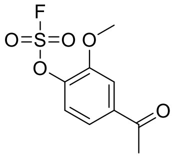<br>$C_9H_9FO_5S$	248.224	0.871	4	0	4
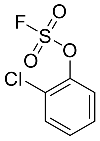<br>$C_6H_4ClFO_3S$	210.603	2.244	2	0	2
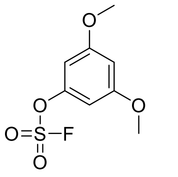<br>$C_8H_9FO_5S$	236.213	1.432	4	0	4
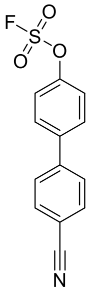<br>$C_{13}H_8FNO_3S$	277.269	3.394	3	0	4

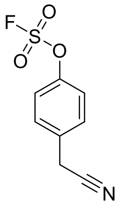 **$C_8H_6FNO_3S$**	**215.198**	**1.663**	**3**	**0**	**4**
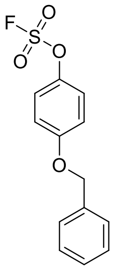 **$C_{13}H_{11}FO_4S$**	**282.285**	**3.292**	**3**	**0**	**5**
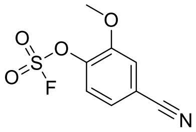 **$C_8H_6FNO_4S$**	**231.197**	**1.592**	**4**	**0**	**4**

Supplementary Table S2: Structures and characterization of the positive and negatives hits. Hits identified via mass spectrometry analysis (after incubation of hits 250 µM with protein 10 µM for 48 h) and denaturation thermal shift measurements with hMcl-1(172-323) and its mutants.
ND = not detected.

Structure	ΔTm Mcl-1 *wt*	% complex Mcl-1 *wt*	ΔTm Mcl-1 H224A	ΔTm Mcl-1 H252A	ΔTm Mcl-1 K234A
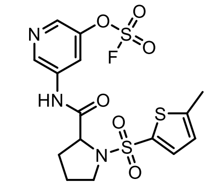 P1 A11 (hit 1)	2.11 ± 0.01	~ 55	-0.34 ± 0.09	2.90 ± 0.17	1.96 ± 0.17
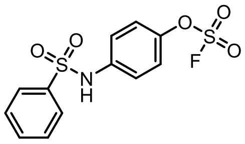 P1 D4	1.2 ± 0.01	~ 20	-0.35 ± 0.01	-0.35 ± 0.09	-0.27 ± 0.09
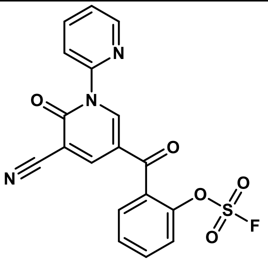 P1 H2	-1.36 ± 0.01	ND	-	-	-
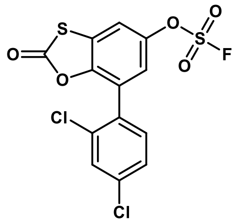 P2 B10	-4.03 ± 0.01	ND	-	-	-
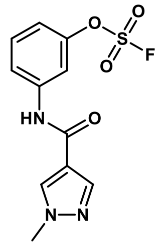 P3 B5	-1.41 ± 0.08	< 5	-0.44 ± 0.09	-0.35 ± 0.09	0.33 ± 0.17
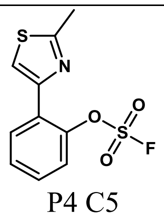 P4 C5	-1.21 ± 0.08	ND	-	-	-

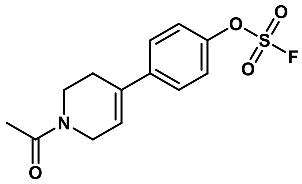 P4 D6	-1.46 ± 0.01	< 5	-1.89 ± 0.09	-1.29 ± 0.26	-0.70 ± 0.09
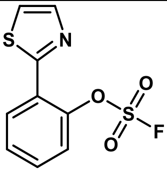 P4 D11	-7.5 ± 0.60	~ 35	-3.68 ± 1.19	-0.10 ± 0.09	-1.97 ± 0.01
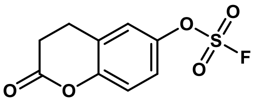 P4 E11	-2.55 ± 0.08	ND	-	-	-
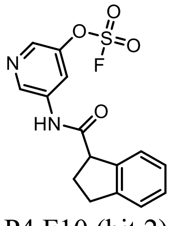 P4 F10 (hit 2)	3.97 ± 0.17	~ 95	-1.28 ± 0.17	6.57 ± 0.26	4.77 ± 0.26
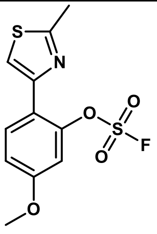 P4 H10	-1.82 ± 0.01	ND	-	-	-

Supplementary Table S3: Mass spectrometry data of hMcl-1(172-323) and its mutants. MS data collected in absence or presence of various agents as indicated. Molecular masses are measured and analyzed using an Agilent 6545 QTOF LC/MS mass spectrometer.

**ID**	**Calcd [M]**	**Obs. (m/z)**
**hMcl-1 *wt***	**19572.16**	**[M] = 19572**<br>**[M] +178 = 19750**
**hMcl-1 *wt* + hit 1**	**20001.66**	**[M] = 20001**<br>**$[M+H]^+$ +178 = 20180**
**hMcl-1 *wt* + hit 2**	**19888.5**	**[M] = 19888**<br>**$[M+H]^+$ +178 = 20067**
**hMcl-1 *wt* + 165D9**	**19922.2**	**$[M+H]^+$ = 19923**<br>**$[M+H]^+$ +178 = 20101**

**ID**	**Calcd [M]**	**Obs. (m/z)**
**hMcl-1 H224A**	**19506.09**	**[M] = 19506**<br>**[M] +178 = 19684**
**hMcl-1 H224A + hit 1**	**19935.59**	**[M] = 19935**
**hMcl-1 H224A + hit 2**	**19822.4**	**[M] = 19822**<br>**$[M+H]^+$ +178 = 20001**
**hMcl-1 H224A + 165D9**	**19856.11**	**$[M+H]^+$ = 19857**

**ID**	**Calcd [M]**	**Obs. (m/z)**
**^{15}N hMcl-1 H252A (no His tag)**	**17981**	**[M] = 17981**<br>**[M] +178 = 18159**
**^{15}N hMcl-1 H252A (no His tag) + hit 1**	**18410.5**	**$[M+H]^+$ = 18411**<br>**$[M+H]^+$ +178 = 18661**
**^{15}N hMcl-1 H252A (no His tag) + hit 2**	**18297.34**	**$[M+H]^+$ = 18298**<br>**$[M+H]^+$ +178 = 20001**
**^{15}N hMcl-1 H252A (no His tag) + 165D9**	**18331.02**	**$[M+H]^+$ = 18332**

**ID**	**Calcd [M]**	**Obs. (m/z)**
**hMcl-1 K234A**	**19515.06**	**[M] = 19515**<br>**[M] +178 = 19693**
**hMcl-1 K234A + hit 1**	**19944.56**	**[M] = 19944**<br>**$[M+H]^+$ +178 = 20123**
**hMcl-1 K234A + hit 2**	**19831.4**	**[M] = 19831**<br>**[M] +178 = 20009**
**hMcl-1 K234A + 165D9**	**19865.08**	**$[M+H]^+$ = 19866**<br>**$[M+H]^+$ +178 = 20044**

Supplementary Table S4. **High-resolution mass-spectrometry data of synthesized compounds**. All the mass-spectrometry data were collected using an Agilent 6545 QTOF LC/MS instrument.

ID	Calcd [M]	Obs. (m/z)
Compound 3 (165D6)	350.0373	$[M+H]^+$ = 351.0470
Compound 4 (165D7)	350.0737	$[M+H]^+$ = 351.0833
Compound 5 (165D9)	370.0190	$[M+H]^+$ = 371.0285
Compound 7 (165D12)	336.0580	$[M+H]^+$ = 337.0658
Compound 8 (165E12)	370.0190	$[M+H]^+$ = 371.0286
Compound 9 (165F6)	336.0580	$[M+H]^+$ = 337.0675

Supplementary Table S5: Data collection and refinement statistics for the complex between hMcl-1(172-323) and fragment hit 2.

	**hMcl-1(172-323)/hit2**
**Data collection**	
Beamline	DLS I-04
Wavelength	0.7460
Resolution range (Å)	39.03 - 1.82 (1.885 - 1.82)
Space group	P 1 21 1
Unit cell	41.556, 40.135, 42.052 (90, 111.868, 90)
R_{merge}[a]	0.08596 (4.649)
Total reflections	11680 (834)
Mean I/sigma(I)	12.29 (0.30)
Completeness (%)	96.96 (73.39)
Wilson B-factor	28.06
Multiplicity	7.0 (7.3)
**Refinement**	
Resolution	39.03 - 1.82 (1.885 - 1.82)
R_{work}	0.2536 (0.4440)
R_{free}	0.2869 (0.4703)
Unique reflections	11327 (833)
Number of non-hydrogen atoms	1227
macromolecules	1196
ligands	22
solvent	9
RMS bonds (Å)	0.009
RMS angles (°)	1.25
Ramachandran favored (%)	92.57
Ramachandran allowed (%)	7.43
Ramachandran outliers (%)	0.00
Rotamer outliers (%)	0.78
Clashscore	9.52

Average B-factor	49.39
macromolecules	49.41
ligands	46.97
solvent	52.37

Statistics for the highest-resolution shell are shown in parentheses.

```
HEADER    165D9_COMPLEX
COMPND    165D9_COMPLEX
REMARK    GENERATED BY SYBYL (TRIPOS, INC.)       22-MAR-25
SEQRES   1 A  150  ASP GLU LEU TYR ARG GLN SER LEU GLU ILE ILE SER ARG
SEQRES   2 A  150  TYR LEU ARG GLU GLN ALA THR GLY ALA LYS ASP THR LYS
SEQRES   3 A  150  PRO MET GLY ARG SER GLY ALA THR SER ARG LYS ALA LEU
SEQRES   4 A  150  GLU THR LEU ARG ARG VAL GLY ASP GLY VAL GLN ARG ASN
SEQRES   5 A  150  HIS GLU THR ALA PHE GLN GLY MET LEU ARG LYS LEU ASP
SEQRES   6 A  150  ILE LYS ASN GLU ASP ASP VAL LYS SER LEU SER ARG VAL
SEQRES   7 A  150  MET ILE HIS VAL PHE SER ASP GLY VAL THR ASN TRP GLY
SEQRES   8 A  150  ARG ILE VAL THR LEU ILE SER PHE GLY ALA PHE VAL ALA
SEQRES   9 A  150  LYS HIS LEU LYS THR ILE ASN GLN GLU SER CYS ILE GLU
SEQRES  10 A  150  PRO LEU ALA GLU SER ILE THR ASP VAL LEU VAL ARG THR
SEQRES  11 A  150  LYS ARG ASP TRP LEU VAL LYS GLN ARG GLY TRP ASP GLY
SEQRES  12 A  150  PHE VAL GLU PHE PHE HIS VAL
HET    DRG  A 401      34
ATOM      1  N   ASP A 172      -1.993   6.617  20.555  1.00 -0.37           N
ATOM      2  CA  ASP A 172      -0.861   6.872  21.448  1.00  0.12           C
ATOM      3  C   ASP A 172      -0.200   5.549  21.808  1.00  0.20           C
ATOM      4  O   ASP A 172       0.722   5.149  21.112  1.00 -0.39           O
ATOM      5  CB  ASP A 172      -1.375   7.753  22.619  1.00  0.04           C
ATOM      6  CG  ASP A 172      -0.300   8.087  23.607  1.00  0.04           C
ATOM      7  OD1 ASP A 172       0.890   8.067  23.208  1.00 -0.57           O
ATOM      8  OD2 ASP A 172      -0.644   8.383  24.779  1.00 -0.57           O
ATOM      9  HA  ASP A 172      -0.098   7.460  20.914  1.00  0.08           H
ATOM     10  HB2 ASP A 172      -1.744   8.711  22.226  1.00  0.05           H
ATOM     11  HB3 ASP A 172      -2.219   7.269  23.129  1.00  0.05           H
ATOM     12  H   ASP A 172      -2.259   5.673  20.371  1.00  0.14           H
ATOM     13 HNCA ASP A 172      -2.526   7.387  20.203  1.00  0.14           H
ATOM     14  N   GLU A 173      -0.648   4.794  22.834  1.00 -0.26           N
ATOM     15  CA  GLU A 173      -0.042   3.484  23.091  1.00  0.13           C
ATOM     16  C   GLU A 173      -0.070   2.635  21.833  1.00  0.20           C
ATOM     17  O   GLU A 173       0.948   2.079  21.452  1.00 -0.39           O
ATOM     18  CB  GLU A 173      -0.759   2.760  24.265  1.00 -0.00           C
ATOM     19  CG  GLU A 173      -0.340   1.274  24.435  1.00  0.00           C
ATOM     20  CD  GLU A 173       1.140   1.062  24.562  1.00  0.04           C
ATOM     21  OE1 GLU A 173       1.900   2.014  24.881  1.00 -0.57           O
ATOM     22  OE2 GLU A 173       1.613  -0.084  24.347  1.00 -0.57           O
ATOM     23  HA  GLU A 173       1.010   3.648  23.362  1.00  0.08           H
ATOM     24  HB2 GLU A 173      -0.544   3.305  25.199  1.00  0.03           H
ATOM     25  HB3 GLU A 173      -1.849   2.785  24.098  1.00  0.03           H
ATOM     26  HG2 GLU A 173      -0.809   0.864  25.343  1.00  0.04           H
ATOM     27  HG3 GLU A 173      -0.688   0.686  23.574  1.00  0.04           H
ATOM     28  H   GLU A 173      -1.375   5.120  23.433  1.00  0.19           H
ATOM     29  N   LEU A 174      -1.223   2.521  21.138  1.00 -0.26           N
ATOM     30  CA  LEU A 174      -1.241   1.780  19.871  1.00  0.13           C
ATOM     31  C   LEU A 174      -0.071   2.184  18.994  1.00  0.20           C
ATOM     32  O   LEU A 174       0.564   1.330  18.393  1.00 -0.39           O
ATOM     33  CB  LEU A 174      -2.563   2.079  19.103  1.00 -0.01           C
ATOM     34  CG  LEU A 174      -2.666   1.465  17.672  1.00 -0.04           C
ATOM     35  CD1 LEU A 174      -2.654  -0.090  17.704  1.00 -0.06           C
```

ATOM	36	CD2	LEU	A	174	-3.964	1.973	16.976	1.00	-0.06	C
ATOM	37	HA	LEU	A	174	-1.163	0.706	20.101	1.00	0.08	H
ATOM	38	HB2	LEU	A	174	-3.415	1.727	19.708	1.00	0.03	H
ATOM	39	HB3	LEU	A	174	-2.644	3.174	19.007	1.00	0.03	H
ATOM	40	HG	LEU	A	174	-1.809	1.806	17.062	1.00	0.03	H
ATOM	41	HD21	LEU	A	174	-3.981	3.073	16.926	1.00	0.02	H
ATOM	42	HD22	LEU	A	174	-4.021	1.585	15.948	1.00	0.02	H
ATOM	43	HD23	LEU	A	174	-4.855	1.635	17.529	1.00	0.02	H
ATOM	44	HD11	LEU	A	174	-3.471	-0.468	18.340	1.00	0.02	H
ATOM	45	HD12	LEU	A	174	-2.789	-0.495	16.688	1.00	0.02	H
ATOM	46	HD13	LEU	A	174	-1.697	-0.473	18.089	1.00	0.02	H
ATOM	47	H	LEU	A	174	-2.059	2.943	21.486	1.00	0.19	H
ATOM	48	N	TYR	A	175	0.232	3.498	18.905	1.00	-0.26	N
ATOM	49	CA	TYR	A	175	1.366	3.934	18.094	1.00	0.14	C
ATOM	50	C	TYR	A	175	2.650	3.439	18.716	1.00	0.21	C
ATOM	51	O	TYR	A	175	3.486	2.888	18.014	1.00	-0.39	O
ATOM	52	CB	TYR	A	175	1.383	5.481	17.954	1.00	0.02	C
ATOM	53	CG	TYR	A	175	2.575	5.965	17.123	1.00	-0.05	C
ATOM	54	CD1	TYR	A	175	3.843	6.050	17.709	1.00	-0.07	C
ATOM	55	CD2	TYR	A	175	2.415	6.339	15.784	1.00	-0.07	C
ATOM	56	CE1	TYR	A	175	4.947	6.469	16.962	1.00	-0.04	C
ATOM	57	CE2	TYR	A	175	3.515	6.804	15.058	1.00	-0.04	C
ATOM	58	CZ	TYR	A	175	4.794	6.817	15.617	1.00	0.08	C
ATOM	59	OH	TYR	A	175	5.885	7.176	14.822	1.00	-0.34	O
ATOM	60	HA	TYR	A	175	1.287	3.496	17.091	1.00	0.08	H
ATOM	61	HB3	TYR	A	175	0.433	5.811	17.503	1.00	0.05	H
ATOM	62	HB2	TYR	A	175	1.455	5.962	18.941	1.00	0.05	H
ATOM	63	HD2	TYR	A	175	1.442	6.277	15.305	1.00	0.05	H
ATOM	64	HE2	TYR	A	175	3.395	7.169	14.048	1.00	0.05	H
ATOM	65	HE1	TYR	A	175	5.919	6.527	17.439	1.00	0.05	H
ATOM	66	HD1	TYR	A	175	3.983	5.798	18.755	1.00	0.05	H
ATOM	67	HH	TYR	A	175	6.718	7.162	15.278	1.00	0.25	H
ATOM	68	H	TYR	A	175	-0.252	4.178	19.458	1.00	0.19	H
ATOM	69	N	ARG	A	176	2.826	3.635	20.041	1.00	-0.26	N
ATOM	70	CA	ARG	A	176	4.062	3.184	20.679	1.00	0.13	C
ATOM	71	C	ARG	A	176	4.263	1.701	20.428	1.00	0.20	C
ATOM	72	O	ARG	A	176	5.331	1.285	20.002	1.00	-0.39	O
ATOM	73	CB	ARG	A	176	4.017	3.433	22.213	1.00	-0.01	C
ATOM	74	CG	ARG	A	176	5.385	3.163	22.901	1.00	-0.02	C
ATOM	75	CD	ARG	A	176	5.205	2.832	24.408	1.00	0.06	C
ATOM	76	NE	ARG	A	176	4.437	1.589	24.555	1.00	-0.27	N
ATOM	77	CZ	ARG	A	176	4.907	0.376	24.357	1.00	0.29	C
ATOM	78	NH1	ARG	A	176	6.158	0.136	24.052	1.00	-0.28	N
ATOM	79	NH2	ARG	A	176	4.092	-0.645	24.463	1.00	-0.28	N
ATOM	80	HA	ARG	A	176	4.906	3.741	20.246	1.00	0.08	H
ATOM	81	HB2	ARG	A	176	3.710	4.473	22.407	1.00	0.03	H
ATOM	82	HB3	ARG	A	176	3.249	2.780	22.650	1.00	0.03	H
ATOM	83	HG2	ARG	A	176	5.884	2.317	22.412	1.00	0.03	H
ATOM	84	HG3	ARG	A	176	6.032	4.046	22.777	1.00	0.03	H
ATOM	85	HD2	ARG	A	176	6.166	2.793	24.940	1.00	0.07	H
ATOM	86	HD3	ARG	A	176	4.644	3.669	24.863	1.00	0.07	H

```
ATOM     87  HE  ARG A 176       3.443   1.694  24.835  1.00  0.26           H
ATOM     88 HH12 ARG A 176       6.504  -0.828  23.907  1.00  0.26           H
ATOM     89 HH11 ARG A 176       6.848   0.880  23.957  1.00  0.26           H
ATOM     90 HH22 ARG A 176       4.413  -1.610  24.303  1.00  0.26           H
ATOM     91 HH21 ARG A 176       3.087  -0.507  24.684  1.00  0.26           H
ATOM     92  H   ARG A 176       2.108   4.076  20.596  1.00  0.19           H
ATOM     93  N   GLN A 177       3.229   0.878  20.703  1.00 -0.26           N
ATOM     94  CA  GLN A 177       3.375  -0.566  20.536  1.00  0.13           C
ATOM     95  C   GLN A 177       3.631  -0.897  19.080  1.00  0.20           C
ATOM     96  O   GLN A 177       4.535  -1.664  18.779  1.00 -0.39           O
ATOM     97  CB  GLN A 177       2.100  -1.297  21.043  1.00  0.00           C
ATOM     98  CG  GLN A 177       2.140  -2.825  20.780  1.00  0.04           C
ATOM     99  CD  GLN A 177       1.046  -3.591  21.486  1.00  0.17           C
ATOM    100  OE1 GLN A 177       1.342  -4.621  22.076  1.00 -0.40           O
ATOM    101  NE2 GLN A 177      -0.234  -3.174  21.480  1.00 -0.30           N
ATOM    102  HA  GLN A 177       4.238  -0.907  21.130  1.00  0.08           H
ATOM    103  HB2 GLN A 177       2.004  -1.118  22.124  1.00  0.03           H
ATOM    104  HB3 GLN A 177       1.224  -0.858  20.540  1.00  0.03           H
ATOM    105  HG2 GLN A 177       2.047  -3.017  19.704  1.00  0.05           H
ATOM    106  HG3 GLN A 177       3.111  -3.218  21.118  1.00  0.05           H
ATOM    107 HE22 GLN A 177      -0.909  -3.727  21.972  1.00  0.18           H
ATOM    108 HE21 GLN A 177      -0.531  -2.336  21.025  1.00  0.18           H
ATOM    109  H   GLN A 177       2.352   1.253  21.023  1.00  0.19           H
ATOM    110  N   SER A 178       2.842  -0.339  18.136  1.00 -0.26           N
ATOM    111  CA  SER A 178       3.130  -0.617  16.731  1.00  0.15           C
ATOM    112  C   SER A 178       4.555  -0.219  16.396  1.00  0.21           C
ATOM    113  O   SER A 178       5.240  -0.967  15.712  1.00 -0.39           O
ATOM    114  CB  SER A 178       2.136   0.133  15.810  1.00  0.08           C
ATOM    115  OG  SER A 178       0.799  -0.363  16.025  1.00 -0.39           O
ATOM    116  HA  SER A 178       3.024  -1.697  16.547  1.00  0.08           H
ATOM    117  HB2 SER A 178       2.187   1.211  16.045  1.00  0.06           H
ATOM    118  HB3 SER A 178       2.422  -0.023  14.755  1.00  0.06           H
ATOM    119  HG  SER A 178       0.152   0.124  15.519  1.00  0.21           H
ATOM    120  H   SER A 178       2.085   0.283  18.376  1.00  0.19           H
ATOM    121  N   LEU A 179       5.034   0.955  16.868  1.00 -0.26           N
ATOM    122  CA  LEU A 179       6.416   1.329  16.578  1.00  0.13           C
ATOM    123  C   LEU A 179       7.365   0.300  17.146  1.00  0.20           C
ATOM    124  O   LEU A 179       8.254  -0.169  16.451  1.00 -0.39           O
ATOM    125  CB  LEU A 179       6.792   2.729  17.138  1.00 -0.01           C
ATOM    126  CG  LEU A 179       8.267   3.151  16.864  1.00 -0.04           C
ATOM    127  CD1 LEU A 179       8.576   3.212  15.344  1.00 -0.06           C
ATOM    128  CD2 LEU A 179       8.550   4.537  17.503  1.00 -0.06           C
ATOM    129  HA  LEU A 179       6.522   1.326  15.488  1.00  0.08           H
ATOM    130  HB2 LEU A 179       6.123   3.489  16.709  1.00  0.03           H
ATOM    131  HB3 LEU A 179       6.628   2.720  18.225  1.00  0.03           H
ATOM    132  HG  LEU A 179       8.946   2.424  17.340  1.00  0.03           H
ATOM    133 HD21 LEU A 179       8.295   4.537  18.574  1.00  0.02           H
ATOM    134 HD22 LEU A 179       9.616   4.793  17.405  1.00  0.02           H
ATOM    135 HD23 LEU A 179       7.964   5.318  17.001  1.00  0.02           H
ATOM    136 HD11 LEU A 179       7.799   3.793  14.828  1.00  0.02           H
ATOM    137 HD12 LEU A 179       9.550   3.690  15.162  1.00  0.02           H
```

ATOM	138	HD13	LEU	A	179	8.614	2.206	14.907	1.00	0.02	H
ATOM	139	H	LEU	A	179	4.469	1.565	17.433	1.00	0.19	H
ATOM	140	N	GLU	A	180	7.183	-0.053	18.435	1.00	-0.26	N
ATOM	141	CA	GLU	A	180	8.085	-1.015	19.075	1.00	0.13	C
ATOM	142	C	GLU	A	180	8.202	-2.290	18.262	1.00	0.20	C
ATOM	143	O	GLU	A	180	9.295	-2.813	18.096	1.00	-0.39	O
ATOM	144	CB	GLU	A	180	7.522	-1.330	20.490	1.00	-0.00	C
ATOM	145	CG	GLU	A	180	8.387	-2.312	21.317	1.00	0.00	C
ATOM	146	CD	GLU	A	180	7.709	-2.486	22.649	1.00	0.04	C
ATOM	147	OE1	GLU	A	180	6.959	-3.477	22.820	1.00	-0.57	O
ATOM	148	OE2	GLU	A	180	7.879	-1.617	23.556	1.00	-0.57	O
ATOM	149	HA	GLU	A	180	9.085	-0.562	19.166	1.00	0.08	H
ATOM	150	HB2	GLU	A	180	7.438	-0.384	21.053	1.00	0.03	H
ATOM	151	HB3	GLU	A	180	6.514	-1.764	20.389	1.00	0.03	H
ATOM	152	HG2	GLU	A	180	8.458	-3.291	20.816	1.00	0.04	H
ATOM	153	HG3	GLU	A	180	9.399	-1.904	21.457	1.00	0.04	H
ATOM	154	H	GLU	A	180	6.426	0.350	18.969	1.00	0.19	H
ATOM	155	N	ILE	A	181	7.063	-2.806	17.754	1.00	-0.26	N
ATOM	156	CA	ILE	A	181	7.097	-4.042	16.966	1.00	0.13	C
ATOM	157	C	ILE	A	181	7.808	-3.803	15.649	1.00	0.20	C
ATOM	158	O	ILE	A	181	8.717	-4.537	15.285	1.00	-0.39	O
ATOM	159	CB	ILE	A	181	5.642	-4.547	16.706	1.00	-0.00	C
ATOM	160	CG1	ILE	A	181	4.991	-4.999	18.047	1.00	-0.05	C
ATOM	161	CG2	ILE	A	181	5.606	-5.689	15.650	1.00	-0.06	C
ATOM	162	CD1	ILE	A	181	3.442	-5.059	17.981	1.00	-0.06	C
ATOM	163	HA	ILE	A	181	7.651	-4.821	17.513	1.00	0.08	H
ATOM	164	HB	ILE	A	181	5.054	-3.711	16.291	1.00	0.03	H
ATOM	165	HG12	ILE	A	181	5.401	-5.982	18.322	1.00	0.03	H
ATOM	166	HG13	ILE	A	181	5.247	-4.295	18.854	1.00	0.03	H
ATOM	167	HD11	ILE	A	181	3.095	-5.808	17.260	1.00	0.02	H
ATOM	168	HD12	ILE	A	181	3.020	-5.315	18.965	1.00	0.02	H
ATOM	169	HD13	ILE	A	181	3.047	-4.077	17.682	1.00	0.02	H
ATOM	170	HG21	ILE	A	181	6.198	-6.550	15.989	1.00	0.02	H
ATOM	171	HG22	ILE	A	181	4.578	-6.028	15.461	1.00	0.02	H
ATOM	172	HG23	ILE	A	181	6.013	-5.340	14.690	1.00	0.02	H
ATOM	173	H	ILE	A	181	6.188	-2.322	17.892	1.00	0.19	H
ATOM	174	N	ILE	A	182	7.383	-2.773	14.891	1.00	-0.26	N
ATOM	175	CA	ILE	A	182	7.915	-2.607	13.536	1.00	0.13	C
ATOM	176	C	ILE	A	182	9.397	-2.281	13.631	1.00	0.20	C
ATOM	177	O	ILE	A	182	10.206	-2.860	12.920	1.00	-0.39	O
ATOM	178	CB	ILE	A	182	7.039	-1.566	12.769	1.00	-0.00	C
ATOM	179	CG1	ILE	A	182	5.610	-2.164	12.562	1.00	-0.05	C
ATOM	180	CG2	ILE	A	182	7.679	-1.166	11.411	1.00	-0.06	C
ATOM	181	CD1	ILE	A	182	4.539	-1.126	12.141	1.00	-0.06	C
ATOM	182	HA	ILE	A	182	7.847	-3.565	12.997	1.00	0.08	H
ATOM	183	HB	ILE	A	182	6.970	-0.649	13.382	1.00	0.03	H
ATOM	184	HG12	ILE	A	182	5.655	-2.961	11.805	1.00	0.03	H
ATOM	185	HG13	ILE	A	182	5.243	-2.622	13.494	1.00	0.03	H
ATOM	186	HD11	ILE	A	182	4.782	-0.672	11.172	1.00	0.02	H
ATOM	187	HD12	ILE	A	182	3.560	-1.623	12.049	1.00	0.02	H
ATOM	188	HD13	ILE	A	182	4.459	-0.333	12.900	1.00	0.02	H

ATOM 189 HG21 ILE A 182 7.708 -2.023 10.723 1.00 0.02 H
ATOM 190 HG22 ILE A 182 7.112 -0.352 10.934 1.00 0.02 H
ATOM 191 HG23 ILE A 182 8.704 -0.806 11.569 1.00 0.02 H
ATOM 192 H ILE A 182 6.708 -2.120 15.259 1.00 0.19 H
ATOM 193 N SER A 183 9.777 -1.359 14.538 1.00 -0.26 N
ATOM 194 CA SER A 183 11.196 -1.047 14.723 1.00 0.15 C
ATOM 195 C SER A 183 12.052 -2.273 14.967 1.00 0.21 C
ATOM 196 O SER A 183 13.054 -2.469 14.291 1.00 -0.39 O
ATOM 197 CB SER A 183 11.353 -0.052 15.903 1.00 0.08 C
ATOM 198 OG SER A 183 12.735 0.103 16.265 1.00 -0.39 O
ATOM 199 HA SER A 183 11.596 -0.570 13.819 1.00 0.08 H
ATOM 200 HB2 SER A 183 10.877 0.894 15.577 1.00 0.06 H
ATOM 201 HB3 SER A 183 10.816 -0.417 16.793 1.00 0.06 H
ATOM 202 HG SER A 183 12.871 1.035 16.526 1.00 0.21 H
ATOM 203 H SER A 183 9.086 -0.895 15.105 1.00 0.19 H
ATOM 204 N ARG A 184 11.680 -3.127 15.943 1.00 -0.26 N
ATOM 205 CA ARG A 184 12.512 -4.300 16.206 1.00 0.13 C
ATOM 206 C ARG A 184 12.536 -5.222 14.998 1.00 0.20 C
ATOM 207 O ARG A 184 13.598 -5.741 14.682 1.00 -0.39 O
ATOM 208 CB ARG A 184 12.034 -5.050 17.481 1.00 -0.01 C
ATOM 209 CG ARG A 184 12.404 -4.292 18.793 1.00 -0.02 C
ATOM 210 CD ARG A 184 11.738 -4.924 20.047 1.00 0.06 C
ATOM 211 NE ARG A 184 12.108 -4.215 21.283 1.00 -0.27 N
ATOM 212 CZ ARG A 184 12.980 -4.623 22.185 1.00 0.29 C
ATOM 213 NH1 ARG A 184 13.738 -5.686 22.075 1.00 -0.28 N
ATOM 214 NH2 ARG A 184 13.106 -3.923 23.286 1.00 -0.28 N
ATOM 215 HA ARG A 184 13.551 -3.969 16.375 1.00 0.08 H
ATOM 216 HB2 ARG A 184 10.944 -5.177 17.414 1.00 0.03 H
ATOM 217 HB3 ARG A 184 12.493 -6.053 17.509 1.00 0.03 H
ATOM 218 HG2 ARG A 184 13.498 -4.304 18.930 1.00 0.03 H
ATOM 219 HG3 ARG A 184 12.077 -3.244 18.716 1.00 0.03 H
ATOM 220 HD2 ARG A 184 10.649 -4.799 19.917 1.00 0.07 H
ATOM 221 HD3 ARG A 184 11.898 -6.011 20.067 1.00 0.07 H
ATOM 222 HE ARG A 184 11.578 -3.350 21.475 1.00 0.26 H
ATOM 223 HH12 ARG A 184 14.401 -5.955 22.825 1.00 0.26 H
ATOM 224 HH11 ARG A 184 13.748 -6.268 21.234 1.00 0.26 H
ATOM 225 HH22 ARG A 184 13.736 -4.223 24.050 1.00 0.26 H
ATOM 226 HH21 ARG A 184 12.562 -3.069 23.462 1.00 0.26 H
ATOM 227 H ARG A 184 10.829 -2.979 16.458 1.00 0.19 H
ATOM 228 N TYR A 185 11.401 -5.458 14.298 1.00 -0.26 N
ATOM 229 CA TYR A 185 11.442 -6.385 13.159 1.00 0.14 C
ATOM 230 C TYR A 185 12.360 -5.870 12.071 1.00 0.21 C
ATOM 231 O TYR A 185 13.177 -6.621 11.558 1.00 -0.39 O
ATOM 232 CB TYR A 185 10.033 -6.605 12.542 1.00 0.02 C
ATOM 233 CG TYR A 185 10.012 -7.640 11.401 1.00 -0.05 C
ATOM 234 CD1 TYR A 185 10.434 -8.958 11.605 1.00 -0.07 C
ATOM 235 CD2 TYR A 185 9.547 -7.279 10.129 1.00 -0.07 C
ATOM 236 CE1 TYR A 185 10.442 -9.878 10.548 1.00 -0.04 C
ATOM 237 CE2 TYR A 185 9.489 -8.214 9.094 1.00 -0.04 C
ATOM 238 CZ TYR A 185 9.979 -9.510 9.283 1.00 0.08 C
ATOM 239 OH TYR A 185 10.002 -10.396 8.201 1.00 -0.34 O

ATOM	240	HA	TYR	A	185	11.838	-7.348	13.516	1.00	0.08	H
ATOM	241	HB3	TYR	A	185	9.342	-6.959	13.317	1.00	0.05	H
ATOM	242	HB2	TYR	A	185	9.654	-5.631	12.193	1.00	0.05	H
ATOM	243	HD2	TYR	A	185	9.225	-6.263	9.942	1.00	0.05	H
ATOM	244	HE2	TYR	A	185	9.066	-7.940	8.134	1.00	0.05	H
ATOM	245	HE1	TYR	A	185	10.812	-10.882	10.722	1.00	0.05	H
ATOM	246	HD1	TYR	A	185	10.757	-9.286	12.588	1.00	0.05	H
ATOM	247	HH	TYR	A	185	10.429	-11.236	8.390	1.00	0.25	H
ATOM	248	H	TYR	A	185	10.538	-4.998	14.543	1.00	0.19	H
ATOM	249	N	LEU	A	186	12.240	-4.578	11.697	1.00	-0.26	N
ATOM	250	CA	LEU	A	186	13.118	-4.047	10.652	1.00	0.13	C
ATOM	251	C	LEU	A	186	14.565	-4.122	11.096	1.00	0.20	C
ATOM	252	O	LEU	A	186	15.399	-4.583	10.329	1.00	-0.39	O
ATOM	253	CB	LEU	A	186	12.753	-2.579	10.296	1.00	-0.01	C
ATOM	254	CG	LEU	A	186	11.633	-2.434	9.221	1.00	-0.04	C
ATOM	255	CD1	LEU	A	186	10.401	-3.345	9.457	1.00	-0.06	C
ATOM	256	CD2	LEU	A	186	11.188	-0.951	9.142	1.00	-0.06	C
ATOM	257	HA	LEU	A	186	13.041	-4.661	9.741	1.00	0.08	H
ATOM	258	HB2	LEU	A	186	12.493	-2.031	11.216	1.00	0.03	H
ATOM	259	HB3	LEU	A	186	13.649	-2.098	9.875	1.00	0.03	H
ATOM	260	HG	LEU	A	186	12.048	-2.715	8.239	1.00	0.03	H
ATOM	261	HD21	LEU	A	186	12.058	-0.287	9.035	1.00	0.02	H
ATOM	262	HD22	LEU	A	186	10.521	-0.801	8.283	1.00	0.02	H
ATOM	263	HD23	LEU	A	186	10.653	-0.669	10.058	1.00	0.02	H
ATOM	264	HD11	LEU	A	186	9.949	-3.143	10.436	1.00	0.02	H
ATOM	265	HD12	LEU	A	186	9.643	-3.169	8.678	1.00	0.02	H
ATOM	266	HD13	LEU	A	186	10.690	-4.404	9.409	1.00	0.02	H
ATOM	267	H	LEU	A	186	11.582	-3.971	12.162	1.00	0.19	H
ATOM	268	N	ARG	A	187	14.901	-3.670	12.326	1.00	-0.26	N
ATOM	269	CA	ARG	A	187	16.300	-3.753	12.746	1.00	0.13	C
ATOM	270	C	ARG	A	187	16.761	-5.197	12.718	1.00	0.20	C
ATOM	271	O	ARG	A	187	17.783	-5.483	12.110	1.00	-0.39	O
ATOM	272	CB	ARG	A	187	16.542	-3.121	14.151	1.00	-0.01	C
ATOM	273	CG	ARG	A	187	16.517	-1.568	14.075	1.00	-0.02	C
ATOM	274	CD	ARG	A	187	16.786	-0.863	15.434	1.00	0.06	C
ATOM	275	NE	ARG	A	187	15.584	-0.833	16.280	1.00	-0.27	N
ATOM	276	CZ	ARG	A	187	15.419	-1.469	17.423	1.00	0.29	C
ATOM	277	NH1	ARG	A	187	16.291	-2.285	17.963	1.00	-0.28	N
ATOM	278	NH2	ARG	A	187	14.305	-1.275	18.086	1.00	-0.28	N
ATOM	279	HA	ARG	A	187	16.920	-3.218	12.006	1.00	0.08	H
ATOM	280	HB2	ARG	A	187	15.783	-3.485	14.862	1.00	0.03	H
ATOM	281	HB3	ARG	A	187	17.533	-3.437	14.520	1.00	0.03	H
ATOM	282	HG2	ARG	A	187	17.307	-1.254	13.371	1.00	0.03	H
ATOM	283	HG3	ARG	A	187	15.550	-1.223	13.676	1.00	0.03	H
ATOM	284	HD2	ARG	A	187	17.723	-1.239	15.862	1.00	0.07	H
ATOM	285	HD3	ARG	A	187	16.994	0.199	15.210	1.00	0.07	H
ATOM	286	HE	ARG	A	187	14.839	-0.176	15.957	1.00	0.26	H
ATOM	287	HH12	ARG	A	187	16.129	-2.704	18.898	1.00	0.26	H
ATOM	288	HH11	ARG	A	187	17.163	-2.538	17.499	1.00	0.26	H
ATOM	289	HH22	ARG	A	187	14.140	-1.710	19.002	1.00	0.26	H
ATOM	290	HH21	ARG	A	187	13.562	-0.677	17.706	1.00	0.26	H

```
ATOM    291  H   ARG A 187      14.206  -3.301  12.956  1.00  0.19           H
ATOM    292  N   GLU A 188      16.028  -6.130  13.363  1.00 -0.26           N
ATOM    293  CA  GLU A 188      16.487  -7.522  13.373  1.00  0.13           C
ATOM    294  C   GLU A 188      16.622  -8.042  11.957  1.00  0.20           C
ATOM    295  O   GLU A 188      17.610  -8.686  11.636  1.00 -0.39           O
ATOM    296  CB  GLU A 188      15.511  -8.406  14.197  1.00 -0.00           C
ATOM    297  CG  GLU A 188      15.959  -9.889  14.296  1.00  0.00           C
ATOM    298  CD  GLU A 188      15.075 -10.650  15.251  1.00  0.04           C
ATOM    299  OE1 GLU A 188      14.602 -10.084  16.273  1.00 -0.57           O
ATOM    300  OE2 GLU A 188      14.774 -11.854  15.032  1.00 -0.57           O
ATOM    301  HA  GLU A 188      17.484  -7.555  13.842  1.00  0.08           H
ATOM    302  HB2 GLU A 188      15.457  -7.983  15.212  1.00  0.03           H
ATOM    303  HB3 GLU A 188      14.503  -8.368  13.751  1.00  0.03           H
ATOM    304  HG2 GLU A 188      15.912 -10.361  13.303  1.00  0.04           H
ATOM    305  HG3 GLU A 188      16.993  -9.950  14.665  1.00  0.04           H
ATOM    306  H   GLU A 188      15.167  -5.886  13.826  1.00  0.19           H
ATOM    307  N   GLN A 189      15.639  -7.770  11.074  1.00 -0.26           N
ATOM    308  CA  GLN A 189      15.771  -8.220   9.688  1.00  0.13           C
ATOM    309  C   GLN A 189      16.984  -7.616   9.014  1.00  0.20           C
ATOM    310  O   GLN A 189      17.657  -8.314   8.271  1.00 -0.39           O
ATOM    311  CB  GLN A 189      14.509  -7.835   8.871  1.00  0.00           C
ATOM    312  CG  GLN A 189      13.332  -8.791   9.204  1.00  0.04           C
ATOM    313  CD  GLN A 189      13.433 -10.078   8.412  1.00  0.17           C
ATOM    314  OE1 GLN A 189      13.580 -10.007   7.199  1.00 -0.40           O
ATOM    315  NE2 GLN A 189      13.342 -11.272   9.027  1.00 -0.30           N
ATOM    316  HA  GLN A 189      15.900  -9.315   9.667  1.00  0.08           H
ATOM    317  HB2 GLN A 189      14.243  -6.789   9.095  1.00  0.03           H
ATOM    318  HB3 GLN A 189      14.720  -7.877   7.792  1.00  0.03           H
ATOM    319  HG2 GLN A 189      13.284  -8.982  10.286  1.00  0.05           H
ATOM    320  HG3 GLN A 189      12.388  -8.310   8.918  1.00  0.05           H
ATOM    321 HE22 GLN A 189      13.346 -12.116   8.480  1.00  0.18           H
ATOM    322 HE21 GLN A 189      13.222 -11.359  10.012  1.00  0.18           H
ATOM    323  H   GLN A 189      14.834  -7.235  11.359  1.00  0.19           H
ATOM    324  N   ALA A 190      17.287  -6.320   9.223  1.00 -0.26           N
ATOM    325  CA  ALA A 190      18.442  -5.746   8.534  1.00  0.13           C
ATOM    326  C   ALA A 190      19.762  -6.213   9.103  1.00  0.20           C
ATOM    327  O   ALA A 190      20.681  -6.490   8.345  1.00 -0.39           O
ATOM    328  CB  ALA A 190      18.427  -4.208   8.652  1.00 -0.02           C
ATOM    329  HA  ALA A 190      18.411  -6.015   7.468  1.00  0.08           H
ATOM    330  HB1 ALA A 190      19.282  -3.776   8.115  1.00  0.03           H
ATOM    331  HB2 ALA A 190      17.502  -3.822   8.211  1.00  0.03           H
ATOM    332  HB3 ALA A 190      18.480  -3.916   9.712  1.00  0.03           H
ATOM    333  H   ALA A 190      16.737  -5.751   9.846  1.00  0.19           H
ATOM    334  N   THR A 191      19.895  -6.275  10.443  1.00 -0.26           N
ATOM    335  CA  THR A 191      21.185  -6.648  11.022  1.00  0.16           C
ATOM    336  C   THR A 191      21.321  -8.147  11.156  1.00  0.21           C
ATOM    337  O   THR A 191      22.446  -8.622  11.194  1.00 -0.39           O
ATOM    338  CB  THR A 191      21.319  -6.016  12.431  1.00  0.09           C
ATOM    339  OG1 THR A 191      20.214  -6.498  13.223  1.00 -0.39           O
ATOM    340  CG2 THR A 191      21.306  -4.466  12.333  1.00 -0.03           C
ATOM    341  HA  THR A 191      22.020  -6.283  10.403  1.00  0.08           H
```

```
ATOM    342  HB  THR A 191      22.275  -6.328  12.889  1.00  0.06           H
ATOM    343  HG1 THR A 191      20.178  -6.107  14.090  1.00  0.21           H
ATOM    344 HG23 THR A 191      22.160  -4.118  11.731  1.00  0.03           H
ATOM    345 HG21 THR A 191      20.374  -4.112  11.866  1.00  0.03           H
ATOM    346 HG22 THR A 191      21.383  -4.018  13.337  1.00  0.03           H
ATOM    347  H   THR A 191      19.120  -6.085  11.061  1.00  0.19           H
ATOM    348  N   GLY A 192      20.219  -8.923  11.263  1.00 -0.27           N
ATOM    349  CA  GLY A 192      20.369 -10.333  11.607  1.00  0.12           C
ATOM    350  C   GLY A 192      20.687 -10.504  13.076  1.00  0.20           C
ATOM    351  O   GLY A 192      21.010 -11.619  13.455  1.00 -0.39           O
ATOM    352  HA3 GLY A 192      19.442 -10.889  11.397  1.00  0.08           H
ATOM    353  HA2 GLY A 192      21.178 -10.792  11.015  1.00  0.08           H
ATOM    354  H   GLY A 192      19.289  -8.547  11.216  1.00  0.19           H
ATOM    355  N   ALA A 193      20.596  -9.452  13.924  1.00 -0.26           N
ATOM    356  CA  ALA A 193      20.896  -9.598  15.346  1.00  0.13           C
ATOM    357  C   ALA A 193      19.637  -9.307  16.138  1.00  0.20           C
ATOM    358  O   ALA A 193      18.913  -8.395  15.772  1.00 -0.39           O
ATOM    359  CB  ALA A 193      22.022  -8.618  15.757  1.00 -0.02           C
ATOM    360  HA  ALA A 193      21.246 -10.615  15.584  1.00  0.08           H
ATOM    361  HB1 ALA A 193      22.263  -8.734  16.825  1.00  0.03           H
ATOM    362  HB2 ALA A 193      22.926  -8.817  15.164  1.00  0.03           H
ATOM    363  HB3 ALA A 193      21.705  -7.581  15.579  1.00  0.03           H
ATOM    364  H   ALA A 193      20.290  -8.548  13.620  1.00  0.19           H
ATOM    365  N   LYS A 194      19.335 -10.059  17.220  1.00 -0.26           N
ATOM    366  CA  LYS A 194      18.060  -9.862  17.914  1.00  0.13           C
ATOM    367  C   LYS A 194      18.214  -8.835  19.018  1.00  0.20           C
ATOM    368  O   LYS A 194      19.155  -8.940  19.789  1.00 -0.39           O
ATOM    369  CB  LYS A 194      17.585 -11.224  18.488  1.00 -0.01           C
ATOM    370  CG  LYS A 194      16.198 -11.141  19.180  1.00 -0.04           C
ATOM    371  CD  LYS A 194      15.701 -12.522  19.709  1.00 -0.01           C
ATOM    372  CE  LYS A 194      15.633 -13.618  18.603  1.00 -0.04           C
ATOM    373  NZ  LYS A 194      14.936 -14.847  19.074  1.00  0.22           N
ATOM    374  HA  LYS A 194      17.288  -9.529  17.197  1.00  0.08           H
ATOM    375  HB2 LYS A 194      17.541 -11.910  17.628  1.00  0.03           H
ATOM    376  HB3 LYS A 194      18.327 -11.602  19.212  1.00  0.03           H
ATOM    377  HG2 LYS A 194      16.255 -10.438  20.029  1.00  0.03           H
ATOM    378  HG3 LYS A 194      15.461 -10.748  18.464  1.00  0.03           H
ATOM    379  HD2 LYS A 194      16.368 -12.860  20.524  1.00  0.03           H
ATOM    380  HD3 LYS A 194      14.689 -12.376  20.130  1.00  0.03           H
ATOM    381  HE2 LYS A 194      15.121 -13.203  17.712  1.00  0.08           H
ATOM    382  HE3 LYS A 194      16.659 -13.907  18.312  1.00  0.08           H
ATOM    383  HZ1 LYS A 194      13.900 -14.656  19.314  1.00  0.20           H
ATOM    384  HZ2 LYS A 194      14.942 -15.626  18.323  1.00  0.20           H
ATOM    385  HZ3 LYS A 194      15.400 -15.262  19.959  1.00  0.20           H
ATOM    386  H   LYS A 194      19.971 -10.748  17.572  1.00  0.19           H
ATOM    387  N   ASP A 195      17.312  -7.832  19.142  1.00 -0.26           N
ATOM    388  CA  ASP A 195      17.490  -6.811  20.177  1.00  0.14           C
ATOM    389  C   ASP A 195      16.881  -7.341  21.460  1.00  0.21           C
ATOM    390  O   ASP A 195      15.684  -7.214  21.670  1.00 -0.39           O
ATOM    391  CB  ASP A 195      16.862  -5.470  19.720  1.00  0.04           C
ATOM    392  CG  ASP A 195      17.279  -4.274  20.538  1.00  0.04           C
```

ATOM	393	OD1	ASP	A	195	18.205	-4.356	21.392	1.00	-0.57	O
ATOM	394	OD2	ASP	A	195	16.675	-3.197	20.293	1.00	-0.57	O
ATOM	395	HA	ASP	A	195	18.563	-6.605	20.322	1.00	0.08	H
ATOM	396	HB2	ASP	A	195	17.195	-5.267	18.690	1.00	0.05	H
ATOM	397	HB3	ASP	A	195	15.765	-5.555	19.707	1.00	0.05	H
ATOM	398	H	ASP	A	195	16.505	-7.779	18.550	1.00	0.19	H
ATOM	399	N	THR	A	196	17.696	-7.946	22.353	1.00	-0.26	N
ATOM	400	CA	THR	A	196	17.175	-8.477	23.612	1.00	0.16	C
ATOM	401	C	THR	A	196	17.020	-7.409	24.677	1.00	0.21	C
ATOM	402	O	THR	A	196	16.784	-7.765	25.821	1.00	-0.39	O
ATOM	403	CB	THR	A	196	18.149	-9.578	24.118	1.00	0.09	C
ATOM	404	OG1	THR	A	196	19.463	-8.988	24.106	1.00	-0.39	O
ATOM	405	CG2	THR	A	196	18.120	-10.815	23.183	1.00	-0.03	C
ATOM	406	HA	THR	A	196	16.178	-8.923	23.470	1.00	0.08	H
ATOM	407	HB	THR	A	196	17.878	-9.906	25.140	1.00	0.06	H
ATOM	408	HG1	THR	A	196	20.137	-9.573	24.437	1.00	0.21	H
ATOM	409	HG23	THR	A	196	17.121	-11.278	23.198	1.00	0.03	H
ATOM	410	HG21	THR	A	196	18.364	-10.530	22.149	1.00	0.03	H
ATOM	411	HG22	THR	A	196	18.856	-11.560	23.523	1.00	0.03	H
ATOM	412	H	THR	A	196	18.681	-8.026	22.195	1.00	0.19	H
ATOM	413	N	LYS	A	197	17.120	-6.095	24.370	1.00	-0.26	N
ATOM	414	CA	LYS	A	197	16.895	-5.102	25.420	1.00	0.13	C
ATOM	415	C	LYS	A	197	15.421	-5.128	25.750	1.00	0.21	C
ATOM	416	O	LYS	A	197	14.656	-5.436	24.842	1.00	-0.39	O
ATOM	417	CB	LYS	A	197	17.382	-3.702	24.958	1.00	-0.01	C
ATOM	418	CG	LYS	A	197	18.925	-3.721	24.775	1.00	-0.04	C
ATOM	419	CD	LYS	A	197	19.459	-2.346	24.263	1.00	-0.01	C
ATOM	420	CE	LYS	A	197	20.569	-2.510	23.184	1.00	-0.04	C
ATOM	421	NZ	LYS	A	197	20.015	-3.007	21.883	1.00	0.22	N
ATOM	422	HA	LYS	A	197	17.460	-5.397	26.320	1.00	0.08	H
ATOM	423	HB2	LYS	A	197	16.887	-3.457	24.003	1.00	0.03	H
ATOM	424	HB3	LYS	A	197	17.123	-2.913	25.678	1.00	0.03	H
ATOM	425	HG2	LYS	A	197	19.416	-3.974	25.732	1.00	0.03	H
ATOM	426	HG3	LYS	A	197	19.181	-4.531	24.075	1.00	0.03	H
ATOM	427	HD2	LYS	A	197	18.646	-1.736	23.827	1.00	0.03	H
ATOM	428	HD3	LYS	A	197	19.856	-1.778	25.130	1.00	0.03	H
ATOM	429	HE2	LYS	A	197	21.047	-1.525	23.032	1.00	0.08	H
ATOM	430	HE3	LYS	A	197	21.341	-3.204	23.563	1.00	0.08	H
ATOM	431	HZ1	LYS	A	197	19.131	-2.496	21.527	1.00	0.20	H
ATOM	432	HZ2	LYS	A	197	20.750	-2.992	21.095	1.00	0.20	H
ATOM	433	HZ3	LYS	A	197	19.655	-4.024	21.904	1.00	0.20	H
ATOM	434	H	LYS	A	197	17.262	-5.781	23.432	1.00	0.19	H
ATOM	435	N	PRO	A	198	14.951	-4.843	26.992	1.00	-0.25	N
ATOM	436	CA	PRO	A	198	13.573	-5.167	27.317	1.00	0.13	C
ATOM	437	C	PRO	A	198	12.564	-4.455	26.450	1.00	0.20	C
ATOM	438	O	PRO	A	198	12.899	-3.564	25.683	1.00	-0.39	O
ATOM	439	CB	PRO	A	198	13.512	-4.756	28.811	1.00	-0.01	C
ATOM	440	CG	PRO	A	198	14.640	-3.711	28.941	1.00	-0.03	C
ATOM	441	CD	PRO	A	198	15.773	-4.297	28.067	1.00	0.04	C
ATOM	442	HA	PRO	A	198	13.413	-6.249	27.203	1.00	0.08	H
ATOM	443	HD3	PRO	A	198	16.304	-5.104	28.599	1.00	0.05	H

ATOM	444	HD2	PRO	A	198	16.481	-3.505	27.786	1.00	0.05	H
ATOM	445	HG3	PRO	A	198	14.946	-3.534	29.983	1.00	0.03	H
ATOM	446	HG2	PRO	A	198	14.290	-2.762	28.504	1.00	0.03	H
ATOM	447	HB2	PRO	A	198	12.529	-4.366	29.117	1.00	0.03	H
ATOM	448	HB3	PRO	A	198	13.766	-5.618	29.449	1.00	0.03	H
ATOM	449	N	MET	A	199	11.286	-4.868	26.566	1.00	-0.26	N
ATOM	450	CA	MET	A	199	10.235	-4.256	25.757	1.00	0.13	C
ATOM	451	C	MET	A	199	9.604	-3.155	26.574	1.00	0.20	C
ATOM	452	O	MET	A	199	9.882	-3.073	27.761	1.00	-0.39	O
ATOM	453	CB	MET	A	199	9.132	-5.302	25.442	1.00	-0.00	C
ATOM	454	CG	MET	A	199	9.679	-6.573	24.737	1.00	-0.00	C
ATOM	455	SD	MET	A	199	10.163	-6.195	23.019	1.00	-0.16	S
ATOM	456	CE	MET	A	199	10.261	-7.899	22.393	1.00	-0.02	C
ATOM	457	HA	MET	A	199	10.612	-3.848	24.806	1.00	0.08	H
ATOM	458	HB2	MET	A	199	8.690	-5.606	26.406	1.00	0.03	H
ATOM	459	HB3	MET	A	199	8.341	-4.850	24.816	1.00	0.03	H
ATOM	460	HG2	MET	A	199	10.556	-6.973	25.268	1.00	0.04	H
ATOM	461	HG3	MET	A	199	8.890	-7.344	24.726	1.00	0.04	H
ATOM	462	LPD1	MET	A	199	9.691	-5.829	22.694	1.00	0.00	LP
ATOM	463	LPD2	MET	A	199	10.760	-5.874	22.985	1.00	0.00	LP
ATOM	464	HE1	MET	A	199	9.255	-8.344	22.408	1.00	0.03	H
ATOM	465	HE2	MET	A	199	10.643	-7.891	21.365	1.00	0.03	H
ATOM	466	HE3	MET	A	199	10.939	-8.494	23.021	1.00	0.03	H
ATOM	467	H	MET	A	199	11.017	-5.531	27.265	1.00	0.19	H
ATOM	468	N	GLY	A	200	8.730	-2.317	25.978	1.00	-0.27	N
ATOM	469	CA	GLY	A	200	7.936	-1.432	26.819	1.00	0.12	C
ATOM	470	C	GLY	A	200	6.800	-2.203	27.458	1.00	0.20	C
ATOM	471	O	GLY	A	200	6.801	-3.425	27.427	1.00	-0.39	O
ATOM	472	HA3	GLY	A	200	8.550	-0.988	27.621	1.00	0.08	H
ATOM	473	HA2	GLY	A	200	7.530	-0.606	26.218	1.00	0.08	H
ATOM	474	H	GLY	A	200	8.517	-2.347	24.993	1.00	0.19	H
ATOM	475	N	ARG	A	201	5.834	-1.464	28.048	1.00	-0.29	N
ATOM	476	CA	ARG	A	201	4.733	-2.072	28.797	1.00	0.01	C
ATOM	477	C	ARG	A	201	4.252	-3.416	28.277	1.00	0.17	C
ATOM	478	O	ARG	A	201	4.357	-4.435	28.945	1.00	-0.40	O
ATOM	479	CB	ARG	A	201	3.546	-1.071	28.747	1.00	-0.30	C
ATOM	480	HA	ARG	A	201	5.043	-2.190	29.848	1.00	0.04	H
ATOM	481	HB2	ARG	A	201	2.661	-1.457	29.275	1.00	-0.21	H
ATOM	482	HB3	ARG	A	201	3.809	-0.082	29.155	1.00	-0.21	H
ATOM	483	H	ARG	A	201	5.885	-0.464	28.052	1.00	0.19	H
ATOM	484	N	SER	A	202	3.694	-3.449	27.052	1.00	-0.26	N
ATOM	485	CA	SER	A	202	3.025	-4.657	26.561	1.00	0.15	C
ATOM	486	C	SER	A	202	3.957	-5.701	25.974	1.00	0.21	C
ATOM	487	O	SER	A	202	3.664	-6.280	24.937	1.00	-0.39	O
ATOM	488	CB	SER	A	202	1.961	-4.180	25.539	1.00	0.08	C
ATOM	489	OG	SER	A	202	2.620	-3.337	24.575	1.00	-0.39	O
ATOM	490	HA	SER	A	202	2.502	-5.163	27.390	1.00	0.08	H
ATOM	491	HB2	SER	A	202	1.461	-5.038	25.062	1.00	0.06	H
ATOM	492	HB3	SER	A	202	1.200	-3.585	26.075	1.00	0.06	H
ATOM	493	HG	SER	A	202	2.009	-3.005	23.924	1.00	0.21	H
ATOM	494	H	SER	A	202	3.693	-2.619	26.493	1.00	0.19	H

ATOM	495	N	GLY	A	203	5.097	-5.994	26.637	1.00	-0.27	N
ATOM	496	CA	GLY	A	203	6.063	-6.914	26.044	1.00	0.12	C
ATOM	497	C	GLY	A	203	5.499	-8.236	25.585	1.00	0.20	C
ATOM	498	O	GLY	A	203	5.922	-8.727	24.550	1.00	-0.39	O
ATOM	499	HA3	GLY	A	203	6.505	-6.412	25.173	1.00	0.08	H
ATOM	500	HA2	GLY	A	203	6.861	-7.157	26.762	1.00	0.08	H
ATOM	501	H	GLY	A	203	5.331	-5.523	27.497	1.00	0.19	H
ATOM	502	N	ALA	A	204	4.567	-8.864	26.331	1.00	-0.26	N
ATOM	503	CA	ALA	A	204	4.037	-10.150	25.870	1.00	0.13	C
ATOM	504	C	ALA	A	204	3.447	-10.009	24.483	1.00	0.20	C
ATOM	505	O	ALA	A	204	3.746	-10.809	23.608	1.00	-0.39	O
ATOM	506	CB	ALA	A	204	2.943	-10.689	26.830	1.00	-0.02	C
ATOM	507	HA	ALA	A	204	4.858	-10.883	25.822	1.00	0.08	H
ATOM	508	HB1	ALA	A	204	2.550	-11.650	26.463	1.00	0.03	H
ATOM	509	HB2	ALA	A	204	3.365	-10.843	27.835	1.00	0.03	H
ATOM	510	HB3	ALA	A	204	2.109	-9.974	26.905	1.00	0.03	H
ATOM	511	H	ALA	A	204	4.230	-8.449	27.180	1.00	0.19	H
ATOM	512	N	THR	A	205	2.593	-8.988	24.266	1.00	-0.26	N
ATOM	513	CA	THR	A	205	1.965	-8.835	22.957	1.00	0.16	C
ATOM	514	C	THR	A	205	3.020	-8.526	21.922	1.00	0.21	C
ATOM	515	O	THR	A	205	3.067	-9.187	20.894	1.00	-0.39	O
ATOM	516	CB	THR	A	205	0.841	-7.768	23.012	1.00	0.09	C
ATOM	517	OG1	THR	A	205	-0.096	-8.218	24.010	1.00	-0.39	O
ATOM	518	CG2	THR	A	205	0.145	-7.598	21.639	1.00	-0.03	C
ATOM	519	HA	THR	A	205	1.481	-9.781	22.679	1.00	0.08	H
ATOM	520	HB	THR	A	205	1.271	-6.797	23.309	1.00	0.06	H
ATOM	521	HG1	THR	A	205	-0.904	-7.708	24.032	1.00	0.21	H
ATOM	522	HG23	THR	A	205	0.882	-7.287	20.886	1.00	0.03	H
ATOM	523	HG21	THR	A	205	-0.323	-8.542	21.323	1.00	0.03	H
ATOM	524	HG22	THR	A	205	-0.636	-6.826	21.713	1.00	0.03	H
ATOM	525	H	THR	A	205	2.399	-8.308	24.977	1.00	0.19	H
ATOM	526	N	SER	A	206	3.892	-7.526	22.173	1.00	-0.26	N
ATOM	527	CA	SER	A	206	4.918	-7.219	21.179	1.00	0.15	C
ATOM	528	C	SER	A	206	5.773	-8.430	20.861	1.00	0.21	C
ATOM	529	O	SER	A	206	6.117	-8.658	19.709	1.00	-0.39	O
ATOM	530	CB	SER	A	206	5.847	-6.078	21.663	1.00	0.08	C
ATOM	531	OG	SER	A	206	5.064	-4.893	21.883	1.00	-0.39	O
ATOM	532	HA	SER	A	206	4.407	-6.895	20.264	1.00	0.08	H
ATOM	533	HB2	SER	A	206	6.340	-6.386	22.600	1.00	0.06	H
ATOM	534	HB3	SER	A	206	6.623	-5.873	20.906	1.00	0.06	H
ATOM	535	HG	SER	A	206	5.605	-4.182	22.237	1.00	0.21	H
ATOM	536	H	SER	A	206	3.846	-6.979	23.018	1.00	0.19	H
ATOM	537	N	ARG	A	207	6.148	-9.226	21.885	1.00	-0.26	N
ATOM	538	CA	ARG	A	207	7.034	-10.363	21.635	1.00	0.13	C
ATOM	539	C	ARG	A	207	6.332	-11.394	20.772	1.00	0.20	C
ATOM	540	O	ARG	A	207	6.939	-11.931	19.855	1.00	-0.39	O
ATOM	541	CB	ARG	A	207	7.486	-10.978	22.988	1.00	-0.01	C
ATOM	542	CG	ARG	A	207	8.820	-11.767	22.922	1.00	-0.02	C
ATOM	543	CD	ARG	A	207	9.120	-12.436	24.298	1.00	0.06	C
ATOM	544	NE	ARG	A	207	8.679	-11.603	25.433	1.00	-0.27	N
ATOM	545	CZ	ARG	A	207	9.344	-10.581	25.929	1.00	0.29	C

```
ATOM    546  NH1 ARG A 207      10.502 -10.186  25.463  1.00 -0.28           N
ATOM    547  NH2 ARG A 207       8.823  -9.922  26.938  1.00 -0.28           N
ATOM    548  HA  ARG A 207       7.919 -10.003  21.091  1.00  0.08           H
ATOM    549  HB2 ARG A 207       7.678 -10.153  23.689  1.00  0.03           H
ATOM    550  HB3 ARG A 207       6.674 -11.603  23.396  1.00  0.03           H
ATOM    551  HG2 ARG A 207       8.769 -12.532  22.130  1.00  0.03           H
ATOM    552  HG3 ARG A 207       9.630 -11.069  22.659  1.00  0.03           H
ATOM    553  HD2 ARG A 207       8.527 -13.369  24.323  1.00  0.07           H
ATOM    554  HD3 ARG A 207      10.169 -12.763  24.377  1.00  0.07           H
ATOM    555  HE  ARG A 207       7.779 -11.864  25.869  1.00  0.26           H
ATOM    556 HH12 ARG A 207      11.003  -9.392  25.883  1.00  0.26           H
ATOM    557 HH11 ARG A 207      10.952 -10.658  24.675  1.00  0.26           H
ATOM    558 HH22 ARG A 207       9.308  -9.118  27.360  1.00  0.26           H
ATOM    559 HH21 ARG A 207       7.914 -10.188  27.341  1.00  0.26           H
ATOM    560  H   ARG A 207       5.814  -9.043  22.818  1.00  0.19           H
ATOM    561  N   LYS A 208       5.042 -11.684  21.054  1.00 -0.26           N
ATOM    562  CA  LYS A 208       4.312 -12.622  20.200  1.00  0.13           C
ATOM    563  C   LYS A 208       4.165 -12.028  18.810  1.00  0.20           C
ATOM    564  O   LYS A 208       4.403 -12.722  17.832  1.00 -0.39           O
ATOM    565  CB  LYS A 208       2.908 -12.944  20.788  1.00 -0.01           C
ATOM    566  CG  LYS A 208       2.986 -13.770  22.106  1.00 -0.04           C
ATOM    567  CD  LYS A 208       1.596 -14.023  22.777  1.00 -0.01           C
ATOM    568  CE  LYS A 208       0.899 -12.721  23.276  1.00 -0.04           C
ATOM    569  NZ  LYS A 208      -0.325 -12.971  24.089  1.00  0.22           N
ATOM    570  HA  LYS A 208       4.881 -13.561  20.109  1.00  0.08           H
ATOM    571  HB2 LYS A 208       2.400 -11.986  20.966  1.00  0.03           H
ATOM    572  HB3 LYS A 208       2.333 -13.520  20.044  1.00  0.03           H
ATOM    573  HG2 LYS A 208       3.434 -14.751  21.868  1.00  0.03           H
ATOM    574  HG3 LYS A 208       3.646 -13.271  22.833  1.00  0.03           H
ATOM    575  HD2 LYS A 208       0.936 -14.552  22.067  1.00  0.03           H
ATOM    576  HD3 LYS A 208       1.760 -14.686  23.648  1.00  0.03           H
ATOM    577  HE2 LYS A 208       1.625 -12.148  23.880  1.00  0.08           H
ATOM    578  HE3 LYS A 208       0.603 -12.108  22.406  1.00  0.08           H
ATOM    579  HZ1 LYS A 208      -0.101 -13.521  24.994  1.00  0.20           H
ATOM    580  HZ2 LYS A 208      -0.815 -12.048  24.381  1.00  0.20           H
ATOM    581  HZ3 LYS A 208      -1.068 -13.538  23.546  1.00  0.20           H
ATOM    582  H   LYS A 208       4.561 -11.219  21.808  1.00  0.19           H
ATOM    583  N   ALA A 209       3.772 -10.741  18.678  1.00 -0.26           N
ATOM    584  CA  ALA A 209       3.668 -10.166  17.336  1.00  0.13           C
ATOM    585  C   ALA A 209       4.975 -10.321  16.584  1.00  0.20           C
ATOM    586  O   ALA A 209       4.975 -10.699  15.421  1.00 -0.39           O
ATOM    587  CB  ALA A 209       3.274  -8.672  17.399  1.00 -0.02           C
ATOM    588  HA  ALA A 209       2.894 -10.710  16.777  1.00  0.08           H
ATOM    589  HB1 ALA A 209       3.192  -8.248  16.387  1.00  0.03           H
ATOM    590  HB2 ALA A 209       2.307  -8.559  17.910  1.00  0.03           H
ATOM    591  HB3 ALA A 209       4.036  -8.112  17.957  1.00  0.03           H
ATOM    592  H   ALA A 209       3.569 -10.174  19.486  1.00  0.19           H
ATOM    593  N   LEU A 210       6.126 -10.032  17.225  1.00 -0.26           N
ATOM    594  CA  LEU A 210       7.390 -10.245  16.524  1.00  0.13           C
ATOM    595  C   LEU A 210       7.545 -11.687  16.091  1.00  0.20           C
ATOM    596  O   LEU A 210       7.919 -11.922  14.952  1.00 -0.39           O
```

ATOM	597	CB	LEU	A	210	8.604	-9.842	17.405	1.00	-0.01	C
ATOM	598	CG	LEU	A	210	8.789	-8.298	17.425	1.00	-0.04	C
ATOM	599	CD1	LEU	A	210	9.479	-7.831	18.733	1.00	-0.06	C
ATOM	600	CD2	LEU	A	210	9.613	-7.843	16.187	1.00	-0.06	C
ATOM	601	HA	LEU	A	210	7.372	-9.643	15.604	1.00	0.08	H
ATOM	602	HB2	LEU	A	210	8.443	-10.231	18.421	1.00	0.03	H
ATOM	603	HB3	LEU	A	210	9.525	-10.306	17.016	1.00	0.03	H
ATOM	604	HG	LEU	A	210	7.798	-7.815	17.382	1.00	0.03	H
ATOM	605	HD21	LEU	A	210	9.184	-8.249	15.262	1.00	0.02	H
ATOM	606	HD22	LEU	A	210	9.608	-6.748	16.112	1.00	0.02	H
ATOM	607	HD23	LEU	A	210	10.659	-8.184	16.252	1.00	0.02	H
ATOM	608	HD11	LEU	A	210	10.477	-8.287	18.818	1.00	0.02	H
ATOM	609	HD12	LEU	A	210	9.581	-6.738	18.716	1.00	0.02	H
ATOM	610	HD13	LEU	A	210	8.887	-8.097	19.623	1.00	0.02	H
ATOM	611	H	LEU	A	210	6.118	-9.695	18.175	1.00	0.19	H
ATOM	612	N	GLU	A	211	7.274	-12.676	16.970	1.00	-0.26	N
ATOM	613	CA	GLU	A	211	7.364	-14.070	16.526	1.00	0.13	C
ATOM	614	C	GLU	A	211	6.538	-14.248	15.273	1.00	0.20	C
ATOM	615	O	GLU	A	211	7.055	-14.689	14.255	1.00	-0.39	O
ATOM	616	CB	GLU	A	211	6.990	-15.033	17.685	1.00	-0.00	C
ATOM	617	CG	GLU	A	211	6.989	-16.524	17.264	1.00	0.00	C
ATOM	618	CD	GLU	A	211	5.702	-16.902	16.585	1.00	0.04	C
ATOM	619	OE1	GLU	A	211	4.615	-16.755	17.201	1.00	-0.57	O
ATOM	620	OE2	GLU	A	211	5.741	-17.369	15.418	1.00	-0.57	O
ATOM	621	HA	GLU	A	211	8.394	-14.298	16.225	1.00	0.08	H
ATOM	622	HB2	GLU	A	211	7.738	-14.889	18.482	1.00	0.03	H
ATOM	623	HB3	GLU	A	211	6.001	-14.786	18.101	1.00	0.03	H
ATOM	624	HG2	GLU	A	211	7.857	-16.753	16.625	1.00	0.04	H
ATOM	625	HG3	GLU	A	211	7.044	-17.151	18.166	1.00	0.04	H
ATOM	626	H	GLU	A	211	6.953	-12.464	17.901	1.00	0.19	H
ATOM	627	N	THR	A	212	5.247	-13.872	15.316	1.00	-0.26	N
ATOM	628	CA	THR	A	212	4.437	-14.005	14.112	1.00	0.16	C
ATOM	629	C	THR	A	212	5.058	-13.324	12.909	1.00	0.21	C
ATOM	630	O	THR	A	212	5.079	-13.919	11.840	1.00	-0.39	O
ATOM	631	CB	THR	A	212	3.031	-13.430	14.398	1.00	0.09	C
ATOM	632	OG1	THR	A	212	2.479	-14.240	15.449	1.00	-0.39	O
ATOM	633	CG2	THR	A	212	2.112	-13.485	13.162	1.00	-0.03	C
ATOM	634	HA	THR	A	212	4.337	-15.081	13.897	1.00	0.08	H
ATOM	635	HB	THR	A	212	3.134	-12.380	14.710	1.00	0.06	H
ATOM	636	HG1	THR	A	212	1.636	-13.939	15.768	1.00	0.21	H
ATOM	637	HG23	THR	A	212	2.551	-12.947	12.315	1.00	0.03	H
ATOM	638	HG21	THR	A	212	1.966	-14.537	12.882	1.00	0.03	H
ATOM	639	HG22	THR	A	212	1.148	-13.016	13.412	1.00	0.03	H
ATOM	640	H	THR	A	212	4.834	-13.534	16.171	1.00	0.19	H
ATOM	641	N	LEU	A	213	5.570	-12.077	13.024	1.00	-0.26	N
ATOM	642	CA	LEU	A	213	6.200	-11.465	11.852	1.00	0.13	C
ATOM	643	C	LEU	A	213	7.354	-12.319	11.375	1.00	0.20	C
ATOM	644	O	LEU	A	213	7.477	-12.489	10.171	1.00	-0.39	O
ATOM	645	CB	LEU	A	213	6.749	-10.026	12.067	1.00	-0.01	C
ATOM	646	CG	LEU	A	213	5.650	-8.923	12.125	1.00	-0.04	C
ATOM	647	CD1	LEU	A	213	6.264	-7.603	12.668	1.00	-0.06	C

```
ATOM    648  CD2 LEU A 213       5.015  -8.634  10.730  1.00 -0.06           C
ATOM    649  HA  LEU A 213       5.461 -11.453  11.038  1.00  0.08           H
ATOM    650  HB2 LEU A 213       7.334 -10.037  12.999  1.00  0.03           H
ATOM    651  HB3 LEU A 213       7.437  -9.764  11.244  1.00  0.03           H
ATOM    652  HG  LEU A 213       4.863  -9.246  12.827  1.00  0.03           H
ATOM    653 HD21 LEU A 213       4.505  -9.513  10.315  1.00  0.02           H
ATOM    654 HD22 LEU A 213       4.266  -7.830  10.813  1.00  0.02           H
ATOM    655 HD23 LEU A 213       5.787  -8.313  10.012  1.00  0.02           H
ATOM    656 HD11 LEU A 213       7.074  -7.256  12.007  1.00  0.02           H
ATOM    657 HD12 LEU A 213       5.493  -6.817  12.720  1.00  0.02           H
ATOM    658 HD13 LEU A 213       6.673  -7.758  13.678  1.00  0.02           H
ATOM    659  H   LEU A 213       5.556 -11.585  13.901  1.00  0.19           H
ATOM    660  N   ARG A 214       8.207 -12.864  12.271  1.00 -0.26           N
ATOM    661  CA  ARG A 214       9.312 -13.702  11.795  1.00  0.13           C
ATOM    662  C   ARG A 214       8.778 -14.849  10.963  1.00  0.20           C
ATOM    663  O   ARG A 214       9.414 -15.246  10.000  1.00 -0.39           O
ATOM    664  CB  ARG A 214      10.180 -14.296  12.944  1.00 -0.01           C
ATOM    665  CG  ARG A 214      10.905 -13.221  13.801  1.00 -0.02           C
ATOM    666  CD  ARG A 214      11.864 -13.845  14.851  1.00  0.06           C
ATOM    667  NE  ARG A 214      12.440 -12.759  15.657  1.00 -0.27           N
ATOM    668  CZ  ARG A 214      11.979 -12.298  16.801  1.00  0.29           C
ATOM    669  NH1 ARG A 214      10.928 -12.781  17.411  1.00 -0.28           N
ATOM    670  NH2 ARG A 214      12.595 -11.295  17.377  1.00 -0.28           N
ATOM    671  HA  ARG A 214       9.956 -13.107  11.129  1.00  0.08           H
ATOM    672  HB2 ARG A 214       9.542 -14.918  13.591  1.00  0.03           H
ATOM    673  HB3 ARG A 214      10.944 -14.949  12.493  1.00  0.03           H
ATOM    674  HG2 ARG A 214      11.484 -12.554  13.144  1.00  0.03           H
ATOM    675  HG3 ARG A 214      10.179 -12.603  14.344  1.00  0.03           H
ATOM    676  HD2 ARG A 214      11.380 -14.659  15.408  1.00  0.07           H
ATOM    677  HD3 ARG A 214      12.684 -14.334  14.297  1.00  0.07           H
ATOM    678  HE  ARG A 214      13.303 -12.329  15.268  1.00  0.26           H
ATOM    679 HH12 ARG A 214      10.598 -12.394  18.305  1.00  0.26           H
ATOM    680 HH11 ARG A 214      10.397 -13.553  17.015  1.00  0.26           H
ATOM    681 HH22 ARG A 214      12.276 -10.896  18.264  1.00  0.26           H
ATOM    682 HH21 ARG A 214      13.417 -10.850  16.935  1.00  0.26           H
ATOM    683  H   ARG A 214       8.059 -12.733  13.258  1.00  0.19           H
ATOM    684  N   ARG A 215       7.611 -15.427  11.310  1.00 -0.26           N
ATOM    685  CA  ARG A 215       7.070 -16.503  10.479  1.00  0.13           C
ATOM    686  C   ARG A 215       6.482 -15.980   9.186  1.00  0.20           C
ATOM    687  O   ARG A 215       6.806 -16.502   8.129  1.00 -0.39           O
ATOM    688  CB  ARG A 215       6.002 -17.309  11.272  1.00 -0.01           C
ATOM    689  CG  ARG A 215       5.219 -18.332  10.404  1.00 -0.02           C
ATOM    690  CD  ARG A 215       4.273 -19.264  11.221  1.00  0.06           C
ATOM    691  NE  ARG A 215       3.157 -18.557  11.877  1.00 -0.27           N
ATOM    692  CZ  ARG A 215       3.170 -18.092  13.109  1.00  0.29           C
ATOM    693  NH1 ARG A 215       4.211 -18.160  13.895  1.00 -0.28           N
ATOM    694  NH2 ARG A 215       2.091 -17.529  13.596  1.00 -0.28           N
ATOM    695  HA  ARG A 215       7.882 -17.199  10.204  1.00  0.08           H
ATOM    696  HB2 ARG A 215       6.557 -17.832  12.065  1.00  0.03           H
ATOM    697  HB3 ARG A 215       5.270 -16.625  11.733  1.00  0.03           H
ATOM    698  HG2 ARG A 215       4.613 -17.788   9.661  1.00  0.03           H
```

ATOM	699	HG3	ARG	A	215	5.950	-18.956	9.861	1.00	0.03	H
ATOM	700	HD2	ARG	A	215	3.819	-19.948	10.481	1.00	0.07	H
ATOM	701	HD3	ARG	A	215	4.847	-19.927	11.885	1.00	0.07	H
ATOM	702	HE	ARG	A	215	2.285	-18.464	11.331	1.00	0.26	H
ATOM	703	HH12	ARG	A	215	4.222	-17.745	14.845	1.00	0.26	H
ATOM	704	HH11	ARG	A	215	5.091	-18.588	13.620	1.00	0.26	H
ATOM	705	HH22	ARG	A	215	2.068	-17.163	14.558	1.00	0.26	H
ATOM	706	HH21	ARG	A	215	1.211	-17.469	13.056	1.00	0.26	H
ATOM	707	H	ARG	A	215	7.098	-15.104	12.111	1.00	0.19	H
ATOM	708	N	VAL	A	216	5.580	-14.977	9.235	1.00	-0.26	N
ATOM	709	CA	VAL	A	216	4.850	-14.606	8.018	1.00	0.13	C
ATOM	710	C	VAL	A	216	5.602	-13.562	7.208	1.00	0.20	C
ATOM	711	O	VAL	A	216	5.614	-13.651	5.988	1.00	-0.39	O
ATOM	712	CB	VAL	A	216	3.407	-14.136	8.379	1.00	-0.01	C
ATOM	713	CG1	VAL	A	216	2.551	-13.901	7.099	1.00	-0.06	C
ATOM	714	CG2	VAL	A	216	2.677	-15.182	9.277	1.00	-0.06	C
ATOM	715	HA	VAL	A	216	4.725	-15.492	7.373	1.00	0.08	H
ATOM	716	HB	VAL	A	216	3.482	-13.185	8.937	1.00	0.03	H
ATOM	717	HG11	VAL	A	216	2.362	-14.856	6.586	1.00	0.02	H
ATOM	718	HG12	VAL	A	216	1.580	-13.452	7.369	1.00	0.02	H
ATOM	719	HG13	VAL	A	216	3.052	-13.231	6.386	1.00	0.02	H
ATOM	720	HG21	VAL	A	216	3.192	-15.345	10.237	1.00	0.02	H
ATOM	721	HG22	VAL	A	216	1.657	-14.832	9.504	1.00	0.02	H
ATOM	722	HG23	VAL	A	216	2.603	-16.151	8.758	1.00	0.02	H
ATOM	723	H	VAL	A	216	5.405	-14.490	10.101	1.00	0.19	H
ATOM	724	N	GLY	A	217	6.249	-12.556	7.839	1.00	-0.27	N
ATOM	725	CA	GLY	A	217	6.878	-11.495	7.052	1.00	0.12	C
ATOM	726	C	GLY	A	217	7.972	-12.042	6.166	1.00	0.20	C
ATOM	727	O	GLY	A	217	8.048	-11.713	4.989	1.00	-0.39	O
ATOM	728	HA3	GLY	A	217	6.112	-11.015	6.429	1.00	0.08	H
ATOM	729	HA2	GLY	A	217	7.322	-10.727	7.704	1.00	0.08	H
ATOM	730	H	GLY	A	217	6.329	-12.524	8.840	1.00	0.19	H
ATOM	731	N	ASP	A	218	8.851	-12.893	6.734	1.00	-0.26	N
ATOM	732	CA	ASP	A	218	9.887	-13.509	5.912	1.00	0.14	C
ATOM	733	C	ASP	A	218	9.264	-14.235	4.734	1.00	0.21	C
ATOM	734	O	ASP	A	218	9.748	-14.108	3.619	1.00	-0.39	O
ATOM	735	CB	ASP	A	218	10.757	-14.475	6.753	1.00	0.04	C
ATOM	736	CG	ASP	A	218	11.563	-13.689	7.751	1.00	0.04	C
ATOM	737	OD1	ASP	A	218	11.007	-12.727	8.343	1.00	-0.57	O
ATOM	738	OD2	ASP	A	218	12.771	-13.990	7.945	1.00	-0.57	O
ATOM	739	HA	ASP	A	218	10.548	-12.718	5.526	1.00	0.08	H
ATOM	740	HB2	ASP	A	218	10.124	-15.202	7.284	1.00	0.05	H
ATOM	741	HB3	ASP	A	218	11.445	-15.016	6.086	1.00	0.05	H
ATOM	742	H	ASP	A	218	8.829	-13.089	7.721	1.00	0.19	H
ATOM	743	N	GLY	A	219	8.174	-15.009	4.934	1.00	-0.27	N
ATOM	744	CA	GLY	A	219	7.563	-15.698	3.797	1.00	0.12	C
ATOM	745	C	GLY	A	219	7.102	-14.741	2.725	1.00	0.20	C
ATOM	746	O	GLY	A	219	7.243	-15.036	1.546	1.00	-0.39	O
ATOM	747	HA3	GLY	A	219	8.298	-16.380	3.351	1.00	0.08	H
ATOM	748	HA2	GLY	A	219	6.688	-16.288	4.105	1.00	0.08	H
ATOM	749	H	GLY	A	219	7.758	-15.093	5.843	1.00	0.19	H

ATOM 750 N VAL A 220 6.526 -13.580 3.095 1.00 -0.26 N
ATOM 751 CA VAL A 220 6.151 -12.625 2.056 1.00 0.13 C
ATOM 752 C VAL A 220 7.410 -12.142 1.369 1.00 0.20 C
ATOM 753 O VAL A 220 7.435 -12.103 0.147 1.00 -0.39 O
ATOM 754 CB VAL A 220 5.387 -11.402 2.624 1.00 -0.01 C
ATOM 755 CG1 VAL A 220 5.232 -10.263 1.580 1.00 -0.06 C
ATOM 756 CG2 VAL A 220 3.988 -11.817 3.154 1.00 -0.06 C
ATOM 757 HA VAL A 220 5.512 -13.126 1.315 1.00 0.08 H
ATOM 758 HB VAL A 220 5.997 -11.019 3.455 1.00 0.03 H
ATOM 759 HG11 VAL A 220 4.570 -10.568 0.759 1.00 0.02 H
ATOM 760 HG12 VAL A 220 4.774 -9.398 2.067 1.00 0.02 H
ATOM 761 HG13 VAL A 220 6.197 -9.932 1.169 1.00 0.02 H
ATOM 762 HG21 VAL A 220 4.073 -12.574 3.947 1.00 0.02 H
ATOM 763 HG22 VAL A 220 3.462 -10.942 3.565 1.00 0.02 H
ATOM 764 HG23 VAL A 220 3.378 -12.231 2.336 1.00 0.02 H
ATOM 765 H VAL A 220 6.401 -13.352 4.068 1.00 0.19 H
ATOM 766 N GLN A 221 8.468 -11.760 2.119 1.00 -0.26 N
ATOM 767 CA GLN A 221 9.681 -11.315 1.438 1.00 0.13 C
ATOM 768 C GLN A 221 10.064 -12.393 0.445 1.00 0.20 C
ATOM 769 O GLN A 221 10.217 -12.090 -0.729 1.00 -0.39 O
ATOM 770 CB GLN A 221 10.874 -11.047 2.398 1.00 0.00 C
ATOM 771 CG GLN A 221 10.671 -9.767 3.248 1.00 0.04 C
ATOM 772 CD GLN A 221 11.644 -9.726 4.401 1.00 0.17 C
ATOM 773 OE1 GLN A 221 12.820 -9.483 4.177 1.00 -0.40 O
ATOM 774 NE2 GLN A 221 11.213 -9.965 5.657 1.00 -0.30 N
ATOM 775 HA GLN A 221 9.464 -10.395 0.874 1.00 0.08 H
ATOM 776 HB2 GLN A 221 11.003 -11.913 3.066 1.00 0.03 H
ATOM 777 HB3 GLN A 221 11.803 -10.930 1.813 1.00 0.03 H
ATOM 778 HG2 GLN A 221 10.801 -8.869 2.625 1.00 0.05 H
ATOM 779 HG3 GLN A 221 9.652 -9.753 3.645 1.00 0.05 H
ATOM 780 HE22 GLN A 221 11.867 -9.960 6.414 1.00 0.18 H
ATOM 781 HE21 GLN A 221 10.258 -10.164 5.877 1.00 0.18 H
ATOM 782 H GLN A 221 8.439 -11.810 3.122 1.00 0.19 H
ATOM 783 N ARG A 222 10.192 -13.659 0.902 1.00 -0.26 N
ATOM 784 CA ARG A 222 10.604 -14.725 -0.008 1.00 0.13 C
ATOM 785 C ARG A 222 9.676 -14.766 -1.209 1.00 0.20 C
ATOM 786 O ARG A 222 10.147 -14.874 -2.330 1.00 -0.39 O
ATOM 787 CB ARG A 222 10.572 -16.127 0.669 1.00 -0.01 C
ATOM 788 CG ARG A 222 11.586 -16.290 1.841 1.00 -0.02 C
ATOM 789 CD ARG A 222 11.453 -17.654 2.576 1.00 0.06 C
ATOM 790 NE ARG A 222 12.251 -17.592 3.813 1.00 -0.27 N
ATOM 791 CZ ARG A 222 11.798 -17.587 5.054 1.00 0.29 C
ATOM 792 NH1 ARG A 222 10.536 -17.668 5.401 1.00 -0.28 N
ATOM 793 NH2 ARG A 222 12.672 -17.489 6.030 1.00 -0.28 N
ATOM 794 HA ARG A 222 11.629 -14.515 -0.355 1.00 0.08 H
ATOM 795 HB2 ARG A 222 9.548 -16.307 1.034 1.00 0.03 H
ATOM 796 HB3 ARG A 222 10.799 -16.894 -0.089 1.00 0.03 H
ATOM 797 HG2 ARG A 222 12.611 -16.175 1.457 1.00 0.03 H
ATOM 798 HG3 ARG A 222 11.436 -15.511 2.598 1.00 0.03 H
ATOM 799 HD2 ARG A 222 10.392 -17.909 2.666 1.00 0.07 H
ATOM 800 HD3 ARG A 222 11.878 -18.433 1.921 1.00 0.07 H

```
ATOM    801  HE  ARG A 222      13.271 -17.539   3.664  1.00  0.26           H
ATOM    802 HH12 ARG A 222      10.249 -17.663   6.390  1.00  0.26           H
ATOM    803 HH11 ARG A 222       9.789 -17.744   4.712  1.00  0.26           H
ATOM    804 HH22 ARG A 222      12.381 -17.462   7.017  1.00  0.26           H
ATOM    805 HH21 ARG A 222      13.683 -17.412   5.844  1.00  0.26           H
ATOM    806  H   ARG A 222       9.981 -13.866   1.860  1.00  0.19           H
ATOM    807  N   ASN A 223       8.340 -14.699  -1.026  1.00 -0.26           N
ATOM    808  CA  ASN A 223       7.452 -14.805  -2.185  1.00  0.15           C
ATOM    809  C   ASN A 223       7.296 -13.524  -2.987  1.00  0.21           C
ATOM    810  O   ASN A 223       6.628 -13.585  -4.007  1.00 -0.39           O
ATOM    811  CB  ASN A 223       6.036 -15.277  -1.774  1.00  0.08           C
ATOM    812  CG  ASN A 223       6.085 -16.656  -1.175  1.00  0.18           C
ATOM    813  OD1 ASN A 223       6.315 -17.603  -1.916  1.00 -0.40           O
ATOM    814  ND2 ASN A 223       5.878 -16.843   0.140  1.00 -0.30           N
ATOM    815  HA  ASN A 223       7.843 -15.561  -2.888  1.00  0.08           H
ATOM    816  HB2 ASN A 223       5.615 -14.549  -1.068  1.00  0.06           H
ATOM    817  HB3 ASN A 223       5.370 -15.329  -2.652  1.00  0.06           H
ATOM    818 HD22 ASN A 223       5.892 -17.777   0.500  1.00  0.18           H
ATOM    819 HD21 ASN A 223       5.730 -16.074   0.767  1.00  0.18           H
ATOM    820  H   ASN A 223       7.952 -14.577  -0.107  1.00  0.19           H
ATOM    821  N   HIS A 224       7.846 -12.347  -2.612  1.00 -0.26           N
ATOM    822  CA  HIS A 224       7.705 -11.169  -3.480  1.00  0.16           C
ATOM    823  C   HIS A 224       9.019 -10.441  -3.664  1.00  0.21           C
ATOM    824  O   HIS A 224       9.008  -9.226  -3.790  1.00 -0.39           O
ATOM    825  CB  HIS A 224       6.604 -10.228  -2.933  1.00  0.11           C
ATOM    826  CG  HIS A 224       5.267 -10.925  -2.872  1.00  0.20           C
ATOM    827  ND1 HIS A 224       4.311 -10.851  -3.781  1.00 -0.16           N
ATOM    828  CD2 HIS A 224       4.871 -11.710  -1.854  1.00  0.14           C
ATOM    829  CE1 HIS A 224       3.279 -11.541  -3.396  1.00  0.19           C
ATOM    830  NE2 HIS A 224       3.552 -12.062  -2.290  1.00 -0.13           N
ATOM    831  HA  HIS A 224       7.436 -11.440  -4.513  1.00  0.09           H
ATOM    832  HB2 HIS A 224       6.905  -9.924  -1.919  1.00  0.08           H
ATOM    833  HB3 HIS A 224       6.500  -9.316  -3.539  1.00  0.08           H
ATOM    834  HD2 HIS A 224       5.365 -12.009  -0.940  1.00  0.13           H
ATOM    835  HE1 HIS A 224       2.324 -11.649  -3.925  1.00  0.19           H
ATOM    836  HD1 HIS A 224       4.356 -10.305  -4.655  1.00  0.27           H
ATOM    837  H   HIS A 224       8.347 -12.264  -1.740  1.00  0.19           H
ATOM    838  N   GLU A 225      10.162 -11.160  -3.705  1.00 -0.26           N
ATOM    839  CA  GLU A 225      11.450 -10.472  -3.808  1.00  0.13           C
ATOM    840  C   GLU A 225      11.498  -9.674  -5.100  1.00  0.20           C
ATOM    841  O   GLU A 225      11.752  -8.480  -5.070  1.00 -0.39           O
ATOM    842  CB  GLU A 225      12.668 -11.439  -3.728  1.00 -0.00           C
ATOM    843  CG  GLU A 225      12.730 -12.211  -2.381  1.00  0.00           C
ATOM    844  CD  GLU A 225      13.970 -13.046  -2.212  1.00  0.04           C
ATOM    845  OE1 GLU A 225      14.720 -13.220  -3.208  1.00 -0.57           O
ATOM    846  OE2 GLU A 225      14.190 -13.537  -1.070  1.00 -0.57           O
ATOM    847  HA  GLU A 225      11.535  -9.766  -2.968  1.00  0.08           H
ATOM    848  HB2 GLU A 225      12.633 -12.171  -4.553  1.00  0.03           H
ATOM    849  HB3 GLU A 225      13.595 -10.849  -3.830  1.00  0.03           H
ATOM    850  HG2 GLU A 225      12.680 -11.507  -1.536  1.00  0.04           H
ATOM    851  HG3 GLU A 225      11.894 -12.917  -2.312  1.00  0.04           H
```

ATOM 852 H GLU A 225 10.142 -12.160 -3.648 1.00 0.19 H
ATOM 853 N THR A 226 11.255 -10.312 -6.266 1.00 -0.26 N
ATOM 854 CA THR A 226 11.327 -9.585 -7.536 1.00 0.16 C
ATOM 855 C THR A 226 10.463 -8.342 -7.543 1.00 0.21 C
ATOM 856 O THR A 226 10.898 -7.289 -7.985 1.00 -0.39 O
ATOM 857 CB THR A 226 10.859 -10.512 -8.693 1.00 0.09 C
ATOM 858 OG1 THR A 226 11.718 -11.667 -8.672 1.00 -0.39 O
ATOM 859 CG2 THR A 226 10.927 -9.813 -10.075 1.00 -0.03 C
ATOM 860 HA THR A 226 12.373 -9.293 -7.716 1.00 0.08 H
ATOM 861 HB THR A 226 9.815 -10.826 -8.513 1.00 0.06 H
ATOM 862 HG1 THR A 226 11.488 -12.323 -9.324 1.00 0.21 H
ATOM 863 HG23 THR A 226 10.224 -8.967 -10.116 1.00 0.03 H
ATOM 864 HG21 THR A 226 11.947 -9.445 -10.262 1.00 0.03 H
ATOM 865 HG22 THR A 226 10.656 -10.526 -10.870 1.00 0.03 H
ATOM 866 H THR A 226 11.068 -11.295 -6.283 1.00 0.19 H
ATOM 867 N ALA A 227 9.203 -8.434 -7.076 1.00 -0.26 N
ATOM 868 CA ALA A 227 8.344 -7.255 -7.124 1.00 0.13 C
ATOM 869 C ALA A 227 8.813 -6.245 -6.096 1.00 0.20 C
ATOM 870 O ALA A 227 8.791 -5.053 -6.369 1.00 -0.39 O
ATOM 871 CB ALA A 227 6.873 -7.685 -6.911 1.00 -0.02 C
ATOM 872 HA ALA A 227 8.415 -6.790 -8.121 1.00 0.08 H
ATOM 873 HB1 ALA A 227 6.189 -6.840 -7.076 1.00 0.03 H
ATOM 874 HB2 ALA A 227 6.605 -8.479 -7.624 1.00 0.03 H
ATOM 875 HB3 ALA A 227 6.752 -8.074 -5.889 1.00 0.03 H
ATOM 876 H ALA A 227 8.868 -9.285 -6.669 1.00 0.19 H
ATOM 877 N PHE A 228 9.263 -6.689 -4.901 1.00 -0.26 N
ATOM 878 CA PHE A 228 9.849 -5.734 -3.962 1.00 0.14 C
ATOM 879 C PHE A 228 11.052 -5.065 -4.601 1.00 0.21 C
ATOM 880 O PHE A 228 11.176 -3.855 -4.479 1.00 -0.39 O
ATOM 881 CB PHE A 228 10.256 -6.418 -2.630 1.00 0.02 C
ATOM 882 CG PHE A 228 9.087 -6.852 -1.728 1.00 -0.04 C
ATOM 883 CD1 PHE A 228 7.735 -6.711 -2.075 1.00 -0.06 C
ATOM 884 CD2 PHE A 228 9.396 -7.397 -0.475 1.00 -0.06 C
ATOM 885 CE1 PHE A 228 6.739 -6.877 -1.111 1.00 -0.07 C
ATOM 886 CE2 PHE A 228 8.408 -7.548 0.499 1.00 -0.07 C
ATOM 887 CZ PHE A 228 7.079 -7.243 0.194 1.00 -0.07 C
ATOM 888 HA PHE A 228 9.127 -4.934 -3.740 1.00 0.08 H
ATOM 889 HB2 PHE A 228 10.908 -7.280 -2.837 1.00 0.05 H
ATOM 890 HB3 PHE A 228 10.838 -5.688 -2.045 1.00 0.05 H
ATOM 891 HD2 PHE A 228 10.415 -7.697 -0.260 1.00 0.06 H
ATOM 892 HE2 PHE A 228 8.668 -7.896 1.495 1.00 0.06 H
ATOM 893 HZ PHE A 228 6.318 -7.291 0.962 1.00 0.06 H
ATOM 894 HE1 PHE A 228 5.696 -6.719 -1.372 1.00 0.06 H
ATOM 895 HD1 PHE A 228 7.436 -6.469 -3.088 1.00 0.06 H
ATOM 896 H PHE A 228 9.265 -7.667 -4.663 1.00 0.19 H
ATOM 897 N GLN A 229 11.942 -5.807 -5.300 1.00 -0.26 N
ATOM 898 CA GLN A 229 13.044 -5.141 -5.998 1.00 0.13 C
ATOM 899 C GLN A 229 12.477 -4.050 -6.885 1.00 0.20 C
ATOM 900 O GLN A 229 12.880 -2.903 -6.767 1.00 -0.39 O
ATOM 901 CB GLN A 229 13.881 -6.086 -6.914 1.00 0.00 C
ATOM 902 CG GLN A 229 14.770 -7.111 -6.157 1.00 0.04 C

ATOM	903	CD	GLN	A	229	15.964	-6.483	-5.481	1.00	0.17	C
ATOM	904	OE1	GLN	A	229	16.159	-5.280	-5.575	1.00	-0.40	O
ATOM	905	NE2	GLN	A	229	16.802	-7.261	-4.773	1.00	-0.30	N
ATOM	906	HA	GLN	A	229	13.712	-4.671	-5.259	1.00	0.08	H
ATOM	907	HB2	GLN	A	229	13.202	-6.648	-7.570	1.00	0.03	H
ATOM	908	HB3	GLN	A	229	14.537	-5.482	-7.564	1.00	0.03	H
ATOM	909	HG2	GLN	A	229	14.172	-7.664	-5.419	1.00	0.05	H
ATOM	910	HG3	GLN	A	229	15.169	-7.839	-6.881	1.00	0.05	H
ATOM	911	HE22	GLN	A	229	17.591	-6.834	-4.328	1.00	0.18	H
ATOM	912	HE21	GLN	A	229	16.652	-8.245	-4.679	1.00	0.18	H
ATOM	913	H	GLN	A	229	11.835	-6.801	-5.375	1.00	0.19	H
ATOM	914	N	GLY	A	230	11.538	-4.379	-7.801	1.00	-0.27	N
ATOM	915	CA	GLY	A	230	11.075	-3.357	-8.739	1.00	0.12	C
ATOM	916	C	GLY	A	230	10.415	-2.181	-8.061	1.00	0.20	C
ATOM	917	O	GLY	A	230	10.499	-1.072	-8.568	1.00	-0.39	O
ATOM	918	HA3	GLY	A	230	11.939	-2.980	-9.309	1.00	0.08	H
ATOM	919	HA2	GLY	A	230	10.348	-3.771	-9.452	1.00	0.08	H
ATOM	920	H	GLY	A	230	11.170	-5.313	-7.860	1.00	0.19	H
ATOM	921	N	MET	A	231	9.726	-2.383	-6.920	1.00	-0.26	N
ATOM	922	CA	MET	A	231	9.172	-1.222	-6.225	1.00	0.13	C
ATOM	923	C	MET	A	231	10.299	-0.421	-5.606	1.00	0.20	C
ATOM	924	O	MET	A	231	10.308	0.794	-5.728	1.00	-0.39	O
ATOM	925	CB	MET	A	231	8.174	-1.661	-5.124	1.00	-0.00	C
ATOM	926	CG	MET	A	231	7.371	-0.472	-4.535	1.00	-0.00	C
ATOM	927	SD	MET	A	231	6.148	0.103	-5.764	1.00	-0.16	S
ATOM	928	CE	MET	A	231	5.840	1.803	-5.197	1.00	-0.02	C
ATOM	929	HA	MET	A	231	8.634	-0.591	-6.949	1.00	0.08	H
ATOM	930	HB2	MET	A	231	7.465	-2.389	-5.551	1.00	0.03	H
ATOM	931	HB3	MET	A	231	8.749	-2.153	-4.322	1.00	0.03	H
ATOM	932	HG2	MET	A	231	6.811	-0.803	-3.647	1.00	0.04	H
ATOM	933	HG3	MET	A	231	8.049	0.342	-4.241	1.00	0.04	H
ATOM	934	LPD1	MET	A	231	6.404	0.094	-6.393	1.00	0.00	LP
ATOM	935	LPD2	MET	A	231	5.582	-0.271	-5.745	1.00	0.00	LP
ATOM	936	HE1	MET	A	231	6.699	2.431	-5.473	1.00	0.03	H
ATOM	937	HE2	MET	A	231	4.940	2.188	-5.699	1.00	0.03	H
ATOM	938	HE3	MET	A	231	5.691	1.827	-4.107	1.00	0.03	H
ATOM	939	H	MET	A	231	9.633	-3.303	-6.521	1.00	0.19	H
ATOM	940	N	LEU	A	232	11.264	-1.084	-4.929	1.00	-0.26	N
ATOM	941	CA	LEU	A	232	12.374	-0.337	-4.331	1.00	0.13	C
ATOM	942	C	LEU	A	232	13.074	0.499	-5.379	1.00	0.20	C
ATOM	943	O	LEU	A	232	13.246	1.692	-5.161	1.00	-0.39	O
ATOM	944	CB	LEU	A	232	13.371	-1.309	-3.634	1.00	-0.01	C
ATOM	945	CG	LEU	A	232	14.729	-0.673	-3.213	1.00	-0.04	C
ATOM	946	CD1	LEU	A	232	14.562	0.494	-2.201	1.00	-0.06	C
ATOM	947	CD2	LEU	A	232	15.646	-1.767	-2.598	1.00	-0.06	C
ATOM	948	HA	LEU	A	232	11.960	0.352	-3.579	1.00	0.08	H
ATOM	949	HB2	LEU	A	232	12.877	-1.753	-2.754	1.00	0.03	H
ATOM	950	HB3	LEU	A	232	13.601	-2.122	-4.342	1.00	0.03	H
ATOM	951	HG	LEU	A	232	15.234	-0.284	-4.115	1.00	0.03	H
ATOM	952	HD21	LEU	A	232	15.792	-2.596	-3.308	1.00	0.02	H
ATOM	953	HD22	LEU	A	232	16.632	-1.338	-2.370	1.00	0.02	H

```
ATOM    954 HD23 LEU A 232      15.206  -2.167  -1.671  1.00  0.02           H
ATOM    955 HD11 LEU A 232      14.059   0.147  -1.288  1.00  0.02           H
ATOM    956 HD12 LEU A 232      15.549   0.897  -1.922  1.00  0.02           H
ATOM    957 HD13 LEU A 232      13.973   1.313  -2.639  1.00  0.02           H
ATOM    958  H   LEU A 232      11.249  -2.089  -4.855  1.00  0.19           H
ATOM    959  N   ARG A 233      13.478  -0.109  -6.517  1.00 -0.29           N
ATOM    960  CA  ARG A 233      14.137   0.673  -7.561  1.00  0.01           C
ATOM    961  C   ARG A 233      13.250   1.836  -7.947  1.00  0.17           C
ATOM    962  O   ARG A 233      13.673   2.982  -7.903  1.00 -0.40           O
ATOM    963  CB  ARG A 233      14.390  -0.215  -8.807  1.00 -0.30           C
ATOM    964  HA  ARG A 233      15.101   1.052  -7.184  1.00  0.04           H
ATOM    965  HB2 ARG A 233      14.811   0.369  -9.640  1.00 -0.21           H
ATOM    966  HB3 ARG A 233      15.066  -1.055  -8.579  1.00 -0.21           H
ATOM    967  H   ARG A 233      13.322  -1.092  -6.680  1.00  0.19           H
ATOM    968  N   LYS A 234      11.983   1.551  -8.316  1.00 -0.27           N
ATOM    969  CA  LYS A 234      11.098   2.637  -8.736  1.00  0.13           C
ATOM    970  C   LYS A 234      10.997   3.725  -7.686  1.00  0.20           C
ATOM    971  O   LYS A 234      10.831   4.879  -8.053  1.00 -0.39           O
ATOM    972  CB  LYS A 234       9.688   2.078  -9.071  1.00 -0.01           C
ATOM    973  CG  LYS A 234       8.749   3.155  -9.684  1.00 -0.04           C
ATOM    974  CD  LYS A 234       7.341   2.602 -10.059  1.00 -0.01           C
ATOM    975  CE  LYS A 234       7.373   1.555 -11.211  1.00 -0.04           C
ATOM    976  NZ  LYS A 234       6.000   1.177 -11.648  1.00  0.22           N
ATOM    977  HA  LYS A 234      11.537   3.083  -9.645  1.00  0.08           H
ATOM    978  HB2 LYS A 234       9.837   1.265  -9.797  1.00  0.03           H
ATOM    979  HB3 LYS A 234       9.236   1.660  -8.155  1.00  0.03           H
ATOM    980  HG2 LYS A 234       8.603   3.960  -8.944  1.00  0.03           H
ATOM    981  HG3 LYS A 234       9.214   3.596 -10.583  1.00  0.03           H
ATOM    982  HD2 LYS A 234       6.874   2.161  -9.160  1.00  0.03           H
ATOM    983  HD3 LYS A 234       6.720   3.456 -10.387  1.00  0.03           H
ATOM    984  HE2 LYS A 234       7.937   1.980 -12.063  1.00  0.08           H
ATOM    985  HE3 LYS A 234       7.896   0.643 -10.874  1.00  0.08           H
ATOM    986  HZ1 LYS A 234       5.441   2.022 -12.025  1.00  0.20           H
ATOM    987  HZ2 LYS A 234       6.018   0.438 -12.439  1.00  0.20           H
ATOM    988  HZ3 LYS A 234       5.424   0.746 -10.840  1.00  0.20           H
ATOM    989  H   LYS A 234      11.669   0.593  -8.319  1.00  0.19           H
ATOM    990  N   LEU A 235      11.082   3.396  -6.379  1.00 -0.26           N
ATOM    991  CA  LEU A 235      11.092   4.455  -5.374  1.00  0.13           C
ATOM    992  C   LEU A 235      12.445   5.116  -5.218  1.00  0.20           C
ATOM    993  O   LEU A 235      12.438   6.273  -4.831  1.00 -0.39           O
ATOM    994  CB  LEU A 235      10.716   3.911  -3.979  1.00 -0.01           C
ATOM    995  CG  LEU A 235       9.260   3.393  -3.871  1.00 -0.04           C
ATOM    996  CD1 LEU A 235       9.041   2.749  -2.478  1.00 -0.06           C
ATOM    997  CD2 LEU A 235       8.227   4.526  -4.089  1.00 -0.06           C
ATOM    998  HA  LEU A 235      10.393   5.252  -5.659  1.00  0.08           H
ATOM    999  HB2 LEU A 235      11.417   3.098  -3.741  1.00  0.03           H
ATOM   1000  HB3 LEU A 235      10.847   4.711  -3.241  1.00  0.03           H
ATOM   1001  HG  LEU A 235       9.089   2.626  -4.639  1.00  0.03           H
ATOM   1002 HD21 LEU A 235       8.170   4.830  -5.144  1.00  0.02           H
ATOM   1003 HD22 LEU A 235       7.242   4.152  -3.794  1.00  0.02           H
ATOM   1004 HD23 LEU A 235       8.464   5.401  -3.468  1.00  0.02           H
```

```
ATOM   1005 HD11 LEU A 235       9.032   3.513  -1.686  1.00  0.02           H
ATOM   1006 HD12 LEU A 235       8.082   2.218  -2.467  1.00  0.02           H
ATOM   1007 HD13 LEU A 235       9.831   2.019  -2.255  1.00  0.02           H
ATOM   1008  H   LEU A 235      11.202   2.434  -6.099  1.00  0.19           H
ATOM   1009  N   ASP A 236      13.590   4.441  -5.464  1.00 -0.26           N
ATOM   1010  CA  ASP A 236      14.882   5.110  -5.282  1.00  0.14           C
ATOM   1011  C   ASP A 236      15.024   5.598  -3.848  1.00  0.21           C
ATOM   1012  O   ASP A 236      15.206   6.783  -3.607  1.00 -0.39           O
ATOM   1013  CB  ASP A 236      15.050   6.228  -6.345  1.00  0.04           C
ATOM   1014  CG  ASP A 236      16.414   6.851  -6.252  1.00  0.04           C
ATOM   1015  OD1 ASP A 236      17.410   6.092  -6.346  1.00 -0.57           O
ATOM   1016  OD2 ASP A 236      16.503   8.100  -6.066  1.00 -0.57           O
ATOM   1017  HA  ASP A 236      15.699   4.391  -5.451  1.00  0.08           H
ATOM   1018  HB2 ASP A 236      14.933   5.797  -7.350  1.00  0.05           H
ATOM   1019  HB3 ASP A 236      14.293   7.015  -6.216  1.00  0.05           H
ATOM   1020  H   ASP A 236      13.569   3.485  -5.781  1.00  0.19           H
ATOM   1021  N   ILE A 237      14.949   4.682  -2.855  1.00 -0.26           N
ATOM   1022  CA  ILE A 237      15.097   5.090  -1.458  1.00  0.13           C
ATOM   1023  C   ILE A 237      16.564   5.124  -1.082  1.00  0.20           C
ATOM   1024  O   ILE A 237      17.148   4.057  -0.999  1.00 -0.39           O
ATOM   1025  CB  ILE A 237      14.333   4.141  -0.482  1.00 -0.00           C
ATOM   1026  CG1 ILE A 237      12.810   4.000  -0.787  1.00 -0.05           C
ATOM   1027  CG2 ILE A 237      14.555   4.572   0.992  1.00 -0.06           C
ATOM   1028  CD1 ILE A 237      11.990   5.318  -0.733  1.00 -0.06           C
ATOM   1029  HA  ILE A 237      14.673   6.092  -1.313  1.00  0.08           H
ATOM   1030  HB  ILE A 237      14.769   3.133  -0.586  1.00  0.03           H
ATOM   1031 HG12 ILE A 237      12.699   3.554  -1.784  1.00  0.03           H
ATOM   1032 HG13 ILE A 237      12.365   3.300  -0.061  1.00  0.03           H
ATOM   1033 HD11 ILE A 237      12.279   5.979  -1.563  1.00  0.02           H
ATOM   1034 HD12 ILE A 237      10.916   5.095  -0.833  1.00  0.02           H
ATOM   1035 HD13 ILE A 237      12.115   5.853   0.219  1.00  0.02           H
ATOM   1036 HG21 ILE A 237      14.212   5.599   1.170  1.00  0.02           H
ATOM   1037 HG22 ILE A 237      14.016   3.894   1.668  1.00  0.02           H
ATOM   1038 HG23 ILE A 237      15.622   4.525   1.242  1.00  0.02           H
ATOM   1039  H   ILE A 237      14.853   3.713  -3.067  1.00  0.19           H
ATOM   1040  N   LYS A 238      17.196   6.298  -0.844  1.00 -0.26           N
ATOM   1041  CA  LYS A 238      18.619   6.331  -0.516  1.00  0.13           C
ATOM   1042  C   LYS A 238      18.962   7.297   0.613  1.00  0.20           C
ATOM   1043  O   LYS A 238      20.138   7.563   0.802  1.00 -0.39           O
ATOM   1044  CB  LYS A 238      19.515   6.604  -1.769  1.00 -0.01           C
ATOM   1045  CG  LYS A 238      18.838   6.550  -3.182  1.00 -0.04           C
ATOM   1046  CD  LYS A 238      18.940   7.889  -3.976  1.00 -0.01           C
ATOM   1047  CE  LYS A 238      18.038   9.042  -3.441  1.00 -0.04           C
ATOM   1048  NZ  LYS A 238      16.570   8.884  -3.636  1.00  0.22           N
ATOM   1049  HA  LYS A 238      18.902   5.366  -0.075  1.00  0.08           H
ATOM   1050  HB2 LYS A 238      19.983   7.590  -1.657  1.00  0.03           H
ATOM   1051  HB3 LYS A 238      20.352   5.888  -1.742  1.00  0.03           H
ATOM   1052  HG2 LYS A 238      19.354   5.783  -3.787  1.00  0.03           H
ATOM   1053  HG3 LYS A 238      17.786   6.254  -3.171  1.00  0.03           H
ATOM   1054  HD2 LYS A 238      20.000   8.201  -3.952  1.00  0.03           H
ATOM   1055  HD3 LYS A 238      18.705   7.730  -5.038  1.00  0.03           H
```

ATOM	1056	HE2	LYS	A	238	18.254	9.176	-2.373	1.00	0.08	H
ATOM	1057	HE3	LYS	A	238	18.325	9.968	-3.963	1.00	0.08	H
ATOM	1058	HZ1	LYS	A	238	16.163	8.060	-3.076	1.00	0.20	H
ATOM	1059	HZ2	LYS	A	238	15.998	9.705	-3.229	1.00	0.20	H
ATOM	1060	HZ3	LYS	A	238	16.274	8.757	-4.659	1.00	0.20	H
ATOM	1061	H	LYS	A	238	16.723	7.175	-0.953	1.00	0.19	H
ATOM	1062	N	ASN	A	239	18.020	7.858	1.405	1.00	-0.26	N
ATOM	1063	CA	ASN	A	239	18.395	8.850	2.417	1.00	0.15	C
ATOM	1064	C	ASN	A	239	17.267	9.051	3.416	1.00	0.21	C
ATOM	1065	O	ASN	A	239	16.222	8.439	3.256	1.00	-0.39	O
ATOM	1066	CB	ASN	A	239	18.796	10.172	1.708	1.00	0.08	C
ATOM	1067	CG	ASN	A	239	17.632	10.685	0.909	1.00	0.18	C
ATOM	1068	OD1	ASN	A	239	16.555	10.764	1.470	1.00	-0.40	O
ATOM	1069	ND2	ASN	A	239	17.762	11.055	-0.377	1.00	-0.30	N
ATOM	1070	HA	ASN	A	239	19.251	8.488	3.003	1.00	0.08	H
ATOM	1071	HB2	ASN	A	239	19.064	10.948	2.437	1.00	0.06	H
ATOM	1072	HB3	ASN	A	239	19.670	10.012	1.062	1.00	0.06	H
ATOM	1073	HD22	ASN	A	239	16.930	11.319	-0.874	1.00	0.18	H
ATOM	1074	HD21	ASN	A	239	18.644	11.059	-0.843	1.00	0.18	H
ATOM	1075	H	ASN	A	239	17.046	7.656	1.308	1.00	0.19	H
ATOM	1076	N	GLU	A	240	17.433	9.892	4.462	1.00	-0.26	N
ATOM	1077	CA	GLU	A	240	16.326	10.090	5.399	1.00	0.13	C
ATOM	1078	C	GLU	A	240	15.126	10.682	4.678	1.00	0.20	C
ATOM	1079	O	GLU	A	240	14.015	10.208	4.872	1.00	-0.39	O
ATOM	1080	CB	GLU	A	240	16.711	10.959	6.628	1.00	-0.00	C
ATOM	1081	CG	GLU	A	240	17.767	10.252	7.524	1.00	0.00	C
ATOM	1082	CD	GLU	A	240	18.089	11.015	8.781	1.00	0.04	C
ATOM	1083	OE1	GLU	A	240	17.352	11.980	9.115	1.00	-0.57	O
ATOM	1084	OE2	GLU	A	240	19.090	10.640	9.448	1.00	-0.57	O
ATOM	1085	HA	GLU	A	240	16.032	9.111	5.798	1.00	0.08	H
ATOM	1086	HB2	GLU	A	240	17.095	11.938	6.293	1.00	0.03	H
ATOM	1087	HB3	GLU	A	240	15.803	11.131	7.232	1.00	0.03	H
ATOM	1088	HG2	GLU	A	240	17.399	9.265	7.844	1.00	0.04	H
ATOM	1089	HG3	GLU	A	240	18.703	10.112	6.963	1.00	0.04	H
ATOM	1090	H	GLU	A	240	18.281	10.400	4.605	1.00	0.19	H
ATOM	1091	N	ASP	A	241	15.316	11.721	3.835	1.00	-0.26	N
ATOM	1092	CA	ASP	A	241	14.172	12.337	3.163	1.00	0.14	C
ATOM	1093	C	ASP	A	241	13.359	11.312	2.400	1.00	0.21	C
ATOM	1094	O	ASP	A	241	12.172	11.186	2.665	1.00	-0.39	O
ATOM	1095	CB	ASP	A	241	14.594	13.477	2.202	1.00	0.04	C
ATOM	1096	CG	ASP	A	241	15.450	14.432	2.981	1.00	0.04	C
ATOM	1097	OD1	ASP	A	241	16.538	13.973	3.427	1.00	-0.57	O
ATOM	1098	OD2	ASP	A	241	15.041	15.609	3.164	1.00	-0.57	O
ATOM	1099	HA	ASP	A	241	13.531	12.782	3.939	1.00	0.08	H
ATOM	1100	HB2	ASP	A	241	15.194	13.091	1.364	1.00	0.05	H
ATOM	1101	HB3	ASP	A	241	13.711	13.991	1.791	1.00	0.05	H
ATOM	1102	H	ASP	A	241	16.223	12.135	3.693	1.00	0.19	H
ATOM	1103	N	ASP	A	242	13.966	10.554	1.459	1.00	-0.26	N
ATOM	1104	CA	ASP	A	242	13.214	9.531	0.748	1.00	0.14	C
ATOM	1105	C	ASP	A	242	12.584	8.602	1.765	1.00	0.21	C
ATOM	1106	O	ASP	A	242	11.426	8.235	1.639	1.00	-0.39	O

ATOM 1107 CB ASP A 242 14.076 8.609 -0.154 1.00 0.04 C
ATOM 1108 CG ASP A 242 15.032 9.210 -1.141 1.00 0.04 C
ATOM 1109 OD1 ASP A 242 14.826 10.295 -1.737 1.00 -0.57 O
ATOM 1110 OD2 ASP A 242 16.067 8.549 -1.415 1.00 -0.57 O
ATOM 1111 HA ASP A 242 12.435 10.032 0.152 1.00 0.08 H
ATOM 1112 HB2 ASP A 242 14.700 7.958 0.476 1.00 0.05 H
ATOM 1113 HB3 ASP A 242 13.390 7.979 -0.735 1.00 0.05 H
ATOM 1114 H ASP A 242 14.936 10.666 1.251 1.00 0.19 H
ATOM 1115 N VAL A 243 13.346 8.185 2.799 1.00 -0.26 N
ATOM 1116 CA VAL A 243 12.759 7.293 3.792 1.00 0.13 C
ATOM 1117 C VAL A 243 11.550 7.932 4.439 1.00 0.20 C
ATOM 1118 O VAL A 243 10.660 7.200 4.842 1.00 -0.39 O
ATOM 1119 CB VAL A 243 13.799 6.794 4.834 1.00 -0.01 C
ATOM 1120 CG1 VAL A 243 13.118 6.193 6.083 1.00 -0.06 C
ATOM 1121 CG2 VAL A 243 14.732 5.718 4.215 1.00 -0.06 C
ATOM 1122 HA VAL A 243 12.357 6.419 3.258 1.00 0.08 H
ATOM 1123 HB VAL A 243 14.389 7.651 5.172 1.00 0.03 H
ATOM 1124 HG11 VAL A 243 12.481 5.380 5.719 1.00 0.02 H
ATOM 1125 HG12 VAL A 243 13.860 5.794 6.789 1.00 0.02 H
ATOM 1126 HG13 VAL A 243 12.508 6.930 6.625 1.00 0.02 H
ATOM 1127 HG21 VAL A 243 15.277 6.120 3.353 1.00 0.02 H
ATOM 1128 HG22 VAL A 243 15.469 5.347 4.937 1.00 0.02 H
ATOM 1129 HG23 VAL A 243 14.139 4.853 3.891 1.00 0.02 H
ATOM 1130 H VAL A 243 14.291 8.502 2.915 1.00 0.19 H
ATOM 1131 N LYS A 244 11.431 9.275 4.535 1.00 -0.26 N
ATOM 1132 CA LYS A 244 10.186 9.874 5.025 1.00 0.13 C
ATOM 1133 C LYS A 244 9.186 10.114 3.906 1.00 0.20 C
ATOM 1134 O LYS A 244 8.431 11.068 4.003 1.00 -0.39 O
ATOM 1135 CB LYS A 244 10.464 11.131 5.916 1.00 -0.01 C
ATOM 1136 CG LYS A 244 11.392 10.855 7.146 1.00 -0.04 C
ATOM 1137 CD LYS A 244 10.723 10.030 8.293 1.00 -0.01 C
ATOM 1138 CE LYS A 244 9.969 10.850 9.388 1.00 -0.04 C
ATOM 1139 NZ LYS A 244 10.786 11.021 10.627 1.00 0.22 N
ATOM 1140 HA LYS A 244 9.632 9.157 5.648 1.00 0.08 H
ATOM 1141 HB2 LYS A 244 10.951 11.877 5.265 1.00 0.03 H
ATOM 1142 HB3 LYS A 244 9.514 11.560 6.277 1.00 0.03 H
ATOM 1143 HG2 LYS A 244 12.275 10.305 6.780 1.00 0.03 H
ATOM 1144 HG3 LYS A 244 11.751 11.808 7.571 1.00 0.03 H
ATOM 1145 HD2 LYS A 244 10.037 9.314 7.836 1.00 0.03 H
ATOM 1146 HD3 LYS A 244 11.478 9.421 8.801 1.00 0.03 H
ATOM 1147 HE2 LYS A 244 9.649 11.824 8.976 1.00 0.08 H
ATOM 1148 HE3 LYS A 244 9.056 10.290 9.679 1.00 0.08 H
ATOM 1149 HZ1 LYS A 244 11.740 11.492 10.446 1.00 0.20 H
ATOM 1150 HZ2 LYS A 244 10.286 11.568 11.416 1.00 0.20 H
ATOM 1151 HZ3 LYS A 244 11.022 10.070 11.083 1.00 0.20 H
ATOM 1152 H LYS A 244 12.160 9.887 4.219 1.00 0.19 H
ATOM 1153 N SER A 245 9.100 9.269 2.850 1.00 -0.26 N
ATOM 1154 CA SER A 245 8.041 9.423 1.847 1.00 0.15 C
ATOM 1155 C SER A 245 7.475 8.078 1.446 1.00 0.21 C
ATOM 1156 O SER A 245 7.396 7.751 0.275 1.00 -0.39 O
ATOM 1157 CB SER A 245 8.593 10.205 0.629 1.00 0.08 C

ATOM 1158 OG SER A 245 9.593 9.429 -0.049 1.00 -0.39 O
ATOM 1159 HA SER A 245 7.186 9.984 2.255 1.00 0.08 H
ATOM 1160 HB2 SER A 245 7.769 10.427 -0.069 1.00 0.06 H
ATOM 1161 HB3 SER A 245 9.028 11.154 0.989 1.00 0.06 H
ATOM 1162 HG SER A 245 9.982 9.877 -0.792 1.00 0.21 H
ATOM 1163 H SER A 245 9.775 8.543 2.694 1.00 0.19 H
ATOM 1164 N LEU A 246 7.033 7.252 2.414 1.00 -0.26 N
ATOM 1165 CA LEU A 246 6.650 5.873 2.106 1.00 0.13 C
ATOM 1166 C LEU A 246 5.191 5.824 1.738 1.00 0.20 C
ATOM 1167 O LEU A 246 4.412 5.095 2.330 1.00 -0.39 O
ATOM 1168 CB LEU A 246 6.954 4.929 3.292 1.00 -0.01 C
ATOM 1169 CG LEU A 246 8.407 5.077 3.814 1.00 -0.04 C
ATOM 1170 CD1 LEU A 246 8.526 4.347 5.169 1.00 -0.06 C
ATOM 1171 CD2 LEU A 246 9.475 4.549 2.818 1.00 -0.06 C
ATOM 1172 HA LEU A 246 7.204 5.491 1.237 1.00 0.08 H
ATOM 1173 HB2 LEU A 246 6.254 5.210 4.092 1.00 0.03 H
ATOM 1174 HB3 LEU A 246 6.750 3.881 3.025 1.00 0.03 H
ATOM 1175 HG LEU A 246 8.620 6.134 4.009 1.00 0.03 H
ATOM 1176 HD21 LEU A 246 9.494 5.138 1.892 1.00 0.02 H
ATOM 1177 HD22 LEU A 246 10.473 4.615 3.272 1.00 0.02 H
ATOM 1178 HD23 LEU A 246 9.290 3.498 2.555 1.00 0.02 H
ATOM 1179 HD11 LEU A 246 8.569 3.273 4.962 1.00 0.02 H
ATOM 1180 HD12 LEU A 246 9.425 4.671 5.706 1.00 0.02 H
ATOM 1181 HD13 LEU A 246 7.669 4.546 5.823 1.00 0.02 H
ATOM 1182 H LEU A 246 6.940 7.591 3.347 1.00 0.19 H
ATOM 1183 N SER A 247 4.800 6.610 0.721 1.00 -0.26 N
ATOM 1184 CA SER A 247 3.462 6.487 0.166 1.00 0.15 C
ATOM 1185 C SER A 247 3.651 5.724 -1.120 1.00 0.21 C
ATOM 1186 O SER A 247 4.697 5.121 -1.281 1.00 -0.39 O
ATOM 1187 CB SER A 247 2.898 7.920 0.018 1.00 0.08 C
ATOM 1188 OG SER A 247 3.685 8.631 -0.954 1.00 -0.39 O
ATOM 1189 HA SER A 247 2.773 5.913 0.812 1.00 0.08 H
ATOM 1190 HB2 SER A 247 1.836 7.895 -0.281 1.00 0.06 H
ATOM 1191 HB3 SER A 247 2.966 8.424 1.001 1.00 0.06 H
ATOM 1192 HG SER A 247 3.417 9.538 -1.046 1.00 0.21 H
ATOM 1193 H SER A 247 5.446 7.193 0.221 1.00 0.19 H
ATOM 1194 N ARG A 248 2.689 5.658 -2.056 1.00 -0.26 N
ATOM 1195 CA ARG A 248 2.872 4.795 -3.229 1.00 0.13 C
ATOM 1196 C ARG A 248 2.858 3.340 -2.799 1.00 0.20 C
ATOM 1197 O ARG A 248 1.988 2.619 -3.255 1.00 -0.39 O
ATOM 1198 CB ARG A 248 4.094 5.145 -4.123 1.00 -0.01 C
ATOM 1199 CG ARG A 248 4.288 6.678 -4.315 1.00 -0.02 C
ATOM 1200 CD ARG A 248 4.908 6.997 -5.704 1.00 0.06 C
ATOM 1201 NE ARG A 248 5.281 8.413 -5.831 1.00 -0.27 N
ATOM 1202 CZ ARG A 248 6.453 8.915 -5.501 1.00 0.29 C
ATOM 1203 NH1 ARG A 248 7.394 8.215 -4.918 1.00 -0.28 N
ATOM 1204 NH2 ARG A 248 6.701 10.176 -5.765 1.00 -0.28 N
ATOM 1205 HA ARG A 248 2.004 4.954 -3.889 1.00 0.08 H
ATOM 1206 HB2 ARG A 248 5.006 4.721 -3.685 1.00 0.03 H
ATOM 1207 HB3 ARG A 248 3.955 4.652 -5.099 1.00 0.03 H
ATOM 1208 HG2 ARG A 248 3.319 7.197 -4.242 1.00 0.03 H

ATOM 1209 HG3 ARG A 248 4.932 7.069 -3.512 1.00 0.03 H
ATOM 1210 HD2 ARG A 248 5.728 6.309 -5.960 1.00 0.07 H
ATOM 1211 HD3 ARG A 248 4.124 6.777 -6.451 1.00 0.07 H
ATOM 1212 HE ARG A 248 4.579 9.034 -6.264 1.00 0.26 H
ATOM 1213 HH12 ARG A 248 8.310 8.628 -4.693 1.00 0.26 H
ATOM 1214 HH11 ARG A 248 7.242 7.242 -4.640 1.00 0.26 H
ATOM 1215 HH22 ARG A 248 7.608 10.604 -5.529 1.00 0.26 H
ATOM 1216 HH21 ARG A 248 6.000 10.778 -6.222 1.00 0.26 H
ATOM 1217 H ARG A 248 1.830 6.159 -1.941 1.00 0.19 H
ATOM 1218 N VAL A 249 3.752 2.859 -1.905 1.00 -0.26 N
ATOM 1219 CA VAL A 249 3.555 1.531 -1.320 1.00 0.13 C
ATOM 1220 C VAL A 249 2.156 1.478 -0.758 1.00 0.20 C
ATOM 1221 O VAL A 249 1.457 0.505 -0.977 1.00 -0.39 O
ATOM 1222 CB VAL A 249 4.558 1.116 -0.200 1.00 -0.01 C
ATOM 1223 CG1 VAL A 249 6.018 1.105 -0.714 1.00 -0.06 C
ATOM 1224 CG2 VAL A 249 4.491 1.995 1.074 1.00 -0.06 C
ATOM 1225 HA VAL A 249 3.619 0.788 -2.129 1.00 0.08 H
ATOM 1226 HB VAL A 249 4.296 0.089 0.102 1.00 0.03 H
ATOM 1227 HG11 VAL A 249 6.370 2.125 -0.914 1.00 0.02 H
ATOM 1228 HG12 VAL A 249 6.688 0.671 0.038 1.00 0.02 H
ATOM 1229 HG13 VAL A 249 6.093 0.505 -1.629 1.00 0.02 H
ATOM 1230 HG21 VAL A 249 3.526 1.908 1.589 1.00 0.02 H
ATOM 1231 HG22 VAL A 249 5.268 1.683 1.788 1.00 0.02 H
ATOM 1232 HG23 VAL A 249 4.667 3.047 0.817 1.00 0.02 H
ATOM 1233 H VAL A 249 4.503 3.438 -1.585 1.00 0.19 H
ATOM 1234 N MET A 250 1.698 2.525 -0.045 1.00 -0.26 N
ATOM 1235 CA MET A 250 0.343 2.496 0.504 1.00 0.13 C
ATOM 1236 C MET A 250 -0.675 2.462 -0.605 1.00 0.20 C
ATOM 1237 O MET A 250 -1.645 1.727 -0.501 1.00 -0.39 O
ATOM 1238 CB MET A 250 0.057 3.750 1.371 1.00 -0.00 C
ATOM 1239 CG MET A 250 0.680 3.614 2.782 1.00 -0.00 C
ATOM 1240 SD MET A 250 -0.130 2.301 3.751 1.00 -0.16 S
ATOM 1241 CE MET A 250 -1.840 2.907 3.919 1.00 -0.02 C
ATOM 1242 HA MET A 250 0.215 1.578 1.099 1.00 0.08 H
ATOM 1243 HB2 MET A 250 0.454 4.642 0.863 1.00 0.03 H
ATOM 1244 HB3 MET A 250 -1.026 3.906 1.471 1.00 0.03 H
ATOM 1245 HG2 MET A 250 1.752 3.384 2.708 1.00 0.04 H
ATOM 1246 HG3 MET A 250 0.567 4.551 3.335 1.00 0.04 H
ATOM 1247 LPD1 MET A 250 -0.113 1.708 3.420 1.00 0.00 LP
ATOM 1248 LPD2 MET A 250 0.164 2.230 4.358 1.00 0.00 LP
ATOM 1249 HE1 MET A 250 -2.411 2.798 2.988 1.00 0.03 H
ATOM 1250 HE2 MET A 250 -2.338 2.315 4.699 1.00 0.03 H
ATOM 1251 HE3 MET A 250 -1.827 3.968 4.203 1.00 0.03 H
ATOM 1252 H MET A 250 2.271 3.330 0.092 1.00 0.19 H
ATOM 1253 N ILE A 251 -0.478 3.247 -1.683 1.00 -0.26 N
ATOM 1254 CA ILE A 251 -1.423 3.179 -2.797 1.00 0.13 C
ATOM 1255 C ILE A 251 -1.386 1.757 -3.299 1.00 0.20 C
ATOM 1256 O ILE A 251 -2.428 1.162 -3.496 1.00 -0.39 O
ATOM 1257 CB ILE A 251 -1.078 4.157 -3.968 1.00 -0.00 C
ATOM 1258 CG1 ILE A 251 -1.202 5.644 -3.503 1.00 -0.05 C
ATOM 1259 CG2 ILE A 251 -1.970 3.882 -5.216 1.00 -0.06 C

ATOM 1260 CD1 ILE A 251 -0.663 6.689 -4.521 1.00 -0.06 C
ATOM 1261 HA ILE A 251 -2.442 3.403 -2.438 1.00 0.08 H
ATOM 1262 HB ILE A 251 -0.041 3.954 -4.274 1.00 0.03 H
ATOM 1263 HG12 ILE A 251 -2.260 5.880 -3.293 1.00 0.03 H
ATOM 1264 HG13 ILE A 251 -0.632 5.772 -2.569 1.00 0.03 H
ATOM 1265 HD11 ILE A 251 -1.296 6.733 -5.421 1.00 0.02 H
ATOM 1266 HD12 ILE A 251 -0.660 7.696 -4.066 1.00 0.02 H
ATOM 1267 HD13 ILE A 251 0.368 6.443 -4.820 1.00 0.02 H
ATOM 1268 HG21 ILE A 251 -3.032 4.071 -4.982 1.00 0.02 H
ATOM 1269 HG22 ILE A 251 -1.672 4.521 -6.063 1.00 0.02 H
ATOM 1270 HG23 ILE A 251 -1.864 2.837 -5.552 1.00 0.02 H
ATOM 1271 H ILE A 251 0.343 3.808 -1.761 1.00 0.19 H
ATOM 1272 N HIS A 252 -0.200 1.168 -3.535 1.00 -0.26 N
ATOM 1273 CA HIS A 252 -0.176 -0.164 -4.132 1.00 0.14 C
ATOM 1274 C HIS A 252 -0.729 -1.201 -3.177 1.00 0.21 C
ATOM 1275 O HIS A 252 -1.456 -2.077 -3.614 1.00 -0.39 O
ATOM 1276 CB HIS A 252 1.264 -0.518 -4.578 1.00 0.04 C
ATOM 1277 CG HIS A 252 1.248 -1.653 -5.557 1.00 0.06 C
ATOM 1278 ND1 HIS A 252 1.627 -2.880 -5.290 1.00 -0.26 N
ATOM 1279 CD2 HIS A 252 0.843 -1.564 -6.845 1.00 -0.03 C
ATOM 1280 CE1 HIS A 252 1.497 -3.618 -6.343 1.00 0.09 C
ATOM 1281 NE2 HIS A 252 1.050 -2.919 -7.283 1.00 -0.34 N
ATOM 1282 HA HIS A 252 -0.819 -0.143 -5.026 1.00 0.08 H
ATOM 1283 HB2 HIS A 252 1.704 0.347 -5.098 1.00 0.05 H
ATOM 1284 HB3 HIS A 252 1.893 -0.743 -3.703 1.00 0.05 H
ATOM 1285 HD2 HIS A 252 0.466 -0.707 -7.395 1.00 0.03 H
ATOM 1286 HE1 HIS A 252 1.726 -4.683 -6.449 1.00 0.11 H
ATOM 1287 HD1 HIS A 252 1.972 -3.204 -4.379 1.00 0.24 H
ATOM 1288 H HIS A 252 0.657 1.637 -3.307 1.00 0.19 H
ATOM 1289 N VAL A 253 -0.406 -1.135 -1.869 1.00 -0.26 N
ATOM 1290 CA VAL A 253 -0.939 -2.116 -0.925 1.00 0.13 C
ATOM 1291 C VAL A 253 -2.430 -1.922 -0.763 1.00 0.20 C
ATOM 1292 O VAL A 253 -3.158 -2.902 -0.759 1.00 -0.39 O
ATOM 1293 CB VAL A 253 -0.278 -1.994 0.479 1.00 -0.01 C
ATOM 1294 CG1 VAL A 253 -0.978 -2.904 1.523 1.00 -0.06 C
ATOM 1295 CG2 VAL A 253 1.222 -2.379 0.441 1.00 -0.06 C
ATOM 1296 HA VAL A 253 -0.758 -3.131 -1.310 1.00 0.08 H
ATOM 1297 HB VAL A 253 -0.364 -0.947 0.814 1.00 0.03 H
ATOM 1298 HG11 VAL A 253 -0.944 -3.951 1.188 1.00 0.02 H
ATOM 1299 HG12 VAL A 253 -0.477 -2.832 2.501 1.00 0.02 H
ATOM 1300 HG13 VAL A 253 -2.027 -2.610 1.652 1.00 0.02 H
ATOM 1301 HG21 VAL A 253 1.739 -1.874 -0.384 1.00 0.02 H
ATOM 1302 HG22 VAL A 253 1.709 -2.097 1.388 1.00 0.02 H
ATOM 1303 HG23 VAL A 253 1.334 -3.464 0.304 1.00 0.02 H
ATOM 1304 H VAL A 253 0.169 -0.388 -1.531 1.00 0.19 H
ATOM 1305 N PHE A 254 -2.915 -0.670 -0.602 1.00 -0.26 N
ATOM 1306 CA PHE A 254 -4.343 -0.449 -0.383 1.00 0.14 C
ATOM 1307 C PHE A 254 -4.953 0.244 -1.581 1.00 0.21 C
ATOM 1308 O PHE A 254 -5.802 1.104 -1.400 1.00 -0.39 O
ATOM 1309 CB PHE A 254 -4.540 0.354 0.930 1.00 0.02 C
ATOM 1310 CG PHE A 254 -3.881 -0.368 2.114 1.00 -0.04 C

ATOM 1311 CD1 PHE A 254 -4.571 -1.359 2.826 1.00 -0.06 C
ATOM 1312 CD2 PHE A 254 -2.573 -0.052 2.505 1.00 -0.06 C
ATOM 1313 CE1 PHE A 254 -3.963 -2.025 3.890 1.00 -0.07 C
ATOM 1314 CE2 PHE A 254 -1.986 -0.673 3.605 1.00 -0.07 C
ATOM 1315 CZ PHE A 254 -2.676 -1.665 4.307 1.00 -0.07 C
ATOM 1316 HA PHE A 254 -4.905 -1.386 -0.266 1.00 0.08 H
ATOM 1317 HB2 PHE A 254 -4.097 1.356 0.810 1.00 0.05 H
ATOM 1318 HB3 PHE A 254 -5.617 0.485 1.126 1.00 0.05 H
ATOM 1319 HD2 PHE A 254 -2.007 0.678 1.941 1.00 0.06 H
ATOM 1320 HE2 PHE A 254 -0.984 -0.401 3.920 1.00 0.06 H
ATOM 1321 HZ PHE A 254 -2.219 -2.156 5.157 1.00 0.06 H
ATOM 1322 HE1 PHE A 254 -4.473 -2.832 4.410 1.00 0.06 H
ATOM 1323 HD1 PHE A 254 -5.588 -1.609 2.542 1.00 0.06 H
ATOM 1324 H PHE A 254 -2.317 0.137 -0.643 1.00 0.19 H
ATOM 1325 N SER A 255 -4.552 -0.114 -2.821 1.00 -0.26 N
ATOM 1326 CA SER A 255 -5.071 0.587 -4.001 1.00 0.15 C
ATOM 1327 C SER A 255 -6.582 0.555 -4.046 1.00 0.21 C
ATOM 1328 O SER A 255 -7.207 1.553 -4.367 1.00 -0.39 O
ATOM 1329 CB SER A 255 -4.527 -0.027 -5.319 1.00 0.08 C
ATOM 1330 OG SER A 255 -4.845 0.841 -6.424 1.00 -0.39 O
ATOM 1331 HA SER A 255 -4.762 1.643 -3.954 1.00 0.08 H
ATOM 1332 HB2 SER A 255 -3.433 -0.142 -5.265 1.00 0.06 H
ATOM 1333 HB3 SER A 255 -4.968 -1.028 -5.467 1.00 0.06 H
ATOM 1334 HG SER A 255 -4.518 0.515 -7.258 1.00 0.21 H
ATOM 1335 H SER A 255 -3.846 -0.814 -2.941 1.00 0.19 H
ATOM 1336 N ASP A 256 -7.198 -0.596 -3.707 1.00 -0.26 N
ATOM 1337 CA ASP A 256 -8.658 -0.680 -3.651 1.00 0.14 C
ATOM 1338 C ASP A 256 -9.194 -0.254 -2.297 1.00 0.21 C
ATOM 1339 O ASP A 256 -10.352 -0.514 -2.014 1.00 -0.39 O
ATOM 1340 CB ASP A 256 -9.110 -2.125 -4.007 1.00 0.04 C
ATOM 1341 CG ASP A 256 -8.383 -3.158 -3.189 1.00 0.04 C
ATOM 1342 OD1 ASP A 256 -7.261 -2.874 -2.675 1.00 -0.57 O
ATOM 1343 OD2 ASP A 256 -8.855 -4.309 -3.041 1.00 -0.57 O
ATOM 1344 HA ASP A 256 -9.120 -0.003 -4.387 1.00 0.08 H
ATOM 1345 HB2 ASP A 256 -10.197 -2.249 -3.885 1.00 0.05 H
ATOM 1346 HB3 ASP A 256 -8.854 -2.332 -5.057 1.00 0.05 H
ATOM 1347 H ASP A 256 -6.677 -1.401 -3.402 1.00 0.19 H
ATOM 1348 N GLY A 257 -8.409 0.391 -1.401 1.00 -0.27 N
ATOM 1349 CA GLY A 257 -8.936 0.748 -0.084 1.00 0.12 C
ATOM 1350 C GLY A 257 -9.136 -0.416 0.866 1.00 0.20 C
ATOM 1351 O GLY A 257 -9.348 -0.162 2.042 1.00 -0.39 O
ATOM 1352 HA3 GLY A 257 -8.266 1.469 0.412 1.00 0.08 H
ATOM 1353 HA2 GLY A 257 -9.912 1.241 -0.208 1.00 0.08 H
ATOM 1354 H GLY A 257 -7.465 0.644 -1.613 1.00 0.19 H
ATOM 1355 N VAL A 258 -9.101 -1.692 0.419 1.00 -0.26 N
ATOM 1356 CA VAL A 258 -9.517 -2.789 1.292 1.00 0.13 C
ATOM 1357 C VAL A 258 -8.448 -3.073 2.333 1.00 0.20 C
ATOM 1358 O VAL A 258 -7.302 -3.255 1.945 1.00 -0.39 O
ATOM 1359 CB VAL A 258 -9.855 -4.097 0.511 1.00 -0.01 C
ATOM 1360 CG1 VAL A 258 -10.363 -5.204 1.479 1.00 -0.06 C
ATOM 1361 CG2 VAL A 258 -10.924 -3.854 -0.594 1.00 -0.06 C

ATOM 1362 HA VAL A 258 -10.453 -2.475 1.780 1.00 0.08 H
ATOM 1363 HB VAL A 258 -8.935 -4.460 0.019 1.00 0.03 H
ATOM 1364 HG11 VAL A 258 -11.289 -4.880 1.979 1.00 0.02 H
ATOM 1365 HG12 VAL A 258 -10.575 -6.130 0.921 1.00 0.02 H
ATOM 1366 HG13 VAL A 258 -9.611 -5.438 2.248 1.00 0.02 H
ATOM 1367 HG21 VAL A 258 -10.578 -3.136 -1.349 1.00 0.02 H
ATOM 1368 HG22 VAL A 258 -11.147 -4.796 -1.121 1.00 0.02 H
ATOM 1369 HG23 VAL A 258 -11.857 -3.468 -0.156 1.00 0.02 H
ATOM 1370 H VAL A 258 -8.832 -1.905 -0.515 1.00 0.19 H
ATOM 1371 N THR A 259 -8.795 -3.130 3.643 1.00 -0.26 N
ATOM 1372 CA THR A 259 -7.794 -3.359 4.686 1.00 0.16 C
ATOM 1373 C THR A 259 -7.809 -4.795 5.166 1.00 0.21 C
ATOM 1374 O THR A 259 -8.887 -5.327 5.367 1.00 -0.39 O
ATOM 1375 CB THR A 259 -8.083 -2.468 5.930 1.00 0.09 C
ATOM 1376 OG1 THR A 259 -8.187 -1.080 5.539 1.00 -0.39 O
ATOM 1377 CG2 THR A 259 -6.993 -2.617 7.024 1.00 -0.03 C
ATOM 1378 HA THR A 259 -6.802 -3.086 4.317 1.00 0.08 H
ATOM 1379 HB THR A 259 -9.040 -2.815 6.361 1.00 0.06 H
ATOM 1380 HG1 THR A 259 -8.630 -0.572 6.223 1.00 0.21 H
ATOM 1381 HG23 THR A 259 -6.925 -3.650 7.395 1.00 0.03 H
ATOM 1382 HG21 THR A 259 -6.013 -2.320 6.625 1.00 0.03 H
ATOM 1383 HG22 THR A 259 -7.235 -1.969 7.879 1.00 0.03 H
ATOM 1384 H THR A 259 -9.745 -3.037 3.936 1.00 0.19 H
ATOM 1385 N ASN A 260 -6.652 -5.447 5.405 1.00 -0.26 N
ATOM 1386 CA ASN A 260 -6.663 -6.751 6.066 1.00 0.15 C
ATOM 1387 C ASN A 260 -5.303 -7.040 6.669 1.00 0.21 C
ATOM 1388 O ASN A 260 -4.354 -6.354 6.318 1.00 -0.39 O
ATOM 1389 CB ASN A 260 -7.144 -7.884 5.128 1.00 0.08 C
ATOM 1390 CG ASN A 260 -6.296 -7.955 3.886 1.00 0.18 C
ATOM 1391 OD1 ASN A 260 -5.085 -8.073 4.008 1.00 -0.40 O
ATOM 1392 ND2 ASN A 260 -6.873 -7.902 2.670 1.00 -0.30 N
ATOM 1393 HA ASN A 260 -7.350 -6.698 6.920 1.00 0.08 H
ATOM 1394 HB2 ASN A 260 -7.077 -8.859 5.635 1.00 0.06 H
ATOM 1395 HB3 ASN A 260 -8.200 -7.721 4.868 1.00 0.06 H
ATOM 1396 HD22 ASN A 260 -6.296 -7.984 1.860 1.00 0.18 H
ATOM 1397 HD21 ASN A 260 -7.859 -7.801 2.546 1.00 0.18 H
ATOM 1398 H ASN A 260 -5.758 -5.049 5.206 1.00 0.19 H
ATOM 1399 N TRP A 261 -5.167 -8.020 7.590 1.00 -0.26 N
ATOM 1400 CA TRP A 261 -3.860 -8.232 8.208 1.00 0.14 C
ATOM 1401 C TRP A 261 -2.809 -8.596 7.173 1.00 0.20 C
ATOM 1402 O TRP A 261 -1.715 -8.056 7.234 1.00 -0.39 O
ATOM 1403 CB TRP A 261 -3.878 -9.305 9.328 1.00 0.00 C
ATOM 1404 CG TRP A 261 -4.531 -8.843 10.600 1.00 -0.04 C
ATOM 1405 CD1 TRP A 261 -5.631 -9.322 11.195 1.00 0.02 C
ATOM 1406 CD2 TRP A 261 -4.005 -7.720 11.472 1.00 -0.02 C
ATOM 1407 NE1 TRP A 261 -5.859 -8.648 12.299 1.00 -0.29 N
ATOM 1408 CE2 TRP A 261 -4.936 -7.697 12.497 1.00 0.06 C
ATOM 1409 CE3 TRP A 261 -2.923 -6.862 11.423 1.00 -0.07 C
ATOM 1410 CZ2 TRP A 261 -4.833 -6.772 13.543 1.00 -0.04 C
ATOM 1411 CZ3 TRP A 261 -2.822 -5.906 12.451 1.00 -0.08 C
ATOM 1412 CH2 TRP A 261 -3.756 -5.878 13.497 1.00 -0.08 C

```
ATOM   1413  HA  TRP A 261      -3.554  -7.284   8.666  1.00  0.08           H
ATOM   1414  HB2 TRP A 261      -4.330 -10.238   8.955  1.00  0.04           H
ATOM   1415  HB3 TRP A 261      -2.841  -9.508   9.623  1.00  0.04           H
ATOM   1416  HE1 TRP A 261      -6.645  -8.836  12.941  1.00  0.22           H
ATOM   1417  HD1 TRP A 261      -6.242 -10.146  10.818  1.00  0.08           H
ATOM   1418  HZ2 TRP A 261      -5.559  -6.759  14.352  1.00  0.05           H
ATOM   1419  HH2 TRP A 261      -3.640  -5.149  14.294  1.00  0.05           H
ATOM   1420  HZ3 TRP A 261      -2.008  -5.191  12.436  1.00  0.05           H
ATOM   1421  HE3 TRP A 261      -2.174  -6.922  10.638  1.00  0.05           H
ATOM   1422  H   TRP A 261      -5.941  -8.577   7.893  1.00  0.19           H
ATOM   1423  N   GLY A 262      -3.090  -9.509   6.216  1.00 -0.27           N
ATOM   1424  CA  GLY A 262      -2.070  -9.851   5.221  1.00  0.12           C
ATOM   1425  C   GLY A 262      -1.496  -8.619   4.567  1.00  0.20           C
ATOM   1426  O   GLY A 262      -0.288  -8.484   4.447  1.00 -0.39           O
ATOM   1427  HA3 GLY A 262      -1.247 -10.388   5.709  1.00  0.08           H
ATOM   1428  HA2 GLY A 262      -2.477 -10.491   4.423  1.00  0.08           H
ATOM   1429  H   GLY A 262      -3.987  -9.952   6.173  1.00  0.19           H
ATOM   1430  N   ARG A 263      -2.359  -7.686   4.129  1.00 -0.26           N
ATOM   1431  CA  ARG A 263      -1.829  -6.463   3.531  1.00  0.13           C
ATOM   1432  C   ARG A 263      -0.981  -5.676   4.509  1.00  0.20           C
ATOM   1433  O   ARG A 263       0.068  -5.168   4.138  1.00 -0.39           O
ATOM   1434  CB  ARG A 263      -2.996  -5.564   3.051  1.00 -0.01           C
ATOM   1435  CG  ARG A 263      -3.554  -6.094   1.707  1.00 -0.02           C
ATOM   1436  CD  ARG A 263      -4.732  -5.222   1.205  1.00  0.06           C
ATOM   1437  NE  ARG A 263      -5.060  -5.563  -0.180  1.00 -0.27           N
ATOM   1438  CZ  ARG A 263      -5.793  -4.794  -0.947  1.00  0.29           C
ATOM   1439  NH1 ARG A 263      -6.406  -3.731  -0.495  1.00 -0.28           N
ATOM   1440  NH2 ARG A 263      -5.921  -5.092  -2.216  1.00 -0.28           N
ATOM   1441  HA  ARG A 263      -1.176  -6.728   2.683  1.00  0.08           H
ATOM   1442  HB2 ARG A 263      -3.786  -5.519   3.816  1.00  0.03           H
ATOM   1443  HB3 ARG A 263      -2.627  -4.539   2.910  1.00  0.03           H
ATOM   1444  HG2 ARG A 263      -2.749  -6.057   0.954  1.00  0.03           H
ATOM   1445  HG3 ARG A 263      -3.879  -7.142   1.813  1.00  0.03           H
ATOM   1446  HD2 ARG A 263      -5.604  -5.359   1.868  1.00  0.07           H
ATOM   1447  HD3 ARG A 263      -4.388  -4.174   1.260  1.00  0.07           H
ATOM   1448  HE  ARG A 263      -4.627  -6.408  -0.587  1.00  0.26           H
ATOM   1449 HH12 ARG A 263      -6.931  -3.130  -1.151  1.00  0.26           H
ATOM   1450 HH11 ARG A 263      -6.423  -3.479   0.502  1.00  0.26           H
ATOM   1451 HH22 ARG A 263      -6.493  -4.494  -2.836  1.00  0.26           H
ATOM   1452 HH21 ARG A 263      -5.467  -5.913  -2.631  1.00  0.26           H
ATOM   1453  H   ARG A 263      -3.352  -7.819   4.232  1.00  0.19           H
ATOM   1454  N   ILE A 264      -1.429  -5.555   5.775  1.00 -0.26           N
ATOM   1455  CA  ILE A 264      -0.612  -4.837   6.752  1.00  0.13           C
ATOM   1456  C   ILE A 264       0.709  -5.561   6.891  1.00  0.20           C
ATOM   1457  O   ILE A 264       1.747  -4.921   6.961  1.00 -0.39           O
ATOM   1458  CB  ILE A 264      -1.355  -4.699   8.114  1.00 -0.00           C
ATOM   1459  CG1 ILE A 264      -2.593  -3.765   7.939  1.00 -0.05           C
ATOM   1460  CG2 ILE A 264      -0.385  -4.200   9.222  1.00 -0.06           C
ATOM   1461  CD1 ILE A 264      -3.603  -3.841   9.112  1.00 -0.06           C
ATOM   1462  HA  ILE A 264      -0.395  -3.827   6.369  1.00  0.08           H
ATOM   1463  HB  ILE A 264      -1.697  -5.702   8.410  1.00  0.03           H
```

ATOM 1464 HG12 ILE A 264 -2.256 -2.728 7.803 1.00 0.03 H
ATOM 1465 HG13 ILE A 264 -3.148 -4.044 7.030 1.00 0.03 H
ATOM 1466 HD11 ILE A 264 -3.154 -3.515 10.057 1.00 0.02 H
ATOM 1467 HD12 ILE A 264 -4.474 -3.197 8.914 1.00 0.02 H
ATOM 1468 HD13 ILE A 264 -3.957 -4.875 9.230 1.00 0.02 H
ATOM 1469 HG21 ILE A 264 0.074 -3.245 8.929 1.00 0.02 H
ATOM 1470 HG22 ILE A 264 -0.897 -4.066 10.184 1.00 0.02 H
ATOM 1471 HG23 ILE A 264 0.418 -4.934 9.388 1.00 0.02 H
ATOM 1472 H ILE A 264 -2.279 -6.013 6.059 1.00 0.19 H
ATOM 1473 N VAL A 265 0.707 -6.909 6.924 1.00 -0.26 N
ATOM 1474 CA VAL A 265 1.985 -7.615 6.964 1.00 0.13 C
ATOM 1475 C VAL A 265 2.787 -7.244 5.737 1.00 0.20 C
ATOM 1476 O VAL A 265 3.935 -6.850 5.877 1.00 -0.39 O
ATOM 1477 CB VAL A 265 1.808 -9.158 7.051 1.00 -0.01 C
ATOM 1478 CG1 VAL A 265 3.151 -9.900 6.823 1.00 -0.06 C
ATOM 1479 CG2 VAL A 265 1.240 -9.567 8.430 1.00 -0.06 C
ATOM 1480 HA VAL A 265 2.549 -7.279 7.849 1.00 0.08 H
ATOM 1481 HB VAL A 265 1.095 -9.490 6.277 1.00 0.03 H
ATOM 1482 HG11 VAL A 265 3.953 -9.452 7.428 1.00 0.02 H
ATOM 1483 HG12 VAL A 265 3.074 -10.966 7.079 1.00 0.02 H
ATOM 1484 HG13 VAL A 265 3.403 -9.830 5.759 1.00 0.02 H
ATOM 1485 HG21 VAL A 265 0.284 -9.056 8.626 1.00 0.02 H
ATOM 1486 HG22 VAL A 265 1.070 -10.656 8.441 1.00 0.02 H
ATOM 1487 HG23 VAL A 265 1.958 -9.303 9.225 1.00 0.02 H
ATOM 1488 H VAL A 265 -0.152 -7.430 6.871 1.00 0.19 H
ATOM 1489 N THR A 266 2.216 -7.356 4.519 1.00 -0.26 N
ATOM 1490 CA THR A 266 3.003 -7.040 3.333 1.00 0.16 C
ATOM 1491 C THR A 266 3.631 -5.668 3.426 1.00 0.21 C
ATOM 1492 O THR A 266 4.798 -5.516 3.091 1.00 -0.39 O
ATOM 1493 CB THR A 266 2.172 -7.210 2.037 1.00 0.09 C
ATOM 1494 OG1 THR A 266 1.739 -8.582 1.979 1.00 -0.39 O
ATOM 1495 CG2 THR A 266 2.992 -6.889 0.768 1.00 -0.03 C
ATOM 1496 HA THR A 266 3.816 -7.769 3.309 1.00 0.08 H
ATOM 1497 HB THR A 266 1.310 -6.527 2.075 1.00 0.06 H
ATOM 1498 HG1 THR A 266 1.188 -8.788 1.228 1.00 0.21 H
ATOM 1499 HG23 THR A 266 3.390 -5.868 0.816 1.00 0.03 H
ATOM 1500 HG21 THR A 266 3.827 -7.591 0.663 1.00 0.03 H
ATOM 1501 HG22 THR A 266 2.349 -6.981 -0.119 1.00 0.03 H
ATOM 1502 H THR A 266 1.272 -7.684 4.410 1.00 0.19 H
ATOM 1503 N LEU A 267 2.890 -4.646 3.901 1.00 -0.26 N
ATOM 1504 CA LEU A 267 3.513 -3.333 4.082 1.00 0.13 C
ATOM 1505 C LEU A 267 4.753 -3.433 4.949 1.00 0.20 C
ATOM 1506 O LEU A 267 5.783 -2.852 4.638 1.00 -0.39 O
ATOM 1507 CB LEU A 267 2.504 -2.334 4.717 1.00 -0.01 C
ATOM 1508 CG LEU A 267 3.081 -0.914 4.998 1.00 -0.04 C
ATOM 1509 CD1 LEU A 267 3.467 -0.160 3.695 1.00 -0.06 C
ATOM 1510 CD2 LEU A 267 2.043 -0.077 5.802 1.00 -0.06 C
ATOM 1511 HA LEU A 267 3.813 -2.957 3.099 1.00 0.08 H
ATOM 1512 HB2 LEU A 267 1.622 -2.246 4.061 1.00 0.03 H
ATOM 1513 HB3 LEU A 267 2.173 -2.758 5.678 1.00 0.03 H
ATOM 1514 HG LEU A 267 3.987 -1.021 5.618 1.00 0.03 H

```
ATOM   1515 HD21 LEU A 267       1.834  -0.560   6.771  1.00  0.02           H
ATOM   1516 HD22 LEU A 267       2.417   0.941   5.996  1.00  0.02           H
ATOM   1517 HD23 LEU A 267       1.101   0.003   5.239  1.00  0.02           H
ATOM   1518 HD11 LEU A 267       2.597  -0.083   3.022  1.00  0.02           H
ATOM   1519 HD12 LEU A 267       3.809   0.856   3.950  1.00  0.02           H
ATOM   1520 HD13 LEU A 267       4.289  -0.666   3.165  1.00  0.02           H
ATOM   1521  H   LEU A 267       1.925  -4.784   4.154  1.00  0.19           H
ATOM   1522  N   ILE A 268       4.671  -4.173   6.070  1.00 -0.26           N
ATOM   1523  CA  ILE A 268       5.812  -4.248   6.986  1.00  0.13           C
ATOM   1524  C   ILE A 268       6.927  -5.056   6.350  1.00  0.20           C
ATOM   1525  O   ILE A 268       8.087  -4.671   6.409  1.00 -0.39           O
ATOM   1526  CB  ILE A 268       5.336  -4.845   8.345  1.00 -0.00           C
ATOM   1527  CG1 ILE A 268       4.335  -3.855   9.016  1.00 -0.05           C
ATOM   1528  CG2 ILE A 268       6.539  -5.167   9.269  1.00 -0.06           C
ATOM   1529  CD1 ILE A 268       3.467  -4.513  10.118  1.00 -0.06           C
ATOM   1530  HA  ILE A 268       6.207  -3.237   7.173  1.00  0.08           H
ATOM   1531  HB  ILE A 268       4.818  -5.800   8.146  1.00  0.03           H
ATOM   1532 HG12 ILE A 268       4.879  -2.992   9.432  1.00  0.03           H
ATOM   1533 HG13 ILE A 268       3.631  -3.464   8.264  1.00  0.03           H
ATOM   1534 HD11 ILE A 268       4.070  -4.811  10.988  1.00  0.02           H
ATOM   1535 HD12 ILE A 268       2.706  -3.787  10.446  1.00  0.02           H
ATOM   1536 HD13 ILE A 268       2.951  -5.402   9.723  1.00  0.02           H
ATOM   1537 HG21 ILE A 268       7.155  -4.271   9.437  1.00  0.02           H
ATOM   1538 HG22 ILE A 268       6.201  -5.551  10.242  1.00  0.02           H
ATOM   1539 HG23 ILE A 268       7.158  -5.944   8.798  1.00  0.02           H
ATOM   1540  H   ILE A 268       3.839  -4.709   6.255  1.00  0.19           H
ATOM   1541  N   SER A 269       6.582  -6.197   5.724  1.00 -0.26           N
ATOM   1542  CA  SER A 269       7.592  -6.987   5.022  1.00  0.15           C
ATOM   1543  C   SER A 269       8.359  -6.157   4.021  1.00  0.21           C
ATOM   1544  O   SER A 269       9.570  -6.293   3.919  1.00 -0.39           O
ATOM   1545  CB  SER A 269       6.854  -8.109   4.259  1.00  0.08           C
ATOM   1546  OG  SER A 269       6.012  -8.741   5.228  1.00 -0.39           O
ATOM   1547  HA  SER A 269       8.284  -7.430   5.754  1.00  0.08           H
ATOM   1548  HB2 SER A 269       6.254  -7.651   3.458  1.00  0.06           H
ATOM   1549  HB3 SER A 269       7.545  -8.828   3.801  1.00  0.06           H
ATOM   1550  HG  SER A 269       5.393  -9.344   4.820  1.00  0.21           H
ATOM   1551  H   SER A 269       5.627  -6.518   5.728  1.00  0.19           H
ATOM   1552  N   PHE A 270       7.669  -5.282   3.260  1.00 -0.26           N
ATOM   1553  CA  PHE A 270       8.417  -4.408   2.362  1.00  0.14           C
ATOM   1554  C   PHE A 270       9.342  -3.541   3.192  1.00  0.21           C
ATOM   1555  O   PHE A 270      10.496  -3.367   2.831  1.00 -0.39           O
ATOM   1556  CB  PHE A 270       7.480  -3.552   1.480  1.00  0.02           C
ATOM   1557  CG  PHE A 270       8.308  -2.689   0.523  1.00 -0.04           C
ATOM   1558  CD1 PHE A 270       8.941  -3.278  -0.580  1.00 -0.06           C
ATOM   1559  CD2 PHE A 270       8.439  -1.309   0.728  1.00 -0.06           C
ATOM   1560  CE1 PHE A 270       9.664  -2.499  -1.480  1.00 -0.07           C
ATOM   1561  CE2 PHE A 270       9.110  -0.520  -0.206  1.00 -0.07           C
ATOM   1562  CZ  PHE A 270       9.715  -1.110  -1.321  1.00 -0.07           C
ATOM   1563  HA  PHE A 270       9.026  -5.038   1.697  1.00  0.08           H
ATOM   1564  HB2 PHE A 270       6.833  -4.222   0.890  1.00  0.05           H
ATOM   1565  HB3 PHE A 270       6.831  -2.927   2.116  1.00  0.05           H
```

ATOM 1566 HD2 PHE A 270 8.017 -0.849 1.618 1.00 0.06 H
ATOM 1567 HE2 PHE A 270 9.174 0.558 -0.070 1.00 0.06 H
ATOM 1568 HZ PHE A 270 10.225 -0.498 -2.052 1.00 0.06 H
ATOM 1569 HE1 PHE A 270 10.188 -2.963 -2.309 1.00 0.06 H
ATOM 1570 HD1 PHE A 270 8.862 -4.346 -0.739 1.00 0.06 H
ATOM 1571 H PHE A 270 6.668 -5.184 3.334 1.00 0.19 H
ATOM 1572 N GLY A 271 8.883 -2.992 4.337 1.00 -0.27 N
ATOM 1573 CA GLY A 271 9.816 -2.267 5.197 1.00 0.12 C
ATOM 1574 C GLY A 271 11.045 -3.084 5.515 1.00 0.20 C
ATOM 1575 O GLY A 271 12.155 -2.607 5.340 1.00 -0.39 O
ATOM 1576 HA3 GLY A 271 10.116 -1.330 4.704 1.00 0.08 H
ATOM 1577 HA2 GLY A 271 9.356 -2.037 6.167 1.00 0.08 H
ATOM 1578 H GLY A 271 7.924 -3.099 4.623 1.00 0.19 H
ATOM 1579 N ALA A 272 10.880 -4.334 5.992 1.00 -0.26 N
ATOM 1580 CA ALA A 272 12.062 -5.162 6.240 1.00 0.13 C
ATOM 1581 C ALA A 272 12.929 -5.254 5.003 1.00 0.20 C
ATOM 1582 O ALA A 272 14.145 -5.154 5.092 1.00 -0.39 O
ATOM 1583 CB ALA A 272 11.656 -6.595 6.657 1.00 -0.02 C
ATOM 1584 HA ALA A 272 12.665 -4.711 7.043 1.00 0.08 H
ATOM 1585 HB1 ALA A 272 12.548 -7.230 6.748 1.00 0.03 H
ATOM 1586 HB2 ALA A 272 11.137 -6.551 7.624 1.00 0.03 H
ATOM 1587 HB3 ALA A 272 10.988 -7.052 5.914 1.00 0.03 H
ATOM 1588 H ALA A 272 9.953 -4.698 6.143 1.00 0.19 H
ATOM 1589 N PHE A 273 12.303 -5.449 3.826 1.00 -0.26 N
ATOM 1590 CA PHE A 273 13.087 -5.506 2.594 1.00 0.14 C
ATOM 1591 C PHE A 273 13.864 -4.222 2.386 1.00 0.21 C
ATOM 1592 O PHE A 273 15.043 -4.264 2.068 1.00 -0.39 O
ATOM 1593 CB PHE A 273 12.134 -5.759 1.400 1.00 0.02 C
ATOM 1594 CG PHE A 273 12.907 -5.972 0.102 1.00 -0.04 C
ATOM 1595 CD1 PHE A 273 13.387 -7.246 -0.223 1.00 -0.06 C
ATOM 1596 CD2 PHE A 273 13.124 -4.897 -0.770 1.00 -0.06 C
ATOM 1597 CE1 PHE A 273 14.033 -7.459 -1.439 1.00 -0.07 C
ATOM 1598 CE2 PHE A 273 13.749 -5.114 -1.996 1.00 -0.07 C
ATOM 1599 CZ PHE A 273 14.210 -6.392 -2.325 1.00 -0.07 C
ATOM 1600 HA PHE A 273 13.800 -6.342 2.661 1.00 0.08 H
ATOM 1601 HB2 PHE A 273 11.528 -6.651 1.612 1.00 0.05 H
ATOM 1602 HB3 PHE A 273 11.444 -4.915 1.260 1.00 0.05 H
ATOM 1603 HD2 PHE A 273 12.803 -3.894 -0.500 1.00 0.06 H
ATOM 1604 HE2 PHE A 273 13.877 -4.293 -2.697 1.00 0.06 H
ATOM 1605 HZ PHE A 273 14.708 -6.555 -3.265 1.00 0.06 H
ATOM 1606 HE1 PHE A 273 14.399 -8.449 -1.701 1.00 0.06 H
ATOM 1607 HD1 PHE A 273 13.258 -8.074 0.469 1.00 0.06 H
ATOM 1608 H PHE A 273 11.298 -5.531 3.790 1.00 0.19 H
ATOM 1609 N VAL A 274 13.219 -3.050 2.568 1.00 -0.26 N
ATOM 1610 CA VAL A 274 13.952 -1.795 2.400 1.00 0.13 C
ATOM 1611 C VAL A 274 15.020 -1.696 3.476 1.00 0.20 C
ATOM 1612 O VAL A 274 16.135 -1.282 3.191 1.00 -0.39 O
ATOM 1613 CB VAL A 274 13.000 -0.562 2.439 1.00 -0.01 C
ATOM 1614 CG1 VAL A 274 13.787 0.772 2.338 1.00 -0.06 C
ATOM 1615 CG2 VAL A 274 11.953 -0.613 1.291 1.00 -0.06 C
ATOM 1616 HA VAL A 274 14.455 -1.803 1.421 1.00 0.08 H

ATOM	1617	HB	VAL	A	274	12.460	-0.563	3.402	1.00	0.03	H
ATOM	1618	HG11	VAL	A	274	14.377	0.808	1.409	1.00	0.02	H
ATOM	1619	HG12	VAL	A	274	13.091	1.625	2.345	1.00	0.02	H
ATOM	1620	HG13	VAL	A	274	14.465	0.885	3.194	1.00	0.02	H
ATOM	1621	HG21	VAL	A	274	11.314	-1.502	1.359	1.00	0.02	H
ATOM	1622	HG22	VAL	A	274	11.303	0.274	1.342	1.00	0.02	H
ATOM	1623	HG23	VAL	A	274	12.444	-0.628	0.309	1.00	0.02	H
ATOM	1624	H	VAL	A	274	12.259	-3.041	2.871	1.00	0.19	H
ATOM	1625	N	ALA	A	275	14.717	-2.079	4.736	1.00	-0.26	N
ATOM	1626	CA	ALA	A	275	15.723	-1.970	5.792	1.00	0.13	C
ATOM	1627	C	ALA	A	275	16.981	-2.737	5.433	1.00	0.20	C
ATOM	1628	O	ALA	A	275	18.079	-2.234	5.626	1.00	-0.39	O
ATOM	1629	CB	ALA	A	275	15.164	-2.477	7.146	1.00	-0.02	C
ATOM	1630	HA	ALA	A	275	15.988	-0.908	5.892	1.00	0.08	H
ATOM	1631	HB1	ALA	A	275	15.838	-2.211	7.972	1.00	0.03	H
ATOM	1632	HB2	ALA	A	275	14.192	-2.015	7.349	1.00	0.03	H
ATOM	1633	HB3	ALA	A	275	15.032	-3.569	7.130	1.00	0.03	H
ATOM	1634	H	ALA	A	275	13.802	-2.429	4.956	1.00	0.19	H
ATOM	1635	N	LYS	A	276	16.845	-3.968	4.893	1.00	-0.26	N
ATOM	1636	CA	LYS	A	276	18.037	-4.689	4.440	1.00	0.13	C
ATOM	1637	C	LYS	A	276	18.782	-3.857	3.412	1.00	0.20	C
ATOM	1638	O	LYS	A	276	19.993	-3.716	3.508	1.00	-0.39	O
ATOM	1639	CB	LYS	A	276	17.659	-6.061	3.811	1.00	-0.01	C
ATOM	1640	CG	LYS	A	276	17.232	-7.094	4.894	1.00	-0.04	C
ATOM	1641	CD	LYS	A	276	16.425	-8.279	4.278	1.00	-0.01	C
ATOM	1642	CE	LYS	A	276	16.179	-9.416	5.319	1.00	-0.04	C
ATOM	1643	NZ	LYS	A	276	14.809	-9.996	5.217	1.00	0.22	N
ATOM	1644	HA	LYS	A	276	18.718	-4.853	5.291	1.00	0.08	H
ATOM	1645	HB2	LYS	A	276	16.842	-5.905	3.087	1.00	0.03	H
ATOM	1646	HB3	LYS	A	276	18.523	-6.472	3.259	1.00	0.03	H
ATOM	1647	HG2	LYS	A	276	18.135	-7.483	5.399	1.00	0.03	H
ATOM	1648	HG3	LYS	A	276	16.590	-6.610	5.653	1.00	0.03	H
ATOM	1649	HD2	LYS	A	276	15.455	-7.879	3.928	1.00	0.03	H
ATOM	1650	HD3	LYS	A	276	16.963	-8.681	3.398	1.00	0.03	H
ATOM	1651	HE2	LYS	A	276	16.938	-10.208	5.186	1.00	0.08	H
ATOM	1652	HE3	LYS	A	276	16.300	-9.021	6.341	1.00	0.08	H
ATOM	1653	HZ1	LYS	A	276	14.501	-10.335	4.239	1.00	0.20	H
ATOM	1654	HZ2	LYS	A	276	14.572	-10.745	5.962	1.00	0.20	H
ATOM	1655	HZ3	LYS	A	276	14.042	-9.267	5.439	1.00	0.20	H
ATOM	1656	H	LYS	A	276	15.929	-4.365	4.758	1.00	0.19	H
ATOM	1657	N	HIS	A	277	18.081	-3.289	2.407	1.00	-0.26	N
ATOM	1658	CA	HIS	A	277	18.789	-2.467	1.428	1.00	0.14	C
ATOM	1659	C	HIS	A	277	19.524	-1.335	2.112	1.00	0.21	C
ATOM	1660	O	HIS	A	277	20.690	-1.112	1.822	1.00	-0.39	O
ATOM	1661	CB	HIS	A	277	17.832	-1.870	0.368	1.00	0.04	C
ATOM	1662	CG	HIS	A	277	18.568	-0.920	-0.529	1.00	0.06	C
ATOM	1663	ND1	HIS	A	277	19.436	-1.288	-1.445	1.00	-0.26	N
ATOM	1664	CD2	HIS	A	277	18.448	0.428	-0.527	1.00	-0.03	C
ATOM	1665	CE1	HIS	A	277	19.898	-0.249	-2.055	1.00	0.09	C
ATOM	1666	NE2	HIS	A	277	19.371	0.781	-1.573	1.00	-0.34	N
ATOM	1667	HA	HIS	A	277	19.531	-3.097	0.917	1.00	0.08	H

ATOM	1668	HB2	HIS	A	277	17.381	-2.679	-0.227	1.00	0.05	H
ATOM	1669	HB3	HIS	A	277	17.017	-1.327	0.868	1.00	0.05	H
ATOM	1670	HD2	HIS	A	277	17.827	1.070	0.090	1.00	0.03	H
ATOM	1671	HE1	HIS	A	277	20.632	-0.229	-2.870	1.00	0.11	H
ATOM	1672	HD1	HIS	A	277	19.712	-2.256	-1.654	1.00	0.24	H
ATOM	1673	H	HIS	A	277	17.083	-3.401	2.346	1.00	0.19	H
ATOM	1674	N	LEU	A	278	18.855	-0.599	3.022	1.00	-0.26	N
ATOM	1675	CA	LEU	A	278	19.534	0.517	3.680	1.00	0.13	C
ATOM	1676	C	LEU	A	278	20.813	0.038	4.326	1.00	0.20	C
ATOM	1677	O	LEU	A	278	21.813	0.739	4.275	1.00	-0.39	O
ATOM	1678	CB	LEU	A	278	18.660	1.190	4.769	1.00	-0.01	C
ATOM	1679	CG	LEU	A	278	17.415	1.931	4.198	1.00	-0.04	C
ATOM	1680	CD1	LEU	A	278	16.493	2.378	5.363	1.00	-0.06	C
ATOM	1681	CD2	LEU	A	278	17.798	3.165	3.334	1.00	-0.06	C
ATOM	1682	HA	LEU	A	278	19.817	1.261	2.924	1.00	0.08	H
ATOM	1683	HB2	LEU	A	278	18.340	0.405	5.470	1.00	0.03	H
ATOM	1684	HB3	LEU	A	278	19.264	1.914	5.339	1.00	0.03	H
ATOM	1685	HG	LEU	A	278	16.839	1.242	3.562	1.00	0.03	H
ATOM	1686	HD21	LEU	A	278	18.250	2.866	2.378	1.00	0.02	H
ATOM	1687	HD22	LEU	A	278	16.895	3.746	3.104	1.00	0.02	H
ATOM	1688	HD23	LEU	A	278	18.498	3.823	3.869	1.00	0.02	H
ATOM	1689	HD11	LEU	A	278	17.005	3.120	5.986	1.00	0.02	H
ATOM	1690	HD12	LEU	A	278	15.559	2.818	4.981	1.00	0.02	H
ATOM	1691	HD13	LEU	A	278	16.237	1.522	6.001	1.00	0.02	H
ATOM	1692	H	LEU	A	278	17.912	-0.835	3.270	1.00	0.19	H
ATOM	1693	N	LYS	A	279	20.815	-1.158	4.948	1.00	-0.26	N
ATOM	1694	CA	LYS	A	279	22.061	-1.652	5.531	1.00	0.13	C
ATOM	1695	C	LYS	A	279	23.051	-1.973	4.433	1.00	0.20	C
ATOM	1696	O	LYS	A	279	24.200	-1.568	4.537	1.00	-0.39	O
ATOM	1697	CB	LYS	A	279	21.757	-2.864	6.451	1.00	-0.01	C
ATOM	1698	CG	LYS	A	279	22.972	-3.347	7.293	1.00	-0.04	C
ATOM	1699	CD	LYS	A	279	23.704	-4.587	6.704	1.00	-0.01	C
ATOM	1700	CE	LYS	A	279	24.793	-5.076	7.706	1.00	-0.04	C
ATOM	1701	NZ	LYS	A	279	25.460	-6.317	7.229	1.00	0.22	N
ATOM	1702	HA	LYS	A	279	22.493	-0.850	6.154	1.00	0.08	H
ATOM	1703	HB2	LYS	A	279	20.994	-2.490	7.149	1.00	0.03	H
ATOM	1704	HB3	LYS	A	279	21.314	-3.694	5.877	1.00	0.03	H
ATOM	1705	HG2	LYS	A	279	23.707	-2.533	7.400	1.00	0.03	H
ATOM	1706	HG3	LYS	A	279	22.608	-3.626	8.299	1.00	0.03	H
ATOM	1707	HD2	LYS	A	279	22.972	-5.401	6.541	1.00	0.03	H
ATOM	1708	HD3	LYS	A	279	24.162	-4.323	5.731	1.00	0.03	H
ATOM	1709	HE2	LYS	A	279	25.537	-4.268	7.850	1.00	0.08	H
ATOM	1710	HE3	LYS	A	279	24.319	-5.271	8.687	1.00	0.08	H
ATOM	1711	HZ1	LYS	A	279	25.959	-6.162	6.282	1.00	0.20	H
ATOM	1712	HZ2	LYS	A	279	26.204	-6.666	7.932	1.00	0.20	H
ATOM	1713	HZ3	LYS	A	279	24.751	-7.123	7.098	1.00	0.20	H
ATOM	1714	H	LYS	A	279	19.984	-1.726	4.984	1.00	0.19	H
ATOM	1715	N	THR	A	280	22.641	-2.686	3.361	1.00	-0.26	N
ATOM	1716	CA	THR	A	280	23.574	-2.923	2.257	1.00	0.16	C
ATOM	1717	C	THR	A	280	24.259	-1.650	1.820	1.00	0.21	C
ATOM	1718	O	THR	A	280	25.445	-1.706	1.534	1.00	-0.39	O

ATOM 1719 CB THR A 280 22.871 -3.558 1.027 1.00 0.09 C
ATOM 1720 OG1 THR A 280 22.455 -4.881 1.408 1.00 -0.39 O
ATOM 1721 CG2 THR A 280 23.791 -3.626 -0.221 1.00 -0.03 C
ATOM 1722 HA THR A 280 24.347 -3.624 2.604 1.00 0.08 H
ATOM 1723 HB THR A 280 21.997 -2.942 0.760 1.00 0.06 H
ATOM 1724 HG1 THR A 280 21.967 -5.335 0.728 1.00 0.21 H
ATOM 1725 HG23 THR A 280 24.034 -2.618 -0.591 1.00 0.03 H
ATOM 1726 HG21 THR A 280 24.725 -4.153 0.020 1.00 0.03 H
ATOM 1727 HG22 THR A 280 23.279 -4.166 -1.031 1.00 0.03 H
ATOM 1728 H THR A 280 21.697 -3.034 3.296 1.00 0.19 H
ATOM 1729 N ILE A 281 23.556 -0.498 1.749 1.00 -0.26 N
ATOM 1730 CA ILE A 281 24.217 0.741 1.327 1.00 0.13 C
ATOM 1731 C ILE A 281 24.741 1.556 2.496 1.00 0.20 C
ATOM 1732 O ILE A 281 24.918 2.753 2.341 1.00 -0.39 O
ATOM 1733 CB ILE A 281 23.336 1.563 0.333 1.00 -0.00 C
ATOM 1734 CG1 ILE A 281 21.992 2.020 0.976 1.00 -0.05 C
ATOM 1735 CG2 ILE A 281 23.080 0.725 -0.952 1.00 -0.06 C
ATOM 1736 CD1 ILE A 281 21.325 3.216 0.249 1.00 -0.06 C
ATOM 1737 HA ILE A 281 25.140 0.514 0.772 1.00 0.08 H
ATOM 1738 HB ILE A 281 23.903 2.464 0.038 1.00 0.03 H
ATOM 1739 HG12 ILE A 281 21.271 1.191 0.971 1.00 0.03 H
ATOM 1740 HG13 ILE A 281 22.172 2.312 2.021 1.00 0.03 H
ATOM 1741 HD11 ILE A 281 21.029 2.946 -0.775 1.00 0.02 H
ATOM 1742 HD12 ILE A 281 20.418 3.515 0.798 1.00 0.02 H
ATOM 1743 HD13 ILE A 281 22.006 4.080 0.205 1.00 0.02 H
ATOM 1744 HG21 ILE A 281 22.508 -0.184 -0.710 1.00 0.02 H
ATOM 1745 HG22 ILE A 281 22.516 1.308 -1.694 1.00 0.02 H
ATOM 1746 HG23 ILE A 281 24.031 0.423 -1.415 1.00 0.02 H
ATOM 1747 H ILE A 281 22.590 -0.477 2.020 1.00 0.19 H
ATOM 1748 N ASN A 282 25.033 0.960 3.674 1.00 -0.26 N
ATOM 1749 CA ASN A 282 25.592 1.740 4.782 1.00 0.15 C
ATOM 1750 C ASN A 282 24.695 2.875 5.231 1.00 0.21 C
ATOM 1751 O ASN A 282 25.211 3.908 5.625 1.00 -0.39 O
ATOM 1752 CB ASN A 282 27.011 2.278 4.458 1.00 0.08 C
ATOM 1753 CG ASN A 282 27.880 1.146 3.982 1.00 0.18 C
ATOM 1754 OD1 ASN A 282 28.287 0.344 4.810 1.00 -0.40 O
ATOM 1755 ND2 ASN A 282 28.193 1.022 2.679 1.00 -0.30 N
ATOM 1756 HA ASN A 282 25.690 1.090 5.668 1.00 0.08 H
ATOM 1757 HB2 ASN A 282 26.966 3.075 3.703 1.00 0.06 H
ATOM 1758 HB3 ASN A 282 27.472 2.704 5.363 1.00 0.06 H
ATOM 1759 HD22 ASN A 282 28.772 0.255 2.401 1.00 0.18 H
ATOM 1760 HD21 ASN A 282 27.866 1.668 1.989 1.00 0.18 H
ATOM 1761 H ASN A 282 24.895 -0.026 3.812 1.00 0.19 H
ATOM 1762 N GLN A 283 23.354 2.715 5.212 1.00 -0.26 N
ATOM 1763 CA GLN A 283 22.466 3.754 5.743 1.00 0.13 C
ATOM 1764 C GLN A 283 21.690 3.175 6.911 1.00 0.20 C
ATOM 1765 O GLN A 283 20.508 3.452 7.046 1.00 -0.39 O
ATOM 1766 CB GLN A 283 21.536 4.266 4.609 1.00 0.00 C
ATOM 1767 CG GLN A 283 22.321 4.944 3.454 1.00 0.04 C
ATOM 1768 CD GLN A 283 22.920 6.281 3.812 1.00 0.17 C
ATOM 1769 OE1 GLN A 283 22.621 6.823 4.866 1.00 -0.40 O

```
ATOM   1770  NE2 GLN A 283      23.769   6.864   2.946  1.00 -0.30           N
ATOM   1771  HA  GLN A 283      23.005   4.621   6.156  1.00  0.08           H
ATOM   1772  HB2 GLN A 283      20.994   3.399   4.203  1.00  0.03           H
ATOM   1773  HB3 GLN A 283      20.799   4.982   5.007  1.00  0.03           H
ATOM   1774  HG2 GLN A 283      23.124   4.276   3.114  1.00  0.05           H
ATOM   1775  HG3 GLN A 283      21.636   5.124   2.611  1.00  0.05           H
ATOM   1776 HE22 GLN A 283      24.164   7.752   3.181  1.00  0.18           H
ATOM   1777 HE21 GLN A 283      24.010   6.426   2.079  1.00  0.18           H
ATOM   1778  H   GLN A 283      22.938   1.870   4.859  1.00  0.19           H
ATOM   1779  N   GLU A 284      22.332   2.367   7.790  1.00 -0.26           N
ATOM   1780  CA  GLU A 284      21.582   1.748   8.886  1.00  0.13           C
ATOM   1781  C   GLU A 284      20.855   2.802   9.699  1.00  0.20           C
ATOM   1782  O   GLU A 284      19.718   2.587  10.091  1.00 -0.39           O
ATOM   1783  CB  GLU A 284      22.459   0.907   9.860  1.00 -0.00           C
ATOM   1784  CG  GLU A 284      23.018  -0.391   9.215  1.00  0.00           C
ATOM   1785  CD  GLU A 284      23.700  -1.309  10.199  1.00  0.04           C
ATOM   1786  OE1 GLU A 284      23.598  -1.061  11.428  1.00 -0.57           O
ATOM   1787  OE2 GLU A 284      24.341  -2.299   9.751  1.00 -0.57           O
ATOM   1788  HA  GLU A 284      20.823   1.090   8.437  1.00  0.08           H
ATOM   1789  HB2 GLU A 284      23.291   1.521  10.244  1.00  0.03           H
ATOM   1790  HB3 GLU A 284      21.831   0.606  10.716  1.00  0.03           H
ATOM   1791  HG2 GLU A 284      22.183  -0.961   8.785  1.00  0.04           H
ATOM   1792  HG3 GLU A 284      23.734  -0.144   8.417  1.00  0.04           H
ATOM   1793  H   GLU A 284      23.309   2.177   7.690  1.00  0.19           H
ATOM   1794  N   SER A 285      21.487   3.966   9.964  1.00 -0.26           N
ATOM   1795  CA  SER A 285      20.789   5.017  10.704  1.00  0.15           C
ATOM   1796  C   SER A 285      19.433   5.312  10.102  1.00  0.21           C
ATOM   1797  O   SER A 285      18.506   5.595  10.846  1.00 -0.39           O
ATOM   1798  CB  SER A 285      21.597   6.337  10.670  1.00  0.08           C
ATOM   1799  OG  SER A 285      21.918   6.621   9.295  1.00 -0.39           O
ATOM   1800  HA  SER A 285      20.636   4.691  11.746  1.00  0.08           H
ATOM   1801  HB2 SER A 285      21.006   7.154  11.119  1.00  0.06           H
ATOM   1802  HB3 SER A 285      22.525   6.210  11.252  1.00  0.06           H
ATOM   1803  HG  SER A 285      22.384   7.445   9.183  1.00  0.21           H
ATOM   1804  H   SER A 285      22.416   4.138   9.635  1.00  0.19           H
ATOM   1805  N   CYS A 286      19.269   5.239   8.762  1.00 -0.26           N
ATOM   1806  CA  CYS A 286      17.959   5.533   8.188  1.00  0.14           C
ATOM   1807  C   CYS A 286      16.948   4.422   8.425  1.00  0.20           C
ATOM   1808  O   CYS A 286      15.852   4.525   7.897  1.00 -0.39           O
ATOM   1809  CB  CYS A 286      18.072   5.889   6.684  1.00  0.03           C
ATOM   1810  SG  CYS A 286      19.263   7.247   6.441  1.00 -0.17           S
ATOM   1811  HA  CYS A 286      17.560   6.433   8.680  1.00  0.08           H
ATOM   1812  HB2 CYS A 286      18.411   5.007   6.124  1.00  0.04           H
ATOM   1813  HB3 CYS A 286      17.106   6.230   6.287  1.00  0.04           H
ATOM   1814  HG  CYS A 286      19.210   7.323   5.435  1.00  0.10           H
ATOM   1815 LPG1 CYS A 286      19.066   7.818   6.750  1.00  0.00          LP
ATOM   1816 LPG2 CYS A 286      19.887   7.079   6.651  1.00  0.00          LP
ATOM   1817  H   CYS A 286      20.016   4.951   8.158  1.00  0.19           H
ATOM   1818  N   ILE A 287      17.235   3.357   9.212  1.00 -0.26           N
ATOM   1819  CA  ILE A 287      16.210   2.350   9.503  1.00  0.13           C
ATOM   1820  C   ILE A 287      15.229   2.840  10.553  1.00  0.20           C
```

ATOM	1821	O	ILE	A	287	14.044	2.572	10.434	1.00	-0.39	O
ATOM	1822	CB	ILE	A	287	16.855	1.006	9.965	1.00	-0.00	C
ATOM	1823	CG1	ILE	A	287	17.701	0.410	8.796	1.00	-0.05	C
ATOM	1824	CG2	ILE	A	287	15.761	0.005	10.443	1.00	-0.06	C
ATOM	1825	CD1	ILE	A	287	18.672	-0.710	9.249	1.00	-0.06	C
ATOM	1826	HA	ILE	A	287	15.640	2.135	8.585	1.00	0.08	H
ATOM	1827	HB	ILE	A	287	17.518	1.212	10.825	1.00	0.03	H
ATOM	1828	HG12	ILE	A	287	17.030	0.010	8.022	1.00	0.03	H
ATOM	1829	HG13	ILE	A	287	18.307	1.201	8.327	1.00	0.03	H
ATOM	1830	HD11	ILE	A	287	18.118	-1.565	9.653	1.00	0.02	H
ATOM	1831	HD12	ILE	A	287	19.267	-1.062	8.392	1.00	0.02	H
ATOM	1832	HD13	ILE	A	287	19.357	-0.337	10.026	1.00	0.02	H
ATOM	1833	HG21	ILE	A	287	15.033	-0.150	9.633	1.00	0.02	H
ATOM	1834	HG22	ILE	A	287	16.184	-0.968	10.728	1.00	0.02	H
ATOM	1835	HG23	ILE	A	287	15.227	0.388	11.326	1.00	0.02	H
ATOM	1836	H	ILE	A	287	18.134	3.243	9.639	1.00	0.19	H
ATOM	1837	N	GLU	A	288	15.646	3.545	11.625	1.00	-0.26	N
ATOM	1838	CA	GLU	A	288	14.655	3.914	12.641	1.00	0.13	C
ATOM	1839	C	GLU	A	288	13.600	4.830	12.024	1.00	0.21	C
ATOM	1840	O	GLU	A	288	12.418	4.561	12.187	1.00	-0.39	O
ATOM	1841	CB	GLU	A	288	15.305	4.440	13.956	1.00	-0.00	C
ATOM	1842	CG	GLU	A	288	14.384	4.276	15.188	1.00	0.00	C
ATOM	1843	CD	GLU	A	288	14.146	2.841	15.583	1.00	0.04	C
ATOM	1844	OE1	GLU	A	288	14.976	1.930	15.298	1.00	-0.57	O
ATOM	1845	OE2	GLU	A	288	13.088	2.596	16.218	1.00	-0.57	O
ATOM	1846	HA	GLU	A	288	14.137	2.984	12.928	1.00	0.08	H
ATOM	1847	HB2	GLU	A	288	16.232	3.883	14.161	1.00	0.03	H
ATOM	1848	HB3	GLU	A	288	15.560	5.506	13.879	1.00	0.03	H
ATOM	1849	HG2	GLU	A	288	14.853	4.759	16.058	1.00	0.04	H
ATOM	1850	HG3	GLU	A	288	13.422	4.775	14.992	1.00	0.04	H
ATOM	1851	H	GLU	A	288	16.609	3.790	11.738	1.00	0.19	H
ATOM	1852	N	PRO	A	289	13.927	5.915	11.271	1.00	-0.25	N
ATOM	1853	CA	PRO	A	289	12.878	6.696	10.618	1.00	0.13	C
ATOM	1854	C	PRO	A	289	12.018	5.868	9.671	1.00	0.20	C
ATOM	1855	O	PRO	A	289	10.834	6.140	9.531	1.00	-0.39	O
ATOM	1856	CB	PRO	A	289	13.750	7.741	9.868	1.00	-0.01	C
ATOM	1857	CG	PRO	A	289	15.082	7.788	10.642	1.00	-0.03	C
ATOM	1858	CD	PRO	A	289	15.304	6.311	11.015	1.00	0.04	C
ATOM	1859	HA	PRO	A	289	12.260	7.187	11.382	1.00	0.08	H
ATOM	1860	HD3	PRO	A	289	16.026	6.242	11.835	1.00	0.05	H
ATOM	1861	HD2	PRO	A	289	15.681	5.755	10.154	1.00	0.05	H
ATOM	1862	HG3	PRO	A	289	14.947	8.378	11.563	1.00	0.03	H
ATOM	1863	HG2	PRO	A	289	15.909	8.223	10.056	1.00	0.03	H
ATOM	1864	HB2	PRO	A	289	13.943	7.408	8.834	1.00	0.03	H
ATOM	1865	HB3	PRO	A	289	13.312	8.746	9.850	1.00	0.03	H
ATOM	1866	N	LEU	A	290	12.589	4.841	8.998	1.00	-0.26	N
ATOM	1867	CA	LEU	A	290	11.788	3.949	8.147	1.00	0.13	C
ATOM	1868	C	LEU	A	290	10.688	3.329	8.977	1.00	0.20	C
ATOM	1869	O	LEU	A	290	9.544	3.288	8.546	1.00	-0.39	O
ATOM	1870	CB	LEU	A	290	12.723	2.880	7.494	1.00	-0.01	C
ATOM	1871	CG	LEU	A	290	12.092	1.753	6.627	1.00	-0.04	C

ATOM	1872	CD1	LEU	A	290	11.621	2.269	5.251	1.00	-0.06	C
ATOM	1873	CD2	LEU	A	290	13.132	0.624	6.367	1.00	-0.06	C
ATOM	1874	HA	LEU	A	290	11.262	4.535	7.385	1.00	0.08	H
ATOM	1875	HB2	LEU	A	290	13.519	3.373	6.917	1.00	0.03	H
ATOM	1876	HB3	LEU	A	290	13.192	2.358	8.327	1.00	0.03	H
ATOM	1877	HG	LEU	A	290	11.221	1.347	7.169	1.00	0.03	H
ATOM	1878	HD21	LEU	A	290	13.637	0.318	7.292	1.00	0.02	H
ATOM	1879	HD22	LEU	A	290	12.640	-0.256	5.935	1.00	0.02	H
ATOM	1880	HD23	LEU	A	290	13.904	0.958	5.661	1.00	0.02	H
ATOM	1881	HD11	LEU	A	290	12.451	2.695	4.668	1.00	0.02	H
ATOM	1882	HD12	LEU	A	290	11.148	1.456	4.681	1.00	0.02	H
ATOM	1883	HD13	LEU	A	290	10.888	3.051	5.414	1.00	0.02	H
ATOM	1884	H	LEU	A	290	13.563	4.646	9.121	1.00	0.19	H
ATOM	1885	N	ALA	A	291	10.998	2.845	10.197	1.00	-0.26	N
ATOM	1886	CA	ALA	A	291	9.932	2.303	11.036	1.00	0.13	C
ATOM	1887	C	ALA	A	291	8.952	3.393	11.426	1.00	0.20	C
ATOM	1888	O	ALA	A	291	7.751	3.165	11.377	1.00	-0.39	O
ATOM	1889	CB	ALA	A	291	10.524	1.641	12.304	1.00	-0.02	C
ATOM	1890	HA	ALA	A	291	9.381	1.538	10.466	1.00	0.08	H
ATOM	1891	HB1	ALA	A	291	9.734	1.141	12.886	1.00	0.03	H
ATOM	1892	HB2	ALA	A	291	11.272	0.897	11.991	1.00	0.03	H
ATOM	1893	HB3	ALA	A	291	11.017	2.391	12.942	1.00	0.03	H
ATOM	1894	H	ALA	A	291	11.939	2.892	10.552	1.00	0.19	H
ATOM	1895	N	GLU	A	292	9.436	4.595	11.820	1.00	-0.26	N
ATOM	1896	CA	GLU	A	292	8.508	5.671	12.183	1.00	0.13	C
ATOM	1897	C	GLU	A	292	7.486	5.884	11.083	1.00	0.20	C
ATOM	1898	O	GLU	A	292	6.290	5.925	11.335	1.00	-0.39	O
ATOM	1899	CB	GLU	A	292	9.175	7.070	12.343	1.00	-0.00	C
ATOM	1900	CG	GLU	A	292	10.205	7.220	13.491	1.00	0.00	C
ATOM	1901	CD	GLU	A	292	10.865	8.583	13.414	1.00	0.04	C
ATOM	1902	OE1	GLU	A	292	10.724	9.296	12.376	1.00	-0.57	O
ATOM	1903	OE2	GLU	A	292	11.539	8.967	14.402	1.00	-0.57	O
ATOM	1904	HA	GLU	A	292	7.981	5.412	13.111	1.00	0.08	H
ATOM	1905	HB2	GLU	A	292	9.686	7.326	11.403	1.00	0.03	H
ATOM	1906	HB3	GLU	A	292	8.384	7.822	12.513	1.00	0.03	H
ATOM	1907	HG2	GLU	A	292	9.690	7.110	14.460	1.00	0.04	H
ATOM	1908	HG3	GLU	A	292	10.988	6.448	13.429	1.00	0.04	H
ATOM	1909	H	GLU	A	292	10.427	4.757	11.854	1.00	0.19	H
ATOM	1910	N	SER	A	293	7.960	6.063	9.833	1.00	-0.26	N
ATOM	1911	CA	SER	A	293	7.035	6.465	8.774	1.00	0.15	C
ATOM	1912	C	SER	A	293	6.067	5.359	8.434	1.00	0.21	C
ATOM	1913	O	SER	A	293	4.892	5.635	8.235	1.00	-0.39	O
ATOM	1914	CB	SER	A	293	7.835	6.952	7.544	1.00	0.08	C
ATOM	1915	OG	SER	A	293	6.971	7.235	6.433	1.00	-0.39	O
ATOM	1916	HA	SER	A	293	6.434	7.318	9.129	1.00	0.08	H
ATOM	1917	HB2	SER	A	293	8.343	7.869	7.866	1.00	0.06	H
ATOM	1918	HB3	SER	A	293	8.609	6.219	7.275	1.00	0.06	H
ATOM	1919	HG	SER	A	293	7.456	7.589	5.687	1.00	0.21	H
ATOM	1920	H	SER	A	293	8.945	5.947	9.636	1.00	0.19	H
ATOM	1921	N	ILE	A	294	6.521	4.088	8.392	1.00	-0.26	N
ATOM	1922	CA	ILE	A	294	5.562	3.010	8.156	1.00	0.13	C

ATOM	1923	C	ILE	A	294	4.525	3.061	9.256	1.00	0.20	C
ATOM	1924	O	ILE	A	294	3.331	3.037	8.989	1.00	-0.39	O
ATOM	1925	CB	ILE	A	294	6.222	1.599	8.106	1.00	-0.00	C
ATOM	1926	CG1	ILE	A	294	7.091	1.510	6.818	1.00	-0.05	C
ATOM	1927	CG2	ILE	A	294	5.149	0.477	8.156	1.00	-0.06	C
ATOM	1928	CD1	ILE	A	294	7.785	0.151	6.591	1.00	-0.06	C
ATOM	1929	HA	ILE	A	294	5.045	3.195	7.205	1.00	0.08	H
ATOM	1930	HB	ILE	A	294	6.873	1.467	8.989	1.00	0.03	H
ATOM	1931	HG12	ILE	A	294	6.496	1.747	5.922	1.00	0.03	H
ATOM	1932	HG13	ILE	A	294	7.894	2.253	6.911	1.00	0.03	H
ATOM	1933	HD11	ILE	A	294	7.074	-0.634	6.296	1.00	0.02	H
ATOM	1934	HD12	ILE	A	294	8.518	0.271	5.777	1.00	0.02	H
ATOM	1935	HD13	ILE	A	294	8.308	-0.154	7.508	1.00	0.02	H
ATOM	1936	HG21	ILE	A	294	4.489	0.576	7.289	1.00	0.02	H
ATOM	1937	HG22	ILE	A	294	5.611	-0.519	8.150	1.00	0.02	H
ATOM	1938	HG23	ILE	A	294	4.532	0.536	9.061	1.00	0.02	H
ATOM	1939	H	ILE	A	294	7.485	3.878	8.583	1.00	0.19	H
ATOM	1940	N	THR	A	295	4.983	3.117	10.521	1.00	-0.26	N
ATOM	1941	CA	THR	A	295	4.026	3.127	11.619	1.00	0.16	C
ATOM	1942	C	THR	A	295	3.096	4.314	11.531	1.00	0.21	C
ATOM	1943	O	THR	A	295	1.897	4.158	11.709	1.00	-0.39	O
ATOM	1944	CB	THR	A	295	4.823	3.165	12.943	1.00	0.09	C
ATOM	1945	OG1	THR	A	295	5.687	2.024	12.853	1.00	-0.39	O
ATOM	1946	CG2	THR	A	295	3.874	3.082	14.165	1.00	-0.03	C
ATOM	1947	HA	THR	A	295	3.445	2.193	11.581	1.00	0.08	H
ATOM	1948	HB	THR	A	295	5.398	4.107	12.995	1.00	0.06	H
ATOM	1949	HG1	THR	A	295	6.504	2.114	13.359	1.00	0.21	H
ATOM	1950	HG23	THR	A	295	3.242	3.977	14.242	1.00	0.03	H
ATOM	1951	HG21	THR	A	295	3.229	2.200	14.056	1.00	0.03	H
ATOM	1952	HG22	THR	A	295	4.457	2.984	15.088	1.00	0.03	H
ATOM	1953	H	THR	A	295	5.967	3.132	10.728	1.00	0.19	H
ATOM	1954	N	ASP	A	296	3.636	5.518	11.260	1.00	-0.26	N
ATOM	1955	CA	ASP	A	296	2.780	6.698	11.170	1.00	0.14	C
ATOM	1956	C	ASP	A	296	1.712	6.482	10.126	1.00	0.21	C
ATOM	1957	O	ASP	A	296	0.534	6.600	10.438	1.00	-0.39	O
ATOM	1958	CB	ASP	A	296	3.624	7.958	10.865	1.00	0.04	C
ATOM	1959	CG	ASP	A	296	2.777	9.194	10.839	1.00	0.04	C
ATOM	1960	OD1	ASP	A	296	2.262	9.630	11.907	1.00	-0.57	O
ATOM	1961	OD2	ASP	A	296	2.573	9.796	9.756	1.00	-0.57	O
ATOM	1962	HA	ASP	A	296	2.257	6.862	12.122	1.00	0.08	H
ATOM	1963	HB2	ASP	A	296	4.409	8.066	11.631	1.00	0.05	H
ATOM	1964	HB3	ASP	A	296	4.104	7.851	9.884	1.00	0.05	H
ATOM	1965	H	ASP	A	296	4.624	5.612	11.104	1.00	0.19	H
ATOM	1966	N	VAL	A	297	2.083	6.152	8.870	1.00	-0.26	N
ATOM	1967	CA	VAL	A	297	1.041	6.039	7.855	1.00	0.13	C
ATOM	1968	C	VAL	A	297	0.138	4.868	8.177	1.00	0.20	C
ATOM	1969	O	VAL	A	297	-1.064	4.984	7.998	1.00	-0.39	O
ATOM	1970	CB	VAL	A	297	1.611	6.010	6.408	1.00	-0.01	C
ATOM	1971	CG1	VAL	A	297	2.155	4.611	6.009	1.00	-0.06	C
ATOM	1972	CG2	VAL	A	297	0.545	6.487	5.384	1.00	-0.06	C
ATOM	1973	HA	VAL	A	297	0.425	6.949	7.940	1.00	0.08	H

ATOM	1974	HB	VAL	A	297	2.447	6.731	6.364	1.00	0.03	H
ATOM	1975	HG11	VAL	A	297	1.347	3.864	5.989	1.00	0.02	H
ATOM	1976	HG12	VAL	A	297	2.622	4.649	5.013	1.00	0.02	H
ATOM	1977	HG13	VAL	A	297	2.917	4.286	6.728	1.00	0.02	H
ATOM	1978	HG21	VAL	A	297	0.153	7.477	5.658	1.00	0.02	H
ATOM	1979	HG22	VAL	A	297	0.993	6.582	4.384	1.00	0.02	H
ATOM	1980	HG23	VAL	A	297	-0.296	5.778	5.347	1.00	0.02	H
ATOM	1981	H	VAL	A	297	3.046	5.977	8.631	1.00	0.19	H
ATOM	1982	N	LEU	A	298	0.676	3.731	8.671	1.00	-0.26	N
ATOM	1983	CA	LEU	A	298	-0.198	2.613	9.035	1.00	0.13	C
ATOM	1984	C	LEU	A	298	-1.194	3.034	10.097	1.00	0.20	C
ATOM	1985	O	LEU	A	298	-2.384	2.788	9.960	1.00	-0.39	O
ATOM	1986	CB	LEU	A	298	0.647	1.425	9.589	1.00	-0.01	C
ATOM	1987	CG	LEU	A	298	-0.174	0.263	10.226	1.00	-0.04	C
ATOM	1988	CD1	LEU	A	298	-1.134	-0.404	9.205	1.00	-0.06	C
ATOM	1989	CD2	LEU	A	298	0.778	-0.805	10.837	1.00	-0.06	C
ATOM	1990	HA	LEU	A	298	-0.750	2.291	8.137	1.00	0.08	H
ATOM	1991	HB2	LEU	A	298	1.286	1.034	8.780	1.00	0.03	H
ATOM	1992	HB3	LEU	A	298	1.306	1.817	10.380	1.00	0.03	H
ATOM	1993	HG	LEU	A	298	-0.774	0.668	11.057	1.00	0.03	H
ATOM	1994	HD21	LEU	A	298	1.399	-0.344	11.623	1.00	0.02	H
ATOM	1995	HD22	LEU	A	298	0.202	-1.627	11.294	1.00	0.02	H
ATOM	1996	HD23	LEU	A	298	1.440	-1.225	10.063	1.00	0.02	H
ATOM	1997	HD11	LEU	A	298	-0.567	-0.790	8.342	1.00	0.02	H
ATOM	1998	HD12	LEU	A	298	-1.670	-1.235	9.689	1.00	0.02	H
ATOM	1999	HD13	LEU	A	298	-1.884	0.316	8.846	1.00	0.02	H
ATOM	2000	H	LEU	A	298	1.669	3.651	8.822	1.00	0.19	H
ATOM	2001	N	VAL	A	299	-0.717	3.654	11.196	1.00	-0.26	N
ATOM	2002	CA	VAL	A	299	-1.617	3.940	12.310	1.00	0.13	C
ATOM	2003	C	VAL	A	299	-2.543	5.077	11.946	1.00	0.20	C
ATOM	2004	O	VAL	A	299	-3.733	4.982	12.207	1.00	-0.39	O
ATOM	2005	CB	VAL	A	299	-0.823	4.270	13.605	1.00	-0.01	C
ATOM	2006	CG1	VAL	A	299	-1.765	4.729	14.754	1.00	-0.06	C
ATOM	2007	CG2	VAL	A	299	-0.014	3.026	14.064	1.00	-0.06	C
ATOM	2008	HA	VAL	A	299	-2.238	3.054	12.511	1.00	0.08	H
ATOM	2009	HB	VAL	A	299	-0.119	5.093	13.386	1.00	0.03	H
ATOM	2010	HG11	VAL	A	299	-2.528	3.963	14.953	1.00	0.02	H
ATOM	2011	HG12	VAL	A	299	-1.190	4.899	15.677	1.00	0.02	H
ATOM	2012	HG13	VAL	A	299	-2.274	5.668	14.491	1.00	0.02	H
ATOM	2013	HG21	VAL	A	299	0.571	2.587	13.244	1.00	0.02	H
ATOM	2014	HG22	VAL	A	299	0.688	3.309	14.857	1.00	0.02	H
ATOM	2015	HG23	VAL	A	299	-0.693	2.252	14.451	1.00	0.02	H
ATOM	2016	H	VAL	A	299	0.251	3.929	11.253	1.00	0.19	H
ATOM	2017	N	ARG	A	300	-2.029	6.176	11.352	1.00	-0.26	N
ATOM	2018	CA	ARG	A	300	-2.925	7.284	11.032	1.00	0.13	C
ATOM	2019	C	ARG	A	300	-4.057	6.823	10.144	1.00	0.20	C
ATOM	2020	O	ARG	A	300	-5.175	7.275	10.339	1.00	-0.39	O
ATOM	2021	CB	ARG	A	300	-2.208	8.445	10.289	1.00	-0.01	C
ATOM	2022	CG	ARG	A	300	-1.288	9.258	11.239	1.00	-0.02	C
ATOM	2023	CD	ARG	A	300	-0.564	10.397	10.479	1.00	0.06	C
ATOM	2024	NE	ARG	A	300	0.289	11.108	11.435	1.00	-0.27	N

ATOM	2025	CZ	ARG	A	300	-0.087	12.073	12.240	1.00	0.29	C
ATOM	2026	NH1	ARG	A	300	-1.315	12.525	12.294	1.00	-0.28	N
ATOM	2027	NH2	ARG	A	300	0.816	12.605	13.029	1.00	-0.28	N
ATOM	2028	HA	ARG	A	300	-3.370	7.664	11.966	1.00	0.08	H
ATOM	2029	HB2	ARG	A	300	-1.627	8.036	9.447	1.00	0.03	H
ATOM	2030	HB3	ARG	A	300	-2.966	9.132	9.875	1.00	0.03	H
ATOM	2031	HG2	ARG	A	300	-1.890	9.692	12.053	1.00	0.03	H
ATOM	2032	HG3	ARG	A	300	-0.527	8.606	11.697	1.00	0.03	H
ATOM	2033	HD2	ARG	A	300	0.065	9.925	9.703	1.00	0.07	H
ATOM	2034	HD3	ARG	A	300	-1.252	11.058	9.934	1.00	0.07	H
ATOM	2035	HE	ARG	A	300	1.275	10.800	11.468	1.00	0.26	H
ATOM	2036	HH12	ARG	A	300	-1.574	13.283	12.937	1.00	0.26	H
ATOM	2037	HH11	ARG	A	300	-2.055	12.137	11.703	1.00	0.26	H
ATOM	2038	HH22	ARG	A	300	0.578	13.366	13.680	1.00	0.26	H
ATOM	2039	HH21	ARG	A	300	1.793	12.273	13.020	1.00	0.26	H
ATOM	2040	H	ARG	A	300	-1.052	6.236	11.113	1.00	0.19	H
ATOM	2041	N	THR	A	301	-3.797	5.950	9.153	1.00	-0.26	N
ATOM	2042	CA	THR	A	301	-4.857	5.598	8.214	1.00	0.16	C
ATOM	2043	C	THR	A	301	-5.675	4.414	8.691	1.00	0.21	C
ATOM	2044	O	THR	A	301	-6.839	4.367	8.325	1.00	-0.39	O
ATOM	2045	CB	THR	A	301	-4.208	5.261	6.851	1.00	0.09	C
ATOM	2046	OG1	THR	A	301	-3.329	4.160	7.128	1.00	-0.39	O
ATOM	2047	CG2	THR	A	301	-3.437	6.483	6.281	1.00	-0.03	C
ATOM	2048	HA	THR	A	301	-5.547	6.445	8.063	1.00	0.08	H
ATOM	2049	HB	THR	A	301	-4.980	4.968	6.120	1.00	0.06	H
ATOM	2050	HG1	THR	A	301	-2.677	3.990	6.457	1.00	0.21	H
ATOM	2051	HG23	THR	A	301	-4.127	7.327	6.128	1.00	0.03	H
ATOM	2052	HG21	THR	A	301	-2.638	6.803	6.967	1.00	0.03	H
ATOM	2053	HG22	THR	A	301	-2.979	6.230	5.312	1.00	0.03	H
ATOM	2054	H	THR	A	301	-2.889	5.533	9.034	1.00	0.19	H
ATOM	2055	N	LYS	A	302	-5.136	3.443	9.472	1.00	-0.26	N
ATOM	2056	CA	LYS	A	302	-5.934	2.275	9.871	1.00	0.13	C
ATOM	2057	C	LYS	A	302	-6.285	2.235	11.351	1.00	0.20	C
ATOM	2058	O	LYS	A	302	-6.696	1.182	11.813	1.00	-0.39	O
ATOM	2059	CB	LYS	A	302	-5.202	0.988	9.398	1.00	-0.01	C
ATOM	2060	CG	LYS	A	302	-4.750	1.038	7.909	1.00	-0.04	C
ATOM	2061	CD	LYS	A	302	-5.936	1.216	6.915	1.00	-0.01	C
ATOM	2062	CE	LYS	A	302	-5.489	1.230	5.424	1.00	-0.04	C
ATOM	2063	NZ	LYS	A	302	-6.660	1.204	4.498	1.00	0.22	N
ATOM	2064	HA	LYS	A	302	-6.930	2.272	9.399	1.00	0.08	H
ATOM	2065	HB2	LYS	A	302	-4.308	0.867	10.027	1.00	0.03	H
ATOM	2066	HB3	LYS	A	302	-5.849	0.110	9.557	1.00	0.03	H
ATOM	2067	HG2	LYS	A	302	-4.028	1.852	7.762	1.00	0.03	H
ATOM	2068	HG3	LYS	A	302	-4.240	0.089	7.668	1.00	0.03	H
ATOM	2069	HD2	LYS	A	302	-6.632	0.381	7.088	1.00	0.03	H
ATOM	2070	HD3	LYS	A	302	-6.467	2.159	7.114	1.00	0.03	H
ATOM	2071	HE2	LYS	A	302	-4.885	2.139	5.260	1.00	0.08	H
ATOM	2072	HE3	LYS	A	302	-4.846	0.355	5.226	1.00	0.08	H
ATOM	2073	HZ1	LYS	A	302	-7.356	1.999	4.711	1.00	0.20	H
ATOM	2074	HZ2	LYS	A	302	-6.361	1.343	3.472	1.00	0.20	H
ATOM	2075	HZ3	LYS	A	302	-7.208	0.272	4.534	1.00	0.20	H

```
ATOM   2076  H   LYS A 302      -4.172   3.485   9.763  1.00  0.19           H
ATOM   2077  N   ARG A 303      -6.164   3.342  12.117  1.00 -0.26           N
ATOM   2078  CA  ARG A 303      -6.500   3.300  13.545  1.00  0.13           C
ATOM   2079  C   ARG A 303      -7.831   2.631  13.853  1.00  0.20           C
ATOM   2080  O   ARG A 303      -7.845   1.694  14.637  1.00 -0.39           O
ATOM   2081  CB  ARG A 303      -6.489   4.747  14.115  1.00 -0.01           C
ATOM   2082  CG  ARG A 303      -6.877   4.825  15.618  1.00 -0.02           C
ATOM   2083  CD  ARG A 303      -6.767   6.284  16.150  1.00  0.06           C
ATOM   2084  NE  ARG A 303      -5.366   6.740  16.162  1.00 -0.27           N
ATOM   2085  CZ  ARG A 303      -4.495   6.441  17.104  1.00  0.29           C
ATOM   2086  NH1 ARG A 303      -4.785   5.671  18.125  1.00 -0.28           N
ATOM   2087  NH2 ARG A 303      -3.281   6.933  17.021  1.00 -0.28           N
ATOM   2088  HA  ARG A 303      -5.712   2.727  14.057  1.00  0.08           H
ATOM   2089  HB2 ARG A 303      -5.477   5.165  13.996  1.00  0.03           H
ATOM   2090  HB3 ARG A 303      -7.180   5.377  13.532  1.00  0.03           H
ATOM   2091  HG2 ARG A 303      -7.917   4.483  15.756  1.00  0.03           H
ATOM   2092  HG3 ARG A 303      -6.223   4.157  16.197  1.00  0.03           H
ATOM   2093  HD2 ARG A 303      -7.351   6.927  15.466  1.00  0.07           H
ATOM   2094  HD3 ARG A 303      -7.254   6.409  17.132  1.00  0.07           H
ATOM   2095  HE  ARG A 303      -5.072   7.358  15.388  1.00  0.26           H
ATOM   2096 HH12 ARG A 303      -4.087   5.463  18.846  1.00  0.26           H
ATOM   2097 HH11 ARG A 303      -5.711   5.254  18.246  1.00  0.26           H
ATOM   2098 HH22 ARG A 303      -2.567   6.720  17.731  1.00  0.26           H
ATOM   2099 HH21 ARG A 303      -3.003   7.548  16.246  1.00  0.26           H
ATOM   2100  H   ARG A 303      -5.784   4.183  11.720  1.00  0.19           H
ATOM   2101  N   ASP A 304      -8.968   3.091  13.286  1.00 -0.26           N
ATOM   2102  CA  ASP A 304     -10.264   2.477  13.612  1.00  0.14           C
ATOM   2103  C   ASP A 304     -10.211   0.981  13.388  1.00  0.21           C
ATOM   2104  O   ASP A 304     -10.597   0.204  14.251  1.00 -0.39           O
ATOM   2105  CB  ASP A 304     -11.384   3.092  12.734  1.00  0.04           C
ATOM   2106  CG  ASP A 304     -12.734   2.508  12.982  1.00  0.04           C
ATOM   2107  OD1 ASP A 304     -13.387   2.865  13.986  1.00 -0.57           O
ATOM   2108  OD2 ASP A 304     -13.187   1.705  12.134  1.00 -0.57           O
ATOM   2109  HA  ASP A 304     -10.510   2.670  14.666  1.00  0.08           H
ATOM   2110  HB2 ASP A 304     -11.470   4.156  12.977  1.00  0.05           H
ATOM   2111  HB3 ASP A 304     -11.144   2.994  11.662  1.00  0.05           H
ATOM   2112  H   ASP A 304      -8.919   3.855  12.643  1.00  0.19           H
ATOM   2113  N   TRP A 305      -9.737   0.566  12.196  1.00 -0.26           N
ATOM   2114  CA  TRP A 305      -9.697  -0.855  11.885  1.00  0.14           C
ATOM   2115  C   TRP A 305      -8.795  -1.561  12.876  1.00  0.21           C
ATOM   2116  O   TRP A 305      -9.175  -2.587  13.418  1.00 -0.39           O
ATOM   2117  CB  TRP A 305      -9.234  -1.029  10.413  1.00  0.00           C
ATOM   2118  CG  TRP A 305      -9.339  -2.438   9.899  1.00 -0.04           C
ATOM   2119  CD1 TRP A 305     -10.339  -2.972   9.176  1.00  0.02           C
ATOM   2120  CD2 TRP A 305      -8.313  -3.520  10.087  1.00 -0.02           C
ATOM   2121  NE1 TRP A 305     -10.065  -4.225   8.906  1.00 -0.29           N
ATOM   2122  CE2 TRP A 305      -8.880  -4.595   9.432  1.00  0.06           C
ATOM   2123  CE3 TRP A 305      -7.069  -3.608  10.718  1.00 -0.07           C
ATOM   2124  CZ2 TRP A 305      -8.254  -5.839   9.383  1.00 -0.04           C
ATOM   2125  CZ3 TRP A 305      -6.431  -4.852  10.682  1.00 -0.08           C
ATOM   2126  CH2 TRP A 305      -6.996  -5.943   9.999  1.00 -0.08           C
```

ATOM 2127 HA TRP A 305 -10.714 -1.260 11.984 1.00 0.08 H
ATOM 2128 HB2 TRP A 305 -9.871 -0.400 9.769 1.00 0.04 H
ATOM 2129 HB3 TRP A 305 -8.194 -0.686 10.294 1.00 0.04 H
ATOM 2130 HE1 TRP A 305 -10.676 -4.852 8.357 1.00 0.22 H
ATOM 2131 HD1 TRP A 305 -11.242 -2.440 8.866 1.00 0.08 H
ATOM 2132 HZ2 TRP A 305 -8.728 -6.687 8.897 1.00 0.05 H
ATOM 2133 HH2 TRP A 305 -6.458 -6.881 9.951 1.00 0.05 H
ATOM 2134 HZ3 TRP A 305 -5.482 -4.980 11.197 1.00 0.05 H
ATOM 2135 HE3 TRP A 305 -6.628 -2.747 11.211 1.00 0.05 H
ATOM 2136 H TRP A 305 -9.388 1.233 11.541 1.00 0.19 H
ATOM 2137 N LEU A 306 -7.580 -1.035 13.145 1.00 -0.26 N
ATOM 2138 CA LEU A 306 -6.710 -1.694 14.119 1.00 0.13 C
ATOM 2139 C LEU A 306 -7.380 -1.791 15.473 1.00 0.20 C
ATOM 2140 O LEU A 306 -7.261 -2.824 16.117 1.00 -0.39 O
ATOM 2141 CB LEU A 306 -5.385 -0.912 14.322 1.00 -0.01 C
ATOM 2142 CG LEU A 306 -4.427 -0.965 13.095 1.00 -0.04 C
ATOM 2143 CD1 LEU A 306 -3.355 0.152 13.209 1.00 -0.06 C
ATOM 2144 CD2 LEU A 306 -3.721 -2.344 12.975 1.00 -0.06 C
ATOM 2145 HA LEU A 306 -6.497 -2.719 13.783 1.00 0.08 H
ATOM 2146 HB2 LEU A 306 -5.659 0.132 14.538 1.00 0.03 H
ATOM 2147 HB3 LEU A 306 -4.852 -1.303 15.204 1.00 0.03 H
ATOM 2148 HG LEU A 306 -4.997 -0.787 12.168 1.00 0.03 H
ATOM 2149 HD21 LEU A 306 -4.445 -3.137 12.746 1.00 0.02 H
ATOM 2150 HD22 LEU A 306 -2.978 -2.322 12.165 1.00 0.02 H
ATOM 2151 HD23 LEU A 306 -3.197 -2.599 13.909 1.00 0.02 H
ATOM 2152 HD11 LEU A 306 -2.737 0.009 14.108 1.00 0.02 H
ATOM 2153 HD12 LEU A 306 -2.702 0.136 12.326 1.00 0.02 H
ATOM 2154 HD13 LEU A 306 -3.835 1.141 13.256 1.00 0.02 H
ATOM 2155 H LEU A 306 -7.297 -0.167 12.723 1.00 0.19 H
ATOM 2156 N VAL A 307 -8.075 -0.736 15.948 1.00 -0.26 N
ATOM 2157 CA VAL A 307 -8.727 -0.853 17.252 1.00 0.13 C
ATOM 2158 C VAL A 307 -9.813 -1.908 17.155 1.00 0.20 C
ATOM 2159 O VAL A 307 -9.863 -2.809 17.981 1.00 -0.39 O
ATOM 2160 CB VAL A 307 -9.310 0.505 17.750 1.00 -0.01 C
ATOM 2161 CG1 VAL A 307 -10.168 0.314 19.034 1.00 -0.06 C
ATOM 2162 CG2 VAL A 307 -8.168 1.522 18.039 1.00 -0.06 C
ATOM 2163 HA VAL A 307 -7.995 -1.200 18.000 1.00 0.08 H
ATOM 2164 HB VAL A 307 -9.962 0.920 16.960 1.00 0.03 H
ATOM 2165 HG11 VAL A 307 -9.566 -0.150 19.831 1.00 0.02 H
ATOM 2166 HG12 VAL A 307 -10.538 1.287 19.395 1.00 0.02 H
ATOM 2167 HG13 VAL A 307 -11.041 -0.326 18.833 1.00 0.02 H
ATOM 2168 HG21 VAL A 307 -7.536 1.678 17.153 1.00 0.02 H
ATOM 2169 HG22 VAL A 307 -8.591 2.497 18.331 1.00 0.02 H
ATOM 2170 HG23 VAL A 307 -7.528 1.160 18.859 1.00 0.02 H
ATOM 2171 H VAL A 307 -8.185 0.104 15.403 1.00 0.19 H
ATOM 2172 N LYS A 308 -10.708 -1.818 16.147 1.00 -0.26 N
ATOM 2173 CA LYS A 308 -11.788 -2.802 16.058 1.00 0.13 C
ATOM 2174 C LYS A 308 -11.243 -4.208 15.999 1.00 0.20 C
ATOM 2175 O LYS A 308 -11.860 -5.104 16.554 1.00 -0.39 O
ATOM 2176 CB LYS A 308 -12.671 -2.589 14.807 1.00 -0.01 C
ATOM 2177 CG LYS A 308 -13.570 -1.341 14.985 1.00 -0.04 C

ATOM	2178	CD	LYS	A	308	-14.273	-0.965	13.654	1.00	-0.01	C
ATOM	2179	CE	LYS	A	308	-15.193	0.273	13.871	1.00	-0.04	C
ATOM	2180	NZ	LYS	A	308	-15.504	0.965	12.599	1.00	0.22	N
ATOM	2181	HA	LYS	A	308	-12.407	-2.732	16.966	1.00	0.08	H
ATOM	2182	HB2	LYS	A	308	-12.020	-2.490	13.927	1.00	0.03	H
ATOM	2183	HB3	LYS	A	308	-13.325	-3.461	14.654	1.00	0.03	H
ATOM	2184	HG2	LYS	A	308	-14.331	-1.531	15.762	1.00	0.03	H
ATOM	2185	HG3	LYS	A	308	-12.957	-0.483	15.309	1.00	0.03	H
ATOM	2186	HD2	LYS	A	308	-13.481	-0.734	12.918	1.00	0.03	H
ATOM	2187	HD3	LYS	A	308	-14.866	-1.812	13.265	1.00	0.03	H
ATOM	2188	HE2	LYS	A	308	-16.126	-0.037	14.364	1.00	0.08	H
ATOM	2189	HE3	LYS	A	308	-14.684	0.977	14.552	1.00	0.08	H
ATOM	2190	HZ1	LYS	A	308	-16.147	0.392	11.954	1.00	0.20	H
ATOM	2191	HZ2	LYS	A	308	-15.931	1.944	12.732	1.00	0.20	H
ATOM	2192	HZ3	LYS	A	308	-14.637	1.171	11.996	1.00	0.20	H
ATOM	2193	H	LYS	A	308	-10.627	-1.095	15.452	1.00	0.19	H
ATOM	2194	N	GLN	A	309	-10.080	-4.447	15.358	1.00	-0.26	N
ATOM	2195	CA	GLN	A	309	-9.556	-5.813	15.308	1.00	0.13	C
ATOM	2196	C	GLN	A	309	-8.718	-6.142	16.527	1.00	0.20	C
ATOM	2197	O	GLN	A	309	-7.874	-7.014	16.412	1.00	-0.39	O
ATOM	2198	CB	GLN	A	309	-8.739	-6.022	14.005	1.00	0.00	C
ATOM	2199	CG	GLN	A	309	-9.577	-5.714	12.738	1.00	0.04	C
ATOM	2200	CD	GLN	A	309	-10.697	-6.676	12.458	1.00	0.17	C
ATOM	2201	OE1	GLN	A	309	-10.763	-7.734	13.066	1.00	-0.40	O
ATOM	2202	NE2	GLN	A	309	-11.609	-6.352	11.523	1.00	-0.30	N
ATOM	2203	HA	GLN	A	309	-10.377	-6.549	15.294	1.00	0.08	H
ATOM	2204	HB2	GLN	A	309	-7.871	-5.342	14.039	1.00	0.03	H
ATOM	2205	HB3	GLN	A	309	-8.368	-7.057	13.944	1.00	0.03	H
ATOM	2206	HG2	GLN	A	309	-9.999	-4.702	12.776	1.00	0.05	H
ATOM	2207	HG3	GLN	A	309	-8.897	-5.765	11.883	1.00	0.05	H
ATOM	2208	HE22	GLN	A	309	-12.357	-6.994	11.334	1.00	0.18	H
ATOM	2209	HE21	GLN	A	309	-11.557	-5.490	11.015	1.00	0.18	H
ATOM	2210	H	GLN	A	309	-9.554	-3.698	14.939	1.00	0.19	H
ATOM	2211	N	ARG	A	310	-8.890	-5.499	17.705	1.00	-0.26	N
ATOM	2212	CA	ARG	A	310	-8.052	-5.852	18.851	1.00	0.13	C
ATOM	2213	C	ARG	A	310	-6.578	-5.727	18.513	1.00	0.20	C
ATOM	2214	O	ARG	A	310	-5.771	-6.494	19.019	1.00	-0.39	O
ATOM	2215	CB	ARG	A	310	-8.425	-7.250	19.415	1.00	-0.01	C
ATOM	2216	CG	ARG	A	310	-9.894	-7.286	19.912	1.00	-0.02	C
ATOM	2217	CD	ARG	A	310	-10.308	-8.727	20.319	1.00	0.06	C
ATOM	2218	NE	ARG	A	310	-9.346	-9.269	21.287	1.00	-0.27	N
ATOM	2219	CZ	ARG	A	310	-8.611	-10.353	21.150	1.00	0.29	C
ATOM	2220	NH1	ARG	A	310	-8.640	-11.156	20.117	1.00	-0.28	N
ATOM	2221	NH2	ARG	A	310	-7.783	-10.663	22.121	1.00	-0.28	N
ATOM	2222	HA	ARG	A	310	-8.205	-5.119	19.661	1.00	0.08	H
ATOM	2223	HB2	ARG	A	310	-8.282	-8.020	18.644	1.00	0.03	H
ATOM	2224	HB3	ARG	A	310	-7.760	-7.491	20.258	1.00	0.03	H
ATOM	2225	HG2	ARG	A	310	-10.000	-6.607	20.775	1.00	0.03	H
ATOM	2226	HG3	ARG	A	310	-10.570	-6.924	19.118	1.00	0.03	H
ATOM	2227	HD2	ARG	A	310	-11.287	-8.671	20.824	1.00	0.07	H
ATOM	2228	HD3	ARG	A	310	-10.480	-9.280	19.388	1.00	0.07	H

ATOM 2229 HE ARG A 310 -9.254 -8.724 22.157 1.00 0.26 H
ATOM 2230 HH12 ARG A 310 -8.048 -12.003 20.093 1.00 0.26 H
ATOM 2231 HH11 ARG A 310 -9.247 -11.020 19.309 1.00 0.26 H
ATOM 2232 HH22 ARG A 310 -7.186 -11.501 22.067 1.00 0.26 H
ATOM 2233 HH21 ARG A 310 -7.696 -10.081 22.964 1.00 0.26 H
ATOM 2234 H ARG A 310 -9.561 -4.761 17.813 1.00 0.19 H
ATOM 2235 N GLY A 311 -6.215 -4.732 17.671 1.00 -0.27 N
ATOM 2236 CA GLY A 311 -4.809 -4.422 17.440 1.00 0.12 C
ATOM 2237 C GLY A 311 -3.861 -5.600 17.330 1.00 0.20 C
ATOM 2238 O GLY A 311 -4.088 -6.502 16.538 1.00 -0.39 O
ATOM 2239 HA3 GLY A 311 -4.695 -3.865 16.496 1.00 0.08 H
ATOM 2240 HA2 GLY A 311 -4.503 -3.746 18.253 1.00 0.08 H
ATOM 2241 H GLY A 311 -6.895 -4.122 17.245 1.00 0.19 H
ATOM 2242 N TRP A 312 -2.744 -5.601 18.092 1.00 -0.26 N
ATOM 2243 CA TRP A 312 -1.713 -6.609 17.856 1.00 0.14 C
ATOM 2244 C TRP A 312 -2.092 -7.978 18.389 1.00 0.21 C
ATOM 2245 O TRP A 312 -1.680 -8.975 17.813 1.00 -0.39 O
ATOM 2246 CB TRP A 312 -0.342 -6.103 18.374 1.00 0.00 C
ATOM 2247 CG TRP A 312 0.072 -4.942 17.509 1.00 -0.04 C
ATOM 2248 CD1 TRP A 312 0.002 -3.630 17.782 1.00 0.02 C
ATOM 2249 CD2 TRP A 312 0.639 -5.066 16.125 1.00 -0.02 C
ATOM 2250 NE1 TRP A 312 0.443 -2.944 16.758 1.00 -0.29 N
ATOM 2251 CE2 TRP A 312 0.830 -3.752 15.749 1.00 0.06 C
ATOM 2252 CE3 TRP A 312 0.959 -6.132 15.280 1.00 -0.07 C
ATOM 2253 CZ2 TRP A 312 1.345 -3.420 14.500 1.00 -0.04 C
ATOM 2254 CZ3 TRP A 312 1.475 -5.818 14.021 1.00 -0.08 C
ATOM 2255 CH2 TRP A 312 1.668 -4.482 13.638 1.00 -0.08 C
ATOM 2256 HA TRP A 312 -1.604 -6.721 16.771 1.00 0.08 H
ATOM 2257 HB2 TRP A 312 -0.415 -5.793 19.429 1.00 0.04 H
ATOM 2258 HB3 TRP A 312 0.417 -6.895 18.290 1.00 0.04 H
ATOM 2259 HE1 TRP A 312 0.481 -1.915 16.724 1.00 0.22 H
ATOM 2260 HD1 TRP A 312 -0.376 -3.205 18.715 1.00 0.08 H
ATOM 2261 HZ2 TRP A 312 1.492 -2.386 14.198 1.00 0.05 H
ATOM 2262 HH2 TRP A 312 2.074 -4.263 12.663 1.00 0.05 H
ATOM 2263 HZ3 TRP A 312 1.729 -6.618 13.328 1.00 0.05 H
ATOM 2264 HE3 TRP A 312 0.812 -7.156 15.600 1.00 0.05 H
ATOM 2265 H TRP A 312 -2.584 -4.907 18.794 1.00 0.19 H
ATOM 2266 N ASP A 313 -2.889 -8.093 19.472 1.00 -0.26 N
ATOM 2267 CA ASP A 313 -3.349 -9.427 19.855 1.00 0.14 C
ATOM 2268 C ASP A 313 -4.184 -10.002 18.728 1.00 0.21 C
ATOM 2269 O ASP A 313 -4.014 -11.166 18.395 1.00 -0.39 O
ATOM 2270 CB ASP A 313 -4.152 -9.438 21.176 1.00 0.04 C
ATOM 2271 CG ASP A 313 -3.200 -9.128 22.301 1.00 0.04 C
ATOM 2272 OD1 ASP A 313 -2.460 -10.062 22.728 1.00 -0.57 O
ATOM 2273 OD2 ASP A 313 -3.178 -7.952 22.749 1.00 -0.57 O
ATOM 2274 HA ASP A 313 -2.471 -10.078 19.998 1.00 0.08 H
ATOM 2275 HB2 ASP A 313 -4.973 -8.705 21.128 1.00 0.05 H
ATOM 2276 HB3 ASP A 313 -4.586 -10.435 21.353 1.00 0.05 H
ATOM 2277 H ASP A 313 -3.208 -7.295 19.985 1.00 0.19 H
ATOM 2278 N GLY A 314 -5.092 -9.217 18.105 1.00 -0.27 N
ATOM 2279 CA GLY A 314 -5.842 -9.766 16.975 1.00 0.12 C

ATOM 2280 C GLY A 314 -4.911 -10.224 15.877 1.00 0.20 C
ATOM 2281 O GLY A 314 -5.100 -11.301 15.334 1.00 -0.39 O
ATOM 2282 HA3 GLY A 314 -6.444 -10.620 17.321 1.00 0.08 H
ATOM 2283 HA2 GLY A 314 -6.521 -9.023 16.538 1.00 0.08 H
ATOM 2284 H GLY A 314 -5.243 -8.258 18.378 1.00 0.19 H
ATOM 2285 N PHE A 315 -3.889 -9.417 15.526 1.00 -0.26 N
ATOM 2286 CA PHE A 315 -2.892 -9.888 14.559 1.00 0.14 C
ATOM 2287 C PHE A 315 -2.306 -11.221 14.975 1.00 0.21 C
ATOM 2288 O PHE A 315 -2.240 -12.129 14.158 1.00 -0.39 O
ATOM 2289 CB PHE A 315 -1.718 -8.880 14.462 1.00 0.02 C
ATOM 2290 CG PHE A 315 -0.468 -9.443 13.783 1.00 -0.04 C
ATOM 2291 CD1 PHE A 315 -0.494 -9.754 12.421 1.00 -0.06 C
ATOM 2292 CD2 PHE A 315 0.716 -9.643 14.505 1.00 -0.06 C
ATOM 2293 CE1 PHE A 315 0.666 -10.213 11.798 1.00 -0.07 C
ATOM 2294 CE2 PHE A 315 1.907 -9.928 13.838 1.00 -0.07 C
ATOM 2295 CZ PHE A 315 1.888 -10.227 12.476 1.00 -0.07 C
ATOM 2296 HA PHE A 315 -3.366 -10.001 13.571 1.00 0.08 H
ATOM 2297 HB2 PHE A 315 -2.037 -7.969 13.939 1.00 0.05 H
ATOM 2298 HB3 PHE A 315 -1.430 -8.587 15.475 1.00 0.05 H
ATOM 2299 HD2 PHE A 315 0.721 -9.570 15.586 1.00 0.06 H
ATOM 2300 HE2 PHE A 315 2.851 -9.915 14.370 1.00 0.06 H
ATOM 2301 HZ PHE A 315 2.801 -10.475 11.951 1.00 0.06 H
ATOM 2302 HE1 PHE A 315 0.627 -10.569 10.780 1.00 0.06 H
ATOM 2303 HD1 PHE A 315 -1.413 -9.644 11.851 1.00 0.06 H
ATOM 2304 H PHE A 315 -3.805 -8.505 15.945 1.00 0.19 H
ATOM 2305 N VAL A 316 -1.857 -11.358 16.242 1.00 -0.26 N
ATOM 2306 CA VAL A 316 -1.273 -12.634 16.660 1.00 0.13 C
ATOM 2307 C VAL A 316 -2.306 -13.726 16.529 1.00 0.20 C
ATOM 2308 O VAL A 316 -2.019 -14.777 15.978 1.00 -0.39 O
ATOM 2309 CB VAL A 316 -0.785 -12.603 18.140 1.00 -0.01 C
ATOM 2310 CG1 VAL A 316 -0.448 -14.022 18.684 1.00 -0.06 C
ATOM 2311 CG2 VAL A 316 0.454 -11.684 18.297 1.00 -0.06 C
ATOM 2312 HA VAL A 316 -0.430 -12.868 15.995 1.00 0.08 H
ATOM 2313 HB VAL A 316 -1.599 -12.197 18.764 1.00 0.03 H
ATOM 2314 HG11 VAL A 316 0.335 -14.497 18.073 1.00 0.02 H
ATOM 2315 HG12 VAL A 316 -0.092 -13.955 19.722 1.00 0.02 H
ATOM 2316 HG13 VAL A 316 -1.333 -14.676 18.686 1.00 0.02 H
ATOM 2317 HG21 VAL A 316 0.295 -10.705 17.826 1.00 0.02 H
ATOM 2318 HG22 VAL A 316 0.664 -11.513 19.362 1.00 0.02 H
ATOM 2319 HG23 VAL A 316 1.328 -12.164 17.834 1.00 0.02 H
ATOM 2320 H VAL A 316 -1.953 -10.606 16.905 1.00 0.19 H
ATOM 2321 N GLU A 317 -3.519 -13.492 17.062 1.00 -0.26 N
ATOM 2322 CA GLU A 317 -4.540 -14.536 17.033 1.00 0.13 C
ATOM 2323 C GLU A 317 -4.841 -14.891 15.591 1.00 0.20 C
ATOM 2324 O GLU A 317 -4.938 -16.061 15.252 1.00 -0.39 O
ATOM 2325 CB GLU A 317 -5.767 -13.987 17.815 1.00 -0.00 C
ATOM 2326 CG GLU A 317 -6.868 -15.018 18.151 1.00 0.00 C
ATOM 2327 CD GLU A 317 -7.945 -14.355 18.973 1.00 0.04 C
ATOM 2328 OE1 GLU A 317 -7.616 -13.437 19.772 1.00 -0.57 O
ATOM 2329 OE2 GLU A 317 -9.137 -14.725 18.837 1.00 -0.57 O
ATOM 2330 HA GLU A 317 -4.151 -15.427 17.550 1.00 0.08 H

ATOM	2331	HB2	GLU	A	317	-5.397	-13.596	18.778	1.00	0.03	H
ATOM	2332	HB3	GLU	A	317	-6.226	-13.160	17.251	1.00	0.03	H
ATOM	2333	HG2	GLU	A	317	-7.310	-15.401	17.222	1.00	0.04	H
ATOM	2334	HG3	GLU	A	317	-6.443	-15.857	18.723	1.00	0.04	H
ATOM	2335	H	GLU	A	317	-3.722	-12.593	17.469	1.00	0.19	H
ATOM	2336	N	PHE	A	318	-4.982	-13.877	14.713	1.00	-0.26	N
ATOM	2337	CA	PHE	A	318	-5.305	-14.143	13.313	1.00	0.14	C
ATOM	2338	C	PHE	A	318	-4.304	-15.058	12.639	1.00	0.21	C
ATOM	2339	O	PHE	A	318	-4.736	-15.952	11.927	1.00	-0.39	O
ATOM	2340	CB	PHE	A	318	-5.406	-12.805	12.533	1.00	0.02	C
ATOM	2341	CG	PHE	A	318	-5.749	-13.041	11.057	1.00	-0.04	C
ATOM	2342	CD1	PHE	A	318	-7.044	-13.421	10.685	1.00	-0.06	C
ATOM	2343	CD2	PHE	A	318	-4.775	-12.875	10.066	1.00	-0.06	C
ATOM	2344	CE1	PHE	A	318	-7.363	-13.617	9.339	1.00	-0.07	C
ATOM	2345	CE2	PHE	A	318	-5.090	-13.067	8.719	1.00	-0.07	C
ATOM	2346	CZ	PHE	A	318	-6.389	-13.434	8.352	1.00	-0.07	C
ATOM	2347	HA	PHE	A	318	-6.285	-14.645	13.284	1.00	0.08	H
ATOM	2348	HB2	PHE	A	318	-6.193	-12.173	12.977	1.00	0.05	H
ATOM	2349	HB3	PHE	A	318	-4.452	-12.259	12.623	1.00	0.05	H
ATOM	2350	HD2	PHE	A	318	-3.766	-12.595	10.342	1.00	0.06	H
ATOM	2351	HE2	PHE	A	318	-4.327	-12.934	7.956	1.00	0.06	H
ATOM	2352	HZ	PHE	A	318	-6.639	-13.579	7.306	1.00	0.06	H
ATOM	2353	HE1	PHE	A	318	-8.371	-13.913	9.056	1.00	0.06	H
ATOM	2354	HD1	PHE	A	318	-7.808	-13.566	11.443	1.00	0.06	H
ATOM	2355	H	PHE	A	318	-4.874	-12.927	15.021	1.00	0.19	H
ATOM	2356	N	PHE	A	319	-2.975	-14.888	12.832	1.00	-0.26	N
ATOM	2357	CA	PHE	A	319	-2.021	-15.849	12.261	1.00	0.14	C
ATOM	2358	C	PHE	A	319	-1.568	-16.850	13.307	1.00	0.21	C
ATOM	2359	O	PHE	A	319	-0.407	-17.233	13.303	1.00	-0.39	O
ATOM	2360	CB	PHE	A	319	-0.772	-15.128	11.694	1.00	0.02	C
ATOM	2361	CG	PHE	A	319	-1.056	-14.297	10.444	1.00	-0.04	C
ATOM	2362	CD1	PHE	A	319	-1.219	-14.929	9.204	1.00	-0.06	C
ATOM	2363	CD2	PHE	A	319	-1.132	-12.902	10.518	1.00	-0.06	C
ATOM	2364	CE1	PHE	A	319	-1.391	-14.177	8.043	1.00	-0.07	C
ATOM	2365	CE2	PHE	A	319	-1.262	-12.152	9.350	1.00	-0.07	C
ATOM	2366	CZ	PHE	A	319	-1.409	-12.783	8.112	1.00	-0.07	C
ATOM	2367	HA	PHE	A	319	-2.456	-16.437	11.440	1.00	0.08	H
ATOM	2368	HB2	PHE	A	319	-0.381	-14.500	12.502	1.00	0.05	H
ATOM	2369	HB3	PHE	A	319	0.021	-15.839	11.406	1.00	0.05	H
ATOM	2370	HD2	PHE	A	319	-1.092	-12.404	11.484	1.00	0.06	H
ATOM	2371	HE2	PHE	A	319	-1.243	-11.071	9.392	1.00	0.06	H
ATOM	2372	HZ	PHE	A	319	-1.538	-12.197	7.214	1.00	0.06	H
ATOM	2373	HE1	PHE	A	319	-1.508	-14.671	7.082	1.00	0.06	H
ATOM	2374	HD1	PHE	A	319	-1.209	-16.013	9.143	1.00	0.06	H
ATOM	2375	H	PHE	A	319	-2.641	-14.157	13.438	1.00	0.19	H
ATOM	2376	N	HIS	A	320	-2.451	-17.298	14.223	1.00	-0.26	N
ATOM	2377	CA	HIS	A	320	-1.999	-18.211	15.272	1.00	0.14	C
ATOM	2378	C	HIS	A	320	-1.334	-19.472	14.749	1.00	0.20	C
ATOM	2379	O	HIS	A	320	-0.294	-19.817	15.288	1.00	-0.39	O
ATOM	2380	CB	HIS	A	320	-3.196	-18.643	16.160	1.00	0.04	C
ATOM	2381	CG	HIS	A	320	-2.820	-19.670	17.189	1.00	0.06	C

```
ATOM   2382  ND1 HIS A 320      -1.897 -19.495  18.107  1.00 -0.26           N
ATOM   2383  CD2 HIS A 320      -3.370 -20.900  17.323  1.00 -0.03           C
ATOM   2384  CE1 HIS A 320      -1.808 -20.548  18.847  1.00  0.09           C
ATOM   2385  NE2 HIS A 320      -2.638 -21.401  18.457  1.00 -0.34           N
ATOM   2386  HA  HIS A 320      -1.265 -17.672  15.892  1.00  0.08           H
ATOM   2387  HB2 HIS A 320      -3.603 -17.767  16.685  1.00  0.05           H
ATOM   2388  HB3 HIS A 320      -3.991 -19.052  15.517  1.00  0.05           H
ATOM   2389  HD2 HIS A 320      -4.151 -21.378  16.738  1.00  0.03           H
ATOM   2390  HE1 HIS A 320      -1.128 -20.705  19.691  1.00  0.11           H
ATOM   2391  HD1 HIS A 320      -1.319 -18.652  18.221  1.00  0.24           H
ATOM   2392  H   HIS A 320      -3.398 -16.963  14.237  1.00  0.19           H
ATOM   2393  N   VAL A 321      -1.940 -20.152  13.746  1.00 -0.29           N
ATOM   2394  CA  VAL A 321      -1.502 -21.484  13.307  1.00  0.01           C
ATOM   2395  C   VAL A 321      -1.256 -22.402  14.477  1.00 -0.19           C
ATOM   2396  O   VAL A 321      -1.640 -23.563  14.464  1.00 -0.72           O
ATOM   2397  CB  VAL A 321      -0.316 -21.498  12.295  1.00 -0.03           C
ATOM   2398  CG1 VAL A 321       1.039 -21.094  12.934  1.00 -0.06           C
ATOM   2399  CG2 VAL A 321      -0.173 -22.903  11.641  1.00 -0.06           C
ATOM   2400  HA  VAL A 321      -2.360 -21.939  12.786  1.00  0.05           H
ATOM   2401  HB  VAL A 321      -0.550 -20.777  11.495  1.00  0.03           H
ATOM   2402 HG11 VAL A 321       1.265 -21.731  13.801  1.00  0.02           H
ATOM   2403 HG12 VAL A 321       1.858 -21.198  12.207  1.00  0.02           H
ATOM   2404 HG13 VAL A 321       1.012 -20.048  13.259  1.00  0.02           H
ATOM   2405 HG21 VAL A 321      -1.108 -23.199  11.141  1.00  0.02           H
ATOM   2406 HG22 VAL A 321       0.628 -22.907  10.887  1.00  0.02           H
ATOM   2407 HG23 VAL A 321       0.065 -23.661  12.403  1.00  0.02           H
ATOM   2408  H   VAL A 321      -2.750 -19.762  13.313  1.00  0.19           H
TER    2409      VAL A 321
HETATM 2410  O   DRG A 401       3.815  -8.209  -2.838  1.00 -0.38           O
HETATM 2411  C   DRG A 401       2.922  -7.400  -3.048  1.00  0.21           C
HETATM 2412  C15 DRG A 401       3.288  -6.103  -3.767  1.00  0.09           C
HETATM 2413  H5  DRG A 401       2.395  -5.620  -4.187  1.00  0.07           H
HETATM 2414  C23 DRG A 401       3.999  -5.046  -2.935  1.00 -0.02           C
HETATM 2415  C18 DRG A 401       5.144  -4.584  -3.593  1.00 -0.04           C
HETATM 2416  C17 DRG A 401       5.246  -5.120  -4.998  1.00 -0.02           C
HETATM 2417  C16 DRG A 401       4.305  -6.356  -4.924  1.00 -0.02           C
HETATM 2418  H7  DRG A 401       4.944  -7.213  -4.665  1.00  0.03           H
HETATM 2419  H6  DRG A 401       3.803  -6.583  -5.878  1.00  0.03           H
HETATM 2420  H9  DRG A 401       6.295  -5.338  -5.257  1.00  0.04           H
HETATM 2421  H8  DRG A 401       4.857  -4.353  -5.687  1.00  0.04           H
HETATM 2422  C19 DRG A 401       6.041  -3.717  -2.958  1.00 -0.07           C
HETATM 2423  H11 DRG A 401       6.979  -3.481  -3.429  1.00  0.05           H
HETATM 2424  C20 DRG A 401       5.690  -3.183  -1.720  1.00 -0.06           C
HETATM 2425  H12 DRG A 401       6.330  -2.428  -1.263  1.00  0.05           H
HETATM 2426  C21 DRG A 401       4.532  -3.607  -1.083  1.00  0.03           C
HETATM 2427 CL13 DRG A 401       4.202  -2.987   0.518  1.00 -0.08          CL
HETATM 2428  C22 DRG A 401       3.657  -4.504  -1.690  1.00 -0.05           C
HETATM 2429  H14 DRG A 401       2.719  -4.779  -1.220  1.00  0.05           H
HETATM 2430  N   DRG A 401       1.615  -7.604  -2.730  1.00 -0.25           N
HETATM 2431  H1  DRG A 401       0.936  -6.902  -2.939  1.00  0.22           H
HETATM 2432  C04 DRG A 401       1.087  -8.826  -2.208  1.00 -0.01           C
```

```
HETATM 2433  C14 DRG A 401       1.825  -9.740  -1.471  1.00 -0.09           C
HETATM 2434  C08 DRG A 401       1.223 -10.959  -1.084  1.00  0.04           C
HETATM 2435  C07 DRG A 401      -0.119 -11.173  -1.367  1.00 -0.11           C
HETATM 2436  N06 DRG A 401      -0.842 -10.250  -2.037  1.00 -0.51           N
HETATM 2437  C05 DRG A 401      -0.271  -9.107  -2.454  1.00 -0.11           C
HETATM 2438  H3  DRG A 401      -0.612 -12.089  -1.052  1.00  0.01           H
HETATM 2439  O09 DRG A 401       1.971 -11.937  -0.419  1.00 -0.18           O
HETATM 2440  S10 DRG A 401       2.459 -13.031  -1.331  1.00  0.19           S
HETATM 2441  O13 DRG A 401       1.391 -13.505  -2.188  1.00 -0.13           O
HETATM 2442  O12 DRG A 401       3.231 -13.975  -0.548  1.00 -0.13           O
HETATM 2443  H4  DRG A 401       2.849  -9.554  -1.167  1.00 -0.02           H
CONECT  830  828  829 2440
CONECT 2410 2411
CONECT 2411 2410 2412 2430
CONECT 2412 2411 2413 2414 2417
CONECT 2413 2412
CONECT 2414 2412 2415 2428
CONECT 2415 2414 2416 2422
CONECT 2416 2415 2417 2420 2421
CONECT 2417 2412 2416 2418 2419
CONECT 2418 2417
CONECT 2419 2417
CONECT 2420 2416
CONECT 2421 2416
CONECT 2422 2415 2423 2424
CONECT 2423 2422
CONECT 2424 2422 2425 2426
CONECT 2425 2424
CONECT 2426 2424 2427 2428
CONECT 2427 2426
CONECT 2428 2414 2426 2429
CONECT 2429 2428
CONECT 2430 2411 2431 2432
CONECT 2431 2430
CONECT 2432 2430 2433 2437
CONECT 2433 2432 2434 2443
CONECT 2434 2433 2435 2439
CONECT 2435 2434 2436 2438
CONECT 2436 2435 2437
CONECT 2437 2432 2436
CONECT 2438 2435
CONECT 2439 2434 2440
CONECT 2440  830 2439 2441 2442
CONECT 2441 2440
CONECT 2442 2440
CONECT 2443 2433
MASTER        0    0    0    0    0    0    0    0 2442    1   35   12
END
```
